# Abstracts of scientific contributions to GCOM 2019: Berlin, Germany. 27 - 30 March 2019

**DOI:** 10.1186/s41927-019-0078-3

**Published:** 2019-08-19

## I1 Selected highlights from the 3rd Global Conference of Myositis, Berlin

### Hector Chinoy^1,2,3^, James B. Lilleker^3,4^

#### ^1^National Institute for Health Research Manchester Biomedical Research Centre, Manchester University NHS Foundation Trust, The University of Manchester (Manchester, UK); ^2^Department of Rheumatology, Salford Royal NHS Foundation Trust, Manchester Academic Health Science Centre (Salford, UK); ^3^Centre for Musculoskeletal Research, Faculty of Biology, Medicine and Health, The University of Manchester (Manchester, UK); ^4^Manchester Centre for Clinical Neurosciences, Salford Royal NHS Foundation Trust (Salford, UK)

##### **Correspondence:** Hector Chinoy (hector.chinoy@manchester.ac.uk)

March 2019 saw the 3rd Global Conference of Myositis at the historical European School of Management and Technology, Berlin, Germany, organized by Professors Werner Stenzel, Olivier Benveniste, Ichizo Nishino, and the Scientific and Organising Committee. The conference was well attended by 375 delegates from 30 different countries, including 20 invited speakers,

A opportunity arose to fund a series of fellowships to junior researchers, from funds left over from the previous GCOM meeting at Potomac. There was a series of wonderful and enlightening talks from all over the world and from neighbouring disciplines.

The conference started off with interesting pathology workshop detailing difficult cases. There then followed an introductory lecture by Voit on recent developments in the therapeutic approaches to neuromuscular diseases, including antisense therapy. Regulators are increasingly willing to accept ‘contemporary controls’ selected according to the same entry criteria to strengthen small placebo controls groups and improve power to detect change where short-term effect sizes are small. Discussion was also made about using myostatin, an activin 2b receptor binder, as a disease progression biomarker. Decreased levels of myostatin and downregulation of the myostatin-activin pathway are seen in neuromuscular disease, potentially also explaining the paucity of therapeutic benefits seen to date from myostatin receptor blockade.

Kjaer discussed how age impairs the performance of the skeletal muscle and cardiovascular system. Three to six months of training can result in a 10-15% increase in strength/function, akin to 3-5 years of rejuvenation. Ageing can affect the transcriptional regulation of human skeletal muscle disuse atrophy, with the result that older people require four times as much time to recover from periods of immobility (1). This is associated with reduced anabolic signaling, and a decrease in number and responsiveness of satellite cells.

Inclusion body myositis (IBM) was the subject of a number of talks. Wiehl discussed pathogenic mechanisms connecting inherited and acquired IBM, including disruptions in protein homeostasis and autophagy which have recently been interrogated using a targetted proteomic approach. (2). Britson described a xenograft model of sporadic IBM which shows impaired regeneration and TDP43 mislocalization. Greenberg discussed a potential therapeutic strategy of targeting highly differentiated T cells, particularly effector memory KLRG1+ cytotoxic T cells in IBM. There appears to be overlapping features between IBM and T cell large granular lymphocytic leukaemia, a malignancy of highly differentiated CD8 cytotoxic T cells which also exhibit upregulation of KLRG1. (3)

Immune checkpoint inhibitors are increasingly being used to activate the immune system against malignant cells, particularly melanoma, but they can have autoimmune adverse effects, including myositis. Moslehi discussed his report in the Lancet where they identified 101 cases of severe myocarditis following treatment with immune checkpoint inhibitors (4). Of relevance to myologists, a substantial increase in incidence over time was noted.

Klareskog gave an eloquent overview of the role of environmental factors, focusing on the lungs as a trigger of autoimmunity. He cited the analogy of rheumatoid arthritis, where a combination of genetics, epidemiological risk factors (including smoking, hormones, stress and the HPA axis), systemic immune dysregulation and disruption of mucosal biology create an at-risk population for development of autoimmunity (5). Smoking may predispose to antigenic peptide fragments being presented on the cell surface by HLA class II molecules (forming part of the common ancestral haplotype), and interact with autoaggressive CD4+ cells, for the subsequent development of anti-aminoacyl tRNA synthetase antibodies (6).

The association of cancer and dermatomyositis was also a theme. Dani described register data from 2002 to 2016 in Sweden. Cancer risk in IIM patients is increased before diagnosis, reaches a peak one year pre-diagnosis, and continues to be significant until 10 years post-diagnosis. Site-specific cancer risk is highest for respiratory before diagnosis and the buccal cavity after. Even patients with IIM diagnoses other than DM are at risk of developing cancer. Oldroyd also described a temporal relationship with cancer and DM (7). Specifically, anti-TIF1gamma-positive-associated malignancy occurs exclusively within the three year period on either side of DM onset, the risk being highest in those 39 years or younger. Within the anti-TIF1gamma-positive cohort, breast cancer was the most common malignancy (33%), followed by ovarian cancer (19%) and lymphoma (14%). Ovarian cancer constituted a significantly higher proportion of cancers in the anti-TIF1gamma-positive vs. -negative cases.

It appears that possession of a specific anti-TIF1gamma IgG subtype is necessary to incur risk of cancer. A poster from Aussy (8) examined 51 anti-TIF1gamma -positive patients, where 40 (78%) had cancer. Detection of anti-TIF1gamma IgG2 was significantly associated with mortality (p=0.0011) and occurrence of cancer during the follow-up period, with a 100% positive predictive value of cancer when then mean fluorescence intensity of anti-TIF1gamma IgG2 was >385.

Patients with anti-MDA5 antibody-associated disease represent a challenging group where disease is often resistant to treatment and mortality is high. Toquet described a cohort of 121 anti-MDA5 antibody-associated DM, where three subgroups with different outcomes were identified. Overall mortality was 27%. Cluster one had less arthralgia/arthritis, but all had interstitial lung disease and there was a high frequency of mechanics’ hands. Cluster two, the “athro-cutaneo”-form, tended to have more arthralgia/arthritis, were less likely to be male and had a better prognosis. Cluster three, the “vasculo-cutaneo-muscular”-form, were more likely to be male, have skin vasculopathy (Raynaud’s, skin ulcers, digital necrosis, calcinosis), myositis and had an intermediate prognosis. Gono discussed an intensive treatment combination used for anti-MDA5 antibody-associated interstitial lung disease (9). This included high dose prednisolone, cyclosporine and intravenous cyclophosphamide. Peng discussed neopterin as a marker of macrophage activation. Increased neopterin levels have been noted in DM and in patients with MDA5. There was a positive correlation of neopterin with ferritin and markers of disease severity, and negative correlation with pulmonary function.

Paik presented results of an open label study of the JAK inhibitor, Tofacinitib, in refractory DM, testing the hypothesis that the drug would inhibit the production or secretion of key pro-inflammatory chemokines e.g. CXCL9/10 to induce clinical remission (10). All 10 patients enrolled met the IMACS definition of improvement. Five of seven anti-TIF1gamma positive patients were moderate responders, and three of five were able to come off steroids.

Generous financial support by Myositis UK formed the basis for a novel “speed-funding” event, where 17 grant applications were initially considered. Six junior researchers were then put forward to present a 15 minute funding proposal to a set of judges and the audience. Amidst a high-pressure atmosphere, the following were successful in securing funding: Kyla Britson, USA: The pathogenesis and treatment of inclusion body myositis in a xenograft model; Saskia Lassche, Netherlands; Inclusion body myositis on a chip, Erin Wilfong, USA; CD27‐CXCR4hiCD21lo cells in Jo1 positive IIM.

Other highlights included a workshop for young researchers, a patient-centred workshop and further discussion about the establishment of a World Myositis Society. We now look forward now to the 4th GCOM which will provisionally be held in Prague, Czech Republic, in 2021.

**Sources of Funding:**

HC is supported by a grant from the Medical Research Council (MR/N003322/1) and the NIHR Biomedical Research Centre Funding Scheme. The views expressed in this publication are those of the authors and not necessarily those of the NHS, the National Institute for Health Research or the Department of Health.

**References**

1. Suetta C, Frandsen U, Jensen L, Jensen MM, Jespersen JG, Hvid LG, et al. Aging affects the transcriptional regulation of human skeletal muscle disuse atrophy. Calbet JAL, ed. PLoS One 2012;7:e51238.

2. Güttsches A-K, Brady S, Krause K, Maerkens A, Uszkoreit J, Eisenacher M, et al. Proteomics of rimmed vacuoles define new risk allele in inclusion body myositis. Ann Neurol 2016.

3. Greenberg SA, Pinkus JL, Amato AA, Kristensen T, Dorfman DM. Association of inclusion body myositis with T cell large granular lymphocytic leukaemia. Brain 2016;139:1348–60.

4. Moslehi JJ, Salem J-E, Sosman JA, Lebrun-Vignes B, Johnson DB. Increased reporting of fatal immune checkpoint inhibitor-associated myocarditis. Lancet (London, England) 2018;391:933.

5. Holers VM, Demoruelle MK, Kuhn KA, Buckner JH, Robinson WH, Okamoto Y, et al. Rheumatoid arthritis and the mucosal origins hypothesis: protection turns to destruction. Nat Rev Rheumatol 2018;14:542–557.

6. Chinoy H, Adimulam S, Marriage F, New P, Vincze M, Zilahi E, et al. Interaction of HLA-DRB1*03 and smoking for the development of anti-Jo-1 antibodies in adult idiopathic inflammatory myopathies: a European-wide case study. Ann Rheum Dis 2012;71:961–5.

7. Oldroyd A, Sergeant JC, New P, McHugh NJ, Betteridge Z, Lamb JA, et al. The temporal relationship between cancer and adult onset anti-transcriptional intermediary factor 1 antibody–positive dermatomyositis. Rheumatology 2018.

8. Aussy A, Fréret M, Gallay L, Bessis D, Vincent T, Jullien D, et al. The IgG2 isotype of anti-transcription intermediary factor 1-gamma autoantibodies is a biomarker of mortality in adult dermatomyositis. Arthritis Rheumatol (Hoboken, NJ) 2019:art.40895.

9. Nakashima R, Hosono Y, Mimori T. Clinical significance and new detection system of autoantibodies in myositis with interstitial lung disease. Lupus 2016;25:925–933.

10. Paik J, Albayda J, Tiniakou E, Koenig A, Christopher-Stine L. Study of Tofacitinib in Refractory Dermatomyositis (STIR): An Open Label Pilot Study in Refractory Dermatomyositis - ACR Meeting Abstracts. Arthritis Rheum 2018;70:L02.

**Oral Presentation**

O1 Xenograft model of sporadic inclusion body myositis

*K. Britson*

O2 Targeting highly differentiated effector memory KLRG1+ cytotoxic T cells in inclusion body myositis

*S. Greenberg*

O3 CD8+T-bet+ cells as a predominant biomarker for inclusion body myositis

*G. Dzangué-Tchoupou*

O4 Overall and site-specific cancer risk before and after diagnosis of idiopathic inflammatory myopathies: Novel register data from 2002 to 2016 in Sweden

*L. Dani*

O5 Immunization of dermatomyositis-specific auto-antigen transcriptional intermediary factor (TIF1)-γ induces myositis in mice

*N. Okiyama*

O6 The temporal relationship between cancer and adult onset anti-transcriptional intermediary factor 1 antibody positive dermatomyositis

*A. Oldroyd*

O7 Pruritonegic mediators in skin samples of patients with dermatomyositis

*A. Vincze*

O8 High level of serum neopterin is associated with rapidly progressive interstitial lung disease and reduced survival indermatomyositis

*Q. Peng*

O9 Anti-MDA5 antibody-associated dermatomyositis: Three subgroups with different outcomes

*S. Toquet*

O10 Risk stratification in patients with anti-melanoma differentiation-associated gene 5 antibody-associated interstitial lung disease treated initially with intensive combination regimen

*T. Gono*

O11 Quantitative high throughput screening of small molecule libraries to inhibit interferon beta-stimulated major histocompatibility complex class I in myositismuscle

*T. Kinder*

O12 Study of Tofacinitib in refractory dermatomyositis

*J. Paik*

O13 A one-year study investigating the effect of specialised and intensive ADL training in patients with idiopathic inflammatory myopathies -preliminary results

*M. Špiritović*

O14 The use of metabolomics to develop novel biomarkers for juvenile dermatomyositis

*J. Dvergsten*

O15 Endothelial biomarker profiles reflect heterogeneity of juvenile dermatomyositisand may predict disease course

*J. Wienke*

O16 The relationship of pain, fatigue and emotional distress with quality of life in juvenile myositis

*K. Ardalan*

O17 Juvenile dermatomyositis – data of a national pediatric rheumatology database

*C. Sengler*

O18 Mitochondrial dysfunction: A novel treatment focus in juvenile dermatomyositis

*M. Wilkinson*

O19 Differential peripheral IFN score expression in juvenile dermatomyositis vs. Mendelian interferonopathies

*H. Kim*

O20 Juvenile inflammatory myopathy with anti-MDA5 auto-antibodies: A specific subgroup demonstrating differentially enhanced upregulation of interferon-alpha signaling

*I. Melki*

O21 Identification of six dermatomyositis subgroups using principal component analysis-based cluster analysis

*H. Zhu*

O22 Overexpression of type 1 IFN in skeletal muscle affects muscle function

*M. Morales*

O23 The role of low-density granulocytes (LDGs) and neutrophil extracellular traps (NETs) in the clinical profile of patients with idiopathic inflammatory myopathies

*J. Torres-Ruiz*

O24 Auto-antibodies targeting components of sarcolemma membrane repair: A pathogenic mechanism in inflammatory myopathies

*W. Jarjour*

O25 Investigating chaperone-mediated selective autophagy in immune-mediated necrotizing myopathies

*N. Fischer*

O26 Severe axial and pelvifemoral muscle damage in immune-mediated necrotizing myopathy evaluated by whole-body MRI

*O. Landon-Cardinal*

O27 Role of MircoRNAs in immune-mediated necrotizing myopathy through regulation of myogenic differentiation-associated genes

*Y. Zuo*

O28 The interferon gene signature in dermatomyositis, the anti-synthetase syndrome, immune-mediated necrotizing myopathy, and inclusion body myositis: A comparative study

*I. Pinal-Fernandez*

O29 Evaluation of a novel particle-based assay for detection of myositis specific antibodies

*K. Malyavantham*

O30 Optimization of muscle NMR imaged segmentation to best discriminate disease progression in adult patients with inflammatory myopathies

*H. Reyngoudt*

O31 Identification of novel auto-antibodies for idiopathic inflammatory myopathies using human proteome microarray

L. Li

O32 IFN level assessed by ultrasensitive detection technology in myositis patients: A promising biomarker of disease activity in dermatomyositis and anti-synthetase syndrome

*L. Bolko*

O33 Analysis of muscle transcriptomic features in idiopathic inflammatorymyopathies

*H. Chen*

## Poster Presentation

### P1 Autoantibody to transcriptional intermediary factor-1β as myositis-specific antibody: clinical correlation with CADM or DM with mild myopathy

#### Yasuhito Hamaguchi, Ikuko Ueda-Hayakawa, Kazuhiko Takehara, Manabu Fujimoto

##### Kanazawa University, Kanazawa, Japan

###### **Correspondence:** Yasuhito Hamaguchi

Background: Myositis-specific autoantibodies (MSAs) are associated with unique clinical subsets in polymyositis/dermatomyositis (PM/DM). Autoantibodies (autoAbs) against transcriptional intermediary factor (TIF) 1α and TIF1γ are known to be MSAs. We reported that TIF1β Abs are also targeted in DM patients with or without concomitant anti-TIF1α/γ Abs. Objectives. We evaluated the clinical features of seven cases with anti-TIF1β Abs alone.

Methods: Anti-TIF1β Abs were identified by protein immunoprecipitation assay and western blotting was performed to confirm autoAbs reactivity against TIF1β. The clinical characteristics of patients with anti-TIF1β Abs alone were examined.

Results: Anti-TIF1β Abs were identified by immunoprecipitation assay in 24 cases. Among them, seven patients were positive for anti-TIF1β Abs alone. Six out of seven patients were classified as DM. Among the six DM cases, two patients had no muscle weakness and normal CK levels, and were classified as clinically amyopathic DM. Four patients had muscle weakness, but 3 of them had normal serum CK levels that responded well to systemic corticosteroids. Characteristic DM skin rashes, such as Gottron’s sign, periungual erythema, punctate hemorrhage on the perionychium, and facial erythema including heliotrope were observed in 86%, 57%, 86%, 71% of our cases, respectively. One out of seven patients had appendiceal cancer. None of the patients had interstitial lung disease.

Conclusion: Seven patients were confirmed to have anti-TIF1β Abs without any other MSAs, including TIF1α/γ Abs, and six of them were diagnosed with DM. Anti-TIF1β Abs are one of the MSAs that is associated with clinically amyopathic DM or DM with mild myopathy.

### P2 Laboratory diagnostics of autoantibodies in autoimmune myopathies

#### Miriam Mende, Wolfgang Meyer, Thomas Scheper

##### Euroimmun AG; Lübeck, Germany

###### **Correspondence:** Miriam Mende

Objectives: Idiopathic inflammatory myopathies (IIM) are rare autoimmune diseases of skeletal muscle. The main forms are polymyositis (PM), dermatomyositis (DM), necrotizing myopathy (NM) and sporadic inclusion body myositis (sIBM). IIM are characterised by a diverse range of autoantibodies. Their specificities provide an indication of the disease subform. Antibodies against Jo-1, PL-7, PL-12, EJ, and OJ are characteristic of PM, antibodies against SRP and HMGCR are indicative for NM, while those against Mi-2alpha, Mi-2beta, SAE1, NXP2, MDA5 and TIF1gamma occur in DM. The only known serum marker for sIBM is anti-cN1A. Antibodies against PM-Scl75, PM-Scl100 and Ku indicate an overlap syndrome, in particular with the autoimmune connective tissue disease systemic sclerosis. Some antibodies, for example anti-MDA5, -PL7, -PL12, -OJ and -EJ, are associated with an increased risk of interstitial lung disease. Positivity for myositis autoantibodies can also be the first indicator of an underlying tumour (e.g. TIF1gamma). The combined frequency of all autoantibodies in myositis amounts to around 60%, whereby there is virtually no overlap in the occurrence of the different antibodies. Therefore, comprehensive multiparametric testing is essential to maximise the diagnostic information obtained from the analysis. In suspected cases of PM/DM, patient sera are serologically investigated using an indirect immunofluorescence test (IIFT) on a substrate combination of HEp-2 cells and primate liver, with confirmation of results by monospecific tests. Since antibodies against cytoplasmic antigens are sometimes not clearly detectable with IIFT, parallel performance of the screening and confirmatory test is recommended. Multiparametric immunoblots are an ideal confirmatory method, as they enable monospecific and simultaneous detection of many different antibodies.

Methods: A total of 804 sera from myositis patients and 786 control sera were investigated using a line immunoblot containing 16 antigens to detect the most relevant myositis-specific and -associated autoantibodies.

Results: The antibody prevalences obtained in the myositis patients ranged from 1% (anti-EJ, -OJ) to 21% (anti-Jo-1), while the specificities for the individual antigens amounted to 97-100%. In another study, a novel Anti-cN-1A ELISA provided a diagnostic sensitivity for sIBM of 35-39% at a specificity of 96%.

Conclusion: Diagnosis of IIM is challenging due to rarity of the diseases, the varying clinical presentation and the possibility of overlap syndromes. The determination of myositis-specific and myositis-associated autoantibodies can significantly reduce the time to diagnosis, with multiparametric testing ensuring the highest serological detection rate. Immunoblots are ideal for multiplex confirmatory testing as they offer broad antigen combinations, easy interpretation and full automatability.

### P3 Comparison of autoantibody specificities tested by a line blot assay and immunoprecipitation-based algorithm in patients with idiopathic inflammatory myopathies

#### Hector Fabricio Espinosa-Ortega^1^, Marie Holmqvist^1^, Helene Alexanderson^1^, Helena Storfors^2^, Tsuneyo Mimori^3^, Ingrid E. Lundberg^1^, Johan Rönnelid^2^

##### ^1^Karolinska Institute, Stockholm, Sweden; ^2^Uppsala University Hospital, Uppsala, Sweden; ^3^Kyoto University, Kyoto, Japan

###### **Correspondence:** Hector Fabricio Espinosa-Ortega

To date, there is no standardized approach of myositis specific autoantibodies/Myositis associated autoantibodies (MSA/MAA) testing to be used in clinical settings. Immunoprecipitation (IP) of RNA’s with silver staining and/or protein IP on cellular lysates is considered the gold standard for most autoantibodies but is time-consuming, does not differentiate antibodies targeting proteins with the same molecular weight, and does not measure levels of autoantibodies and is not routinely available in clinical settings. Line blot assays (LB) represent a faster and semi-quantitative option to detect autoantibodies.

Objectives: We aimed to compare the performance of a line blot (LB) and immunoprecipitation (IP) assays and to describe clinical associations to autoantibody specificities in a cohort of patients classified as idiopathic inflammatory myopathies (IIM). 119 patients were tested by a commercial LB (Euroline Myositis Antigen Profile 4, Euroimmun, Germany) and a combination of protein- and RNA- IP together with an anti-MDA5 ELISA. Agreement between assays was calculated by Cohen´s kappa coefficient.

Results: The overall concordance was 76% with moderate agreement (k:0.49). Agreement was very good for anti-SRP (k: 0.85) and anti-Ku (k: 0.85), good for anti-Jo-1 (k: 0.69) and moderate for anti-PmScl (k:0.58), anti-MDA5 (k:0.49), anti-TIF1gamma (0.57). Jo-1 LB+ IIM had interstitial lung disease and arthritis compared to LB-negative (p < 0.001). Nineteen % of DM/PM patients were Jo-1+ compared to 0% of IBM (p < 0.001). Anti-TIF1gamma+ patients had higher frequency of DM skin rash than patients with other specificities (p = 0.003). LB-Mi-2+ was more common compared to IP+.

Conclusion: In conclusion, the concordance rate between the two assays for detection of myositis autoantibodies was moderate to very good for SRP and most of prevalent specificities. The LB assay seems to be valid and useful to identify subgroups of IIM with specific clinical features.

### P4 The RIG-I pathway is involved in lymphopenia in patients with dermatomyositis

#### Lu Zhang, Qisheng Xia, Wenli Li, Qinglin Peng, Xin Lu, Guochun Wang

##### China-Japan Friendship Hospital, Beijing, China

###### **Correspondence:** Lu Zhang

Objective: To determine retinoic acid-inducible gene I (RIG-I) expression levels in peripheral lymphocytes and to explore the mechanism of RIG-I in the development of lymphopenia in patients with dermatomyositis (DM).

Methods: The mRNA and protein expression levels of the RIG-I were determined in peripheral T lymphocytes of 26 treatment-naive DM patients by q-PCR and Western blot, respectively. The associations between RIG-I expression levels and clinical characteristics were investigated. We examined the relationship between RIG-I expression and cell apoptosis by RIG-I overexpression or knockout in Jurkat cells. Cell apoptosis was assessed by flow cytometry. Fas and Caspase 3 were identified by Western blot. CCK8 colorimeter was performed to monitor cell proliferation.

Results: The average lymphocyte count decreased significantly in DM compared with that in the normal control group (1.03±0.58×109 vs 2.14±0.43×109, P=0.000). Lymphopenia (0.64±0.23×109) was noted in 14 patients. The ratio of apoptosis cell from DM patients were increased significantly than healthy control (8.53±2.47 vs 5.61±1.44, P=0.002) and significantly correlated with peripheral lymphocyte count(r=-0.595, P=0.032).RIG-I expression gene and protein levels were significantly higher in peripheral T lymphocytes of DM patients than those in control subjects (0.091 ± 0.051 vs 0.052 ± 0.024; p=0.011, 0.30 ± 0.18 vs 0.18 ± 0.08; p=0.005, respectively). The gene and protein expression levels of RIG-I correlated negatively with the lymphocyte count(r=-0.478, p=0.014; r=-0,517, p=0.007, respectively). RIG-I protein expression decreased significantly and the number of lymphocytes increased when disease was improved. In Jurkat cells, increased apoptosis were observed following RIG-I overexpression (9.13 ± 1.63 vs 0.57 ± 0.31; p=0.001) or RIG-I specific ligand (pppRNA) 12 and 24 hours activation (4.07 ± 0.21 vs 0.53 ± 0.15; p=0.000, 11.03 ± 1.70 vs 2.53 ± 0.59; p=0.001, respectively). The increased apoptosis also be confirmed by elevated expressions of Fas and cleaved Caspase-3 protein. Furthermore, the proliferation of Jurkat cells was markedly reduced by RIG-I overexpression (1.13 ± 0.44 vs 0.91± 0.45; p=0.000) or pppRNA 12 and 24 hours activation (1.43 ± 0.89 vs 1.17 ± 0.25; p=0.000, 1.17 ± 0.82 vs 1.05 ± 0.27; p=0.017). Whereas, neither the ratio of apoptotic population nor the cell viability of the RIG-I knockout clones exhibited significant changes following pppRNA activation.

Conclusion: The present study indicated that increased expression of RIG-I in peripheral T lymphocytes was significantly associated with lymphopenia in patients with DM. The RIG-I pathway played a critical role in the induction of lymphocyte apoptosis.

### P5 Clinical symptoms indicative of a paraneoplastic etiology in adult dermatomyositis patients: A retrospective case-control study

#### Géza Nagy, Sándor Husz, Lajos Kemény, Lászlo Kovács, Zsuzsanna Bata-Csörgő

##### University of Szeged; Szeged, Hungary

###### **Correspondence:** Géza Nagy

Objective: While some clinical features are reported as risk factors for cancer-associated dermatomyositis (DM), most results are conflicting with paucity in the literature regarding paraneoplastic DM. The aim of our study was to determine clinical symptoms, which are characteristic for a paraneoplastic etiology by comparing our findings with those in the literature.

Methods: A retrospective analysis of adult patients who were diagnosed with dermatomyositis between January 1998 and January 2014 was performed. Demographic data, the presence or absence of objective and subjective symptoms, initial and follow-up treatments, and the times of relapses were examined.

Results: In all, 30 idiopathic and 11 paraneoplastic DM cases were reviewed and compared. Of the cutaneous symptoms, heliotrope rash with a profound periorbital oedema (43.3% vs 81.8% %; p=0.038), pruritus (16.7% vs 63.6%; p=0.007) and skin ulceration (0.0% vs 27.3%; p=0.015) were found to be associated with a paraneoplastic etiology. In addition, respiratory muscle involvement (3.3% vs 36.4%; p=0.014), lower creatine kinase increase (3355.9 ± 53116 vs 1009 ± 813.3; p=0.037), lack of ANAs or ENAs (40.0% vs 100%; p=0.002) and absence of immune complex deposits in the intramuscular vasculature of the skeletal muscles (16.7% vs 72.7%; p=0.001) also showed a positive association with paraneoplasia.

Conclusion: The clinical manifestations of paraneoplastic DM differ from previously described risk factors for cancer-associated DM. This indicates a need for etiological stratification when assessing malignancy-associable features in future studies. Additionally, presence of these symptoms should warrant more extensive tumor screenings following initial DM diagnosis.

### P6 Pronounced focal muscle pathology in a patient with Anti-NXP2 autoantibody associated severe myositis

#### Leonie Rager^1^, Marco Meyer^1^, Mesut Yenigün^1^, Corinna Preusse^2^, Heidrun Krämer-Best^1^, Stephanie Wolff^1^, Werner Stenzel^2^, Anne Schänzer^1^

##### ^1^Justus-Liebig-Universität Gießen, Gießen, Germany; ^2^Charité Universitätsmedizin Berlin, Berlin, Germany

###### **Correspondence:** Anne Schänzer

Introduction: Classification of idiopathic inflammatory myopathies (IIM´s) including dermatomyositis (DM), polymyositis (PM), immune-mediated-necrotizing-myopathy (IMNM) and sporadic inclusion body myositis (sIBM) are based on the clinical presentation and muscle pathology. Myositis autoantibodies (MSAs) are useful tools for identifying subgroups and predicting the clinical progress and outcome under therapy. Anti-NXP2 is a major MSA in juvenile DM and is associated with severe muscle pathology. In adults, anti-NXP2 is rather rare and thorough descriptions of muscle pathology findings are sparse.

Case report: We describe a 59 years-old male patient who developed a proximal myopathy with increased CK levels up to 40.000 U/l. MRI images of the proximal tighs showed muscle oedema and areas with high signal intensity suggestive for myositis and electromyography was compatible with a myopathy. The muscle biopsy showed a large regional oedema with pre-necrotic muscle fibres with decreased enzymatic staining. MHC1 was upregulated in these muscle fibres with deposits of complement (C5b9) without significant lymphocytic infiltrates. The pathology was similar as described for so called 'regional ischemic myopathy' (RIIM). RIIM is proposed as a separate entity by some authors but has not been included in the classification of IIMs. In the follow-up of this patient, highly elevated anti-NXP2 ABs and PM75 and RO52 antibodies were detected. Additional electron microscopy findings as endomysial capillaries with endothelial cells containing microtubular inclusions and immunohistochemistry with a sarocplasmatic expression of myxovirus resitance A (MxA) were consistent with the diagnosis of DM. Under immunosuppression the patient`s muscle strength slowly increased and he was able to walk without help. The patient was former smoker but tumour screening was negative. The level of anti-NXP2 ABs decreased and PM75 was not detectable whereas RO52 was still high.

Conclusion: We demonstrate a new morphological phenotype in a patient with severe myositis associated with anti-NXP2 ABs showing a strong focal pathology resembling a focal ischemic damage. Additional TEM findings and analysis of MxA expression supports the diagnosis of DM, hence placing this phenotype in the spectrum of Dermatomyositis pathology.

### P7 Short-term changes after treatments in needle electromyography among patients with polymyositis and dermatomyositis: A retrospective study

#### Hidenaga Kawasumi^1^, Yasuhiro Katsumata^2^, Eiichi Ito^3^, Yasushi Kawaguchi^2^

##### ^1^Tokyo Metropolitan Ohtsuka Hospital, Tokyo, Japan; ^2^Tokyo Women’s Medical University, Tokyo, Japan; ^3^Fukushima National Hospital, Fukushima, Japan

###### **Correspondence:** Hidenaga Kawasumi

Objectives: Although needle electromyography (EMG) is useful for diagnosis of polymyositis (PM) and dermatomyositis (DM), the efficiency of EMG on estimating the response to any therapies remains to be elucidated. We aimed to examine short-term changes after treatments in needle EMG findings among patients with PM/DM.

Methods: This is a retrospective and observational study. Patients were included when they met all these criteria: (1) fulfill the Bohan and Peter classification criteria; (2) were administered to our University hospital from 2009 through 2014; (3) received needle EMG both before and after treatments. The data of manual muscle testing (MMT), serum creatine kinase (CK) levels, and needle EMG findings: fibrillation potential, positive sharp wave, low amplitude motor unit potential (MUP), and short duration MUP were retrospectively collected.

Results: Ultimately, 23 patients were included in the present study, and 23, 16, and 10 patients received needle EMG at 0, 4, and 8 weeks after start of initial treatments, respectively: fibrillation potential, positive sharp wave, low amplitude MUP, and short duration MUP improved in 56%, 63%, 31%, and 25% of the patients at 4 weeks, and in 90%, 90%, 100%, and 90% of the patients at 8 weeks, respectively. Fibrillation potential and positive sharp wave significantly improved at 4 weeks, and all of 4 findings improved at 8 weeks (p < 0.05 in each comparison). The improvements of MMT and CK levels did not significantly agree with those of needle EMG findings except for fibrillation potential/positive sharp wave and CK levels at 8 weeks.

Conclusion: The present study showed that electrical activities in muscles significantly improved at as early as 4 and 8 weeks after start of initial treatments. Needle EMG findings can serve as an outcome measure independent upon MMT and CK levels in PM/DM.

### P8 Clinical characteristics of patients with clinically amyopathic dermatomyositis positive for anti-TIF1r antibody

#### Yukie Yamaguchi, Miwa Kanaoka, Asami Akita, Yasushi Ototake, Tomoya Watanabe, Nobuaki Ikeda, Michiko Aihara

##### Yokohama City University Graduate School of Medicine, Yokohama, Japan

###### **Correspondence:** Yukie Yamaguchi

Objectives: It has been recognized that myositis-specific autoantibodies are correlated with unique sets of clinical manifestations. For instance, higher prevalence of malignancy is associated with dermatomyositis (DM) patients positive for anti-TIF1r antibody (Ab). Anti-MDA5 Ab is highly found in patients with clinically amyopathic DM (CADM) with rapidly progressive interstitial lung disease. In this study, we analyzed clinical features of patients with anti-TIF1r Ab-positive DM, especially in cases of CADM.

Methods: We enrolled 32 DM patients with anti-TIF1γ Ab, who were treating in our department. CADM cases were extracted from anti-TIF1r Ab-positive DM and their clinical features, such as skin manifestations, prevalence of concomitant malignancy and interstitial lung disease, and the level of anti-TIF1r Ab, were evaluated.

Results: Within 32 cases of DM patients positive for anti-TIF1γ Ab, there were 9 CADM cases (28.1%). In these cases, Gottron's papule, Gottoron’s sign, and heliotrope rash tended to be highly observed compared to that in patients with non-CADM patients with this Ab. The prevalence of concomitant malignancy, which was defined by the diagnosis within 2 years before or after DM diagnosis, was 60.9% (14/23) in anti-TIF1r Ab-positive non-CADM patients, while that was 11.1% (1/9) in anti-TIF1r Ab-positive CAMD patients, which was significantly lower (P = 0.02). Meanwhile, interstitial lung disease was observed in 4 out of 9 patients with CADM (44.1%), which tended to be higher than that in anti-TIF1r Ab-positive non-CADM patients (3/23, 13%, P = 0.08). There was no rapidly progressive type in all cases. Finally, serum anti-TIF1r antibody level was significantly lower in CADM patients than that in non-CADM cases (71.6 ± 39.7, 106.8 ± 35.2, respectively, P = 0.03).

Conclusion: Anti-TIF1r Ab-positive CADM cases may have different clinical features from the normally recognized profile of this antibody. The population of this study is small and further study in a larger population will be required.

### P9 Anti-calreticulin antibody – A Novel Autoantibody in Patients with Idiopathic Inflammatory Myopathies and Its Association with Malignancy

#### He Chen, Yong-Peng Ge, Guo-Chun Wang, Han-Bao Yang, Qing-Lin Peng, Ya-Mei Zhang, Xin Lu

##### China Japan Friendship Hospital, Beijing, China

###### **Correspondence:** He Chen

Objective: To investigate the occurrence of anti-calreticulin antibodies (anti-CRT Abs) and evaluate its association with malignancy in patients with idiopathic inflammatory myopathies (IIM).

Methods: In total, 211 patients with IIM were enrolled in our study and patients complicated with malignancy were separated into a single category. The levels of anti-CRT Abs were measured in serum samples from 106 with dermatomyositis (DM), 21 with polymyositis (PM), 34 with immune-mediated necrotizing myositis (IMNM), 50 IIM with malignancy and 40 healthy controls (HC) by an in-house enzyme-linked immunosorbent assay (ELISA) using recombinant full-length CALR. The clinical and laboratorial data were collected and compared between anti-CRT Abs positive and negative patients. Then 8 IIM patients were followed longitudinally whose changes in concentrations of anti-CRT Ab were assessed and variations in disease activity were measured by myositis disease activity assessment visual analog scales (MYOACT) scores. Receiver operating characteristic (ROC) curve analysis was performed to determine the value of anti-CRT Abs in distinguishing IIM with malignancy from those without malignancy.

Results: Serum levels of anti-CRT Abs were significantly higher in IIM [median 5.3 AU (IQR 2.5-11.6)] than in HC [median 3.8 AU (IQR 1.8-6.4), P = 0.003] and anti-CRT Abs were judged to be positive in 48 of 211 IIM patients (22.7%). Among the IIM subgroups, 17/106 (16.1%) DM, 2/21 (9.5%) PM, 7/34(20.6%) IMNM and 22/50 (44%) IIM with malignancy were seropositive for anti-CRT Abs. Higher frequency of malignancy, heliotrope rash and gottron papules as well as elevated concentrations of immunoglobulin G (IgG) and anti-nuclear Abs (ANA) were present in IIM patients with positive anti-CRT Abs than in those negative. A positive correlation between serum anti-CRT Abs concentrations and MYOACT scores was shown by longitudinal study. ROC curve analysis revealed that the anti-CRT Abs had diagnostic value in distinguishing IIM with malignancy from those without malignancy, with an area under curve (AUC) value of 0.65 (95% CI 0.56 – 0.74, P = 0.001).

Conclusion: This is the first report to discover that anti-CRT Abs could be a novel autoantibody detected in IIM. The levels of anti-CRT Abs increased significantly and markedly high anti-CRT Abs levels could be a possible serological marker of malignancies in IIM patients.

### P10 Specific autoantibodies and clinical phenotypes correlate with the aberrant expression of immune-related microRNAs in Dermatomyositis

#### Lifang Ye, Xiaoming Shu, Guochun Wang

##### China-Japan Friendship Hospital, Beijing, China

###### **Correspondence:** Lifang Ye

Aims: The serum concentrations of miRNAs, miR-23a-3p, miR-23b-3p, miR-146a-5p, miR-146b-5p, and miR-150-5p were shown to be associated with immune and inflammatory progression. We assessed the expressions of these five miRNAs in association with clinical p henotypes and myositis-specific autoantibody-defined subgroups of Dermatomyositis (DM).

Methods: The present study included 49 patients with DM and 30 healthy controls. Serum conc entrations of miR-23a-3p, miR-23b-3p, miR-146a-5p, miR-146b-5p, and miR-150-5p were m easured by quantitative reverse transcription polymerase chain reaction (qRT-PCR). Associatio ns between serum concentrations of miRNAs and DM clinical immune-phenotypes were exa mined as well.

Results: Serum concentrations of miR-23b-3p, miR-146a-5p, and miR-150-5p were significantly down-regulated in DM patients (P < 0.001, P < 0.001, P = 0.002, respect ively), while miR-146b-5p was remarkably up-regulated in DM patients compared with those in healthy controls (P = 0.039). Similarly, the expressions of miR-23b-3p, miR-146a-5p, an d miR-150-5p were significantly down-regulated in peripheral blood mononuclear cells (PBM Cs) from DM patients. Further study indicated that serum level of miR-23b-3p was significa ntly correlated with creatine kinase (CK) (r = -0.286, P = 0.046), and serum level of miR -146a-5p was evidently correlated with C-reactive protein (CRP) (r = -0.358, P = 0.012). Significant correlations were also observed between serum level of miR-146b-5p and CRP (r = -0.347, P = 0.014) and erythrocyte sedimentation rate (ESR) (r = -0.287, P = 0.046). In addition, the expression level of miR-146b-5p was up-regulated in DM complicated with tumors compared with those without tumors (P = 0.001, P < 0.001, respectively). Espe cially, miR-150-5p was significantly down-regulated in DM patients with anti-MDA5 antib odies and anti-NXP2 antibodies compared with those without (P = 0.017, P = 0.047, respecti vely). No significant differences were observed between four sera microRNAs in patients with and without interstitial lung diseases (all P > 0.05).

Conclusion: The results suggest an association between four immune-related microRNAs and different clinical immune-phenotypes and this association may regulate the complexity of disease processes through multi-path ways in DM patients.

### P11 Increased serum Galectin-9 (Gal-9) in patients with idiopathic inflammatory myopathies

#### Lin Liang, Qinglin Peng, He Chen, Yamei Zhang, Xin Lu, Guochun Wang

##### China-Japan Friendship Hospital; Beijing, China

###### **Correspondence:** Lin Liang

Objective: To investigate the levels of serum galectin9(Gal-9) in patients with idiopathic inflammatory myopathies (IIMs) and their relation to IIM-related features.

Methods: Serum levels of Gal-9 were detected in 107 Dermatomyositis (DM)patients, 22 immune mediated necrotizing myositis(IMNM) patients ,22 anti-synthetic enzyme syndrome(ASS) patients and 30 healthy controls by using the ELISA method. Total RNA isolated from Peripheral blood mononuclear cells (PBMC)of 12 anti-MDA5 positive IIM patients ,7 IMNM patients and 6 healthy controls were used for analyzing the mRNA levels of Tim3, CD44, MIX1 and IFIH1 by quantitative reverse transcription polymerase chain reaction (qRT-PCR).

Results: Serum levels of Gal-9 were significantly increased in IIM patients [median 19.8, interquartile range (IQR) 10.0-33.6] compared to those in healthy controls (median 4.9 ng/ml, IQR3.5-6.3, p< 0.001). The Gal-9 levels in cultured supernatant of PBMC from anti-MDA5 DM patients(mean 3.7 ±2.3ng/ml) was higher than those from healthy controls(mean 1.4±1.2ng/ml, p=0.049). A significantly high level of serum Gal-9 was found in patients with positive MDA5 antibodies, rapidly progressive interstitial lung disease(RPILD), dysphagia and arthritis. According to cross-sectional study and follow-up study, there was a positive correlation between serum Gal-9 levels and disease activity. Interestingly, serum Gal-9 levels were found to be associated with the expression of type I interferon-inducible genes MX1 and IFIH1, which were observed to be elevated in the PBMC of IIM patients and it was consistent with the trend of serum Gal-9. In addition, CD44, a receptor of Gal9, was found increased in the PBMC of anti-MDA5 positive DM patients than IMNM patients(p< 0.001), while there was no significant difference in Tim3 mRNA expression between anti-MDA5 positive IIM patients and IMNM patients.

Conclusions: The serum Gal-9 levels were significantly increased and associated with disease activity in DM patients. The dysregulated Gal-9 and its receptor CD44 may play a role in DM pathogenesis.

### P12 Myogenesis impairment in Dermatomyositis

#### Laure Gallay, Nathalie Streichenberger, Guy Mouchiroud, Bénédicte Chazaud

##### Institut NeuroMyoGène, Universite Claude Bernard, Lyon, France

###### **Correspondence:** Laure Gallay

Objectives: Dermatomyositis (DM) is a rare acquired myopathy, occurring during child and adulthood, and potentially life-threatening. Diagnosis is based on the association of muscle weakness, inflammatory infiltrates on histological muscle sections and auto-antibodies in the serum. Etiopathogenesis, that has mainly focused on adaptative immunity, remains largely unknown. In DM, the necrosis/regeneration balance appears to be negative, with a chronic course and alleged clinical outcome. Nevertheless, the capacity of muscle regeneration has been barely studied. In normal muscle, lesion is followed by regenerative process, achieved by adult myogenesis of myogenic precursor cells (MPCs) that build new myofibers. The aim of our project is to evaluate DM-derived MPC capacity to undertake myogenesis.

Methods: DM (severe [n=5] and mild [n=3]), and healthy controls (HC [n=5]) selection (sex and age paired) was performed in the Hospices Civils de Lyon hospital database and samples were obtained from the hospital biobank. Myogenesis was explored in vitro by the culture of MPCs for the evaluation of their proliferation, differentiation as well as fusion, and were analyzed by immunofluorescence. Proliferation was evaluated as the incorporation of a thymidine analog into DNA during proliferation. Terminal myogenic differentiation was evaluated by myogenin expression, an essential transcription factor of myogenic differentiation. Cell fusion was quantified as the number of nuclei incorporated into myotubes. Quantitative analysis was performed using Image J software, and statistical analysis was performed by mean comparison (t test or ANOVA).

Results: Analysis of MPC proliferation indicates that DM-derived MPCs exhibited a significanty lower proliferation than HC-derived MPCs, and this alteration was more pronounced in severe DM than in mild (p=0.02 and p=0.001, respectively). Terminal myogenic differentiation was also strongly impaired in severe DM-derived MPCs while less altered in mild-DM-derived cells (p=0.002 and p=0.004, respectively). MPC fusion was significantly altered in DM-derived MPCs with a dramatic decreased observed in severe DM and to a lesser degree in mild DM (p= 0.0005 and p=0.003, respectively).

Conclusion: These results show that skeletal muscle regeneration is impaired in DM due to altered capacities of MPCs to proliferate, to differentiate and to fuse. This may explain in part the muscle alterations and muscle weakness that are observed in patients. Further investigations will include the seek for molecular mechanisms underlying myogenesis alteration in DM. These results support the hypothesis of intrinsic alterations of MPC properties in DM, participating to the pathogenesis of the disease.

### P13 The IgG2 isotype of anti-transcription intermediary factor 1-gamma autoantibodies is a biomarker of mortality and cancer in adult dermatomyositis

#### Audrey Aussy^1^, Manuel Fréret^1^, Laure Gallay^2^, Laurent Drouot^1^, Fabienne Jouen^1^, Yves Allenbach^3^, Olivier Benveniste^4^, Nadège Cordel^5^, Olivier Boyer^1^

##### ^1^Rouen University Hospital, Rouen, France; ^2^Edouard Herriot University Hospital, Lyon, France; ^3^Centre de Recherche en Myologie, Paris, France; ^4^Pitié-Salpêtrière University Hospital, Paris, France; ^5^French West Indies University, Pointe-à-Pitre, Guadeloupe

###### **Correspondence:** Audrey Aussy

Objective: Anti-TIF1gamma antibodies (aAb) are the main predictors of cancer in dermatomyositis (DM). Yet, a substantial proportion of anti-TIF1gamma positive DM patients remain without cancer. Our objective was to identify biomarkers to better evaluate the risk of cancer and mortality.

Methods: Anti-TIF1gamma aAb levels and IgG subclasses were identified using a home-developed quantitative immunoassay. Adult anti-TIF1gamma+ DM patients detected with this assay were enrolled between August 2013 and August 2017 in numerous French university hospitals . Age, gender, DM signs and activity, malignancy, and creatine kinase (CK) level were recorded. anti-TIF1gamma level and IgG subclasses were also recorded. Risk factors were determined by univariate and multivariate analysis according to a Cox regression model.

Results: Among the 51 adult patients enrolled (mean age 61+/- 17 years, sex-ratio m/f: 0.65), 40 (78 %) had cancer and 21 (41%) died, with 10+/-6 months mean survival. Detection of anti-TIF1gamma-IgG2 was significantly associated with mortality (p=0.0011) and occurrence of cancer during follow-up (p < 0.0001), with a 100% positive predictive value of cancer when mean fluorescence intensity of anti-TIF1gamma IgG2 was > 385. No cancer developed after 24 months of follow-up. Univariate survival analyses also showed that mortality was associated with age > 60 years (p=0.0003), active DM (p=0.0042), cancer (p=0.0031), male gender (p=0.011), and CK level > 1084 U/L (p=0.005). Multivariate analysis confirmed that age > 60 years (p=0.015) and presence of anti-TIF1gamma IgG2 (p=0.048) were independently associated with mortality. Concerning the evolution of the disease, 8/11 (73%) patients without cancer had persistence of DM signs. Among patients with cancer, 18/19 (95%) living patients had cancer in remission, among whom 13/18 (72%) had concomitanly complete remission of DM. Within the deceased group, 13/15 (87%) patients had active cancer and active DM at last follow-up.

Conclusion: This study has evidenced anti-TIF1gamma IgG2 as a potential new biomarker of cancer which should be helpful to identify the risk of mortality among anti-TIF1gamma+ DM patients with cancer. The absence of cancer occurrence after 24 months could lead to alleviate the screening examinations. The severity of this form of DM is here highlighted and suggest the importance of an early and strong treatment.

### P14 Association of IL-17RC rs708567 polymorphism with dermatomyositis: a pilot study

#### Joana Pozharashka, Lyubomir Dourmishev, Zornitsa Kamenarska, Maria Hristova, Gyulnas Dzhebir, Svetla Nikolova, Anton Vinkov, Radka Kaneva, Lyubka Miteva

##### Medical University Sofia, Sofia, Bulgaria

###### **Correspondence:** Joana Pozharashka

Objectives: Polymorphisms in the cytokine genes and their receptors are thought to influence the predisposition to dermatomyositis (DM). Evidence suggests that both the dysfunction of IL-17RC and the IL-17/IL-17R signalling axis could be implicated in the development of DM. The aim of this case-control study is to investigate the association between the IL-17RC rs708567 polymorphism and the susceptibility to DM in Bulgarian patients.

Methods: Altogether 33 patients with DM as well as 93 unrelated healthy controls were included in this study. The analysis of IL-17RC rs708567 was performed by using TaqMan genotyping assay. Allele and genotype frequencies were compared between DM cases and controls, using Fisher’s exact test to calculate p values for 2×2 tables. Where significant, data were expressed as p-value, odds ratios (OR) with exact 95% confidence intervals (CI). Test for Hardy-Weinberg equilibrium was done by χ2 statistics.

Results: The distribution of the genotypes was in Hardy-Weinberg equilibrium (HWE). The IL-17RC rs708567 CC+CT types were found associated with the development of DM (p=0.035, OR 2.8, 1-8). No association was found concerning the allele distribution, although the C allele was prevalent among the patients compared to controls, leading to increased OR (53.0% vs. 44.1%, p=0.13, OR 1.4, 95% CI 0.8-2.5).

Conclusion: Our results indicate that the IL-17RC rs708567 polymorphism might play a role in the development of DM.

### P15 Dermatomyositis associated with antibodies to small ubiquitin like modifier activating enzyme: a retrospective series of 41 cases

#### Mathieu Vautier^1^, Pierre Duffau^2^, Olivier Chosidow^3^, Alice Berezne^4^, Nadege Cordel^5^, Jean Scmidt^6^, Marie Jachiet^7^, Julie Graveleau^8^, Nicolas Champtiaux^1^, Perrine Guillaume-Jugnot^1^, Baptiste Hervier^1^, Judith Victor^2^, Aude Rigolet^1^, Oceane Landon-Cardinal^1^, Olivier Benveniste^1^, Yves Allenbach^1^

##### ^1^Pitié-Salpêtrière University Hospital, Paris, France; ^2^Bordeaux University Hospital, Bordeaux, France, ^3^Hôpital Henri Mondor, Créteil, France; ^4^CH Annecy Genevois, Metz-Tessy, France; ^5^Guadeloupe University Hospital, Les Abymes, Guadeloupe; ^6^Amiens University Hospital, Salouël, France; ^7^Hôpital Saint-Louis, Paris, France; ^8^St Nazaire Hospital, Saint-Nazaire, France

###### **Correspondence:** Mathieu Vautier; Yves Allenbach

Myositis-specific antibodies detection is a crucial diagnostic tool since they are specific and delimit homogenous groups of patients. Anti-SAE antibody positive (anti-SAE+) DM represent less than 10% of DM, and the number of reported cases remains limited. Muscular and extra-muscular feature remains to be characterized in anti-SAE+ DM. This multicentric national study (n = 37 centers) included DM patients according to the ENMC criteria, with anti-SAE antibodies detected with immuno-DOT test, according to local practice. Demographic, clinical, paraclinical data and treatment were analyzed retrospectively. Patients (n = 41) were predominantly women (85.4%) with a median age at diagnosis of 53 years [5 - 90]. All patients had a classical DM skin rash in photo-exposed areas (erythema of the neckline (56%), shawl sign (56%), and hands, peri-inguinal erythema (77%), a sign of gottron (79%)). Nine patients (23%) presented cutaneous ulcerations and 4 patients (10%) had subcutaneous calcifications. Muscular involvement was observed in 82% of patients. Two-thirds (62.5%) of the patients had proximal muscle weakness, but the muscular deficit was mild (median Medical research Council (MRC) Score 4 [2 - 5]). Creatine kinase (CK) level was moderately elevated (median = 216 IU / l [63 - 10511]) and 62.5% had an increased level. In contrast, almost half of the patients (40%) had swallowing disorders. Six out of 41 (14.6%) patients had parenchymal computed tomography CT lesions: organized pneumonia (OP) (n=3/6) and/or NSIP (n = 3/6). The respiratory functional explorations were normal in half of the cases (n = 3/6). Five patients (12%), with a median age of 68 years [39 – 81], had synchronous neoplastic pathology (rectal cancer, gynecologic cancer, and pulmonary cancer). The majority of patients received first-line treatment with systemic corticosteroids (84%) and 73% received concomitant immunosuppressive therapy (methotrexate in n =17/37). Thirty-eight percent of patients received intravenous immunoglobulin, in most cases, due to swallowing disorders (78.6%). The median duration of follow-up was 21 months [1 - 302]. Majority (55%), of patients received only one line of treatment. The others received several lines of treatment because of relapse or persistence of cutaneous and/or muscular symptoms (2 lines, n = 6, 3 lines, n = 7 and 4 lines, n = 3). Only one patient died during follow-up, from complications of disseminated tuberculosis. DM SAE is a rare subgroup of DM characterized by a classical DM skin rash with a mild muscle weakness but frequently swallowing disorders. Non-serious pulmonary

### P16 Dermatomyositis with anti-MDA5 auto-antibodies: is there a seasonal pattern of disease onset?

#### Kubéraka Mariampillai^1^, Ségolène Toquet^1^, Yurdagul Uzunhan^2^, Gaelle Leroux^1^, Laure Gallay^3^, Alain Meyer^4^, Hilario Nunes^2^, Benjamin Granger^1^, Olivier Benveniste^1^, Yves Allenbach^1^

##### ^1^Pitié Salpêtrière Hospital, Paris, France; ^2^Avicenne Hospital, Paris, France; ^3^Lyon Sud University, Lyon, France; ^4^Strasbourg University, Strasbourg, France

###### **Correspondence:** Kubéraka Mariampillai

Objectives: Melanoma differentiation-associated gene 5 (MDA5) is a protein involves in anti-viral defense. Anti-MDA5 autoantibodies are associated with dermatomyositis (DM) characterized by a very high frequency of interstitial lung disease (ILD) with life-threatening complications. We hypothesize that viral infections may trigger the disease, thus we test if there is evidence for seasonal variation in MDA5 DM.

Methods: An observational, retrospective multicentric (n=37) cohort study was performed. Anti-MDA5 antibody positive (Euroimmun® or D-Tek®) patients were included. Myositis onset periodicity and associated predictive factors were assessed by goodness of fit chi squared test and non-linear model.

Results: Anti-MDA5+ patients (n=122) were 49 years [34-58] at diagnosis. Skin lesions were highly frequent (87.5%) as well as ILD (76.47%) and the mortality was high (27.27%). The first symptoms appeared in spring for 36% (n=37; p=0.05). There was no evidence for seasonality regarding the first respiratory symptoms (p=0.16) and deaths (p=0.34). Next we compared MDA5 DM patients with a spring onset to the others. ILD occurred in 70.3% in patients with a spring onset and in 87.9% for the others (p=0.05). No significant differences were highlighted regarding the gender (p=0.4), age at diagnosis (p= 0.4), ethnicity (p=0.3), skin lesions (p=0.6), proximal deficit (p=0.2), or rheumatologic signs (p=0.3). In addition the frequency in admission in intensive care unit was similar 37% for the spring onset patients and 40% for the others (p=0.9) as well the mortality rate 27% in spring onset patients and 34% in others (p=0.5).

Conclusion: These exploratory results may suggest a seasonal distribution in the onset of the disease without showing a clear different phenotype for patients with a spring onset. Harmonic analyses are ongoing to explore and identify factors associated with seasonality.

### P17 Polymyositis (PM) and Dermatomyositis (DM) Symptom Flares and Associated Impact from the Patient Perspective

#### Lisa Christopher-Stine^1^, Winn Nelson^2^, William Kelly^1^, Linda Kobert^3^, Barima Opong-Owusu^2^, Michael Reed^4^

##### ^1^Johns Hopkins University, Baltimore, USA; ^2^Mallinckrodt Pharmaceuticals, California, USA, ^3^The Myositis Association, Alexandria, USA; ^4^Vedanta Research, Chapel Hill, USA

###### **Correspondence:** Lisa Christopher-Stine

Objectives: Flare activity or worsening symptoms are not well defined for myositis. This analysis characterizes PM and DM flares from the patient perspective and reports the corresponding disability and rate of unplanned medical encounters.

Methods: Online surveys were collected from volunteer patients from The Myositis Association and Johns Hopkins Myositis Center. Data collection included sociodemographics, flare symptoms, Health Assessment Questionnaire Disability Index (HAQ-DI) and HAQ-Pain index, Work Productivity and Activity Impairment (WPAI), emergency department and urgent care (ER/UC) visits during the past year. Flare frequency was assessed asking, “Have you ever had a flare or worsening myositis symptoms?” if yes, “How many times in the past 12 months?”

Results: 564 individuals with self-reported diagnoses of PM (n=243) and DM (n=321) completed the survey between December 2017 and May 2018. Recall of symptom flares was reported by 524 (86.6% Caucasian, 78.1% female, 43.1% employed, mean age 55.4 years). Among the 524 respondents, 33 (6.3%) reported never experiencing a flare, 113 (21.6%) reported 0 flares in the past year, 244 (46.6%) reported 1-3 flares, and 134 (25.6%) reported ³ 4 flares in the past year. The pattern of flare frequency was similar for PM and DM respondents. Among the 378 respondents with self-reported flares in the past year, the most common worsened symptoms were muscle weakness (84%), extreme fatigue (79%), muscle pain/discomfort (64%), trouble climbing stairs (62%), trouble standing from a seated position (50%), and skin rash (45%). Dysphagia occurred for 27%. Increasing flare frequency was associated with greater mean HAQ-DI (F=17.653, P < .001), HAQ-Pain (F=28.291, P < .001), WPAI activity impairment (F=20.109, P < .001) and, for the employed, lost productive work time (F=9.933, P < .001). Rates for one or more past year ER/UC visits were also related (χ2=11.634, P < .009) to flare frequency (never 9.1%, 0 flares 8.8%, 1-3 flares 20.5%, ³ 4 flares 23.1%).

Conclusion: PM/DM-related flares or worsening symptoms are common, with 72% of respondents reporting one or more flares in the past year. Exacerbations of muscle weakness and fatigue were the most common flare symptoms, and flare frequency was associated with greater disability, more pain, more absenteeism and presenteeism, and activity impairment. ER/UC utilization was also more common as flare frequency increased. Higher frequency of patient-reported flares may serve as a marker of worsening physical functioning and intensifying health care needs, and therefore suggests their importance in the clinical assessment of patients with PM/DM.

### P18 murin model of anti-TIF1gamma dermatomyositis: preliminary results

#### Audrey Aussy^1^, Maire Chilles^1^, Laurent Drouot^1^, Fabienne Jouen^1^, Christophe Arnoult^2^, Olivier Benveniste^3^, Olivier Boyer^1^

##### ^1^Rouen University Hospital, Rouen, France; ^2^Normandie Université, Rouen, France; ^3^Pitié Salpêtrière Hospital, Paris, France

###### **Correspondence:** Audrey Aussy

Introduction: Pathophysiology of dermatomyositis involve type 1 interferon pathway, complement system, vasculopathy and antibodies but remain elusive. The absence of murin model is limiting the capacity to establish the exact role of these different actors. Among the specific autoantibodies (aAb) associated with DM, anti-TIF1gamma aAb are associated with poor prognosis in juvenile and adult DM, with an higher risk of cancer in adults. The objective is to evaluate the role of anti-TIF1gamma aAb in murin model.

Methods: The first approach consisted of daily injection of 400μg of purified IgG from patient with anti-TIF1gamma+ DM or from healthy donors. The second approach consisted of immunization of mice with human recombinant TIF1gamma protein diluted in adjuvant containing ODN-CpG. The level of anti-TIF1gamma antibodies were evaluated with home-made assay in ALBIA (Adressable Laser Beads Immunoassay). Muscular strength was analyzed using grip-test. Muscles were analyzed after staining with Eosin-hematoxylin, indirect immunofluorescence or immunohistochemistry.

Results: Passive transfer of purified IgG containing anti-TIF1gamma antibodies provokes moderate muscle weakness but no histological abnormalities. Immunization with recombinant TIF1gamma protein leads to muscle weakness and moderate histological lesions such as edema, necrosis and IgG deposits.

Conclusion and perspectives: IgG containing anti-TIF1gamma antibodies from patients are not sufficiant to induce DM lesions. The anti-TIF1gamma immune response lead to muscular weakness and moderate inflammatory lesion in muscle, suggesting the associated role of antigen-presnetating cells, T and B cells in the occurrence of disease. These results encouraged us to reproduce the experiments using other adjuvants which activate the type 1 interferon pathways.

### P19 Novel Nailfold Video Capillaroscopy Parameters for Monitoring Juvenile and Adult Dermatomyositis

#### Nathan Arnett, Hans Prakash, Alexander Gorbach, Frederick Miller, Lisa Rider, Adma Schiffenbauer

##### National Institutes of Health, Bethesda, USA

###### **Correspondence:** Nathan Arnett; Adma Schiffenbauer

Objectives: Nailfold capillaroscopy has previously been used to assess nailfold capillary density in patients with dermatomyositis (DM). Additional attributes of nailfold capillary architecture, such as tortuosity and interlimb distance, as well as nailfold capillary blood velocity, have not yet been fully described in a quantifiable manner. In this study, quantification methods were developed to evaluate three attributes of nailfold capillary architecture (capillary density, interlimb distance, and tortuosity) and two attributes of nailfold capillary blood velocity [arterial blood velocity (BVA) and venous blood velocity (BVV)]. These attributes were then evaluated in DM patients and healthy control subjects.

Methods: Eighteen patients with DM, twenty-eight patients with Juvenile Dermatomyositis (JDM), and fifteen healthy control subjects were enrolled in a myositis natural history study from July 2013 to March 2017. These subjects underwent Nailfold Video Capillaroscopy (NFVC) and Nailfold Capillaroscopy (NFC) evaluation. During the study, participants sat in a comfortable upright position, and NFC photography was obtained and NFVC was conducted on the left ring finger of each patient. Imaging was performed using a Leica M205C stereomicroscope and six to ten images were taken with the recording of videos up to fifteen seconds long. Measurements of the five capillary parameters were performed on five to ten capillaries and averaged. The Spearman’s rank correlation coefficient was used to identify significant correlations among the five capillaroscopy parameters.

Results: Significant differences were found between JDM/DM patients and healthy control subjects for capillary density (medians of 5.0 capillaries/mm vs. 6.3 capillaries/mm, p < 0.001), interlimb distance (medians of 13.7 um vs. 11.0 um, p < 0.001), tortuosity (medians of 5.0 radians vs. 4.0 radians, p < 0.05), arterial blood velocity (medians of 552.5 um/s vs. 1068.0 um/s, p < 0.05), and venous blood velocity (medians of 360.2 um/s vs. 720.4 um/s, p < 0.05). Significant correlations were found between capillary density and interlimb distance in DM patients (ρ = -0.51, p < 0.001), and between BVA and BVV in DM patients (ρ = 0.96, p < 0.001) and controls (ρ = 0.85, p < 0.001).

Conclusions: This study presents new analytical techniques for NFVC that offer significant promise as tools in diagnosing patients with JDM/DM due to their quantifiable objective nature and the significant differences detected between patients and controls.

### P20 Distinct clinical profiles of Chinese and Swedish idiopathic inflammatory myopathy patients with anti-melanoma differentiation associated gene 5 (MDA5) antibody

#### Ho So^1^, Ingrid Lundberg^2^, Maryam Dastmalchi^2^, Valérie Leclair^2^, Tommy Lam, Roy Ho, Victor Wong

##### ^1^Kwong Wah Hospital, Hong Kong, Hong Kong; ^2^Karolinska Institutet, Solna, Sweden

###### **Correspondence:** Ho So

Objective: The objectives of the study was to compare the prevalence of the anti-melanoma differentiation-associated gene 5 antibody (anti-MDA5 Ab) and various clinical features between a Chinese and a Swedish IIM cohort .

Methods: This multicenter retrospective cohort study was conducted on IIM patients with available anti-MDA5 Ab results seen in the rheumatology clinic or admitted to the rheumatology wards of the participating hospitals in Hong Kong and Karolinska University Hospital, Sweden from September 2015 until August 2018. The diagnosis of IIM was based on the 2017 European League Against Rheumatism/American College of Rheumatology Classification Criteria with definite or probable cases being included. Patients with juvenile onset myositis were excluded. Demographics and clinical features were collected by reviewing medical records. A commercial line blot immunoassay kit (EUROIMMUN) was used to detect the anti-MDA5 autoantibodies.

Results: Altogether, 402 patients with IIM (Sweden: 206, Hong Kong: 196) were recruited. The Swedish patients were older (mean age 58.1 vs 51.3 years, p < 0.001), and the frequency of male was higher (41.4% vs 23.5%, p < 0.001). Subgroups of patients were: dermatomyositis 39.5%, polymyositis 39%, inclusion body myositis 15% and clinically amyopathic dermatomyositis 2% in the Swedish cohort, and 39.8%, 38.8%, 0% and 21.4% respectively in the Chinese cohort. Anti-MDA5 Ab was found in 11.7% of the Chinese and 5.83% of the Swedish patients (p=0.036). The Hong Kong Chinese patients were found to have significantly more ILD (53.6% vs 35.4%) and RPILD (9.23% v 3.95%), while the Swedish patients had more dysphagia (50% vs 28.7%) and cardiac involvement (13.5% vs 5.5%). Anti-MDA5 Ab was associated with male gender, younger age at diagnosis, amyopathy, ILD and RPILD in the Swedish patients. The same associations were found in the Chinese patients except the young age at diagnosis. Besides, in the Chinese cohort, the antibody was also positively associated with skin ulceration and vasculitis, and negatively associated with cancer and dysphagia. When grouping all the anti-MDA5 Ab positive patients together, Chinese patients had significantly more vasculitic skin changes (68% vs 0%, p < 0.001) than the Swedish. They also had a tendency towards developing more skin ulcers (47.8% vs 9.09%, p=0.053). No other significant difference was identified.

Conclusion: The distinct clinical profiles identified in both groups could enhance our knowledge about the clinical utility of the antibody and the deciphering of the pathogenesis of this complex disease.

### P21 The clinical significance of progranulin in DM patients

#### Shan-Shan Li, Yamei Zhang, Xin Lu, Guochun Wang

##### China-Japan Friendship Hospital, Beijing, China

###### **Correspondence:** Shan-Shan Li

Objective: To investigate the clinical significance of progranulin (PGRN) in patients with dermatomyositis (DM).

Methods: The levels of PGRN and IL-6 in 15 patients with DM (untreated before and 3 months after treatment) and 8 healthy controls were detected by ELISA in serum. The relationship between PGRN and clinical data was analyzed. T-test and Spearman correlation analysis were used.

Results: The level of PGRN in untreated DM patients (109.25 + 74.01 ng/ml) was much higher than those in healthy controls (27.04 + 15.07 ng/ml) (t = 3.076, P = 0.006). There was no difference in PGRN between patients with and without ILD. The expression of PGRN in patients, whose muscle strength was less than or equal to grade 3, was higher than those with muscle strength over grade 3 (150.16+75.17 ng/ml vs 62.51+37.52 ng/ml, t=2.787, P = 0.015). In laboratory data, PGRN was correlated with the levels of AST (r = 0.806, P = 0.000), LDH (r = 0.595, P = 0.019) and HBDH (r = 0.668, P = 0.007), but not with ALT or CK (p > 0.05). There was no significant difference in PGRN levels between MSA positive group and negative one (t = 0.808, P = 0.434). PGRN in serum was correlated with the expression of IL-6 (r = 0.824, P = 0.000). After 3 months of treatment, PGRN (62.41 +48.49 ng/ml) decreased significantly (t=3.956, p=0.001) in these patients, and there was no difference compared with healthy controls (t=1.993, p=0.059).

Conclusions: PGRN levels of DM patients increased significantly in disease onset, and decreased after effective treatment, which may be related to the degree of muscle injury and inflammation.

### P22 Performance of the 2017 European League Against Rheumatism / American College of Rheumatology (EULAR/ACR) Classification Criteria for adult idiopathic inflammatory myopathies in a Hong Kong cohort

#### Ho So, Roy Ho, Tommy Lam, Victor Wong

##### Kwong Wah Hospital, Hong Kong, Hong Kong

###### **Correspondence:** Ho So

Objectives: The objective of the study was to evaluate the performance of the 2017 EULAR/ACR classification criteria in a cohort of Hong Kong adult idiopathic inflammatory myopathy (IIM) patients. The secondary objectives included examining the level of agreement between the new criteria and the traditional criteria and assessing the effect of including other myositis specific autoantibodies (MSAs) into the criteria.

Methods: This was a multi-centre retrospective cross-sectional study. Consecutive patients with a clinical diagnosis of IIMs and MSA tested seen in the rheumatology clinic or admitted to the rheumatology wards of the participating hospitals in Hong Kong up till April 2018 were recruited. Patients with juvenile onset myositis were excluded. Clinical parameters required by the two criteria will be collected by reviewing the medical records. A commercial line blot immunoassay kit (EUROIMMUN) was used to detect the MSAs.

Results: Two hundred and four patients with IIM were recruited. The mean age was 59.3 years. There was a female predominance of 76.5%. The subgroups of the patients were: polymyositis 40.7%, dermatomyositis 38.2%, clinically amyopathic dermatomyositis patients 21.1%. MSAs were detected in 59.3% of the patients with anti-Jo-1 antibody being the commonest (13.2%). The new 2017 EULAR/ACR Criteria could classify 96.1% of the patients as having definite or probable IIM. The Bohan and Peter criteria could only classify 76.0% of the patients. When combining with the Sontheimer’s criteria for CADM, 93.1% of the patients could be classified. The percentage agreement of the new and the Bohan and Peter criteria also increased from 77.0% to 93.1% when the latter was supplemented by the Sotheimer’s criteria. If the presence of any MSAs is considered one of the criteria, the performance of the 2017 criteria improved to 97.5% while the combined Bohan and Peter/Sontheimer’s criteria to 96.1%. However, the new criteria still failed to highlight the important subtype of IIM associated with anti-MDA5 autoantibody.

Conclusion: In a population with a significant proportion of CADM patients, the new 2017 EULAR/ACR classification criteria outperformed the old criteria. Finally, a clinico-serological criteria for “anti-MDA5 syndrome” is proposed in which a patient must have positive serologic testing for an anti-MDA5 autoantibody, plus one of the following conditions: myositis by the new EULAR/ACR criteria, interstitial lung disease or typical rash (skin ulceration, palmer papules).

### P23 Anti-NXP2 Dermatomyositis: a severe muscle and skin disease

#### Camille Rasmussen, Laure Gallay, Alain Meyer, Delphine Larivière, Sarah Leonard-Louis, Perrine Guillaume-Jugnot, Mathieu Vauthier, Kuberaka Mariampillai, Olivier Benveniste, Yves Allenbach

##### Hôpital Pitié Salpêtrière; Paris, France

###### **Correspondence:** Camille Rasmussen

Objectives: Myositis specific antibodies (MSA) delineates homogeneous groups of myositis patients with characteristic muscular and extra-muscular phenotypes. Anti-nuclear matrix protein 2 (NXP2) occurs only in juvenile and adult dermatomyositis (DM) patients (1-30%). Anti-NXP2 DM have been associated with (i) calcinosis and edema, (ii) a severe muscle vasculopathy at least for juvenile DM and (iii) an increased risk of malignancy in adults. The number of reported cases is limited and studies sometimes showed conflicting results (e.g on the risk of malignancy). We aimed to characterize the muscular and extra-muscular phenotype in an independent cohort.

Methods: This multicentric (n=9) retrospective study included adults and juvenile DM based on Bohan and Peter’s and/or the Sontheimer’s criteria, with anti-NXP2 antibody (Immunodot DTEK® or Euroimmun®). Clinical and paraclinical data were collected retrospectively using medical charts and an electronic database. Muscle biopsies were analyzed by two independent experts.

Results: Thirty nine patients were included (n=8 juvenile, n=31 adults DM). They were 29 years [IQR;19-49] (jDM 6.5 y [IQR;3-14.7] and aDM 38 y [IQR;22-52]) and mainly female (69 %). Nearly all patients presented myalgia (84.2%) and a proximal muscle weakness (89.7%), rather severe (57% had a MRC score ≤ 3); and half of them also showed a distal weakness (54.5%). The CK was 1983 U/L [IQR;744.3-6900]. The majority of patients (92.1%) presented a classical DM skin rash (heliotropic rash, gottron papules, manicure sign). Otherwise, edema was frequently observed (67.5%) and nearly a quarter (23%) had severe cutaneous manifestation, defined by the presence of either superficial ulceration or necrosis). Calcinosis was also reported in a third of the cases. 60% had dysphagia, and among them a quarter (25.9%) needed enteric nutrition. A quarter of patients presented a gastro-intestinal involvement (colitis, n=7). Cardiac involvement was reported in 5.4% and interstitial lung disease was identified in 14.8% of patients (with a chest CT). Cancer associated myositis was observed in 7.8% of people (n=3/38). Furthermore, comparison between adult and juvenile DM, showed that dysphagia and skin ulceration occurred more frequently in adult DM. All but one patient received corticosteroids and 92% were also treated with an immuno-suppressant. Patients received 1.5 line of treatment [IQR;1-2.250]. Median number of relapses per patient was 0 [range 0-4]. Follow-up was 21 months [IQR;7-35].

Conclusion: Anti-NXP2 DM is associated with a severe vasculopathy attested by important skin lesions and frequent GI involvement as well as a severe muscle disease (major weakness and dysphagia).

### P24 NVC (nailfold videocapilaroscopy) in MDA5 positive patients with and without skin ulcers

#### Florentina Berianu

##### Mayo Clinic, Ponte Vedra Beach, USA

Background/Purpose: The MDA5 was classified as a myositis-specific antibody and has been associated with rapidly progressive interstitial lung disease (ILD), amyopathic dermatomyositis (aDM), mechanic’s hands, ulcerations, inflammatory arthritis, and increased mortality. Nailfold videocapilaroscopy (NVC) represents the best method to assess the microvascular abnormalities. A defined pattern on NVC has been reported in patients with dermatomyositis. This study tought to distinguish the NVC characteristics of MDA5-positive with skin ulcers versus non skin ulcers to see if NVC findings will correlate with the severity of skin lesion.

Methods: The NVC was performed in all 7 MDA5 positive patients; mean age 59.6, 3 male, 4 female. The capillary parameters were scored as reported in literature. 3 patients MDA5 positive with skin ulcers were compared to 4 patients MDA5 positive without skin ulcers. Baseline clinical characteristics and serology were recorded in all patients.

Results: A scleroderma like pattern was noted in 5 out of 7 patients but no significant differences were noted between the 2 groups. In our sample only 2 patients MDA5 positive presented with Raynaud’s. 5 out of 7 patients MDA5 positive had periungual erythema, 2 had mechanics hands. 1 patient with cutaneous ulceration died soon after diagnosis and his NVC was normal.

Conclusion: No statistical significant differences were reported between the 2 groups. The comparison was done at different stages of the disease and response to therapy. Further studies on larger cohort of patients may find differences between the 2 groups.

### P25 Infections are leading cause of in-hospital mortality in Indian patients with inflammatory myositis

#### Hafis Muhammed, Latika Gupta, Abhishek Arvind Zanwar, Durga Misra, Able Lawrence, Vikas Agarwal, Amita Aggarwal, Ramnath Misra

##### Sanjay Gandhi Post Graduate Institute of Medical Science, Uttar Pradesh, India

###### **Correspondence:** Hafis Muhammed

Background: Idiopathic inflammatory myositis (IIM) are debilitating and often lead to mortality. We explored causes of in-hospital mortality and predictors of early mortality at a tertiary care center in northern India.

Methods: Records of adults and children with dermatomyositis (DM), polymyositis (PM), or anti-synthetase syndrome (ASS) who died between 2000 and 2018 were reviewed and cause of death determined. Early mortality was defined as death with 6 months of diagnosis. For comparison patients with IIM surviving more than 6 months after diagnosis and having registration number before and after the index case were included. Severe weakness was defined as MRC less than 3 in any group of muscles.

Results: Of the 38 (32 women) deaths, 20 were DM including 2 clinically amyopathic DM, 4 JDM, 12 PM and 2 ASS. Median age at death was 42.2 years. At time of death 18 had active disease, while 14 and 6 had grumbling and inactive disease respectively. Thirty-two (84.2%) had infection (11 bacteriologically proven), of which 19 died of septic shock. Other causes of death included: myocarditis (n=3), respiratory failure (n=4), cerebral bleed (n=1) and pulmonary embolism (n=1). One patient of ASS succumbed to rapidly progressive ILD, while another one died following RTX induced ARDS. 25 (65.7%) patients had died within 6 months of disease. In comparison to controls (n=50) they were older, had higher frequency of severe weakness (OR: 5.73, 95% CI: 1.62-20.26; p0.007), neck muscle weakness (OR:3.55, 95% CI :1.26-10.01; p:0.015), respiratory involvement (OR: 12.25, 95% CI :1.35-111.5; p:0.014) and thrombocytopenia (OR: 9.04, 95% CI :2.48-32.87; p:0.001). Conditional logistic regression was conducted using statistically significant variables (severe weakness, neck muscle weakness, thrombocytopenia and age at presentation) with death with in 6 months of presentation as outcome. In the final model only severe muscle weakness at onset remained as independent risk factor (OR: 10.83, 95% CI: 1.17-100.55; p:0.04).

Conclusions: Infections are the most common cause of in-hospital mortality in IIM patients. Severe muscle weakness if the single most important predictor of early mortality.

### P26 The Predictive Risk Factors for Opportunistic Infection during treatment for Polymyositis/dermatomyositis

#### Yumiko Sugiyama^1^, Ryusuke Yoshimi^1^, Mitsuhiro Takeno^2^, Yohei Kirino^1^, Shigeru Ohno^1^, Hideaki Nakajima^1^

##### ^1^Yokohama City University Medical Center, Yokohama, Japan; ^2^Nippon Medical School Graduate School of Medicine, Tokyo, Japan

###### **Correspondence:** Yumiko Sugiyama

Background/Purpose: Although concomitant infectious diseases are the predominant causes of death in patients with polymyositis (PM)/dermatomyositis (DM), intensive immunosuppressive treatment are necessary for severe cases. We have already reported that high initial dose of glucocorticoid and combination immunosuppressive therapy for induction therapy, and KL-6 levels at the baseline were independent risk factors for infection. Here we investigated the predictive risk factors for opportunistic infection during immunosuppressive treatment for PM/DM by assessing cytomegalovirus (CMV) antigen test as a barometer for immunocompromised status.

Methods: We retrospectively analyzed clinical features, laboratory data at baseline in the patients with PM/DM who had received initial treatment at six hospitals affiliated to Yokohama City University from 2003 to 2016. We also investigated initial therapeutic regimens and clinical outcomes including CMV antigenemia as a complication. We conducted univariate and multivariate analyses to extract risk factors for CMV antigenemia.

Results: One hundred sixty-eight (PM 42, DM 82, and clinically amyopathic DM (CADM) 44) were recruited. As initial therapies, oral prednisolone (PSL) was prescribed in all patients. Methylprednisolone (mPSL) pulse, intravenous cyclophosphamide (IVCY), and oral calcineurin inhibitor therapies were performed in 93 (56%), 50 (30%) and 86 (51%), respectively. Forty patients (24%) received combination therapy with IVCY and a calcineurin inhibitor. Fifty-two patients (31%) had CMV antigenemia within 6 months from initiation of immunosuppressant. A multivariate logistic regression analyses revealed that low PaCO2 (p = 0.023, OR 0.78) and old age (p = 0.040, OR 1.07) were independent risk factors for CMV antigenemia. As we focused on PM/DM-ILD patients, a multivariate logistic regression analyses revealed that low PaCO2 (p = 0.044, OR 4.96), above 0.6 mg/kg/day of initial PSL dose (p = 0.027, OR 3.56) and the treatment with oral calcineurin inhibitor (p = 0.007, OR 21.23) were independent risk factors for CMV antigenemia. Moreover, the patients with positive CMV antigenemia tended to infected which were needed antibiotic therapy with significant differences (OR 4.52, p < 0.001). There were no patients with organ dysfunction as CMV infection.

Conclusion: Although rapid and intensive therapies are required for PM/DM, appropriate monitoring, prophylaxis and early treatment for opportunistic infection are important, especially in patients who receive strong immunosuppressive therapy and who show low PaCO2 level and old age at baseline.

### P27 Pneumocystis jirovecii pneumonia (PCP) in Patients with Idiopathic inflammatory myopathy

#### Yong-Peng Ge, Xin Lu, Guochun. Wang, Si-Zhao Li

##### China Japan Friendship Hospital, Bejing, China

###### **Correspondence:** Yong-Peng Ge

Objective: Immunosuppressant medications increase the occurrence of Pneumocystis jirovecii pneumonia (PCP) in patients with idiopathic inflammatory myopathy (IIM). The objective of this study was to describe clinical features, risk factors and prognosis of PCP in IIM.

Methods: This retrospective cohort study analyzed data from 830 patients (2008-2018) diagnosed as having IIM. Clinical and laboratory data of these patients with PCP were compared to those of matched patients suffering from IIM but no PCP.

Results: A total of 28 (3.4%) cases of IIM with PCP including 27 DM (96.4) and 1 PM (3.6%), and 24 (85.7%) patients were diagnosed by bronchoalveolar lavage (BAL) fluid. Twenty two (78.6%) patients developed PCP within 6 months after onset, 27 (96.4%) patients were receiving average >30 mg of prednisone equivalent daily therapy and/or cytotoxic drugs for at least one month before developed to PCP. Most of patients (77.8%) had acute/subacute intestinal lung disease (ILD). The average CD4 lymphocyte counts of DM patients with PCP was 314.8 cells/mm3, which were significantly lower than those in DM patients without PCP (608.2 cells/mm3). Among myositis special antibodies (MSAs), anti-MDA5 antibodies occurred more frequent in DM patients with PCP than those without PCP (40.7% vs 17.5%, p=0.008). Multivariate analysis revealed that CD4 lymphocyte count < 200 cells/mm3 were the most important risk factors for PCP (OR 24.9, P < 0.001). Comparison with DM patients without PCP, those with PCP had higher mortality rate (44.4% vs 7.1%, p< 0.001).

Conclusions: PCP is not a rare disease in patients with IIM; especially in anti-MDA5 related DM. BAL is major tool for the diagnosis of PCP. Low CD4 lymphocyte count shows the risk factors for PCP.

### P28 Alterations of Body Composition in Myositis Patients Are Associated with Disease Activity, Duration and Muscle Involvement

#### Sabina Oreska, Maja Spiritovic, Hana Storkanova, Barbora Hermankova, Karel Pavelka, Ladislav Senolt, Herman Mann, Jiri Vencovsky, Michal Tomcik

##### Charles University, Prague, Czech Republic

###### **Correspondence:** Sabina Oreska

Objectives: Skeletal muscle, pulmonary and articular involvement in idiopathic inflammatory myopathies (IIM) limit the mobility/self-sufficiency of patients, and can have a negative impact on body composition. The aim was to assess body composition and physical activity of IIM patients and healthy controls (HC).

Methods: 54 patients with IIM (45 females; mean age 57.7; disease duration 5.8 years; PM, 22 / DM, 25 / IMNM, 7) and 54 age-/sex-matched HC (45 females, mean age 57.7) without rheumatic/tumor diseases were included. PM/DM patients fulfilled Bohan/Peter criteria for PM/DM. Anthropometric parameters and body composition were assessed (by densitometry: iDXA Lunar, and by bioelectric impedance: BIA2000-M), and physical activity was evaluated using Human Activity Profile (HAP) questionnaire. Disease activity was evaluated by MITAX and MYOACT activity score and muscle involvement by manual muscle test (MMT-8) and functional index 2 (FI2). Data are presented as mean±SD.

Results: Compared to HC, patients with IIM had a trend towards increased body fat % (BF%) (iDXA: 39.9±7.1 vs. 42.4±7.1 %, p=0.077), but significantly decreased lean body mass (LBM) (iDXA: 45.6±8.1 vs. 40.6±7.2 kg, p=0.001; BIA: 52.6±8.8 vs. 48.7±9.0 kg, p=0.023), and increased extracellular mass/body cell mass (ECM/BCM) ratio (1.06±0.15 vs. 1.44±0.42, p < 0.001). Higher ECM/BCM ratio reflects worse muscle predispositions for physical exercise, and deteriorated nutritional status. Compared to HC, IIM patients had significantly lower bone mineral density (BMD: 1.2±0.1 vs. 1.1±0.1 g/cm2, "p < 0.001"). Disease duration negatively correlated with BMD (r=-0.392, p=0.004) and LBM-BIA (r=-0.272, p=0.047). Disease activity assessed by both MITAX and MYOACT positively correlated with LBM-BIA (MITAX: r=0.294, p=0.031; MYOACT: r=0.335, p=0.013) and LBM-DXA (MITAX: r=0.341, p=0.012; MYOACT: r=0.368, p=0.007), as well as with basal metabolic rate (BMR; MITAX: r=0.336, p=0.014; MYOACT: r=0.351, p=0.010), and fat free mass (FFM; MITAX: r=0.338, p=0.014; MYOACT: r=0.356, p=0.009). CRP was positively associated with BF% assessed both by DEXA (r=0.276, p=0.035) and BIA (r=0.306, p=0.025). Higher BF%-DEXA was associated with worse physical endurance (FI2: r=-0.311, p=0.026) and worse ability to perform physical activity (HAP: r=-0.292, p=0.032). MMT-8 scores negatively correlated with ECM/BCM ratio (r=-0.385, p=0.006).

Conclusions: Compared to healthy age-/sex-matched individuals we found significant negative changes in body composition of our IIM patients, which are associated with their disease activity and duration, inflammatory status, skeletal muscle involvement, and physical activity. These data could reflect their impaired nutritional status and predispositions for physical exercise, aerobic fitness and performance.

Acknowledgements: Supported by AZV NV18-01-0016116-33574A, MHCR 023728 and GAUK 312218.

### P29 Prevalent Vertebral Fractures incur high risk of future fractures in inflammatory myositis

#### Sujata Ganguly, Able Lawrence, Ramnath Misra, Latika Gupta, Saroj Sahoo

##### Sanjay Gandhi Post Graduate Institute of Medical Science, Uttar Pradesh, India

###### **Correspondence:** Sujata Ganguly

Objective: To assess accrual of new vertebral fractures (VF) in patients with inflammatory myositis over a period of time.

Methods: Hundred patients who were previously enrolled for a cross-sectional study on prevalence of asymptomatic VF were telephonically requested to review with repeat dorsal and lumbar Spinal radiographs and dual‐energy X‐ray absorptiometry (DEXA) and Bone mineral Density (BMD) evaluation 3 years after the initial assessment. Radiographs were scored using Genant’s semi-quantitative technique by two independent observers. Involvement of new vertebra, worsening of fracture grade of a previously fractured one or new site of fracture within a fractured vertebra were recorded as a fracture event. Demographics, previous fractures, menopausal status and disease related variables were recorded. All results are expressed in median and interquartile range. Statistical analysis was done using Prism Graph Pad 8.0 (trial version for windows 8.0).

Results: Radiographs from 21 patients (5 men and 16 women) of median age 42 years were reviewed. Of these, 7 patients had Polymyositis, 9 dermatomyositis and 5 overlap myositis. Fifteen of the 21 (71.4%) had VF at baseline. At 61.9 patient years of follow-up, twelve of 15 (75%) patients with previous VF had new events (p=0.009), while none of those without previous fractures had new fractures. There was an increase in total number of fractures (25 to 43, p: 0.01). Accounting for worsening of previous fractures as well, there were 25 new fractures events. Most patients had more than 1 fracture (11 of 15,73.3 %) although most of these (31 of 43, 72.09%) were grade 1 VF. The increase in lumbar fractures was greater than thoracic. (p=0.04, RR:2.2). Patients with previous VF have 5 times higher risk of developing a new VF. Patients with old VF accrue fractures at a rate of 8.5/year. Of the 13 patients who underwent DEXA scans, 3 had osteoporosis and 5 were osteopenic. T scores at the middle (r=-0.7, p-0.015) and lower third (r- -0.7,p- 0.0094) of radius correlated with fracture number. Conventional risk factors such as age, weight, and gender did not differ between fracture progressors and non-progressors. Change in disease activity had no bearing on progression of fractures (p=ns).

Conclusion: Patients with inflammatory myositis with a prior asymptomatic vertebral fracture have a very high risk of subsequent vertebral fractures irrespective of disease activity.

### P30 Poor muscle endurance of swallowing muscles in myositis patients with dysphagia

#### Alba Azola, Tae Chung, Lisa Christopher-Stein

##### Johns Hopkins University School of Medicine, Lutherville, USA

###### **Correspondence:** Alba Azola

Introduction: The prevalence of dysphagia in patients with immune-mediated inflammatory myopathies (myositis) has been reported to be as high as 60%. Aspiration pneumonia is one of the main causes of mortality in patients affected by myositis, especially in those with chronic deterioration. However, the pathophysiology of myositis-related dysphagia has not been well characterized.

Objectives: The goal of this study was to characterize the pathophysiologic changes in swallowing of myositis patients by examining videofluoroscopic swallow studies (VFSS) for kinematic, temporal, and functional measures of swallowing. Methods: VFSS of 23 myositis patients (IBM = 7, Necrotizing = 2, DM = 13, PM =1, age range = 21 - 81) complaining of dysphagia were collected, and frame by frame analysis of temporal and kinematic measures were performed. The swallows of each subjects were rated by MBSImPª© and Penetration-Aspiration Scale. Swallowing measures from patients were compared to those from 64 healthy subjects (ages range = 18 - 90) by Wilcoxon rank-sum tests.

Results: In patients with myositis, food entered the pharynx earlier relative to the initiation of swallowing than in healthy participants (p < 0.0001). Myositis patients had a significantly shorter duration of upper esophageal sphincter (UES) opening and laryngeal vestibule closure than healthy subjects (p < 0.0001). There were no statistically significant differences in AP diameter of the UES between healthy subjects and myositis patients (p = 0.9). In myositis patients, several MBSImPª© component scores indicated impairment, particularly those related to residue after swallowing, base of tongue movement, and posterior pharyngeal wall movement. Aspiration was observed in 26% of the patients.

Conclusion: Poor endurance of swallowing muscles, which causes short UES opening and other changes in swallowing pattern, appears to be an underlying mechanism of dysphagia in patients with myositis. Our findings suggest that endurance exercises of the tongue and submental muscles are potential therapeutic targets for preservation of swallowing function in myositis patients.

### P31 miR-1 Is a Novel Biomarker for Polymyositis/dermatomyositis-associated Interstitial Lung Disease

#### Yumiko Sugiyama, Ryusuke Yoshimi, Mitsuhiro Takeno, Yohei Kirino, Shigeru Ohno, Hideaki Nakajima

##### Yokohama City University Medical Center, Yokohama, Japan; ^2^Nippon Medical School Graduate School of Medicine, Toyko, Japan

###### **Correspondence:** Yumiko Sugiyama^1^

Background/Purpose: Although intensive immunosuppressive treatment are necessary for the severe cases with polymyositis (PM)/dermatomyositis (DM), the prognostic factors or disease activity indices for PM/DM have not established yet. On the other hand, microRNAs are small non-coding RNAs, some of which have a certain function such as a transcriptional regulation. MicroRNA-1 (miR-1) has been shown to be associated with myocyte differentiation, decreased in muscle biopsy sample from patients with inflammatory myopathies. Here we investigated the association between serum miR-1 level and clinical course of PM/DM patients.

Methods: We retrospectively analyzed clinical features, laboratory data at baseline in patients with PM/DM patients who had received initial treatment at Yokohama City University Hospital from 2008 to 2017. We also investigated initial therapeutic regimens, clinical outcomes, and episodes of serious infection The serum samples from PM/DM were collected before and after starting treatment and those from healthy controls were recruited from the biobank institution in the hospital. The serum miR-1 levels were measured by quantitative real-time PCR.

Results: Twenty-two patients (PM 4, DM 11, clinically amyopathic DM (CADM) 7) were recruited. The mean age was 63.5 ± 8.5 years, 13 (59%) were female, and 14 patients (64%) had interstitial lung disease. The serum miR-1 level was significantly elevated in PM /DM patients as compared to healthy control (p = 0.008). In PM/DM patients, the serum miR-1 level was significantly decreased by treatment (p = 0.03). There was a correlation between serum CK and miR-1 levels in PM/DM patients (p = 0.005, r = 0.58), although there was no correlation between serum CK and miR-1 levels in PM/DM-ILD patients. We identified the cutoff value of serum miR-1 level from the two standard deviations in healthy controls, and divided the PM/DM-ILD into two groups, high miR-1 group, and normal miR-1 group, by serum miR-1 level at baseline. Although there were no significant differences in the clinical data and the initial prednisolone (PSL) dose between the two groups, PSL dose at 16 weeks and cumulative PSL dose until 16 weeks were significantly higher in the high miR-1 group (p = 0.025 and p = 0.036, respectively). We also found that serious infections were significantly more frequent in the high miR-1 group (p =0.026).

Conclusion: We propose serum miR-1 as a promising novel biomarker for predicting therapeutic response in PM/DM-ILD.

### P32 Clinical significance of radiological pattern of HRCT and macrophage activation for polymyositis and dermatomyositis patients with different MSAs

#### Yu Zuo, Lifang Ye, Min Liu, Fang Chen, Xin Lu, Guochun Wang, Xiaoming Shu

##### China-Japan Friendship Hospital, Beijing, China

###### **Correspondence:** Yu Zuo

Objectives: Polymyositis and dermatomyositis-associated interstitial lung disease (PM/DM-ILD) have variable courses with different myositis specific autoantibodies (MSAs) and different histopathological and radiological types. The aim of this study was to examine the different distributions of radiological types of different MSAs group, to identify the prognostic value of high-resolution computed tomography (HRCT) in PM/DM-ILD and to explore the possible mechanism.

Methods: The present study included 165 patients with PM/DM-ILD. The distribution of HRCT radiological type with different MSAs was analysed as well as the relationship between radiological features with the ILD course and prognosis, especially consolidation in the posterobasal portions in HRCT of patients with anti-MDA5 and anti-aminoacyl-tRNA synthetase antibodies (ARS). In addition, immunohistochemistry analysis using anti-human CD163 antibody was performed on the lung sections of 15 patients with PM/DM-ILD to assess the macrophage activation.

Results: Organizing pneumonia (OP) pattern was dominant in HRCT of 165 PM/DM-ILD patients, especially in patients with anti-SAE (6/6, 100%) and patients with anti-MDA5 (46/62, 74.2%). The ratio of OP pattern and nonspecific interstitial pneumonia (NSIP) pattern was almost equal in patients with anti-ARS and NSIP pattern was associated with mild clinical course and favorable prognosis. Among patients with anti-MDA5, consolidation with a gravitationally dependent gradient in HRCT was related to rapidly progressive ILD (RP-ILD) and overall mortality. Ferritin levels >1000 ng/mL, elevated LDH and several tumor markers including CEA, CA125, CA19-9, SCC, and the presence of anti-Ro52 were significantly associated with the lower consolidation pattern in patients with anti-MDA5. Multivariate analysis revealed that ferritin levels >1000 ng/mL (odds ratio, 12.3; P=0.009), elevated CEA (odds ratio, 5.8; P=0.046) and CA19-9 (odds ratio, 7.8; P=0.018) were independent predictors of lower consolidation pattern. Meanwhile, immunohistochemical analysis of lung specimens obtained from 15 patients with DM-related ILD showed that compared with patients with anti-ARS, accumulation of CD163-positive macrophages at alveolar spaces seemed to be more evident in the lungs of patients with anti-MDA5 or only with anti-Ro52. The macrophage infiltration in the lung of cases with RP-ILD was apparently severe.

Conclusion: HRCT patterns at diagnosis can help predict the prognosis of patients with PM/DM-ILD. Our results also suggest the importance of macrophage activation in the disease.

### P33 Reliability of PET/TC cancer screening in a large cohort of patients diagnosed with idiopathic inflammatory myopathy

#### Albert Gil-Vila, Ernesto Trallero-Araguás, Marcelo Alvarado-Cardenas, Iago Pinal-Fernandez, Xavier. Martínez-Gómez, Joan Castells-Conesa, Albert Selva-O'Callaghan

##### Vall d'Hebron General Hospital, Barcelona, Spain

###### **Correspondence:** Albert Gil-Vila

Objectives: Cancer associated myositis develops in a third of patients with idiopathic inflammatory myopathy (IIM), mainly dermatomyositis (DM). Previous data suggest that PET/CT seems to be a useful approach for cancer screening in those patients. Our aim was to ascertain the reliability of PET/CT for cancer screening in patients diagnosed with myositis from 2010 to 2018.

Methods: A total of 115 adult patients (74 female) were diagnosed with IIM (59 DM -20 of which amyopathic-, 14 IMNM, 36 overlap -28 ASS, 10 of them without myositis, and 8 scleromyositis-, and 6 PM). PET/CT was performed at disease onset, other confirmatory tests were carried out when data obtained with PET/CT was inconsistent in terms of cancer or not. Occult cancer was defined when cancer was diagnosed by means of PET/CT in a patient without cancer-concerning symptoms.

Results: PET/CT was performed mostly in patients with DM [30 out of 39 classical DM (77%), and 9 out of 20 amyopathic form (45%)]. The two main causes because some DM patients do not received a PET/CT were MDA5 positive rapidly progressive ILD (8 cases) and a previous diagnosis of cancer (7). PET/CT was also performed in 10 out of 14 IMNM patients, 12 out of 36 overlap myositis (9 anti-synthetase positive), and 2 out of 6 PM. PET/CT raised the suspicion of cancer in 14 patients with DM/ADM. Complementary studies confirmed cancer diagnosis in 8 cases, all of them occult. Among the other myositis phenotypes only 5 out 24 patients had PET/CT findings that prompted to further evaluation, being all of them negative for cancer. Only 2 DM patients, with a normal PET/CT at disease onset developed a cancer during follow-up, one and five years respectively after DM diagnosis. None of the 104 patients in whom PET/CT was not performed, was normal or had suspicious features that were not confirmed as cancer, developed any type of malignancy at follow up (median 3 years, IQR 0.9-5.2 years). PET/CT in patients with cancer-associated DM/ADM yields a PPV of 57%, NPV of 92%, with a sensitivity of 80% and specificity of 79%., and an AUC ROC of 0.79 that means a good test for screening purposes.

Conclusions: PET/CT screening in real clinical practice is a good option for cancer screening in patients with DM/ADM, allowing diagnosis of occult cancer. Clinical judgement is of paramount relevance to perform this test in cases of myositis phenotypes different than DM.

### P34 Tumour Infiltrating Lymphocytes quantification in Cancer-Associated Dermatomyositis

#### Javi Ros^1^, Julia Lostes^1^, Paolo Nuciforo^2^, Cristina Viaplana^1^, Jóse Milisenda^3^, Ernesto Trallero-Araguás^1^, Iago Pinal-Fernandez^1^, Albert Gil-Vila^1^, Josep Maria Grau-Junyent^3^, Rodrigo Dienstmann^1^, Albert Selva-O'Callaghan^1^

##### ^1^Vall d'Hebron General Hospital, Barcelona, Spain; ^2^Instituto de Salud Carlos III, Madrid, Spain; ^3^Idibaps-Cellex-Hospital Clinic of Barcelona, Barcelona, Spain

###### **Correspondence:** Javi Ros

Objectives: Tumour infiltrating-lymphocytes (TILs) are a validated prognostic marker in different neoplasms and have been linked to anti-tumour immune response. Dermatomyositis (DM) is an autoimmune disorder frequently associated with cancer. Our aim was to quantify the intensity of TILs in a series of patients diagnosed with cancer-associated dermatomyositis (CADM).

Method: A total of 16 adult patients (12 female, 4 amyopathic), with mean (SD) age 55.3 (4.8) diagnosed with CADM (cancer occurring within 3 years of the DM diagnosis), were proposed for the study of the presence and density of TILs. Immunological profile and DM outcome in regard with cancer evolution and chemotherapy were also analysed. Histopathologic analysis of the proportion of TILs was done in whole sections of tumour tissue stained with hematoxylin and eosin. TILs were quantified according to the 2014 Guidelines developed by the International TILs Working Group. Briefly, TILs are reported as an overall percentage of the stromal area within the borders of the invasive tumour that is covered by mononuclear immune cells.

Results: Nine out of 16 patients had remaining tissue available for TILs quantification. Ten patients, 7 anti-TIF1g (+), 2 anti-NXP2 (+), and 1 anti-MDA5 (+) presented as paraneoplastic (DM runs in parallel with the activity of cancer), and the other 6, 4 anti-TIF1g (+), 1 anti-MDA5 (+), and 1 without antibodies, flared after chemotherapy treatment. The types of neoplasms studied for TILs quantification were as follows: small cell lung cancer (SCLC, 3 patients), breast cancer (2 patients), cervical cancer (2 patients), colorectal, and ovarian cancer (one patient each). Only the 2 SCLC cases had low percentage of TILs (1%) and 6 patients had “hot” tumours (> 20% TILs), with the all gynaecological cancers displaying unusually high levels (30%, 50%, 60%).

Conclusions: Microenvironment TILs density is a potential predictor of response to immune checkpoint inhibitors (ICIs). Until now, patients with autoimmune disorders have been consistently excluded from clinical trials with these agents. CADM may be a surrogate of an active immune response, also in the tumor microenvironment with high infiltration with TILs. Our preliminary findings open the door for careful evaluation of ICIs in patients with CADM. We will expand our cohort and perform more in-depth biomarker research (tumour mutation burden and PDL1 expression).

### P35 Traditional and disease-related risk factors for arterial and venous thrombotic events (TE) in idiopathic inflammatory myopathies (IIM)

#### Antonella Notarnicola, Simone Barsotti, Linnea Näsman, Quan Tang, Marie Holmqvist, Ingrid E. Lundberg, Aleksandra Antovic

##### Karolinska Institute and Karolinska University Hospital, Stockholm, Sweden

###### **Correspondence:** Antonella Notarnicola

Objectives: To assess the prevalence of traditional and disease-related risk factors for arterial and venous TE in patients with IIM by comparing those reporting TE (cases) with those without history of TE (comparators). To compare clinical characteristics, autoantibody profile inclusive antiphospholipid antibodies (aPL) and serum levels of adhesion molecules (VCAM, ICAM and e-selectin) between cases and comparators as well as between cases reporting arterial versus venous TE.

Methods: Using national and international registries, and medical charts, we identified 58 cases and 158 comparators with IIM followed at Karolinska University Hospital between 1993 and 2014. Information on gender, age at the time of diagnosis, IIM subgroup, presence of interstitial lung disease (ILD), myositis specific antibodies (MSAs), was retrospectively collected. Information on traditional risk factors for arterial and venous TE (essential hypertension, diabetes mellitus, dyslipidemia, smoking, malignancy) was retrieved for both groups. Serum levels of aPL and adhesion molecules were analyzed in stored sera from the time of diagnosis in both groups, before TE in cases and in 40 age and gender matched heathy controls (HC).

Results: One out of 5 IIM patients (22,92%) had suffered from at least one TE, which was observed especially during the first 5 years after diagnosis. Myocardial infarction was the most frequent TE, followed by pulmonary embolism and deep venous thrombosis. In the multivariate analysis, male gender and older age were independent risk factors for TE. Essential hypertension had statistically significant higher prevalence in cases than comparators. Arterial TE was more common in polymyositis, while venous TE occurred more frequently in patients with dermatomyositis, history of malignancy and in those with MSAs. At time of IIM diagnosis, the prevalence of aPL was 6% with no difference between cases and comparators. Significantly higher levels of VCAM and ICAM were obtained in IIM patients compared to HC but without difference between cases and comparators. Lower levels of e-selectin were associated with higher odds of developing TE, especially in males and older patients, with no difference between arterial and venous TE.

Conclusions: A high risk of arterial and venous TE should be taken into account in patients with IIM, particularly close to time of diagnosis, with extra attention in male patients and older individuals. Preventive measures should be considered especially in patients with concomitant essential hypertension and malignancy. Lower serum levels of e-selectin might predict TE in IIM patients but the mechanism for this risk factor is not known.

### P36 The potassium channel KCNK2 is a regulator of immune cell trafficking and inflammatory responses in idiopathic inflammatory myopathies

#### Thomas Müntefering

##### University Clinic Münster, Münster, Germany

Objectives: KCNK2, a two-pore domain potassium channel, has been implicated as important regulator of leukocyte transmigration into the central nervous system (CNS). In experimental autoimmune encephalomyelitis (EAE), an animal model of multiple sclerosis, Kcnk2-/- mice demonstrated an increased disease severity with facilitated immune-cell migration into the CNS. An upregulation of cellular adhesion molecules on brain endothelial cells in Kcnk2-/- mice were found as potential underlying mechanisms. Of note, immune-cell infiltration into the muscle is also a pathogenic hallmark of idiopathic-inflammatory-myopathies (IIM), however the underlying mechanisms remain to be elucidated. Following our previous results, we therefore investigated if KCNK2 might also be involved in the peripheral autoimmune responses of IIMs.

Methods: Murine muscle tissue was dissociated and primary murine microvascular endothelial cells (PMMEC) and primary murine muscle cells (PMMC) were isolated by magnetic-activated cell sorting technique. Purified primary cells were cultured and KCNK2 gene expression was measured by qPCR whereas protein expression was detected by western blot. Pro-inflammatory and adhesion molecule expression in stimulated primary cells were investigated via flow cytometry. To test the functional role of KCNK2 for the interaction of T cells and PMMEC in vitro, we used a flow assay. Further, the regulatory role of KCNK2 for the migratory behavior of T cells in vivo was characterized by intravital microscopy of cremaster muscle microvessels.

Results: In accordance to our previous results, we found KCNK2 expression in primary murine microvascular endothelial cells (PMMEC) and in primary murine muscle cells (PMMC) on gene and protein level. Stimulation with pro-inflammatory cytokines led to a downregulation of KCNK2 expression on differentiated PMMC and PMMEC. KCNK2 knockout or pharmacological blockade with spadin increased pro-inflammatory and adhesion molecule expression in muscle and endothelial cells. Correspondingly, we found significantly more adhered T cells on KCNK2-/- PMMEC in vitro under low sheer flow and in vivo in cremaster muscle microvessels.

Conclusion: In summary, KCNK2 might be critically involved in the regulation of immunological processes in the skeletal muscle, especially immune cell trafficking. These regulatory mechanisms might be impaired in IIMs and therefore represent targets for new therapeutic strategies.

### P37 Infection in patients with Idiopathic Inflammatory Myopathies

#### Yong-Peng Ge, Guo-Chun Wang, Xin Lu, Si-Zhao Li, Lin-Rong He

##### China Japan Friendship Hospital, Beijing, China

###### **Correspondence:** Yong-Peng Ge

Objectives: To assess the incidence and characteristics of infections in idiopathic inflammatory myopathies (IIM) patients and to analyze the risk factors for infections on clinical presentation and biochemical findings of IIM.

Methods: The medical records of IIM patients followed up in a single medical center from January 2008 to December 2017 were retrospectively reviewed.

Results: In the 779 IIM patients, 215 (27.6%) suffered from infections. The incidence of infection in dermatomyositis (DM) (29.8%) was more than polymyositis (PM) (18.5%). Pulmonary infection was the most common infection site (66.5%). Opportunistic infections comprised 37.7% of all infectious episodes. Multivariate analysis showed that pulsed methylprednisolone (OR 3.22; 95% CI 1.60-6.48; P = 0.001), age of onset >50 years (OR 1.02; 95% CI 1.00-1.03; P = 0.011), anti-melanoma differentiation-associated gene 5 (MDA5) antibody (OR 1.93; 95% CI 1.20-3.11; P = 0.007), lymphocyte count < 1200/mm3 (OR 2.85; 95% CI 1.89-4.30; P < 0.001) and interstitial lung diseases (ILD) (OR 2.03; 95% CI 1.30-3.71; P = 0.002) are risk factors for infections. Survival analysis showed that survival rate in infection group was lower than no-infection group (69.8% vs 92.9%, P < 0.001).

Conclusion: Among hospitalized individuals with IIM, infection is frequency and the leading cause of mortality. Anti-MDA5 antibody, lymphopenia, ILD, old age of onset and treatment with methylprednisolone pulse are contributing factors for the development of infections in IIM patients. Strategies to lower infection risk in IIM patients should be evaluated to improve disease outcomes.

### P38 Frequency, Risk factor, Outcome and Prognostic factor in Calcinosis of Adult Idiopathic Inflammatory Myopathy: Single Center Cohort Study

#### Wei Jiang, Kanbo Yang, Sizhao Li, Xiaolan Tian, Lu Zhang, Xin Lu, Guochun Wang

##### China-Japan Friendship Hospital, Beijing, China

###### **Correspondence:** Wei Jiang

Objective: To determine the prevalence of calcinosis and to analyze clinical features and risk factors for calcinosis in adult idiopathic inflammatory myopathy(IIM). To assess outcomes in adult patients with calcinosis, with emphasis on survival probability and prognostic factors.

Methods: A total of 480 patients with clinical data were collected. Multiple factors logistic regression model analysis was performed to assess the risk factors for calcinosis in adult IIM. Survival probability at 1, 5 and 10 years was estimated according to the Kaplan–Meier method. Cox regression was used to analyze the prognostic factors for calcinosis.

Results: The frequency of calcinosis in adult IIM was 4.6% in our cohort. Patients with calcinosis had a longer disease duration(P=0.000)and more myothenia(P=0.038), panniculitis(P=0.011), perlungual erythematosus(P=0.011), skin ulcer(P=0.000), raynaund’s phenomenon(P=0.035). The anti-NXP2 antibody(P=0.000)and anti-MDA5 antibody(P=0.021)were more frequently found in patients with calcinosis. Multiple factors logistic regression showed that anti-NXP2 antibody(OR=10.899, 95%CI 2.593, 45.816), long diseases duration(OR=1.105, 95%CI 1.008, 1.021) and skin ulcer(OR=31.585, 95%CI 10.683, 93.387) were the risk factors of calcinosis in adult patients. The survival probability at 1, 5 and 10 years respectively were 95.8%, 91.3%, 91.3%. Patients with calcinosis improvement had lower baseline calcification VAS score(P=0.035), higher rate of V sign(P=0.033) and clinical remission within 3 months(P=0.04). Clinical remission within 3 months(HR=0.236) was the protective factor for calcinosis.

Conculusion: Calcinosis was uncommon clinical feature in our cohort. Long disease duration, skin ulcer and anti-NXP2 positive were independent risk factors for calcinosis. Adult patients with calciniosis were lower mortality. Clinical remission within 3 months was the protective factor for calcinosis, which indicated that aggressive treatment for patients with calcinosis at very early stage of disease may improve the outcome of calcinosis.

### P39 A single center experience using a clustering approach to identify clinico-serological phenotypes in Idiopathic Inflammatory Myopathies

#### Valérie Leclair, Angeles Shunashy Galindo Feria, Ingrid E. Lundberg, Leonid Padyukov, Lina Marcella Diaz-Gallo

##### Karolinska Institutet, Stockholm, Sweden

###### **Correspondence:** Valérie Leclair

Background: A large number of idiopathic inflammatory myopathy (IIM) patients are presenting extramuscular features. Novel approaches to identify more homogenous IIM subgroups are explored to improve the classification of these patients for research and clinical purposes. The aim of this project is to use a clustering approach to identify subgroups of IIM patients based on serological and clinical features.

Methods: We included 319 Swedish IIM subjects followed in the EuroMyositis registry, regardless of their specific subset. The occurrence of 18 autoantibodies was determined: anti-Jo1, -PL7, -PL12, -EJ, -OJ, -SRP, -RNP, -Ro, -Mi2, -TIF1g, -MDA5, -Ku, -PmScl, -PmScl75, -PmScl100, -SAE1, -NXP2 and -HMGCR. A cluster analysis was done using Gower distance matrix followed by partition around medoids cluster calculation. The number of clusters was validated using Silhouette metric. Logistic regression was applied to estimate the p-value, odds ratios (OR) and 95% confidence intervals (CI) of having a clinical manifestation depending on the cluster assigned.

Results: Figure 1 presents six (1-6) clusters based on the occurrence of 18 autoantibodies, while Table 1 shows the relations between specific clinical manifestations and each cluster. Each cluster is grouped around a medoid representing the predominant antibody profile of that group: 1) antibody negative (49%), 2) Jo1+Ro+ (11%), 3) Jo1-Ro+ (19%), 4) Jo1+Ro- (9%), 5) PmScl+ (6%), and 6) TIF1g+ (7%). Compared to the other clusters, muscle weakness was significantly more prevalent in cluster 1. In cluster 2, ILD was strongly overrepresented, and arthritis and mechanic’s hand were common. Heliotrope rash and ulcerations were more frequent in cluster 3 compared to the other clusters. Subjects in cluster 4 had significantly more ILD, arthritis and mechanic’s hand, but to a lesser extent than cluster 2. For cluster 5, Raynaud, calcinosis and ulceration were more frequent than in other clusters. Heliotrope rash and Gottron´s papules or sign were more frequent in cluster 6.

Conclusion: These preliminary results indicate that IIM individuals can be grouped in six clinico-serological phenotypes. Further studies are needed to validate these results in other cohorts.

### P40 Creatinine- and cystatin C-estimated glomerular filtration rate for estimating renal function during inclusion body myositis

#### Olivier Mangin^1^, Jean Philippe Bertocchio^2^, Yves Allenbach^1^, Gerard Maruani^2^, Pascal Houillier^2^, Olivier Benveniste^1^

##### ^1^Hôpital Pitié Salpêtrière, Paris, France; ^2^Hôpital Européen Georges-Pompidou, Paris, France

###### **Correspondence:** Olivier Mangin

Creatinin is creatin metabolite. The main glomerular filtration rate (GFR) estimation formulas use creatininemia and thus rely upon muscular mass. Inclusion body myositis (IBM) is an acquired myopathy leading to amyotrophia. It only concerns senior patients, a population with a high incidence of chronic kidney disease (CKD). Our objective was to find out whether GFR estimations based on creatininemia are reliable in IBM patients. 20 patients from the internal medicine service of the Pitié Salpêtrière hospital in Paris had their GFR simultenously measured with 51Cr-EDTA (GFRm) and estimated (GFRe) with MDRD, CKD-EPI creatinin and CKD-EPI C cystatin. Creatininemia and 24h urines were sampled the day before. The body composition (lean appendicular mass) was measured by X ray biphotonic absorptiometry (DXA). Height men and twelve women, aged 65±9 years were included. Their mean lean appendicular mass and creatininemia were in the lower values of normal, respectively -1.85 [-2.50;-1.18] and 43.5μM [35,3-56,8]. GFRm was 78±18 mL/min/1.73m2, height patients (40%) had a CKD according to KDIGO 2012 criteria, and four of them had a GFRm < 60mL/min/1.73m2 ; GFRm were 157±64, 109±18, 54±17 mL/min/1.73m2, respectively with MDRD, CKD-EPI creatinin and CKD-EPI C cystatine formulas. The average measurement bias were 80±55, 32±17 and -24±17mL/min/1.73m2, respectively. There was no statistically significant correlation between the lean appendicular mass and GFRm measurement bias. Only the patients without CKD were correctly classified, whatever the formula. Amongst these patients with low muscular mass, GFR estimation based on creatininemia or C cystatine is biased, especially with MDRD formula. This measurement bias prevents the diagnostic and classification of CKD in these patients. Using those formulas may lead to a delay in the care of CKD and also overdoses of drugs eliminated by the kidneys. In our population, using a CKD-EPI creatinin eGFR threshold of 100mL/min/1.73m2, the sensitivity to screen stage ≥ 3 CKD is 100% and specificity 87.5% (χ2=11.67). Surprisingly the bias between eGFR and mGFR were not correlated with lean appendicular mass. This may be explained by the small size of our population, but also by the muscle quality which isn't assessed by DXA. During IBM, GFR is surestimated by usual estimation formulas in spite of a moderately lower muscular mass. GFR estimation formulas don't allow to screen CKD in this population. We propose that each IBM patient with a CKD-EPI creatinin eGFR < 100mL/min/1.73m2 has a measure of GFR with

### P41 Design of Phase 3 Study of Lenabasum for the Treatment of Dermatomyositis

#### Victoria P. Werth^1^, Chester V. Oddis^2^, Ingrid E. Lundberg^3^, David Fiorentino^4^, Caitlin Cornwall^5^, Nancy Dgetluck^5^, Scott Constantine^5^, Barbara White^5^

##### ^1^University of Pennsylvania, Philadelphia, USA; ^2^University of Pittsburgh, Pittsburgh, USA; ^3^Karolinska Institutet, Stockholm, Sweden; ^4^Stanford University, Stanford, USA; ^5^Corbus Pharmaceuticals, Massachusetts, USA

###### **Correspondence:** Victoria P. Werth

Objectives: To provide Phase 3 efficacy and safety data on lenabasum for the treatment of DM.

Methods: Lenabasum is a synthetic, non-immunosuppressive, selective cannabinoid receptor type 2 agonist that activates resolution of innate immune responses. Lenabasum had acceptable safety and tolerability and improved efficacy outcomes in the double-blinded, randomized, placebo-controlled Phase 2 trial in dermatomyositis (DM) subjects with refractory, skin-predominant involvement. Phase 3 trial design was based on Phase 2 data, input from a steering committee of experts in DM clinical trials, and recommendations made by FDA at an End-of-Phase 2 meeting. Input from EMA and PMDA is pending.

Results: A global, double-blind, randomized, interventional design was chosen to provide an unbiased assessment of the efficacy, safety and tolerability of lenabasum 20 mg bid and 5 mg bid compared to placebo in the treatment of DM. A 52-week treatment duration was selected to provide safety and efficacy data adequate to support chronic treatment of DM patients. Eligibility criteria include DM patients with active disease, allowing for a range of muscle, skin, and other disease manifestations. Subjects must be on stable doses of current DM treatments with any background immunosuppressive medications allowed except prednisone ≥ 20 mg per day or equivalent. This inclusivity allows testing of efficacy and safety of lenabasum in the setting of current treatment practice and reduces risk of disease flare early in the study. The primary efficacy outcome is change from Baseline in 2016 ACR/EULAR Total Improvement Score (TIS). This composite outcome has six domains that broadly capture improvement in disease activity, is relevant to the range of manifestations in DM, and is applicable to the assessment of efficacy in the target patient population. Secondary efficacy outcomes were chosen to reflect how the subject functions (Short Form – 36 physical functioning domain), assess major organ involvement (MMT-8, CDASI activity score, and a new Investigator Global Assessment scale of skin activity designed specifically for this study), and measure lung function (FVC). Change in oral corticosteroid dose also will be captured.

Conclusions: To our knowledge, this is the first Phase 3 study in DM with a new molecular entity and primarily sponsored by industry. As such, agreement with experts and regulatory authorities on design represents a step forward in the development pathway of new treatments for DM.

### P42 Patient reported outcome core set for adult idiopathic inflammatory myopathies

#### Christopher Mecoli^1^, Malin Regardt, Jin Kyun Park, Ingrid de Groot, Catherine Sarver, Merrilee Needham, Marianne de Visser, Clifton Bingham III, Ingrid Lundberg, Yeong Wook Song, Helene Alexanderson, Lisa Christopher-Stine

##### Johns Hopkins University School of Medicine, Baltimore, USA

###### **Correspondence:** Christopher Mecoli

Objective: Currently, the patient-reported outcome measures (PROMs) included in the IMACS Core Set (HAQ-DI, Patient Global Activity Visual Analog Scale) fall short in encapsulating the myositis life experience of adult IIM patients. Our goal is to develop a patient-reported outcome core domain set specific to idiopathic inflammatory myopathies (IIM) following methodology outlined by Outcome Measures in Rheumatology (OMERACT).

Methods: The OMERACT Myositis Working Group consists of IIM experts and qualitative methodologists from Sweden, The USA, The Netherlands, Australia, and South Korea, as well as IIM patient research partners. Over a 6-year period, 11 international focus groups and three modified Delphis were performed with multiple stakeholder groups including patients, healthcare providers, and primary caregivers. From these results, we developed a preliminary PRO core domain set in agreement with OMERACT methodology.

Results: Four domains - muscle symptoms, fatigue, levels of physical activity and pain, achieved widespread consensus to be assessed in all clinical research studies. Three additional domains - lung, joint, and skin symptoms were considered mandatory only in specific circumstances. Additional domains (including cognitive effects, emotional distress, sleep quality, and GI symptoms) were deemed important for further research. This core set was strongly endorsed in OMERACT 2018 by patients, healthcare providers, and qualitative researchers.

Conclusion: We propose a PRO core set for patients with adult IIM. The next steps of the Myositis OMERACT Working Group are to develop and validate instruments to measure these domains.

### P43 Preliminary Response to Janus Kinase (JAK) Inhibition with Baricitinib in Refractory Juvenile Dermatomyositis

#### Hanna Kim, Samantha Dill, Michelle O'Brien, April Brundidge, Lisa G. Rider, Robert A. Colbert

##### National Institute of Arthritis and Musculoskeletal and Skin; Bethesda, USA

###### **Correspondence:** Hanna Kim

Objectives: Juvenile dermatomyositis (JDM) is a systemic autoimmune disease with a prominent interferon (IFN) signature. Treatment is generally empiric, requiring prolonged high-dose steroids and often ≥2 other immunosuppressive medications. In a compassionate use program, we assessed efficacy and safety of baricitinib (JAK 1/2 inhibitor) in active refractory JDM.

Methods: Active (based on ≥3 core set measures (CSM)) and refractory (active after high dose steroids and ≥2 other medications, including ≥1 biologic therapy) JDM subjects were enrolled after washing out biologic agents other than IVIG. Bariticinib was dosed based on weight and renal function. Primary outcome was reduction in symptom daily diary score (DDS) of weakness, fatigue, musculoskeletal pain, and rash. Other assessments included International Myositis Assesment and Clinical Studies (IMACS) disease activity CSMs, including manual muscle testing (MMT8), as well as Cutaneous Dermatomyositis Disease Area and Severity Index (CDASI). Paired t-test was used to compare baseline to 12 weeks. Safety and tolerability were also assessed.

Results: Four JDM patients (5.8-20.7 years old) who had failed high dose steroids, IVIG, and at least 1 other medication were enrolled (NCT01724580). Patients received baricitinib 4-8 mg/day PO divided BID. After 12 weeks, DDS reduced from a mean of 2.0 (range 1.1-2.3) to 1.0 (0.8-1.3; mean decrease 46%, p=0.02). Physician global activity visual analog scale (VAS) decreased from a mean of 5.2 (3.5-7.7) to 3.9 (3.0-6.0; 24% decrease, p=0.04). In the 2/4 with baseline weakness, MMT8 increased from 108 to 134 and 116 to 141 (mean improvement 23%). Extramuscular global VAS decreased from mean 5.2 (3.0-7.7) to 3.6 (1.0-4.9; mean 34% decrease, p=0.047). CDASI activity score reduced from mean 42 (26-53) to 27 (18-46; mean improvement 35%, p=0.04). One individual reduced prednisone dose from 21 to16.5 mg/day; 3 patients’ doses remained unchanged. Baricitinib was generally well tolerated. One patient had an increase in plasma BK viral titer; the other 3 patients were negative for BK virus in blood and urine. No adverse events required holding/discontinuing baricitinib.

Conclusions: Preliminary data on the use of barcitinib (JAK 1/2 inhibitor) in 4 refractory JDM patients are encouraging, showing improvement in symptom DDS as well as in validated measures of rash (4/4) and strength (2/2 with baseline weakness), and other CSMs. Baricitinib was generally well tolerated and further evaluation in this population should be considered. Disclosures: Baricitinib provided by Eli Lilly and Company, expanded access program sponsor.

Other support: Intramural Research Program of NIH, NIAMS, NIEHS, CC.

### P44 Intravenous iMMunoglobulins as early treatment in newly DIAgnosed idiopathic inflammaTory myopathiEs (IMMEDIATE): a phase-2 open-label pilot study.

#### Johan Lim, Myrthe Jaring, Amin Amin, Yigal Pinto, Nils Planken, Matthijs Boekholdt, Anneke van der Kooi

##### University of Amsterdam; Amsterdam, Netherlands

###### **Correspondence:** Johan Lim

Background: Cardiac involvement in patients with myositis - a group of rare auto-immune disorders – causes myositis-related mortality in approximately 5%, yet standardized and evidence-based screening strategies lack. Therefore, we explored different diagnostic modalities to screen for cardiac involvement in new-onset myositis, and potententially reversible peri/myocarditis in particular.

Methods: Adult patients with biopsy-proven myositis from April 2017 to April 2018 were consecutively included at three neuromuscular referral centers in The Netherlands. All patients were referred to a cardiologist for standardized clinical evaluation and cardiac investigations at diagnosis which consisted of: cardiac enzymes (cardiac troponin T, cardiac troponin I, and N-terminal pro b-type natriuretic peptide, electrocardiography (ECG), cardiac ultrasound (US; consisting of standard and strain investigations), and cardiac Magnetic Resonance Imaging (MRI; amongst others consisting of late gadolinium enhancement, T1 mapping, and extracellular volume investigations). Peri/myocarditis and/or other cardiac abnormalities were defined as cardiac involvement at the discretion of the treating physician.

Results: We included 25 patients: 11 with dermatomyositis, seven with immune-mediated necrotizing myopathy, four with non-specific myositis/overlap myositis, and three with anti-synthetase syndrome. Median age at onset was 56 years (range: 25-80 years). There was a female predominance of patients (F:M ratio of 2:1). The median disease duration at diagnosis was five months (range: 1-25 months). Three of 25 (12%), three of 23 (14%), six of 23 (26%), and 11 of 13 (85%) patients were already treated with immunosuppressants during evaluation of cardiac enzymes, ECG, US and MRI, respectively. Eleven patients (44%) had complaints (dyspnea, palpitations, peripheral edema, atypical chest pain) of possible cardiac origin at time of diagnosis. One of six patients (17%) with clinical relevant abnormalities on ancillary investigations had none of the abovementioned complaints. We found four cases (16%) of peri/myocarditis confirmed by cardiac MRI and one suspected case of peri/myocarditis as assessed by the treating cardiologist. Other signs of cardiac involvement were arrhythmias in two patients; no cases of cardiac failure or pulmonary hypertension were found. We found that peri/myocarditis was reversible on cardiac MRI after 9 weeks of intravenous immunoglobulin (IVIg) monotherapy in at least one patient.

Conclusion: Early multimodality screening for potentially reversible cardiac involvement in myositis has added value as illustrated by the number of patients with (suspected) peri/myocarditis (16%) and the patient whose peri/myocarditis improved IVIg therapy. A subsequent RCT on an alternative treatment regimen for cardiac involvement in myositis on clinical outcome is needed.

### P45 Preliminary prospective validation of the juvenile dermatomyositis activity index (JDMAI)

#### Silvia Rosina^1^, Giulia Camilla Varnier^2^, Alessandro Consolaro^1^, Pieter van Dijkhuizen^1^, Kiran Nistala^2^, Nicola Ruperto^1^, Angela Pistorio^1^, Clarissa Pilkington^1^, Angelo Ravelli^1^

##### ^1^Istituto Giannina Gaslini, Genova, Italy; ^2^Great Ormond Street Hospital, London, UK

###### **Correspondence:** Silvia Rosina

Objectives: To test prospectively the validity of the first composite disease activity score for juvenile dermatomyositis (JDM), named JDMAI, in a sample of patients seen in daily practice. The 6 preliminary versions of the tool performed similarly in previous validation analyses.

Methods: 57 JDM patients seen consecutively at 2 tertiary-care paediatric rheumatology centers, either at disease onset or at a follow-up visit, a median of 2 years after disease onset, were enrolled. Each patient received a retrospective assessment and a cross-sectional evaluation at study entry. 30 patients also underwent a second assessment after a median of 3.6 months. Physician-centered evaluations included assessment of muscle, skin and overall disease activity on a visual analogue scale (VAS) or through the Disease Activity Score (DAS), rating of disease state and course and measurement of disease damage with the Myositis Damage Index (MDI). Laboratory data comprised muscle enzymes and ESR. Parent-reported outcomes were collected through the administration of the Juvenile Dermatomyositis Multidimensional Assessment Report (JDMAR). Validation analyses included assessment of construct validity, internal consistency (with Cronbach’s α), discriminant ability, and responsiveness to change in disease state over time (with standardized response mean, SRM).

Results: All JDMAI versions showed strong (r > 0.7) correlations with muscle disease activity VAS (0.83-0.84), muscle section of DAS (0.78-0.80), total DAS (0.86-0.89), pain VAS (0.71-0.74), and fatigue VAS (0.88-0.89). The correlations of the different JDMAI versions with the skin index not contained in the specific version (skin disease activity VAS or skin section of DAS) were strong (0.72-0.76). As expected, JDMAI correlations were moderate (r=0.4-0.7) or low (r < 0.4) with laboratory parameters and with the MDI (0.04-0.52). The SRM was greater in patients judged as improved by the physician or the parent (0.83-1.15) than in those judged as not improved (0.02-0.51). Cronbach’s α was very high (0.884-0.900) for all JDMAI versions. All JDMAI versions discriminated strongly between patients classified in different disease activity states by the physician (p < 0.0001) or by the parent (p=0.011-0.031), and between patients whose parents were satisfied or not with their children’s disease status (p ≤ 0.0001).

Conclusion: Validation analyses in this prospective patient sample confirmed that the JDMAI possesses good measurement properties and is a suitable and reliable tool for the assessment of disease activity in children with JDM both in clinical practice and research. Importantly, the new tool revealed a strong capacity to capture the improvement of disease activity over time.

### P46 Nodular Regenerative Hyperplasia of the Liver in Juvenile Dermatomyositis

#### Rita Volochayev, David E. Kleiner, Prateek Gowda, Anusha Vittal, Theo Heller, Lisa G. Rider

##### National Institutes of Health, Bethesda, USA

###### **Correspondence:** Rita Volochayev

Objectives: To present two cases of Nodular Regenerative Hyperplasia (NRH) of the liver associated with Juvenile Dermatomyositis (JDM).

Methods: We reviewed liver biopsies of four JDM patients from a cohort of 159 patients, and two had changes consistent with NRH. A PubMed search revealed 2 patients with NRH in adult Polymyositis and Dermatomyositis, but no previously reported cases in JDM.

Results: Case 1. Nine-year-old male with anti-MJ autoantibodies, diagnosed with JDM at two years of age, with chronically active disease with severe weakness and rashes, severe calcinosis and dysphagia, dysphonia, colitis with diarrhea and weight loss, hepatosplenomegaly, and portal hypertension. Prior immunosuppressive medications, often used in combination, included prednisone, mycophenolate mofetil, azathioprine, methotrexate, cyclophosphamide, hydroxychloroquine, etanercept, infliximab, abatacept, and tocilizumab. Blood laboratories were notable for development of elevated liver enzymes (ALT 84 U/L (5-30), AST 83 U/L (0-40), GGT 72 U/L (3-22), Aldolase 11.6 U/L (1.0-8.0); normal alkaline phosphatase and total bilirubin; and decreased Platelet Count 127K (206-369K). Hepatitis, HIV and Epstein Barr testing was negative for active infection. Abdominal ultrasound showed splenomegaly and portal hypertension. Liver biopsy showed vaguely nodular architecture that on reticulin stain revealed regenerative nodules bounded by atrophic liver plates, with mild sinusoidal fibrosis and congestion, diagnostic of NRH. Case 2. 18-year-old Asian female with anti-MDA5 autoantibodies, diagnosed with JDM at age 16, who had ulcerative skin lesions, Raynaud’s, arthritis, and interstitial lung disease with pulmonary hypertension, moderate to severe weakness, calcinosis and chronically active disease. Medications included prednisone, methotrexate, abatacept, cyclophosphamide, tofacitinib, and sildenafil. Laboratories were remarkable for elevated alkaline phosphatase of 133 U/L (35-105), ALT 34 U/L (0-33), AST 43 U/L (0-32), GGT 89 U/L (0-39), and progressively decreasing platelet count of 154K (173-369K), but normal Aldolase at 4.9 U/L (1.0-8.0). Hepatitis, HIV and Epstein Barr testing was negative for active infection. Ultrasound of liver, abdomen and spleen was normal. Elastography was 1.63 m/s and FibroScan 11 kPa, in a range indicative of fibrosis. Liver biopsy showed nodular parenchyma without fibrosis, that on reticulin stain also demonstrated characteristic regenerative nodules bounded by atrophic liver plates.

Conclusion: We have identified 2 cases of NRH in patients with severe JDM via liver biopsy, which is currently the only way to diagnose NRH. Both patients received Azathioprine, which has been associated with NRH, and had severe, chronically active disease. These patients need to be monitored for complications of NRH, including development of portal hypertension and varices.

### P47 Preliminary Validation of Rectus Femoris Muscle Ultrasound in Juvenile Dermatomyositis

#### Erica McBride^1^, Gulnara Mamyrova^1^, Michael Harris-Love^2^, Ahalya Premkumar^2^, Deloris Koziol^2^, Jianhua Yao^2^, Lawrence Yao^2^, Joseph Shrader^2^, Rodolfo Curiel^1^, Lisa Rider^2^

##### ^1^George Washington University, Alexandria, USA; ^2^National Institutes of Health, Bethesda, USA

###### **Correspondence:** Erica McBride

Objectives: The goal of this study was to examine muscle ultrasound (MUS) parameters in Juvenile Dermatomyositis (JDM), to assess correlation with disease activity and damage, and to compare the sensitivity and specificity of MUS to magnetic resonance imaging (MRI).

Methods: MUS of the right mid-rectus femoris (RF) using the Acuson Sequoia was performed in 21 JDM patients (PTS) meeting probable or definite Bohan and Peter criteria and 28 age-, gender- and race-matched healthy controls (CON). Quantitative MUS parameters of patients were correlated (Spearman rank) with JDM disease activity and damage assessments and semi-quantitative thigh MRI short tau inversion recovery (STIR) and T1 scores. Echogenicity and Doppler was quantified using imaging processing software. The area under the receiver operating characteristic (ROC) was used to compare sensitivity and specificity of MUS to MRI.

Results: The median age at diagnosis of JDM PTS was 12 years, 67% were female, 81% were Caucasian. The median age of CON was 14.1 years, with similar gender and races as PTS. Median MD Global Disease Activity was 3.1 cm and MD Global Disease Damage was 1.9 cm (10 cm VAS). There was a significant increase in RF echogenicity [median 47.8 in PTS vs. 38.5 CON, p=0.002], in JDM PTS compared with CON. Mean echogenicity correlated with MD Global Activity (rs=0.46, p=0.04) and inversely with MMT (rs -0.54, p ≤ 0.01). Resting and contracted RF area inversely correlated with MD Global Activity (rs -0.46-; -0.56, p=0.04) and positively with proximal MMT (rs=0.47 -0.79, p ≤ 0.03). Contracted RF area also correlated with CMAS (rs=0.80, p < 0.0001) and serum creatinine kinase (CK) (rs=0.47, p=0.03). Several MUS parameters also correlated with isometric dynamometry: notably, QMT Knee Extension correlated with RF area (rs=0.76, p < 0.0001) and RF contracted area (rs=0.86, p < 0.0001). MUS echogenicity moderately correlated with both STIR (rs=0.43 p=0.058) and T1 MRI sequences (rs=0.55, p=0.01). T1 MRI correlated with the difference in RF area between the contracted and resting states (rs=0.62, p=0.004). The area under the curve by ROC analysis of MUS echogenicity vs. STIR MRI was 0.75, and using an average of STIR and T1 MRI, improved to 0.87.

Conclusions: Several MUS parameters of RF differed between JDM vs. CON. MUS also had moderate to strong construct validity with JDM disease activity, strength and MRI measures. MUS echogenicity has strong sensitivity and specificity compared to MRI, and is a promising imaging modality for JDM patients.

### P48 Circulating microparticles as potential markers of disease progression and vascular dysfunction in Juvenile Dermatomyositis

#### Charalampia Papadopoulou^1^, Ying Hong^1^, Petra Krol^1^, Yiannis Ioannou^2^, Clarissa Pilkington^1^, Marietta Charakida^3^, Lucy R. Wedderburn^1^, Paul Brogan^1^, Despina Eleftheriou^1^

##### ^1^Great Ormond Street Institute of Child Health, London, UK; ^2^Arthritis Research UK, Chesterfield, UK; ^3^University College London, London, UL

###### **Correspondence:** Charalampia Papadopoulou

Background: JDM is an autoimmune disease characterised by proximal muscle weakness and pathognomonic skin rashes. Vasculopathy plays a central role into disease pathogenesis. There is, however, a lack of appropriate biomarkers to detect and monitor endothelial injury and persistently high disease activity relating to the vasculopathy of JDM. Microparticles (MP) are membrane vesicles released during cell activation or apoptosis that can allow tracking of endothelial injury and cellular activation. MP also have important procoagulant properties.

Objectives: To investigate the MP profiles associated with endothelial activation and cellular activation in children with JDM, and to examine whether this approach could serve as a biomarker for monitoring disease activity relating to the vasculopathy of JDM. Methods: 90 patients recruited to the UK JDM Cohort & Biomarker Study were included; median age 10.21 (range 6.68-13.40) years with median disease duration of 1.63 (0.28-4.66) years. Inactive disease was defined as per modified PRINTO criteria: no skin rashes, CK ≤ 150, CMAS ≥ 48, MMT8 ≥ 78, Physician ‘s global assessment (PhyGLOVAS) ≤ 0.2 on a visual analogue scale. MPs were identified with multicolour flow cytometry, respectively. Plasma thrombin generation was determined using a fluorogenic assay. Results are expressed as median and range.

Results: Total circulating MP counts differed significantly between those with active JDM, 301 (186-584) x103/ml and those with inactive disease, 81 (34-191) x103/ml, p < 0.0001; and controls 44 (15-249) x103/ml, p < 0.0001. These circulating MPs were predominantly of platelet and endothelial origin. Enhanced plasma mediated thrombin generation likely due to MP presence was demonstrated in active compared to inactive JDM and controls (p=0.001) while TF+CD14+MP counts strongly associated with the endogenous thrombin potential (r=0.21, p=0.01) suggesting for the first time that MP could have an important prothrombotic role in the pathogenesis of JDM vasculopathy. Total Annexin V+ MP counts were strongly correlated with circulating endothelial cells (r=0.42, p < 0.0001) and strongly correlated with the PhyGLOVAS (r=0.43, p=0.0002)

Conclusion: Circulating MP profiles may reflect distinct disease activity status in patients with JDM. These novel biomarkers of vascular pathology, platelet activation and thrombotic propensity can track disease trajectories in JDM and predict high risk groups for poorer disease outcomes.

### P49 Efficacy of Adrenocorticotropic Hormone Gel (Acthar) in Refractory Juvenile Dermatomyositis

#### Aarat Patel^1^, Thomas Michael Pender^2^, Margalit Rosenkranz^3^

##### ^1^University of Virginia, Charlottesville, USA; ^2^Eastern Virginia Medical School, Norfolk, USA; ^3^Children's Hospital of Pittsburgh, Pittsburgh, USA

###### **Correspondence:** Aarat Patel

Patient 1: 12 year old obese male presented with proximal muscle weakness, characteristic skin rash, elevated muscle enzymes (Aldolase 57.2U/L, LDH 917U/L, CK 12,480U/L). Prednisone caused anxiety. Keeping obesity in mind, acthar 80U twice weekly was used (3 months) with IVIG (6 months) and methotrexate. After 1 month of acthar, all enzymes had normalized. No weight gain or other steroidogenic AE’s occurred. Methotrexate (18 months) was weaned with remission maintained.

Patient 2: 15 year old female presented with proximal muscle weakness, characteristic skin rash, elevated muscle enzymes and inflammatory arthritis. MRI was positive for myositis. Patient started prednisone, methotrexate and plaquenil and flared when prednisone was weaned. IVIG (6 months) was initiated and restarting prednisone caused acne, cushingoid appearance and striae. Acthar 80units q5days (6 months) was used as a steroid-sparing agent. Methotrexate and plaquenil have kept the patient in remission.

Patient 3: 5-year-old female presented with proximal muscle weakness, characteristic skin rash, elevated muscle enzymes, lymphadenopathy and skin ulcerations. There was minimal response to methotrexate and plaquenil; no response to azathioprine; good response to IVIG however severe headaches. Chronic prednisone use caused gastritis, cushingoid appearance and striae. Acthar 80U twice weekly (4 months) with methotrexate did not help wean off of IVIG and prednisone. Finally she responded to rituximab every 6 months with methotrexate. Acthar was not effective.

Patient 4: 20 year female presented with proximal muscle weakness, characteristic skin rash, elevated muscle enzymes, calcinosis, inflammatory arthritis, Raynaud’s, mechanics hands, difficulty swallowing, SOB. CT scan showed ILD changes. Labs revealed elevated muscle enzymes (Aldolase 54U/L, and CK 2,309U/L). She was initially treated with prednisone, IVIG and azathioprine. Azathioprine was not effective. Prednisone caused anxiety and worsening depression. Acthar 80U twice weekly (11 months) was used while IVIG was tapered and methotrexate was started; she achieved remission in all disease manifestations. Discussion: Acthar is thought to play an immunomodulatory role through engagement of the melanocortin receptors in the immune system.

All patients in this case series were refractory to first and second line treatments and/or had AE’s to corticosteroids. Acthar was used as a bridge therapy and steroid sparing agent while patients were experiencing ongoing disease. Improvement was noted in three of four patients. AE’s that were seen with steroids were absent with acthar. Our results are similar to other retrospective case series of patients with refractory myositis however this would be the first reported data in JDM.

### P50 Long-term Outcome of Children with Juvenile Dermatomyositis: a single-centre study from North India

#### Avinash Sharma^1^, Anju Gupta^2^, Amit Rawat^2^, Deepti Suri^2^, Surjit Singh^2^

##### ^1^Dr. Rajendra Prasad Government Medical College, Himachal Pradesh, India; ^2^Post Graduate Institute of Medical Education & Research, Chandigrah, India

###### **Correspondence:** Avinash Sharma

Background: Juvenile dermatomyositis (JDM) is a rare inflammatory myopathy seen in children. There have been few studies on long-term outcome in children with JDM using validated outcome measures. We undertook this study to assess the long-term outcome of JDM using validated measures of outcome.

Methods: Cross-sectional observational study. All children diagnosed to have JDM for more than 2 years and registered in Pediatric Rheumatology Clinic at Post Graduate Institute of Medical Education and Research, Chandigarh, India, were deemed eligible for recruitment. Study was conducted from January 1, 2015 to June 30, 2016. Those who were not on regular follow-up were called for assessment. Assessment was done by a single observer using CMAS, MMT8, MDAAT, aCAT, MDI and CHAQ.

Results: Thirty-five children were enrolled in this study. Twenty-two (62.9%) were on regular follow-up. Mean age was 13.9 years (range 4-29). Mean age at diagnosis was 7.51 ± 3.56 years with median time interval between onset of symptoms and diagnosis being 5 months. Mean duration of disease at the time of enrolment was 7.18 years. Disease course was monocyclic in 24 (68.6 %). Muscle strength was normal in 71.4% with MMT8 score of 80 and normal CMAS. Severe involvement defined as MMT8 score below 64 was seen in 8.6%. Cutaneous activity was determined by aCAT with 14 (40%) children having a score of ≥ 1 suggesting some form of cutaneous activity. Based on MYOACT, 31.4% children in our study had evidence of disease activity at the time of assessment with skin being the commonest organ system involved in 28.6% followed by muscles in 22.9%. Twenty-one (60%) children had some form of cutaneous damage. Calcinosis was seen in 12 (34.3%) and lipodystrophy in 8 (22.9%). Twenty-four (68.6%) subjects had an MDI score of ≥ 1 at the time of evaluation suggesting damage in at least one organ system. Most commonly affected organs were cutaneous, endocrine and muscles in 20 (57.1%), 12 (34.3%) and 9 (25.7%) subjects respectively. Nine (25.7%) subjects in our study had some form of a physical dysfunction suggested by a CHAQ score above 0 at the time of cross-sectional assessment.

Conclusion: The highlight of this study is use of validated outcome measures for evaluation of long-term outcomes. After mean disease duration of 7.18 years, one-third subjects had evidence of disease activity with almost one-tenth having moderate to severe activity. About 2/3rd had damage in at least one organ system.

### P51 Presentation Features and Misdiagnosis in anti-MDA5 Autoantibody (Ab) Associated Juvenile Dermatomyositis (JDM)

#### Gulnara Mamyrova^1^, Lan Wu^2^, Adam M. Huber^3^, Ira N. Targoff^4^, Rodolfo V. Curiel^1^, Frederick W. Miller^2^, Lisa G. Rider^2^

##### ^1^George Washington University, Washington, USA; ^2^National Institute of Environmental Health Sciences, Bethesda, USA; ^3^IWK Health Centre and Dalhousie University, Halifax, Canada; ^4^University of Oklahoma Health Sciences, Oklahoma City, USA

###### **Correspondence:** Gulnara Mamyrova

Background/Purpose: To assess presentation features of anti-MDA5 (melanoma-differentiation associated gene 5) Ab associated JDM.

Methods: Physician completed questionnaire about presentation features for 35 MDA5 Ab+ JDM patients meeting probable or definite Bohan and Peter criteria and compared to those of 132 patients with anti-TIF1, 84 anti-NXP2, 15 anti-ARS (aminoacyl-tRNA synthetase), 12 anti-Mi2 Abs, and 144 MSA/MAA negative patients. Myositis Abs were tested by validated immunoprecipitation (IP) and IP-immunoblotting.

Results: Median delay to diagnosis in MDA5 (4 [1-8] months) was shorter compared to anti-ARS (7.5 [3-13.2] months, p=0.033). The most frequent first symptoms in anti-MDA5 Ab+ were cutaneous (37%), constitutional (14%), muscular and skeletal (8.6% each). Rash as a first symptom was less frequent in anti-MDA5 Ab+ compared to anti-TIF1 (75%, p=0.0001). Weakness as a first symptom was less frequent in anti-MDA5 (8.6%) compared to anti-NXP2 (48%, p=0.0001), ARS (32%, p=0.05), Mi2 Ab+ (25%, p=0.018), and Ab negative (35%, p=0.0012). Skeletal symptoms were less frequently first compared to anti-ARS (8.6% vs. 32%, p=0.05). 28% of anti-MDA5 had a combination of cutaneous, constitutional, and skeletal symptoms initially; 26% of anti-MDA5 Ab+ presented with rash and weakness simultaneously. Rash before weakness was less frequent in anti-MDA5 Ab+ compared to anti-TIF1 (49% vs. 72%, p=0.014). Gottron’s papules (31%) were most often first rash followed by both Gottron’s and heliotrope (14%), and heliotrope alone (11%). In 11.6% of anti-MDA5 patients, first rash was a combination of both Gottron’s and heliotrope with other rashes (malar, V-sign, skin ulcerations). Gottron’s combined with ulceration, malar, or periungual erythema were first rashes in 8.7%. Other initial rashes included malar and/or other erythema (5.7% each), or mucus membrane lesions, maculopapular and vasculopathic rashes (2.9% each). Heliotrope was less frequent first rash in anti-MDA5 Ab compared to anti-NXP2 (11% vs. 34%, p=0.013) and anti-ARS (47%, p=0.01). First weakness was proximal in 80% of anti-MDA5 patients, both proximal and distal in 8.6%, and distal in 11%. Early distal weakness was more frequent in anti-MDA5 compared to anti-TIF1 Ab+ (11% vs. 0.8%, p=0.007), anti-NXP2 (0%, p=0.006), and MSA negative patients (1.8%, p=0.019). 23.5% of anti-MDA5 Ab patients had one and 14.7% two misdiagnoses, including infections (17.6%), skin diseases (14.7%), other autoimmune (5.9%), cardiac and psychologic disorders (2.9% each).

Conclusion: In anti-MDA5 Ab+ JDM, cutaneous, constitutional and skeletal symptoms are observed most frequently at illness onset, and patients present most often with rash before weakness. Illness presentations of anti-MDA5 Abs differ from other MSAs.

### P52 Autoantibody Profile of Children with Juvenile Dermatomyositis from a single-centre in North India

#### Avinash Sharma^1^, Anju Gupta, Amit Rawat, Deepti Suri, Surjit Singh

##### ^1^Dr. Rajendra Prasad Government Medical College, Himachal Pradesh, India; ^2^Post Graduate Institute of Medical Education & Research, Chandigrah, India

###### **Correspondence:** Avinash Sharma

Background: Children with JDM can have autoantibodies in their sera like other autoimmune diseases. Over the last few years, few novel Myositis Specific Antibodies (MSA) have been identified, like anti-p155/140 (Anti-TIF1γ), anti-p140 (anti NXP2), CADM-140 (anti-MDA5), anti-SAE and anti 200/100 (anti HMG-CoA reductase). Some phenotypical associations have been described with these autoantibodies. Few of these autoantibodies have not been studied in children. We undertook this study to look for autoantibodies including anti p200/100 (anti HMG CoA reductase) which has not been evaluated in children and anti p-140 (anti NXP2) which has been shown to have correlation with calcinosis in children with JDM.

Methods: Cross-sectional study. All children diagnosed to have JDM for more than 2 years and registered in Pediatric Rheumatology Clinic at PGIMER, Chandigarh, India, were deemed eligible for recruitment. Study period: January 1, 2015 to June 30, 2016. Autoantibody testing for MSA and MAA was done for enrolled children. Immunodot was done to detect IgG antibodies against Jo1, threonyl-tRNA synthetase (PL7), alanyl-tRNA synthetase (PL12), glycyl-tRNA synthetase (EJ), Signal Recognition Particle (SRP), Mi-2, MDA-5, TIF-1γ, Ku, PMScl 100, Scl 70 and SSA/Ro52. Evaluation for anti-p-140 or NXP2 and anti-200/100 or 3HMG CoA reductase was done using ELISA.

Results: Antinuclear antibody (ANA) testing was done in 34 patients. Fourteen (40%) tested positive. Anti-SRP antibodies were present in 4 (11.4%) children, anti-MDA5 in 3 (8.6%), anti-Mi2 in 1 (2.9%) and 1 patient tested positive for anti-SSA/Ro52 antibodies. All 4 children with anti-SRP were girls, had polycyclic course and 2 of them had calcinosis and signs of disease activity at the time of evaluation. Patients with anti-MDA5 had predominant skin involvement, less severe muscle disease and followed monocyclic course. Two of them had arthritis/arthralgia at the time of presentation. The only patient in our study with anti-Mi2 had normal muscle strength/endurance at the time of assessment. None had anti-synthetase antibodies (anti-Jo1, anti-PL-7, anti-PL-12, anti-EJ), anti-ku or anti-Scl70. None of the subjects tested positive for anti-NXP2 or anti-HMG CoA.

Conclusion: Prevalence of autoantibodies in children with JDM in our study is similar to what has been described previously. Type of autoantibodies, though, is not similar. This may be due to ethnic differences of the population. Autoantibodies were tested in children while they were on treatment. This may have resulted in lower positivity. Evaluation of autoantibody profile at the time of diagnosis may assist in predicting the course of disease and response to treatment.

### P53 Myositis-specific antibodies in juvenile dermatomyositis and their associated clinical phenotypes in a German cohort

#### Svea Horn^1^, Claudia Sengler^1^, Nadine Grösch^1^, Jens Klotsche^1^, Johannes-Peter Haas^2^, Claas Hinze^3^, Gerd Horneff^4^, Tilmann Kallinich^5^, Frank Weller-Heinemann^6^, Kirsten Minden^1^

##### ^1^German Rheumatism Research Center, Berlin, Germany; ^2^German Center for Pediatric and Adolescent Rheumatology Garmisch-Partenkirchen, Garmisch-Partenkirschen, Gemany; ^3^Universität Münster, Münster, Germany; ^4^Asklepios Klinik St. Augustin, Sankt Augustin, Germany; ^5^Charité - Universitätsmedizin Berlin, Berlin, Germany; ^6^Prof.-Hess-Kinderklinik Bremen, Bremen, Germany

###### **Correspondence:** Svea Horn

Objectives: Juvenile dermatomyositis (JDM) is a heterogenous disease – next to weakness of the proximal muscles and typical skin lesions as leading symptoms there can be involvement of the joints, lungs, heart, gastrointestinal tract or mucosa. Myositis-specific antibodies (MSA) may help to distinguish clinically distinct phenotypes of JDM with possibly different treatment response and outcome.

Methods: Cross-sectional data of patients with JDM documented in the national pediatric rheumatology database (NPRD) between 2014 and 2016 were analyzed. In addition, a retrospective chart review was conducted for all patients with MSA determination to further specify the phenotype and patient`s outcome. MSA were determined centrally by a commercial multiplex array (EUROLINE AutoImmune Inflammatory Myopathies).

Results: MSA testing was performed on 88 patients with JDM (73% female). Mean age at disease onset was 7.6 years (SD 3.6) with a mean age at documentation of 12.2 years (SD 3.8). MSA could be detected in 42% of patients with the following frequencies: anti-NXP2 16%, anti-TIF1ƴ 14%, anti-MDA5 6%, anti-Mi2 3%, anti-synthetase-antibodies 3% (anti-Jo1, anti-PL-12, anti-OJ, anti-EJ). Anti-SAE and anti-SRP that are also part of the test assay were not found in our patients. The most common clinical feature was dysphagia in patients with anti-NXP2, calcinosis in patients with anti-TIF1ƴ, lung involvement in patients with anti-synthetase antibodies, and muscle weakness in patients with anti-Mi2. Patients with anti-MDA5 detection were characterized by frequent interstitial lung involvement, mucosal ulcers, fever and polyarticular arthritis of small joints. The extent of muscle weakness based on the Childhood Myositis Assessment Score (CMAS) was significantly associated with increased levels of creatine kinase. At last consultation (mean disease duration 4.7 years), 32% and 14% received oral glucocorticoids < 0.2 mg/kg or ≥ 0.2 mg/kg body weight, respectively, 52% were treated with methotrexate (MTX), 27% had received intravenous immunoglobulins within the past 12 months. Only 6 patients received biologics, 5 rituximab and 1 etanercept. Physician`s global assessment of disease activity (PGA) was equal or less than one in 65% of patients at last consultation, 22% of those were off therapy.

Conclusion: Myositis-specific antibodies could be found in almost half of the patients examined by a commercial multiplex array. Clinical phenotypes were associated with specific MSA. Outcome after 5 years was good in 2/3 of patients, with still about half of the patients receiving MTX and/or glucocorticoid therapy.

### P54 Genetic Differences in the HLA Region Between Adult- and Juvenile-Onset IIM Patients with Anti-TIF1 Autoantibodies

#### Simon Rothwell^1^, Ingrid E. Lundberg^2^, Frederick W. Miller^3^, Lisa G. Rider^3^, Lucy R. Wedderburn^4^, Neil J. McHugh^5^, Hector Chinoy^1^, Janine A. Lamb^1^

##### ^1^University of Manchester, Manchester, UK; ^2^Karolinska Institute and Karolinska University Hospital, Stockholm, Sweden; ^3^National Institutes of Health, Bethesda, USA; ^4^Great Ormond Street Institute of Child Health, London, UK; ^5^University of Bath, Bath, UK

###### **Correspondence:** Simon Rothwell

Objectives: Idiopathic inflammatory myopathies (IIM) are a spectrum of rare autoimmune diseases characterised clinically by muscle weakness and heterogeneous systemic organ involvement. The strongest genetic risk is thought to be within the major histocompatibility complex (MHC) in autoantibody defined subgroups. Clinical features of disease may differ between adult- and juvenile-onset disease with the same autoantibody. For example, there is association of anti-TIF1 autoantibodies and cancer in adult-onset disease that is not present in juvenile-onset disease. We investigated potential differences in HLA associations between adult- and juvenile-onset myositis in autoantibody subgroups.

Methods: We used SNP2HLA to impute classical HLA alleles and amino acids from Immunochip genotyping data generated through the Myositis Genetics Consortium (MYOGEN). Genotypes from an additional 19 UK juvenile-onset patients were included from the Juvenile Dermatomyositis Cohort Biomarker Study and Repository. Autoantibodies were detected using immunoprecipitation (IP), IP-immunoblot, lineblot and/or ELISA. We stratified the autoantibody defined subgroup by age and compared the frequency of HLA variants against healthy controls.

Results: We report a strong association between HLA-DQB1*02 and anti-TIF1 (n=197) autoantibodies, and identify genetic differences at this locus between adult-(n=91) and juvenile-onset (n=106) patients with this autoantibody. In adult-onset IIM, the strongest HLA association was with HLA-DQB1*02:02 (p=2.63x10-5). This allele was not significantly associated in juvenile patients, whereas a strong association was found with HLA-DRB1*03:01 (p=3.51x10-6), which is present on the 8.1 ancestral haplotype. An additional analysis was restricted to UK anti-TIF1 patients serotyped using IP in the same centre at the University of Bath, including adult- (n=40) and juvenile-onset patients (n=48) with anti-TIF1 autoantibodies. We replicated observations from the combined cohort showing this difference in association was not due to batch effects or method of autoantibody detection. For anti-Jo-1, anti-PM/Scl, anti-Mi-2 and anti-cN1A the trends for HLA association were in the same direction in juvenile-onset IIM as in the combined cohort, but did not reach statistical significance due to small sample numbers.

Conclusions: We show for the first time that there are genetic differences between adult and juvenile-onset patients with anti-TIF1 autoantibodies. These genetic differences may suggest a different aetiology, and may provide insight into specific mechanisms of disease.

### P55 Using the internationally agreed-upon Consensus Dataset for Juvenile Dermatomyositis (JDM) in clinical practice and research

#### Liza McCann^1^; Michael W. Beresford^2^; Clarissa A. Pilkington^3^; Adam Huber^4^; Angelo Ravelli^5^; Claire T. Deakin^6^; Hector Chinoy^7^; Lucy R. Wedderburn^6^

##### ^1^Alder Hey Children's NHS Foundation Trust, Liverpool, UK; ^2^University of Liverpool, Liverpool, UK; ^3^Great Ormond Street Hospital for Children, London, UK; ^4^IWK Health Centre and Dalhousie University, Halifax, Canada; ^5^Università degli Studi di Genova and Istituto Giannina Gaslini Pediatria IIm, Genova, Italy; ^6^University College London, London, UK; ^7^University of Manchester, Manchester, UK

###### **Correspondence:** Liza McCann

Objectives: Through a robust international process, using the infrastructure of paediatric rheumatology and myositis organisations and patient/parent groups (including IMACS, CARRA, PReS, JDRG, PRINTO, Euromyositis, COMET, OMERACT, Myositis UK, Cure JM), to develop a JDM Consensus Dataset that can capture disease activity and damage over time, be used in clinical practice and research, and that is freely available.

Methods: Exploiting Delphi methodology, two web-based questionnaires were distributed to healthcare professionals caring for JDM identified through e-mail distribution lists of international paediatric rheumatology and myositis research groups (181 replies). A separate questionnaire was sent to parents of children with JDM and patients with JDM identified through established research networks and patient/parent support groups (301 replies). Results informed a face-to-face consensus meeting of 18 voting experts from paediatric and adult healthcare with representation from rheumatology, neurology, dermatology and physiotherapy, tasked with defining contents of the dataset.

Results: A JDM Consensus Dataset (123 items) has been formulated into 3 sections, with accompanying glossaries of definitions. Form A, collected at baseline only, contains demographic and diagnostic data. Form B includes disease activity measures, collected at every visit. Damage items, contained within form C, are collected at baseline and annually. After testing in practice, the dataset has been incorporated into two large research registries: the JDM component of the Childhood Arthritis and Rheumatology Research Alliance (CARRA) and JDM Euromyositis. Work has started to migrate the dataset into the UK Juvenile Dermatomyositis Cohort and Biomarker Study (JDCBS), which currently holds data on >580 subjects, with collaboration allowing the greatest number of children and young people with JDM to be analysed. Data captured from patient/parent questionnaires can be used to help inform the development of Patient Reported Outcome Measures relevant and specific to children and young people.

Conclusions: International collaboration for JDM is essential to increase patient numbers to help understand how risk factors interplay with age or environment and influence long-term outcome. Use of the JDM Consensus Dataset allows standardisation of data collection to aid data sharing and comparison between groups. Current barriers to data sharing between registries include legal and ethical regulations and the fact that data collection on juvenile-onset myositis within paediatric registries often stops when young people transfer to adult services. Our group is working to resolve these issues and test the dataset within hypothesis driven research projects. We welcome collaboration with other groups and use of the JDM Consensus Dataset.

### P56 Physical Function Trajectories in Children with Juvenile Myositis

#### Kaveh Ardalan^1^, Elizabeth Gray^1^, Julia Lee^1^, Madison Wolfe^2^, Gabrielle Morgan^3^, Lauren Pachman^1^

##### ^1^Northwestern University, St. Evanston, USA; ^2^Creighton University, Omaha, USA; ^3^Ann & Robert H. Lurie Children’s Hospital of Chicago, Chicago, USA

###### **Correspondence:** Kaveh Ardalan

Objectives: Juvenile myositis (JM) is an inflammatory disease that causes muscle weakness, skin rashes, and significant deconditioning. Little is known about long-term resolution of physical disability. We examined trajectories of physical function in JM.

Methods: JM registry data were collected at routine visits (January 2000 to June 2014) to Ann & Robert H. Lurie Children’s Hospital of Chicago and analyzed in this study. Only patients whose baseline visit was < 3 months after initiation of treatment were included. The following variables were extracted: parent-proxy reported Childhood Health Assessment Questionnaire (CHAQ), gender, race, duration of untreated disease, Disease Activity Score (DAS) - muscle/skin domains, Childhood Myositis Assessment Scale (CMAS), muscle enzymes, nailfold capillary end row loops (NFC-ERL), von Willebrand factor antigen (vWFAg), calcinosis (ever experienced), lipodystrophy (ever experienced), TNFalpha-308A allele, and treatments. Descriptive statistics were calculated. Latent trajectory analysis was performed assessing probability of CHAQ > 0 (binary outcome 0 vs > 0). Baseline values for demographic/clinical variables above were compared across identified trajectories (Fisher’s exact; Kruskal-Wallis).

Results: Baseline descriptive statistics for n = 104 included patients with median 13.5 study visits and median follow-up 54 months are similar to published JM cohorts. Three trajectories corresponding to mild (n = 46 [44%]), moderate (n = 32 [31%]), and severe disability (n = 26 [25%]) were identified. Statistically significant differences in baseline variables for mild vs moderate vs severe groups were noted for: CHAQ (0.13 vs 0.88 vs 1.35, p < 0.001), DAS-Muscle (4 vs 6 vs 6, p = 0.001), and CMAS (40 vs 31 vs 34, p = 0.01). Median baseline NFC-ERL differed, with higher counts in the mild group (= 5) vs moderate and severe groups (= 3.83 and 4.14), p = 0.047. Race trended toward significance (p = 0.08), with fewer white patients in the severe group (n = 13 white patients vs n = 26 total patients in severe group).

Conclusion: To our knowledge, this is the first study describing longitudinal trajectories of physical function (distinct from disease activity or fitness) in JM. Baseline physical function (i.e. CHAQ) and muscle weakness (i.e. DAS-Muscle, CMAS) predict long-term physical function trajectory. Baseline vasculopathy (i.e. NFC-ERL) may be less prominent in JM patients following the mild physical function trajectory. There may also be racial disparities in physical function trajectories among youth with JM, though this requires further study.

### P57 Myositis Autoantibodies in Paediatric Idiopathic Inflammatory Myopathies - a Case Series from Singapore

#### Santosi Buvaneswarran, Pei Ling Ooi, Pauline Chan Ng, Kean Lim Lee, Elizabeth Y. Ang

##### Khoo Teck Puat - National University Children's Medical Institute, Singapore, Singapore

###### **Correspondence:** Santosi Buvaneswarran

Objectives: Distinct clinical-serological profiles in patients with Idiopathic Inflammatory Myopathies (IIMs) have been described, with differences even among patients of varying ethnicities. Much less is known about the associations of myositis autoantibodies in children. We describe a series of children with IIMs and aim to correlate their serological profiles with their clinical course.

Methods: Retrospective review of medical records of patients with IIMs attending the Paediatric Rheumatology Clinic. All patients were tested for myositis-specific and myositis-associated antibodies (MSAs, MAAs), together with anti-nuclear antibodies (ANAs).

Results: Ten patients were identified: nine were female; age at diagnosis 3-15 years. Nine had juvenile dermatomyositis (JDMS) and one had necrotizing autoimmune myositis (NAM). Median time to diagnosis was 6 months (1-36 months). At diagnosis, MMT8 testing scores were 90 to 145/150, serum CK 50-9598 IU/ml, LDH 555-2292 IU/ml. Six patients (60%) had positive MSAs and all of these patients had JDMS: two with anti-TIF-1-gamma and one each with anti-Mi2, anti MDA5, anti-EJ and anti Ro52. The two patients with anti-TIF-1-gamma had significant cutaneous involvement, one with ulceration and the other with lipoatrophy. The patient with anti-MDA5 had mild proximal myopathy, marked cutaneous features of JDMS, scleroderma-like changes, oligoarthritis and restrictive lung disease on pulmonary function (PFT) testing with a normal HRCT of the lungs. The patient with anti-Mi2 had minimal skin involvement but marked proximal myopathy and arthritis, with restrictive lung disease on PFT only. The patient with anti-EJ presented with weight loss and had lipoatrophy, calcinosis and polyarthritis with minimal myopathy. The patient with anti-Ro52 had proximal myopathy and characteristic skin manifestations including calcinosis. Three patients were positive for ANA (>1:320): two with JDMS and one with NAM. All patients received steroids and methotrexate, 70% IVIG, 70% hydroxychloroquine, 40% mycophenolate mofetil, 20% tacrolimus, and 20% cyclosporine A. Five patients are in remission at the time of writing, with only one off medication.

Concnlusion: Similar to previous paediatric series, approximately two-thirds of patients tested positive for myositis autoantibodies, with anti-TIF-1-gamma being the most prevalent MSA and associated with significant cutaneous disease. Different to other cohorts, anti-NXP2 was not found in our patients; none of them had clinically significant interstitial lung disease, even with a positive anti-MDA5. We postulate that South East Asian patients have a distinct autoantibody profile from their Caucasian or Japanese counterparts. A regional database would be useful in studying this hypothesis before these antibodies can definitively contribute to clinically significant decision-making.

### P58 MYOSITIS DAMAGE INDEX IN A COHORT OF CHILDREN WITH JUVENILE DERMATOMYOSITIS (JDM) FROM A SINGLE CENTRE IN MUMBAI, INDIA

#### Santan Godad, Pallavi Pimpale Chavan, Raju P. Khubchandani

##### Jaslok Hospital and Research Centre, Mumbai, India

###### **Correspondence:** Pallavi Pimpale Chavan

Introduction: IMACS developed Myositis Damage Index (MDI) separately for pediatrics and adults to document persistent changes in 11 organ systems thought to be related to damage.There is a dearth of studies on damage caused by JDM from less resourced countries.

Objectives:

Primary: Assessing MDI in our cohort of children with JDM

Secondary: Study associations of factors leading to long term damage in these children

Methods: After ethics approval/consents, 23 patients with JDM under regular treatment and at least a 2 year follow up, at first study visit were identified. Children with overlap syndrome were excluded.MDI was assessed as severity of damage and extent of damage at the first study visit and reassessed at second visit at least six months later.Damage present at both visits were scored to give a final severity and extent of damage score. Results: 23 children with age range at disease onset 1y-17.9 y (mean 6.9 y,median 6.8 y,IQR 3.5-8.8) were diagnosed as JDM after a duration of symptoms ranging 1m-30 m (mean 6.8 m,median 5 m,IQR 3-8.5).Age at the study visit ranged from 5.5 y-24.8 y (mean 13.4 y,median 12.9 y,IQR 10.4-15.3) after a follow up duration ranging from 2y-20.1y (mean 5.9 y,median 4.2 y,IQR 2.6-8.5,Total 136.5 patient yrs).Disease course was monocyclic in 14,continuous in 6,polycyclic in 3.Total MDI extent of damage score ranged from 0-8/35 (children) or 37 (adolescents) (mean 2.04,median 2.0,IQR 0.5-2.5) and severity of damage score ranged from 0-24.7/110 (mean 4.7,median 3.5,IQR 1.3-6.4).17/23 children had damage in 1 or more organ systems,with 9 showing damage in one organ system,4 in two organ systems,3 in three organ systems and 1 child showed damage in six systems.Cutaneous (16/23),endocrine ie growth retardation (6/23) and muscle (5/23) were the most commonly damaged organ systems.Two children showed skeletal damage while ocular,gastrointestinal and cardiac domains were involved in one each.Importantly,over this duration of follow up there were no children with infection,pulmonary,peripheral vascular disease or malignancy.Correlation between age at onset,gender,time to diagnosis,course of disease and duration of follow up did not yield statistically conclusive results.

Conclusion: This is amongst the earliest studies to report damage due to JDM from less resourced countries.Over the median follow up period of 4.2 yrs about 75% of the children suffer damage in one or more domains, cutaneous damage being the commonest.Uniquely despite high infection rates in the community,this domain was unaffected in our cohort.Continued enrolment with longer duration of follow up are needed to shed light on factors influencing damage.

### P59 PROFILE OF MYOSITIS ANTIBODIES IN A COHORT OF CHILDREN WITH JUVENILE DERMATOMYOSITIS (JDM) FROM A SINGLE CENTRE IN MUMBAI, INDIA

#### Pallavi Pimpale Chavan, Rajesh Bendre, Raju P. Khubchandani

##### Jaslok Hospital and Research Centre, Mumbai, India

###### **Correspondence:** Raju P. Khubchandani

Introduction: The study of myositis specific and myositis associated autoantibodies (MSA and MAA) in JDM is helping us gain insight into clinical and laboratory features of the disease and even its course and prognosis. Besides few large studies there is limited data from Asian geographies.

Objective: Profiling autoantibodies in a cohort of patients with JDM at our centre seen between 2003-2018.

Methods: We were able to contact 36 patients with conclusive JDM who we have seen at our centre since 2003. After ethics approval /consents blood samples were drawn for qualitative assessment of MSA viz Mi -2 (Mi-2α, Mi-2β), TIF1γ, MDA5, NXP2, SAE1, SRP, anti synthetase (Jo-1, PL-7, PL-12, EJ, OJ) and MAA viz Ku, Ro-52, PmScl (100 &75) using the Immunoblot technique with a kit manufactured by Euroimmune. All testing was performed by a single technician, supervised by the pathologist. Only tests reported as positive/strongly positive were recorded.

Results: Our cohort consisted of 15 males and 21 females in the age range 4 y -23 y (mean 10.4 yrs). 10/36 samples were drawn at diagnosis before starting therapy while 22/36 patients were on therapy and 4/36 were in remission and off therapy. The duration of follow up in the last 2 categories ranged from 0.5 y -14 y (mean 3.12y, median 3y). 25/36 (69.4%) were positive for at least one of the autoantibodies studied, with 24/36 demonstrating at least one MSA (MSA alone - 18, MSA + MAA - 6, MAA alone- 1).13/36 demonstrated only one MSA while 3 showed two MSA and 2 had three MSA. The MSA which tested positive were MDA5 (19.4%), NXP2 (19.4%),Mi-2 (16.6%),TIF1γ (13.8%) and anti synthetase (11.1%) respectively. Of the 7/36 with MAA, 6 had these in combination with one MSA while one child had MAA alone. The MAA detected were Ro52 (11.1%) and PmScl (8.3%). 9/10 treatment naïve patients were positive for at least one autoantibody while 13/22 on treatment were positive. 3/4 who had stopped treatment remained positive for at least one autoantibody.

Conclusions: 66.6% of our cohort test positive for MSA with 50 % having just one MSA. The commonest MSA in our series are MDA5 and NXP2 and the commonest MAA is Ro52. We will be glad to share available clinical and laboratory data with multicentre studies on myositis autoantibodies.

### P60 Correlation of type I interferon score and interferon induced mediators CXCL10 and neopterin with disease activity in Juvenile Dermatomyositis

#### Rebecca Nicolai^1^, Ivan Caiello^1^, Lucilla Rava^1^, Silvia Rosina^2^, Luisa Bracci Laudiero^1^, Angelo Ravelli^3^, Fabrizio De Benedetti^1^, Gian Marco Moneta^1^

##### ^1^Ospedale Pediatrico Bambino Gesù, Rome, Italy; ^2^University of Genova, Genoa, Italy; ^3^Giannina Gaslini Institute, Genoa, Italy

###### **Correspondence:** Rebecca Nicolai

Objectives: The aim of this study was to investigate expression of interferon regulated genes (IRGs), serum levels of two type I and type II IFN induced chemokines (CXCL9, CXCL10) and neopterin in peripheral blood of JDM patients, and to assess their correlations with clinical and laboratory findings.

Methods: We collected 189 blood samples from 39 JDM patients at different time points during follow-up. In 11 patients the first blood sample was obtained at time of muscle biopsy. We measured expression of type I IRGs (IFI27, IFI44L, IFIT1, ISG15, RSAD2, SIGLEC1), IFNgamma and type II IRGs (CXCL9, CIITA, IDO1) by quantitative PCR (qPCR) and calculated a type I and type II IFN score for muscle and blood samples; serum levels of CXCL9, CXCL10 and neopterin were analyzed by ELISA. Ten healthy subjects were used as controls (HC). At each visit, the following clinical data were recorded: physician’s global assessment (PGA) of disease activity VAS (Visual Analogue Scale), cutaneous VAS, Cutaneous Assessment Tool (CAT) activity score, Childhood Myositis Assessment Score (CMAS), serum levels of creatine phosphokinase (CK, IU/l), presence of myositis specific or myositis associated antibodies (MSA/MAA), prednisone (or equivalent) dose (mg/kg/daily), ongoing immunosuppressive medications.

Results: Serum levels of CXCL9 where significantly correlated with muscle expression of IFNgamma and type II IFN score. The correlation of CXCL10 levels with muscle type I and type II IFN score was weaker. Muscle expression of CXCL9 and CXCL10 correlated with serum levels of these chemokines. Type I IFN score in blood of JDM patients was increased compared to HC and significantly correlated with PGA, cutaneous VAS, CAT activity score. Serum levels of CXCL9 and CXCL10 were significantly higher in JDM patients compared to HC. MSA positive JDM patients showed higher levels of CXCL9 and CXCL10 compared to MSA negative patients. CXCL10 levels were correlated with PGA and CMAS, but not with cutaneous disease activity. CXCL9 showed no significant association with the evaluated clinical features. Neopterin levels were significantly correlated with PGA, cutaneous VAS, CAT activity score and CMAS. During the study period two patients experienced a disease flare. A progressive increase of type I IFN score preceeded any clinical or laboratory abnormality, suggesting that type I IFN score might predict flare in JDM patients.

Conclusion: Our findings indicate that type I IFN score, serum levels of CXCL10 and neopterin reflect specific features of disease activity in JDM, supporting their role as valuable disease biomarkers.

### P61 Identifying and predicting novel classes of long-term disease trajectories for patients with juvenile dermatomyositis using growth mixture models

#### Claire T. Deakin, Charalampia Papadopoulou, Muthana Al-Obaidi, Clarissa A. Pilkington, Lucy R. Wedderburn, Bianca L. De Stavola

##### Great Ormond Street Institute of Child Health, London, UK

###### **Correspondence:** Claire T. Deakin

Objectives: Uncertainty around medium- to long-term outcomes for patients with juvenile dermatomyositis (JDM) represents a major burden of disease for patients and their parents, as well as posing a challenge for clinical management. To address this problem, we sought to identify: 1. Novel classes of patients with JDM having similar temporal patterns in global assessment of disease activity; and 2. Clinical features at baseline that predict class membership.

Methods: Data were obtained for n=508 patients recruited to the UK-wide JDM Cohort & Biomarker Study (JDCBS), a registry with predominantly inception patients. Patients had a median and interquartile range of 5.1 [2.6-8.6] years of follow-up. The physician’s global assessment of disease (PGA) was the main long-term outcome of interest which was analysed using growth mixture models (GMM), a probabilistic model-based method for deriving classes of patients with similar trajectories in PGA over time. Baseline predictors included diagnosis, demographic features, autoantibodies, abnormal respiration, ulceration, lipodystrophy, calcinosis, and skin and muscle disease activities as measured by the modified disease activity score for skin (modDAS) and childhood myositis assessment scale (CMAS), respectively. Clinical features and medications that characterised the classes over time were also described. Baseline predictors of class membership were identified in a second step using lasso regression, a method involving automatic selection of predictors and regularisation of coefficients. R packages “lcmm” and “glmnet” were used for the GMM and lasso regression analyses, respectively.

Results: GMM identified two classes of patients. Patients in Class 1 (88.6%) tended to improve their PGA over time, while patients in Class 2 (11.6%) tended to have more ongoing disease. Higher proportions of patients in Class 2 had arthritis, ulceration, lipodystrophy, calcinosis and abnormal respiration ever during their disease course. Lasso regression analysis identified modDAS, abnormal respiration and lipodystrophy as baseline predictors of Class 2 membership, with estimated odds ratios of 0.96, 1.48 and 1.68 respectively. Interestingly, demographic features and myositis-specific or -associated autoantibodies had similar prevalence across the two classes.

Conclusions: We have identified two classes of patients with different trajectories of global disease activity, using a large number of patients and a data-driven approach to defining long-term outcomes in JDM. Baseline predictors of being in the class with more ongoing disease include modDAS and having abnormal respiration or lipodystrophy. Ongoing work will use GMM to model skin disease activity, and identify genetic and other biological predictors of class membership.

### P62 Myositis-Specific Antibodies and Acquired Lipodystrophy in Juvenile Dermatomyositis: Evaluation of Fat Distribution using Dual-Energy X-ray Absorptiometry (DXA)

#### Amer Khojah^1^, Victoria Liu, Gabrielle Morgan^2^, Chiang-Chiang Huang^3^, Kaveh Ardalan^1^, Richard Shore^1^, Jackie Bellm^1^, Lauren Pachman^1^

##### ^1^Ann & Robert H. Lurie Children's Hospital, Chicago, USA; ^2^Stanley Manne Research Center, Chicago, USA; ^3^University of Wisconsin-Milwaukee, Milwaukee, USA

###### **Correspondence:** Amer Khojah, Kaveh Ardalan

Objectives: Juvenile dermatomyositis (JDM) is the most common idiopathic inflammatory myopathy in childhood. Myositis-specific antibodies (MAS) have been utilized to categorize JDM into multiple distinct phenotypes. Of these MSA, p155/140 (aka anti-TIF-γ) antibody is the most common and has been associated with generalized lipodystrophy in small retrospective studies. The aim of this study to investigate the relationship between lipodystrophy and p155/140 antibody using DXA scan, which is a more objective tool to measure fat distributions.

Methods: An IRB approved chart review study (IRB# 2012-14858) was conducted at Ann & Robert H. Lurie Children’s Hospital of Chicago on patient records spanning 2000 to 2017. We included all subjects with JDM who had at least 5 years of follow up data and DXA scan done during the study period. DXA was performed using GE-LUNAR iDXA bone densitometer and data were analyzed by Encore 16 software. Subjects with overlap syndrome were excluded from the analysis. We divided the study population into three groups based on their MSA (p155/140 antibody, other MSA, and MSA negative). We used One-way ANOVA and chi-square to compare the baseline characteristics and fat distribution (Trunk:Legs fat ratio) among the 3 groups.

Results: 96 subjects (78% female, 70% white, 44% p155/140 antibody, 23% other MSA, 33% MSA negative) were included. There were no significant difference in the initial total and skin disease activity scores (DAS) between the 3 groups, though DAS muscle domain did differ (4.3 in P155/140 group vs 5.8 and 5.9 in other MSA and MSA negative group respectively and p = 0.049). Interestingly, p155/140 group had decreased nailfold capillary end row loop (NFC-ERL) counts compared with the other groups (p = 0.006). Percentage of total body fat and trunk: legs fat ratio were similar among the 3 groups at different time points. Percentages of subjects with lipodystrophy by physician assessment were the same in all three groups (p = 0.69)

Conclusion: P155/140 antibody group have lower baseline NFC-ERL counts than the other two groups. Generalized lipodystrophy did not differ by MSA groups based on DXA and physician assessment.

### P63 Investigating Investigating the role of complement activation in driving the Th1/17 imbalance observed in Juvenile Dermatomyositis

#### Lucy Marshall, Elizabeth Rosser, Claire Deakin, Cerise Johnson-Moore, Despina Eleftheriou, Lucy Wedderburn

##### Great Ormond Street Institute of Child Health, London, UK

###### **Correspondence:** Lucy Marshall

Objectives: A skewed Th17 phenotype in CD4+ T cells resulting in a Th1/17 imbalance has been observed in both child and adult-onset immune-mediated diseases including rheumatoid arthritis, SLE (systemic lupus erythematosus), and multiple sclerosis. This project aimed to investigate whether a Th1/17 imbalance can also be observed in patients with Juvenile Dermatomyositis (JDM) compared to age/sex-matched healthy controls.

Methods: PBMC from JDM (n=15) and age/sex-matched controls (CHC, n=6) were stimulated with PMA/Ionomycin in the presence of Brefeldin A for 4 hours with the Th1/17 phenotypes measured by intracellular staining for IFN-γ and IL-17 respectively. Using previously generated RNAseq data within the Wedderburn lab, immune pathways defined by hallmark gene sets, were analysed within peripheral blood CD4+ T cells from treatment-naïve patients and patients after approx. 12 months of standard treatment (n=10). To further investigate Th1 response and the role of complement an in vitro CD46-activation, assay was performed on JDM (n=6) and CHC (n=3). Read outs of the assay included IFN-γ and IL-10 secretion via cytokine bead array.

Results: Analysis of the ratio of CD4+IFN-γ+ to CD4+IL-17+ cells within peripheral blood demonstrated that the CD4+ T cell phenotype is significantly skewed towards Th17 cells in JDM (p=0.04) compared to CHC. Isolated CD4+ T cells included in RNAseq analysis revealed that the complement pathway was over-represented in pre-treatment JDM versus on-treatment JDM CD4+ T cells. Using an in vitro modelling assay to mimic intracellular complement activation in CD4+ T cells via anti-CD46 activation, a complement receptor known to induce Th1 responses, our preliminary analysis revealed that there is also low IFN-γ production in JDM compared to CHC under these conditions.

Conclusions: IFN-γ is reduced in CD4+ T cells from JDM patients compared to CHC leading to a Th17-skewed immunophenotype. Consistent with this observation, RNAseq analysis highlighted the complement pathway in CD4+ T cells from treatment naïve JDM patients compared to patients on treatment. Importantly, intracellular complement activation within CD4+ T cells has been shown to be critical for Th1 induction in healthy adults. To summarise, our initial results display a lack of Th1 response, via IFN-γ, in JDM patients compared to CHC. Future work aims to confirm these findings and investigate possible mechanisms involved.

### P64 A single centre experience of immune-mediated necrotising myopathies: a comparison of anti-3-hydroxy-3-methylglutaryl-coenzyme A reductase and anti-signal recognition particle myopathies

#### Su-Ann Yeoh^1^, Rhys Thomas^1^, Hasan Tahir^1^, Aleksander Radunovic^2^

##### ^1^Whipps Cross University Hospital, London, UK; ^2^Royal London Hospital, London, UK

###### **Correspondence:** Su-Ann Yeoh

Objectives: Immune-mediated necrotising myopathy (IMNM) is a rare condition characterised by muscle fibre necrosis with minimal inflammation. IMNM is associated with anti-signal recognition particle (SRP) and anti-3-hydroxy-3-methylglutaryl-coenzyme A reductase (HMGCR) antibodies. IMNM patients have been reported to be more resistant to conventional immunosuppression with anti-SRP myopathy following a more severe disease course. We analysed our IMNM cohort, comparing disease characteristics and treatment outcomes of anti-SRP and anti-HMGCR patients.

Methods: Subjects diagnosed with IMNM from 2008 to 2018 were included in this study. Electronic patient records were reviewed. Patient characteristics and treatment outcomes were compared.

Results: 31 subjects were included, with 20 anti-HMGCR and 11 anti-SRP subjects. There was a female predominance in the anti-HMGCR group, with 15 (75%) versus 6 anti-SRP subjects (55%). Median age of anti-HMGCR subjects was 65 vs 52 in the anti-SRP group. There was a Caucasian predominance in the anti-HMGCR group (75%, 15/20 subjects) with an African/African-Caribbean predominance in the anti-SRP group (91%, 10/11 subjects). Statin exposure was prevalent in the anti-HMGCR group (90%, 18/20 subjects). Cardiac involvement was present in 2 anti-SRP subjects. Malignancy was present in 3 anti-HMGCR subjects vs 1 anti-SRP subject. 11 anti-HMGCR and 7 anti-SRP subjects were treated with ‘triple therapy’ – IV methylprednisolone followed by oral prednisolone, intravenous immunoglobulin (IVIg) and methotrexate (MTX)/azathioprine. 6 of the 11 anti-HMGCR subjects treated with triple therapy had complete response, not requiring further treatment. The remaining 5 responded initially but relapsed, treated with IVIg to good effect. 2 of the 7 anti-SRP subjects responded to triple therapy not requiring further treatment. 5 (46%) anti-SRP subjects were treated with rituximab compared to 3 (15%) anti-HMGCR subjects. 3 out of 5 anti-SRP subjects responded to rituximab clinically and biochemically (with 2 patients being followed up by local hospitals). One anti-HMGCR subject responded clinically but not biochemically, one failed to respond requiring plasma exchange and another anti-HMGCR subject has only recently been treated with rituximab.

Conclusions: IMNM is a rare condition often described to follow a severe disease course, refractory to standard therapy. We demonstrate that in our cohort, anti-SRP myopathy was more common in African/African-Caribbean patients and associated with cardiac involvement. Anti-HMGCR subjects responded more frequently to conventional immunosuppression compared to anti-SRP subjects. Anti-SRP subjects were treated with rituximab more frequently. Our study illustrates the heterogeneity in treatment choice given the paucity of data, highlighting the need for prospective studies and standardised protocols.

### P65 Muscular dystrophy masquerading as inflammatory myositis

#### Char Loo Tan, Kong Bing Tan, Stacey Tay, Pei Ling Ooi, Elizabeth Y. Ang

##### National University of Singapore, Singapore, Singapore

###### **Correspondence:** Elizabeth Y. Ang

Objective: The limb-girdle muscular dystrophies (LGMD) are a diverse group of disorders caused by mutations in different genes. Infrequently, some cases may show superimposed inflammatory changes, e.g. cases of dysferlinopathy, resulting in diagnostic confusion.

Method: We report a case of genetically confirmed calpainopathy masquerading as an immune-mediated necrotizing myopathy in a 7-year-old girl and highlight the diagnostic dilemma.

Result: Physical examination revealed only mild proximal weakness. Serological tests showed a markedly elevated creatine kinase, a positive anti-MDA5, anti-nuclear antibody of 1:80 and a negative anti-HMGCR. Muscle biopsy revealed patterns suggestive of necrotizing myopathy with some lymphocytic and eosinophilic infiltrates. Notably, MHC1 was diffusely upregulated. Endomysial fibrosis or fatty infiltration was not identified. The patient was treated with immunosuppressant therapy but with minimal transient response after 12 months. Genetic testing for muscular dystrophies later revealed 2 pathogenic variants in CAPN3, clinching the diagnosis of calpainopathy.

Conclusion: Calpainopathy can rarely show pronounced necrotizing features or even diffuse MHC1 upregulation on muscle biopsy, mimicking an immune-mediated necrotizing myopathy. Re-evaluation of a patient who does not respond as expected to immunomodulatory therapy is vital, even in the presence of myositis-specific antibodies. In our patient, genetic studies allowed for definitive diagnosis prior to the onset of symptoms of the dystrophy and also precluded the need for a repeat biopsy.

### P66 Chronic disease course and IVIg-dependance in long-term follow-up of anti-HMGCR immune-mediated necrotizing myopathy

#### Océane Landon-Cardinal, Kuberaka Mariampillai, Céline Anquetil, Aude Rigolet, Baptiste Hervier, Nicolas Champtiaux, Olivier Benveniste, Yves Allenbach

##### Pitié-Salpêtrière University Hospital, Paris, France

###### **Correspondence:** Océane Landon-Cardinal

Objectives: Anti-HMGCR antibodies have been associated with a severe form of immune-mediated necrotizing myopathy (IMNM) with a poor muscle strength recovery and early muscle damage. These patients tend to require aggressive immunosuppressive therapy and present relapsing disease course. Our objective was to evaluate real-life treatment strategies in anti-HMGCR IMNM patients.

Methods: This monocentric study included all patients with anti-HMGCR IMNM with at least 12 months of follow-up. Medical records were retrospectively reviewed to assess clinical features at diagnosis, HLA typing, treatment strategies over the follow-up period (including corticosteroid (CS) use, number of immunosuppressive (IS) agent and intravenous immunoglobulin (IVIg) duration), disease course, and clinical status and therapeutic profile at last follow-up. Remission was defined as the presence of CK level ≤ 2 times the upper limit of normal associated with a stable manual muscle testing for ≥3 months. Quantitative variables are reported as median [IQR1-IQR3].

Results: Thirty-five patients were included. Age at diagnosis was 47.1 [26.1-60.2] years, 74% of patients were female, 29% were statin-exposed, all patients presented with muscle weakness (deltoid and psoas MRC-5 was 4.0 [2-4] and 4.0 [3-4], respectively) and highest CK level was 8146 [5000-12090] IU/L. Time from symptoms onset to treatment initiation was 0.8 [0.3-4.7] years. During the follow-up period, 91% of patients were treated with CS in combination with an IS agent, the majority of patients received IVIg (91%) and the number of treatment intensification was 2 [1-4]. Fourty percent of patients also received plasma exchanges as part of induction therapy. All patients demonstrated a chronic disease course and no patients were in treatment-free remission at last follow-up after 4.9 [3.1-8.9] years. At last follow-up, 60% of patients were in remission - most of which with IVIg (71%) -, 57% of patients were still receiving CS and CS dose was 8 [5-10] mg/day and 54% of patients had an IS agent. At last follow-up, only 40% of patients had a normal muscle strength, deltoid and psoas MRC-5 was 5 [4-5] and 4 [3-5], respectively, and CK level was 299 [200-559] IU/L. No predictors of remission or IVIg use at last follow-up were identified, including demographic features, HLA-DRB1*11:01 and HLA-DRB1*07:01 status, muscle disease severity at onset and statin use. Therapy-related side effects were reported in 26% of patients.

Conclusion: In our population, anti-HMGCR IMNM was associated with a chronic disease course associated with IVIg-dependance.

### P67 Clinical and histological features of necrotising myopathy: a prospective multi-centre Australian cohort study

#### Jessica Day, Sophia Otto, Kathy Cash, Vidya Limaye

##### University of South Australia,Adelaide, Australia

###### **Correspondence:** Jessica Day

Background/Purpose: Differentiation of necrotising myopathy (NM) from other forms of idiopathic inflammatory myopathy (IIM) has important therapeutic implications. Herein we describe the demographic, clinical and histological features of a consecutive cohort of NM patients in order to define the spectrum of disease, characterise the features that distinguish NM from other IIM subtypes and identify distinct phenotypes of NM.

Methods: Muscle samples and clinical details were prospectively collected from patients with NM (n = 63), other forms of IIM (n = 45) and controls (n = 17). Consecutive muscle sections were stained using immunohistochemistry and graded by a muscle pathologist. Clinical and histological subgroups of NM were defined and compared.

Results: NM patients displayed marked clinical, biochemical and histological heterogeneity. Twenty-four percent (15/63) demonstrated widespread muscle necrosis, with the remainder exhibiting only mild (23/63, 37%) or moderate (25/63, 40%) necrosis. There was notable variability in creatine phosphokinase (CK) levels (range: 23 – 219000 IU/L) and muscle strength. Patients of Aboriginal and Torres Strait Islander heritage appear to suffer a severe form of the disease. Sarcolemmal and sarcoplasmic complement deposition was common and correlated with the degree of necrosis and clinical severity. Those who were seropositive for myositis-specific autoantibodies (MSAs) featured higher capillary complement deposition (p = 0.02) and more widespread sarcolemmal MHC I expression (p = 0.04) compared with seronegative patients.

Conclusions: We found NM to be a markedly heterogeneous condition histologically, and identified certain clinical and histological features which are associated with severe forms of the disease. In addition, we identified capillary complement deposition and widespread MHC I deposition to be associated with features of systemic immune activation; routine evaluation for these could have diagnostic and therapeutic utility.

### P68 Tocilizumab Improve Muscle Strength in Patients with Refractory Anti-Signal Recognition Particle and Anti-3-Hydroxy-3-Methylglutaryl-CoA Reductase Myopathies

#### Sizhao Li, Wenli Li, Wei Jiang, Linrong He, Xia Liu, Xiaoming Shu, Qinglin Peng, Li Ma, Guochun Wang, Xin Lu

##### China-Japan Friendship Hospital, Beijing, China

###### **Correspondence:** Sizhao Li

Objective: This study aimed to assess the clinical response of tocilizumab (TCZ), a humanized anti-interleukin-6 (IL-6) receptor monoclonal antibody, in a series of patients with refractory anti-signal recognition particle (SRP) and anti-3-hydroxy-3-methylglutaryl-CoA reductase (HMGCR) myopathies and explored the role of IL-6 in muscle weakness.

Methods: The clinical, laboratory, and histopathological data of 11 patients with refractory anti-SRP and anti-HMGCR myopathies treated with TCZ were analyzed. The clinical response was assessed by the changes in Manual Muscle Testing 8 (MMT-8) scores and serum creatine kinase (CK) levels. Serial muscle biopsies were conducted to compare the changes in muscular pathology.

Results: After 3–7 rounds of TCZ, the mean dosage of adjunctive prednisone was reduced from 36.8 to 11.8 mg/day. Nonetheless, the MMT-8 score increased from 42.2 (±4.5) to 75.9 (±5.9) and all patients achieved more than 50% improvement in the MMT-8 score. The serum CK decreased from 3105.9 (±2555.8) to 614.1 (±681.3) IU/L and all patients achieved more than 25% improvement in serum CK levels. Before TCZ treatment, the muscle samples showed local expression of IL-6 around infiltrating macrophages, prominent regeneration (25.7% ± 14.8%, n = 9), and high levels of atrophy factor (562.7±146.2, n = 9). After TCZ treatment, the levels of atrophy factor markedly reduced from 535.2 ± 171.9 to 75.4 ± 68.6 (n = 5), and the impaired regeneration substantially improved.

Conclusions: TCZ is a promising therapeutic agent for refractory anti-SRP and anti-HMGCR myopathies. It recovers muscle regeneration and improves muscle strength.

### P69 Clinical Characteristics of Immune-Mediated Necrotizing Myopathies: A Retrospective Study in a Single-center Muscle Biopsy Cohort

#### Hongxia Yang, Xiaolan Tian, Hanbo Yang, Yamei Zhang, Xiaoming Shu, Guochun Wang, Xin Lu

##### China Japan Friendship Hospital, Beijing, China

###### **Correspondence:** Hongxia Yang; Xin Lu

Objective: Immune-mediated necrotizing myopathies(IMNM) is recently entitled a novel subset of idiopathic inflammatory myopathies(IIM) which patients has predominant muscle weakness and are resistant to conventional therapy. The aim of this study was to investigate the frequency and characteristics of patients with IMNM in our single-center muscle biopsy cohort.

Methods: A total of 512 patients who were admitted to our hospital for muscle biopsy due to muscle weakness or elevated serum level of creatine kinase (CK) from May 2008 to December 2017 were involved in this study. All clinical data including demographic information, clinical manifestations, laboratory tests, the reports of muscle biopsy were collected retrospectively from the record of Hospital Information System. The diagnosis of IMNM was according to 2004 the European Neuromuscular Centre criteria for pathological features of muscle biopsy of IMNM or patients with anti-SRP or anti-HMGCR autoantibodies.

Results: Of 512 patients in our cohort, 71 of patients (13.8%) were diagnosed as IMNM and 39 of patients were polymyositis (PM) (7.6%). Of 253 patients (49.4%) were definitely diagnosed as non-IIM such as muscular dystrophy(MD) (11.9%), asymptomatic hyperCKemia (9.1%), lipid storage disease(LSD) (3.1%), neurogenic myopathy (3.7%), infection-related myopathies (2.7%), endocrine myopathies (2.5%) and other connective tissue disease accompanied muscle symptom (12.1%). Of 13 patients (2.5%) were diagnosed as certain disease unrelated to muscular disorders. Of 63 cases (12.3%) cannot be diagnosed as definited disease. Compared the characteristics of IMNM with PM, MD and LSD in our cohort, which those patients had similar manifestations of muscle weakness and high CK level, the age of onset of patients with IMNM and PM were older than those with MD and LSD (IMNM vs PM, MD and LSD: 41.63 vs 40.14, 24.72 and 24.69, p < 0.001). IMNM patients had more manifestation of upper limb weakness (56.3%), while LSD patients more frequently presented lower limb weakness (75%). Patients with IMNM had more frequent manifestations of rash (23.9%), interstitial lung disease (31%) and dysphagia (36.6%) compare to patients with PM, MD and LSD. Patients with MD had the highest CK level (3324.50 ± 874.25), however patients with PM had the lowest CK level (450.50 ± 121.25), there were statistical significant difference of CK level among the four groups (p < 0.001).

Conclusions: IMNM is the most common subset in the patients with muscle weakness and high CK level in our single-center muscle biopsy cohort, which should be differentiated from PM, MD and LSD.

### P70 The role of dysfunctional T-cells in immune mediated myositis

#### Samuel Knauss^1^, Corinna Preuße^1^, Yves Allenbach^2^, Sarah Leonard-Louis^2^, Mehdi Touat^2^, Norina Fischer^1^, Helena Radbruch^1^, Ronja Mothes^1^, Vitaly Matyash^1^, Wolfgang Boehmerle^1^, Matthias Endres^1^, Hans-Hilmer Goebel^1^, Olivier Benveniste^2^, Werner Stenzel^1^

##### ^1^Charité Universitätsmedizin Berlin, Berlin, Germany; ^2^Pitié-Salpêtrière University Hospital, Paris, France

###### **Correspondence:** Samuel Knauss

Objective: The objective of the present study was to investigate the presence of T cells exhibiting dysfunctional phenotypes in immune-mediated myopathies. To this effect we analysed T cell exhaustion and senescence markers, in skeletal muscle biopsies from patients with IMNM, sIBM and myositis induced by immune checkpoint inhibitors (irMyositis).

Methods: We analysed skeletal muscle biopsies from 12 IMNM, seven sIBM and eight irMyositis patients using immunostaining and immunofluorescence as well as by qPCR. Eight biopsies from non-disease subjects and six biopsies from patients with Duchenne muscular dystrophy served as controls.

Results: In double-immunostainings of biopsies from IMNM, sIBM and irMyositis patients, CD3+CD8+ T cells were largely PD1-positive, while CD68+ macrophages were sparsely PD-L1-positive. PD-L2 was strongly expressed on the sarcolemma of myofibers and co-localised with MHC class I. CD68+ macrophages co-localised with PD-L2. In qPCR analyses we found a strong signal of senescent T cells in skeletal muscle of sIBM, revealing a distinct immunological signature. Biopsies from patients with irMyositis showed mild signs of senescence and exhaustion. Of note, biopsies from Duchenne boys did not exhibit any molecular signs of senescence or exhaustion.

Conclusion: We found evidence of T cells expressing markers of exhaustion and senescence in IMNM, sIBM and irMyositis. Activation of the pathways involving the programmed cell death protein 1 (PD1) receptor and its cognate ligands PD-L1/PD-L2 have been linked to a persistent exposure of antigens. To our knowledge, this data is the first evidence of presence of dysfunctional T cells in IMNM, sIBM and irMyositis. These findings may guide the way to a novel understanding of the immune pathogenesis of immune-mediated myopathies.

### P71 Use of Protein Convertase Subtilisin/Kexin Type 9 (PCSK9) inhibitors in statin-associated autoimmune myopathy; a case-series

#### Eleni Tiniakou^1^; Andrew Mammen^2^; Lisa Christopher-Stine^1^

##### ^1^Johns Hopkins University School of Medicine, Baltimore, USA; ^2^National Institute of Health, Bethesda, USA

###### **Correspondence:** Eleni Tiniakou

Objective: Statin-triggered immune mediated necrotizing myopathy (IMNM) is associated with autoantibodies against HMGCR, the pharmacologic target of statins. Re-introduction of statins is avoided or suboptimal dosing is attempted with careful monitoring. As these patients commonly have a high cardiovascular (CV) risk, physicians face a conundrum as the recommendations for a lipid-lowering diet can only achieve minimal results. The introduction though of PCSK9 inhibitors not only promoted a more effective cholesterol control, but also raised the question of their safety in patients with anti-HMGCR IMNM.

Methods: Amongst 122 anti-HMGCR+ myositis patient evaluated at the Johns Hopkins Myositis Center, we identified nine patients with severe CV disease and/or diabetes who were placed on a therapeutic trial with PCSK9 inhibitors.

Results: The mean age at initiation of PCSK9 inhibitors was 69.75 years and the mean duration of anti-HMGCR myositis was 7 years (SD 5.73 years). Six patients were placed on evolocumab and three on alirocumab. The average duration of follow-up on PCSK9 inhibitors was 19 months (2-31 months). For two of the patients, the initiation of PCSK9 inhibitors was concurrent with changes in their immunosuppressive treatments, while the rest were clinically stable. The mean CK level prior to the initiation of PCSK9 inhibitors was 943±1135 IU per liter and at the following visit it was slightly reduced to 810±1142. The mean CK at the most recent evaluation was substantially lower at 395±391, which is attributed to intense immunosuppressive treatment of the two active myositis patients. Parallel trend was observed for the titers of anti-HMGCR antibodies. Similarly, there was no decrease in the hip flexion and deltoid muscle strength during their follow up visits. Remarkably, two patients placed on PCSK9 inhibitors exhibited improvement of their CPK and muscle strength without the addition of immunosuppression.

Conclusion: The exact pathogenesis of anti-HMGCR IMNM and the link with statins remain unknown. Statin-induced overexpression of HMGCR in muscle tissue and/or statin binding to HMGCR potentially revealing cryptic epitopes could lead to initiation and propagation of an autoimmune response. Therefore, statins are avoided in these patients. However, we cannot dismiss an association with different steps of the cholesterol pathway, and therefore, we could not exclude similar interaction with PCSK9 inhibitors. We present nine patients with severe CV risk who were able to tolerate PCSK9 inhibitors without exacerbation of underlying myopathy. Thus, our experience to date suggests that PCSK9 inhibitors are safe in anti-HMGCR IMNM patients with high CV risk.

### P72 Clinical presentation and outcome in anti-SRP IMNM: french cohort of 54 patients

#### Marion Peyre, Nicolas Champtiaux, Kuberaka Mariampillai, Océane Landon-Cardinal, Mathieu Vautier, Baptiste Hervier, Perrine Guillaume-Jugnot, Aude Rigolet, Yves Allenbach, Olivier Benveniste

##### Hôpital Pitié Salpêtrière, Paris, France

###### **Correspondence:** Marion Peyre

Objective: Anti-SRP Immune-Mediated Necrotizing Myopathies (IMNM) were identified in 2004, and represent a rare cause of auto-immune myopathy, with a particularly brutal presentation and high CPK level. Clinical caracteristics of anti-SRP myopathies are now well described. Thus, their evolutive profil is less studied.

Method: We report a retrospective cohort of 54 adult patients with anti-SRP necrotizing myopathy, based upon a review of every successive patient with anti-SRP admitted in our tertiary center since 2000.

Results: Fifty-four patients were included (sex ratio M/F: 0,4), and we disposed of follow-up datas for 42 of them. Mean age at symptoms was 44 years old. First signs were myalgias (61%), amyotrophia (48%), and muscular impairment. Mean MMT8 score was 109 (for a maximal score of 150 points), and seven patients had a MMT8 < 80. Fourty-eight percent of patients presented swallowing disorders. Seventeen patients (31%) had cardiac involvement at MRI, and 27 (50%) had pulmonary involvement. Notably we observed that black patients tended to have a more rapid onset of disease (time between first symptoms and the diagnosis: 4 +/- SD months) compare to the other patients (vs 18+/- months; p=0,08) and the CPK level was higher (12.558UI/L vs 7.130UI/L, p=0,008). Patients received an average number of 2,4 disease-modifying antirheumatic drugs (DMARDs). All patients but one received corticosteroids, and 35% encountered severe adverse events. Methotrexate, Azathioprine and Rituximab were given to respectively 79%, 46% and 31 patients. Fourty-five patients (83%) received at least one perfusion of IVIg. Mean follow-up duration was 86 +/- SD months after diagnosis. Seventeen patients (40%) only recovered a normal muscular function, and twenty three (54%) had low disabily attested by a Rankin scale < 2. Thirty-five percent of patients still have an active disease at the end of the follow-up, defined by either CPK rate higher than 3N or clinical progression. Black patients had more refractory disease (10/14 patients still have active disease, vs 5/25 in Caucasian patients, p=0,004). Thirty-four patients were still treated with corticosteroids at the end of the follow-up.

Conclusion: This cohort is, to our knowledge, the biggest in Europe. Our results are consistent with litterature, except that we found higher rate of pulmonary and cardiac involvement. Originally, a new pattern is described here, with a more severe and more refractory disease in black patients.

### P73 Anti-HMGCR antibodies as a biomarker for immune-mediated necrotizing myopathies: Experience from a large single center study

#### Maryam Dastmalchi^1^, Simone Barsotti^2^, Andrew Mammen^3^, Katherine Pak^3^, Karina Gheorghe^1^, Ingrid Lundberg^1^

##### ^1^Karolinska University Hospital, Stockholm, Sweden; ^2^University of Pisa, Pisa, Italy; ^3^National Institute of Arthritis and Musculoskeletal and Skin Diseases, Bethesda, USA

###### **Correspondence:** Maryam Dastmalchi

Objectives: The immune-mediated necrotizing myopathies (IMNMs) are a group of acquired autoimmune muscle disorders characterized by proximal muscle weakness, high levels of creatinine kinase (CK), and infrequent extra-muscular involvement. Muscle biopsies in IMNM differ from other subgroups of idiopathic inflammatory myopathies (IIM) by the presence of myofiber necrosis and prominent regeneration without focal endomysial collections lymphocytic inflammatory infiltrates. Perifascicular atrophy has been reported in 20% of patients. Anti-HMGCR myopathy is a form of IMNM traditionally associated with statin exposure and may be resistant to standard immunosuppressive agents, particularly among younger statin-naïve patients.

Methods: Patients with IIM were identified in the SweMyoNet registry between 1988 and 2014 and classified according to 2017 EULAR/ACR criteria. The first available serum sample was analyzed for anti-HMGCR autoantibodies by ELISA. Positive sera were confirmed by immunoprecipitation. Clinical data were extracted from SweMyoNet or medical records. Demographic data, statins exposure status, disease activity at presentation and at last follow up, and muscle biopsy features were compared between HMGCR+ and HMGCR- groups. Comparisons between groups were evaluated using non-parametric statistical tests (Mann-Whitney).

Results: 312 patients were included, and 13 (4.16%) were anti-HMGCR+. Women accounted for 12 (92.3%) of HMGCR+ individuals and 182 (60.9% p = 0.021) of anti-HMGCR- patients. Age at onset was 70.5 for HMGCR+ and 67.2 for HMGCR-, NS. None of the anti-HMGCR+ patients had been exposed to statins. There was no difference between the anti-HMGCR+ and negative group concerning age at disease onset, sub-diagnosis of IIM, extra-muscular involvement such as heart/skin involvement or ILD, muscle function measured by MMT8 and HAQ or serum levels of CK. At last evaluation, the anti-HMGCR+ patients had higher serum CK levels without other differences in outcome measures. There was no difference in the use of disease modifying drugs or biologic agents between the two groups except for IVIg, which was more often used in HMGCR+ patients p < 0.041. The prevalence of coexisting myositis-specific or myositis-associated autoantibodies was not significantly different between anti-HMGCR+ and anti-HMGCR- groups. We found a tendency towards increased presence of Ro52 antibodies in the HMGCR- group. In muscle biopsies, perifascicular atrophy was presented in 40% in the HMGCR+ group and 13.4% in HMGCR- group p= 0.041.

Conclusion: In this cohort of Swedish IIM patients, the prevalence of anti-HMGCR autoantibodies was low (4.1%) and all were statin naive. The high frequency of perifascicular atrophy in muscle biopsies was surprising and requires further evaluation.

### P74 Anti-RNP antibodies delineate a subgroup of necrotizing myositis

#### Nadège Wesner, Akinori Uruha, Shigeaki Suzuki, Kuberaka Mariampillai, Benjamin Granger, Nicolas Champtiaux, Aude Rigolet, Yoland Schoindre, Sylvain Le Jeune, Perrine Guillaume-Jugnot, Mathieu Vautier, Baptiste Hervier, Anne Simon, Francoise Granier, Laure Gallay, Ichizo Nishino, Olivier Benveniste, Yves Allenbach

Objectives: Myositis can occur as a specific entity or as a part of a spectrum of non-specific connective tissue disorders. Myositis-specific antibodies are known to delineate subgroups of myositis; however, the ability of myositis-associated antibodies to isolate homogeneous groups of patients remains unknown. Anti-U1 small nuclear ribonucleoprotein particle (RNP) antibodies are among the most prevalent myositis-associated antibodies. It may also be detected in mixed connective tissue disease, systemic lupus erythematosus, systemic sclerosis or Sjögren’s syndrome without myositis. We aimed to describe the clinical and pathological myositis phenotypes associated with this particular auto-antibody, and to compare them to myositis patients without anti-RNP antibodies.

Methods: In this international multicentric (France and Japan) retrospective study, myositis patients with anti-RNP antibodies with positive RNA immunoprecipitation were enrolled and compared to a cohort of myositis patients without anti-RNP antibodies. Clinical and pathological data were analysed. Unsupervised descriptive methods were used to compare patients.

Results: Unsupervised hierarchical cluster analysis of the global cohort including anti-RNP- myositis (n=208) identified four clusters. One cluster corresponded to the anti-RNP patients: indeed, 70% of patients with anti-RNP antibody were in this cluster and presented the classical anti- RNP+ phenotype. The three others clusters corresponded to well-known myositis entities: immune-mediated necrotizing myopathies, anti-synthetase syndrome with anti-Jo1 antibodies and dermatomyositis. Anti-RNP+ patients (n=46) were characterized by severe necrotizing myositis associated with frequent extra-muscular manifestations, including Raynaud phenomenon and/or puffy hands, arthralgia and/or interstitial lung disease. Only, twelve anti-RNP+ patients were also positive for myositis-specific antibodies. Patients’ outcome was characterized by a frequent therapeutic intensification (14/20, 70%), a frequent remission rate (14/20, 70%) but also by remaining muscle damage for 20% of patients (4/20).

Conclusion: Anti-RNP+ myositis display characteristic muscular and extra-muscular phenotype, distinct from anti-RNP- myositis. This leads to distinction of anti-RNP+ myositis as a particular subgroup of idiopathic inflammatory myopathies.

### P75 Myositis autoantigen expression correlates with muscle regeneration but not autoantibody specificity

#### Iago Pinal-Fernandez^1^, David R. Amici^1^, Cassie Parks^1^, Assia Derfoul^1^, Maria Casal Dominguez^1^, Katherine Pak^1^, Richard Yeker^1^, Paul Plotz^1^, Jose C. Milisenda^2^, Josep M. Grau-Junyent^2^, Albert Selva-O'Callaghan^3^, Julie Paik^4^, Jemima Albayda^4^, Andrea Corse^4^, Tom Lloyd^4^, Lisa Christopher-Stine^4^, Andrew Mammen^1^

##### ^1^National Institutes of Health, Bethesda, USA; ^2^Clinic Hospital, Barcelona, Spain; ^3^Valle de Hebron, Barcelona, Spain; ^4^Johns Hopkins University, Baltimore, USA

###### **Correspondence:** Iago Pinal-Fernandez

Objectives: Although more than a dozen myositis-specific autoantibodies (MSAs) have been identified, most myositis patients produce a single MSA. The specific overexpression of a given myositis autoantigen in myositis muscle has been proposed to initiate and/or propagate autoimmunity against that autoantigen. To test this hypothesis, we quantified autoantigen RNA expression in myositis muscle biopsies, regenerating mouse muscles, and cultured human muscle cells.

Methods: RNA-sequencing was performed on biopsies from MSA-positive myositis patients, regenerating mouse muscles, and cultured human muscle cells.

Results: Muscle biopsies were available from 106 patients with autoantibodies recognizing HMGCR, SRP, Jo1, NXP2, Mi2, TIF1γ, or MDA5. The increased expression of a given autoantigen in myositis muscle was not associated with autoantibodies recognizing that autoantigen. In both myositis muscle biopsies and regenerating mouse muscles, myositis autoantigen expression correlated directly with the expression of markers of muscle regeneration and inversely with the expression of genes encoding mature muscle proteins. Myositis autoantigens were also expressed at high levels in cultured human muscle cells.

Conclusions: Most myositis autoantigens are highly expressed during muscle regeneration, which may relate to the propagation of autoimmunity. However, factors other than specific autoantigen overexpression are likely to govern the development of unique autoantibodies in individual patients.

### P76 Aberrant expression of ‘High Mobility Group Box Protein 1” (HMGB1) in the idiopathic inflammatory myopathies

#### Jessica Day, Sophia Otto, Kathy Cash, Preethi Eldi, Susanna Proudman, Pravin Hissaria, John Hayball, Vidya Limaye

##### University of South Australia, Adelaide, Australia

###### **Correspondence:** Jessica Day

Objectives: High Mobility Group Box Protein 1 (HMGB1), is a ubiquitous nuclear DNA-binding protein that can translocate to the cytoplasm and extracellular space, where it exerts pro-inflammatory or pro-repair effects depending on its molecular state and the surrounding cytokine milieu. Herein, we evaluate expression of HMGB1 in different forms of IIM and correlate it with other intramuscular histological processes to determine the role(s) of this complex protein in these conditions.

Methods: Consecutive muscle sections were obtained from 132 IIM patients with necrotising myopathy (NM, n = 59), dermatomyositis (DM, n = 17), polymyositis (PM, n = 19), inclusion body myositis (IBM, n = 22) and non-specific IIM (NSIIM, n = 15) in addition to 18 control samples. Sections were stained for HMGB1, CD68 (macrophages), CD45 (lymphocytes), neonatal myosin heavy chain (nMHC, regenerating myofibres) and LC3 (an autophagic protein) using immunohistochemistry. Slides were independently graded by a muscle pathologist.

Results: Muscle samples from 132 IIM patients with necrotising myopathy (NM, n = 59), dermatomyositis (DM, n = 17), polymyositis (PM, n = 19), inclusion body myositis (IBM, n = 22) and non-specific IIM (NSIIM, n = 15) were analysed, in addition to 18 control samples. Sarcoplasmic HMGB1 was significantly elevated in all IIM subtypes compared with controls (p < 0.001). Structures exhibiting aberrant HMGB1 expression included: infiltrating immune cells, necrotic myofibres, regenerating myocytes and myofibres exhibiting autophagic or mitochondrial dysfunction. Patients with NM and IBM had significantly increased sarcoplasmic HMGB1 compared with DM, PM and NSIIM. In NM, HMGB1 grades highly correlated with the degree of necrosis (Rs 0.74, p < 0.001) and inflammation (CD68: Rs 0.66, p < 0.001; CD45: Rs 0.62, p < 0.001). In IBM, HMGB1 grades highly correlated with regenerating myofibers (Rs 0.81, p < 0.001) and autophagic proteins (Rs 0.85, p = 0.002).

Conclusion: Aberrant sarcoplasmic HMGB1 expression was observed in all forms of IIM, but a notably elevated levels were detected in NM and IBM. Overall, HMGB1 correlated with a number of inflammatory, metabolic, regenerative and destructive processes in the muscle microenvironment, suggesting it plays a complex and multi-faceted role in the pathophysiology of autoimmune muscle disease. The relative balance of these processes within an IIM patient may explain the varying degrees of sarcoplasmic HMGB1 staining we observed across subtypes. Understanding how HMGB1 contributes to the pathogenesis of these complex conditions may lead to novel diagnostic paradigms and therapeutic interventions.

### P77 Identification of heat shock factor 1 (HSF1) as a novel autoantigen in idiopathic inflammatory myopathy

#### Yamei Zhang, Qing-Lin Peng, Han-Bo Yang, Jing-Li Shi, He Chen, Xin Lu, Guochun Wang

##### China-Japan Friendship Hospital, Beijing, China

###### **Correspondence:** Yamei Zhang

Objectives: Antibodies in idiopathic inflammatory myopathy (IIM) were useful for clinical diagnosis and prognostication. We previously identified a novel antibody against heat shock factor 1 (HSF1) in IIM patients, and our study aimed to evaluate disease specificity and clinical significance of it.

Methods: Anti-HSF1 was assayed by ELISA using serum from patients with IIM (n=613), Sjogren’s syndrome (SS, n=40), systemic lupus erythematosus (SLE, n=40), rheumatoid arthritis (RA, n=37) and healthy controls (HC, n=65), and validated by immunoblotting and dot blotting. Immunofluorescence staining was used to detect the expression of HSF1 in IIM patients muscle tissues and C2C12 cells.

Results: Anti-HSF1 could be detected in 65/612 (10.6%) IIM, 4/37 (10.8%) RA, 5/40 (12.5%) SS, 2/40 (5%) SLE and 2/65 (3.1%) HCs. Subgroup analyses in IIM patients revealed that anti-HSF1 was present in 56/489 (11.5%) of DM, 8/112 (7.1%) of PM and 1/11 (9.1%) of myositis-overlap syndrome. Anti-HSF1+ IIM patients had a higher prevalence of TIF1γ positivity (P=0.046), hypergammaglobulinemia (P < 0.001), elevated C-reactive protein (CRP, P=0.039) and erythrocyte sedimentation rate (ESR, P=0.006). Moreover, anti-HSF1 was found associated with higher incidence of malignancy (P=0.008), and intriguingly we found no detectable anti-HSF1 in 27 cancer patients. In addition, multivariate analysis confirmed that anti-HSF1 was associated with an increased risk of malignancy (OR 2.4 [95%CI 1.0-5.9], P=0.05). Cross-sectional study in 226 IIM patients without malignancy found that anti-HSF1+ patients (n=51) had higher PGA VAS (P=0.014) than anti-HSF1- patients (n=175), and anti-HSF1 levels in anti-HSF1+ patients were positively correlated with PGA VAS (r=0.325, P=0.02), constitutional VAS (r=0.312, P=0.026), muscle VAS (r=0.399, P=0.004) and LDH levels (r=0.405, P=0.003). In the longitudinal study involving 10 anti-HSF1+ IIM patients without malignancy, generalized estimating equation (GEE) model confirmed that anti-HSF1 levels were positively correlated with PGA (physician global assessment) VAS (β=2.9, P=0.001), muscle VAS (β=4.3, P < 0.001) and pulmonary VAS (β=4.7, P < 0.001). Furthermore, we found HSF1 protein co-expressed with neural cell adhesion molecule (NCAM) in muscle biopsy specimens of IIM patients. By analyzing the expression of HSF1 protein in different stage of cultured muscle cells, we found HSF1 was expressed by differentiating muscle cells, while no HSF1 expression was observed in myoblasts.

Conclusions: Anti-HSF1 was associated with malignancy and disease activity in IIM, and its target represented a novel autoantigen in IIM.

### P78 Investigating the Specificity of the Myositis Profile-4 EUROLINE assay

#### Clara Ventin-Rodriguez^1^, James B. Lilleker^2^, Shuayb Elkhalifa^2^, Hector Chinoy^2^

##### ^1^Complejo Hospitalario Universitario de A Coruña, A Coruna, Spain; ^2^The University of Manchester, Manchester, UK

###### **Correspondence:** Clara Ventin-Rodriguez; James B. Lilleker

OBJECTIVES: The Myositis Profile 4 EUROLINE immunoblot includes 16 myositis-related autoantibodies (Mi2-alpha, Mi2-beta, TIF1-gamma, MDA5, NXP2, SAE1, KU, PM-Scl75, PM-Scl100, Jo1, SRP, PL-7, PL-12, EJ, OJ, Ro52) and is increasingly used in patients with suspected idiopathic inflammatory myopathy (IIM). Sensitivity is thought to be high, but concerns have been raised about low specificity. We investigated the sensitivity and specificity of antibody positivity for IIM and studied factors associated with true and false positivity.

METHODS: All patients between January of 2016 and July 2018 whom where testing using the Myositis Profile 4 EUROLINE immunoblot were identified. Assay results were matched with clinical data (highest creatine kinase (CK) level, electromyogram, muscle biopsy, pre-test working diagnosis and final diagnosis) collected retrospectively. For patients with suspected IIM, we also calculated the classification probability according to EULAR/ACR criteria.

RESULTS: Of 401 identified patients, clinical data was available for 349. 65% (226/349) were female and the mean age was 54 years (SD 14). Overall, the assay found strong and weak antibody positivity for 23% (79/349) and 19% (66/349) of patients respectively. Pre-test working diagnoses were categorised as IIM (27% 94/349), other connective tissue diseases (CTDs) without suspected muscle involvement (40% 139/349) and myopathic syndromes with low probability of IIM (33%, 116/349). The likelihood of any strong positive result was highest for those with suspected IIM (38% versus 21% and 12% respectively, p< 0.001). Weak positivity was found at a similar rate across the three groups (20%, 20% and 16% respectively, p=0.709). The false positive rate for strong positivity was 15% (40/266). The rate of false positivity was highest for those with a pre-test working diagnosis of other CTDs (20% 28/137) and myopathic syndromes with low probability of IIM (10% 11/110), compared to the suspected IIM group (5% 1/19). Sensitivity and specificity for IIM of any strong antibody positivity was 47% and 85% respectively. Only a pre-test working diagnosis of IIM was associated with increased likelihood of strong antibody positivity (46% (36/79) for the strong positive groups versus 22% (58/270) in the negative group, OR 3.1 (95%CI 1.80-5.20, p< 0.001)), with no associations demonstrated for age, gender, myopathic electromyogram, CK level or inflammatory muscle biopsy.

CONCLUSION: In our cohort, immunoblot testing sensitivity for IIM was moderate and specificity was high. However, false positive results were common, especially in those where the pre-test probability of IIM was low, potentially leasing to unnecessary further investigation in such cases.

### P79 MicroRNA and mRNA profiling in the idiopathic inflammatory myopathies

#### Joanna E. Parkes, Anastasia Thoma, Adam Lightfoot, Philip J. Day, Hector Chinoy, Janine A. Lamb

##### University of Manchester, Manchester, UK

###### **Correspondence:** Joanna E. Parkes

Objectives: The idiopathic inflammatory myopathies (IIMs) are heterogeneous autoimmune conditions of skeletal muscle inflammation and weakness. MicroRNAs (miRNAs) are short, non-coding RNA which regulate gene expression of target mRNAs. The aim of this study was to profile miRNA and mRNA in IIM and identify miRNA-mRNA relationships which may be relevant to disease.

Methods: mRNA and miRNA in whole blood samples from 7 polymyositis (PM), 7 dermatomyositis (DM), 5 inclusion body myositis (IBM) and 5 non-myositis controls was profiled using next generation RNA sequencing. Gene ontology and pathway analyses were performed using GOseq and Ingenuity Pathway Analysis. Dysregulation of miRNAs and opposite dysregulation of predicted target mRNAs in IIM subgroups was validated using RTqPCR and investigated by transfecting human skeletal muscle cells with miRNA mimic.

Results: Overall, 129, 53 and 24 differentially expressed mRNAs were identified for PM, DM and IBM compared to controls, respectively (false discovery rate < 0.05). Analysis of the 5 anti-Jo1 positive samples (4 PM and 1 DM) compared to controls identified 691 differentially expressed genes. In analysis of miRNAs, 4, 4, 7 and 3 miRNAs were significantly differentially expressed for the PM, DM, IBM and anti-Jo1 positive subgroups compared to controls, respectively (p < 0.01). Analysis of differentially expressed genes showed that interferon signalling and anti-viral response pathways were upregulated in PM and DM compared to controls. The anti-Jo1 autoantibody positive subset of PM and DM had more significant upregulation and predicted activation of interferon signalling and highlighted T-helper (Th1 and Th2) cell pathways. In miRNA profiling miR-96-5p was significantly upregulated in PM, DM and the anti-Jo1 positive subset. RTqPCR replicated miR-96-5p upregulation and predicted mRNA target (ADK, CD28 and SLC4A10) downregulation. Transfection of a human skeletal muscle cell line with miR-96-5p mimic resulted in significant downregulation of ADK.

Conclusion: MiRNA and mRNA profiling identified dysregulation of interferon signalling, anti-viral response and T-helper cell pathways, and indicates a possible role for miR-96-5p regulation of ADK in pathogenesis of IIM.

### P80 Alterations in activin A-myostatin-follistatin system associate with disease activity in inflammatory myopathies

#### Lucia Vernerová, Veronika Horváthová, Tereza Kropáčková, Martina Vokurková, Martin Klein, Michal Tomčík, Sabína Oreská, Hana Štorkánová, Herman Mann, Olga Kryštůfková, Jiri Vencovský

##### Institute of Rheumatology, Prague, Czech Republic

###### **Correspondence:** Lucia Vernerová

Objectives: The aim of this study was to investigate myokines involved in muscle atrophy, such as myostatin, follistatin and activin A, in idiopathic inflammatory myopathies (IIM). Recent findings show that rather than myostatin alone, the activin A-myostatin-follistatin system is believed to play an important role in musculoskeletal growth, development and aging. The myostatin biology known by now encourages considering its blocking therapy in IIM as the muscle weakness and atrophy persist in patients even after the suppression of inflammation.

Methods: A total of 94 patients with IIM and 155 healthy controls (HC) were enrolled in the study. Apart from serum samples taken during regular patients’ visits, 20 IIM patients and 21 HC underwent a muscle biopsy. Circulating concentrations of myostatin, follistatin, activin A and TGF-β1 were assessed by ELISA. The expression of myokines and associated genes involved in myostatin signalling pathway in muscle tissue was determined by real-time PCR using TaqMan® Gene Expression Assays.

Results: We report decreased levels of circulating myostatin (2024±1111 vs. 2647±792 pg/ml; p=0.0003) and increased follistatin (1542±564 vs. 1332±670 pg/ml; p=0.008) in IIMs compared to HC. Activin A levels were also higher in IIM (394±142 vs. 334±132 pg/ml; p=0.013) compared to controls while no significant difference was observed for serum TGF-β1. Myostatin was negatively correlated to disease activity measures such as muscle disease activity (muscle DA) (r=-0.289, p=0.015) and manual muscle testing of 8 muscles (MMT8) (r=-0.366, p=0.002). On the other hand, follistatin correlated positively with muscle DA (r=0.235, p=0.047). Gene expression analysis showed higher follistatin (p=0.040) and myostatin inhibitor FSTL3 (p=0.008) levels and lower expression of activin receptor type 1B (ALK4) (p=0.034) and signal transducer SMAD3 (p=0.023) in IIM muscle tissue compared to controls.

Conclusion: The findings of this study contradict the expected pattern of activin A-myostatin-follistatin system in muscle wasting diseases by showing lower myostatin and higher follistatin in circulation and attenuated expression of myostatin pathway signalling components in skeletal muscle of patients with inflammatory myopathies. The activin A-myostatin-follistatin system is altered in myositis either to counterbalance the muscle wasting induced by inflammation, or on the other hand, as a result of prolonged inflammation.

### P81 Differential immune response of skeletal muscle groups in idiopathic inflammatory myopathies

#### Alexander Michels

##### University Hospital Muenster, Münster, Germany

Oobjectives: Idiopathic inflammatory myopathies (IIM) comprise a heterogenic group of immune- mediated diseases leading to progressive destruction of the muscle. IIM subtypes exhibit different patterns of affected muscle groups. In polymyositis, dermatomyositis and immune-mediated necrotizing myopathy proximal muscle groups are affected symmetrically. In contrast, inclusion body myositis is mainly characterized by asymmetric weakness of distal muscle groups. Interestingly, specific cytokine patterns can be found in the different IIM subtypes. In this study we aim to investigate whether differential immunological properties and responses of muscle and endothelial cells from distinct skeletal muscle groups underlie the observed patterns.

Methods: We isolated primary murine skeletal muscle (pmMCs) and microvascular endothelial (pmMMECs) cells from proximal and distal muscle groups using mechanic/enzymatic dissociation and magnetic-activated cell sorting technique. Purified primary cells were cultured and pro-inflammatory, adhesion and MHC molecule expression were analyzed under basal or stimulated conditions (TH1 cytokines: TNFα and IFNγ; Type I interferons: IFNα and IFNβ) via qPCR and flow cytometry. Produced chemokines were detected by LEGENDplex immunoassay.

Results: Under basal conditions, pmMCs of distal muscle groups show enhanced expression levels of pro-inflammatory, adhesion and MHC molecules on their surface. However, pmMMECs of distal muscle groups are characterized by a higher expression of MHC-1. Besides, pmMMECs of the proximal arm display a higher expression of ICAM-1 and VCAM-1 compared to pmMMECs of the distal arm. After stimulation with TNFα and IFNγ pmMCs and pmMMECs of distal muscle groups demonstrate increased expression levels in most of the pro-inflammatory markers, adhesion molecules and MHC complexes. In contrast, an opposed effect of this pattern could be seen at some molecules after a treatment with IFNα and IFNβ: pmMCs of the proximal arm display an enhanced expression of MHC-1 and CD80 compared to the distal arm. In addition, pmMMECs of proximal muscle groups show a higher expression of MHC-1. Furthermore, chemokine secretion by pmMCs and pmMMECs shows distinctive patterns in the different muscle groups (e.g. TARC, KC, Eotaxin, MIP-1α).

Conclusion: The distinct immunological properties in the different muscle groups might explain the specific clinical patterns observed in IIM. However, the regulatory mechanisms and the interaction between the different cell-types still need to be further elucidated. These insights might help to identify new, more specific and urgently needed therapeutic targets.

### P82 Work disability in patients with idiopathic inflammatory myopathies in moldovan myositis group

#### Natalie Loghin-Oprea, Snejana Vetrila, Lucia Mazur-Nicorici, Virginia Salaru, Minodora Mazur

##### State University of Medicine and Pharmacy Nicolae Testemitanu, Chisinau, Moldova

###### **Correspondence:** Natalie Loghin-Oprea

Objectives: To assess the disability and work productivity in patients with with idiopathic inflammatory myopathies

Methods: We performed a cross-sectional study from December 2017 to September 2018, patients included fulfilled the ACR/EULAR classification criteria for IIM. Demographic and clinical data were collected. Consistent with the objective we applied the modified Rankin scale for determining disability. To assess the work productivity, we used Work Productivity and Activity Impairment: General Health (WPAI) questionnaire, that was administered only in employed subjects. The study was performed according to the Declaration of Helsinki for human rights and signed the informed consent. Statistical data was analyzed using MedCalc software version 12.

Results: There were 67 patients enrolled in the study, including 51 females and 16 males with a F:M ratio of 3.2:1, mean age at the time of assessment was 53.1±12.5 (range 25-78) years. The mean disease duration was 8.3±5.3 (range 0.5-12) years. In the study group the disability, according to Rankin scale, was determined as follows: 1st degree – 21 (31.34 %), 2nd degree – 26 (38.81 %), 3rd degree – 11 (16.41 %), 4th degree – 8 (11.94 %), 5th degree was established in one patient. To be noted that 0 and 6th degree was not determined in the study group. The employment status was appreciated in 19 patients: 10 (14.93 %) worked full-time and 9 (13.43 %) part-time, only 4 (5.97 %) patients with disease duration less than 2 years were employed. The mean absenteeism score was 17.06±21.27 (range 0-86.5) %, presenteeism – 26.47±21.20 (range 0-70)%, work productivity loss – 36.19±28.09 (range 1-93.25)% and activity impairment was calculated 28.82±22.33 (range 0-80) percent. We analyzed multiple variables in order to establish which determine loss of productivity and was found moderate correlation between absenteeism and muscle force, r=0.46, p<0.05.

Conclusions: Patients with idiopathic inflammatory myopathies have diverse degree of disability, with majority from slight to moderate. The muscle force impairment seems to be the main cause of reduced work productivity.

### P83 Investigating the Sensitivity and Specificity of the Myositis Profile-4 EUROLINE assay

#### Clara Ventín-Rodríguez^1^, James Lilleker^2^, Shuayb Elkhalifa^3^, Hector Chinoy^3^

##### ^1^Complejo Hospitalario Universitario de A Coruña, A Coruña, Spain; ^2^University of Manchester, Manchester, UK; ^3^Salford Royal Hospital, Salford, UK

###### **Correspondence:** James Lilleker

BACKGROUND: The Myositis Profile-4 EUROLINE immunoblot (EUROIMMUN AG, Germany) includes 16 autoantibodies (Mi2-alpha, Mi2-beta, TIF1-gamma, MDA5, NXP2, SAE1, KU, PM-Scl75, PM-Scl100, Jo1, SRP, PL-7, PL-12, EJ, OJ, Ro52) and is increasingly used in the workup of patients with suspected idiopathic inflammatory myopathy (IIM). Sensitivity is high, but concerns have been raised about low specificity for IIM. We investigated the sensitivity and specificity of antibody positivity for IIM using this assay.

METHODS: All patients tested using the Myositis Profile-4 EUROLINE immunoblot between January of 2016 and July 2018 at a UK hospital were identified. Assay results were matched to clinical data (highest creatine kinase [CK] level, electromyogram, muscle biopsy, pre-test working diagnosis and final diagnosis) collected retrospectively.

RESULTS: Of 401 identified patients, accompanying clinical data was available for 349 (87%). 65% (226/349) were female and the mean age was 54 years (SD 14). Strong and weak antibody positivity was found for 23% (79/349) and 19% (66/349) of patients respectively. Dual strong specificities were found in 5% (18/349). Pre-test working diagnoses were categorised as "IIM" (27%, 94/349), "other connective tissue diseases (CTDs) without suspected muscle involvement" (40%, 139/349) and "myopathic syndromes with low probability of IIM" (33%, 116/349). The likelihood of any strong positive result was highest for those with suspected IIM (38%, versus 21% and 12% respectively, p < 0.001). Weak positivity was found at a similar rate across the three groups (20%, 20% and 16% respectively, p=0.709). The false positive rate for strong antibody positivity was 15% (40/266) overall, highest for those with a pre-test working diagnosis of CTDs without suspected muscle involvement (20%, 28/137) and myopathic syndromes with low probability of IIM (10%, 11/110), compared to 5% (1/19) for those with a pretest diagnosis of IIM. Sensitivity and specificity for IIM of any strong antibody positivity was 47% and 85% respectively. Only a pre-test working diagnosis of IIM was associated with increased likelihood of strong antibody positivity (46% (36/79), versus 22% (58/270) in the antibody negative group, OR 3.1 (95%CI 1.80-5.20, p < 0.001)), with no associations demonstrated for age, gender, myopathic electromyogram, CK level or inflammatory muscle biopsy.

CONCLUSION: Strong antibody positivity using this EUROLINE immunoblot showed moderate sensitivity and high specificity for IIM. False strong positive results were common especially in those where the pre-test probability of IIM was low, potentially leading to unnecessary further investigation in such cases.

### P84 A longitudinal analysis of anti-FHL1 antibodies in a Swedish cohort of patients with Idiopathic Inflammatory Myopathies

#### Angeles Shunashy Galindo-Feria^1^, Eliska Divišová^2^, Cátia Fernandes-Cerqueira^1^, Maryam Dastmalchi^1^, Edvard Wigren^1^, Susanne Gräslund^1^, Ingrid. E. Lundberg^1^

##### ^1^Karolinska Institutet, Stockholm, Sweden; ^2^Charles University, Prague, Czech Republic

###### **Correspondence:** Angeles Shunashy Galindo-Feria

Objectives: Antibodies targeting a novel and muscle-specific autoantigen, the Four-and-a-half-LIM-domain 1 (anti-FHL1), have been identified in patients with idiopathic inflammatory myopathies (IIM). The aim of this project was to evaluate when anti-FHL1 autoantibodies are present in the course of the disease and if autoantibody titers vary over time.

Methods: The anti-FHL1 antibody status was obtained from a previous serological cross-sectional analysis from where we selected sera from IIM anti-FHL1+ patients (n=25) and sex and age matched sera from IIM anti-FHL1- patients (n=51), and healthy controls (HCs, n=50). Levels of anti-FHL1+ autoantibodies were evaluated by ELISA. Patients included in the study had at least one sample available at time of diagnosis and one consecutive serum sample within an interval of 36 months. All patients were followed at the Division of Rheumatology, Karolinska University Hospital from January 1982 to December 2017. HCs sera collected at a single time point were tested.

Results: In the IIM group we included 76 patients with a total of 320 serum samples. Median follow-up time was 108 months, with median of 4 samples available per patient. In total, we identified the presence of anti-FHL1+ antibodies in n=31 IIM patients, corresponding to the group of anti-FHL1+ (n=25) and in anti-FHL1- (n=6) from the cross-sectional analyses. One HC had positive anti-FHL1 titers. We subdivided the patients into 4 groups: anti-FHL1+: “highly positive” (O.D.>1, n=11), “intermediate positive” (1>O.D.>0.75, n=6), “baseline negative” that seroconverted (O.D.>0.75, n= 14), and one anti-FHL1 “negative” group. All groups were compared by given OD medians at each of the point of observation. Highly positive patients had persisting high anti-FHL1 titers during the follow-up; on the contrary, both intermediate positive and baseline negative groups were fluctuating around the cut-off point. Eight of 14 (57%) patients from the group “baseline negative” seroconverted to anti-FHL1+ within the first 36 months. The anti-FHL1 “negative” group presented constant low antibody titers during the longitudinal follow-up.

Conclusions: Anti-FHL1 antibody positivity was detected in patients with high titers of anti-FHL1+ autoantibodies in the first available serum sample or developed within the first 36 months after diagnosis and persisted over many years. A group of patients with intermediate levels had fluctuating positivity over the years. It is still unknown if the anti-FHL1 antibody titer is a marker of prognosis and/or damage in IIM; thus, clinical data needs to be addressed, and in-vitro experiments and biochemical characterization of this autoantibody are still required.

### P85 Shear Wave Elastography; Preliminary Validation and Diagnostic Utility in the Evaluation of Muscle

#### Shereen Paramalingam, Helen Keen, Merrilee Needham

##### Fiona Stanley Hospital, Murdoch, Australia

###### **Correspondence:** Shereen Paramalingam

Background: Idiopathic inflammatory myopathies (IIM) is a rare, heterogeneous group of acquired muscle diseases characterised by progressive muscle weakness. Shear wave elastography (SWE) is a novel ultrasound (US) technique that uses acoustic radiation force impulse (ARFI) to generate a shear wave which estimates shear wave speed (SWS) and ultimately tissue elasticity of muscle. There is currently no standardised methods to evaluate muscle using SWE.

Objective: The objective of this study is to begin validation of SWE as an outcome tool in muscle disease.

Methodology: This is a pilot cross-sectional study looking at an incident group of IIM (n=10) and healthy controls (n=30) using US (grey scale B mode and power Doppler) and SWE in the evaluation of muscle. All patients (n=40) will receive US (grey scale B mode and power Doppler) and SWE over the deltoid and vastus lateralis and just US (grey scale B mode and power Doppler) over the flexor digitorium profundus, flexor carpi ulnaris, tibialis anterior and lateral gastrocnemius muscle. All patients will have routine clinical assessments- Manual Muscle Testing (MMT8 and MMT26), patient and physician Visual Analogue Scale (VAS), Health Assessment Questionnaire (HAQ) score, laboratory assessments (full blood picture (FBC), urea and creatinine (U&E), alanine aminotransferase (ALT), creatine kinase (CK), erythrocyte sedimentation rate (ESR) and C-reactive protein (CRP) and an additional myositis serology, antinuclear antibody (ANA), extractable nuclear antigen (ENA) and HMG CoA Reductase enzyme for the IIM group. The incident (n=10) IIM group will have clinically indicated magnetic resonance imaging (MRI) that will be used as a comparator to SWE. The initial data analysis is descriptive, with non-standardly distributed data being analysed with non- parametric tests. Linear regression analysis will be used to determine the association between SWS and other commonly used measures of muscle disease. Logistic regression will be used to determine the sensitivity and specificity of SWS in distinguishing between diseased and control participants. Results: Pending, to be finalised prior to GCOM 2019

Conclusion: SWE may have a role in both the research and clinical setting when managing patients with IIM

### P86 Comparing muscle ultrasound to whole body muscle magnetic resonance imaging to as biomarker of disease activity in patients with new-onset myositis: a prospective cohort study

#### Johan Lim^1^, Frank Smithuis^1^, Esther Brusse^2^, Jessica Hoogendijk^3^, Christiaan Saris^4^, Marije Wolvers^1^, Rob De Haan^1^, Eleonora Aronica^1^, Mario Maas^1^, Marianne De Visser^1^, Filip Eftimov^1^, Camiel Verhamme^1^, Anneke van der Kooi^1^

##### ^1^University of Amsterdam, Amsterdam, Netherlands; ^2^Erasmus MC, Rotterdam, Netherlands; ^3^UMC Utrecht, Utrcht, Netherlands; ^4^Radboud University, Nijmegen, Netherlands

###### **Correspondence:** Johan Lim

Objective: Biomarkers of disease activity in myositis lack varying degrees of diagnostic accuracy, which may consequently lead to both under and overtreatment. Muscle ultrasound (US) and muscle Magnetic Resonance Imaging (MRI) are two non-invasive biomarkers used in clinical practice, however, no direct comparisons have been made. Therefore, we conducted this observational study to compare US to MRI as diagnostic biomarkers and as marker of disease activity during follow-up in new-onset patients with myositis.

Methods: Cohort of myositis patients (excluding inclusion body myositis) who were included in the ongoing IMMEDIATE study (NTR6160). All patients were clinically assessed before and after nine weeks of IVIg monotherapy by the IMACS Core Set Measures (CSMs) and Total Improvement Score (TIS). US (Esaote SpA, Genoa, Italy) consisted amongst others of quantitative gray-scale analysis of muscle echointensity (EI) and quantitative analysis of subcutis, fascia, and muscle diameter. Whole body MRI (WB-MRI) (Philips, Best, The Netherlands) consisted amongst others of 2-point Dixon (3 Tesla) generated coronal and axial T2-weighted fat-suppression 2D images in which 42 muscles and 8 fascia/subcutaneous locations were semi-quantitatively scored for edema.

Results: So far, we included 16 patients: dermatomyositis (n=8), non-specific myositis/overlap myositis (n=3), immune-mediated necrotizing myopathy (n=4), and anti-synthetase syndrome (n=1). We intend to include four other patients. We will present results of baseline assessment of US and MRI and of changes over time after nine weeks of IVIg therapy. We will explore discriminating validity of various imaging parameters between responders and non-responders, and explore correlations with clinical parameters (amongst others IMACS CSMs).

Conclusion: This study may show the usefulness of ultrasound and MRI as biomarkers of disease activity in new-onset patients with myositis.

### P87 May the muscular MRI identify a pattern of muscular involvement in patients with idiopathic inflammatory myopathies? Analysis of a monocentric cohort

#### Simone Barsotti, Mugellini Barbara, Alessandra Tripoli, Giacomo Aringhieri, Chiara Cardelli, Elisa Cioffi, Virna Zampa, Davide Caramella, Marta Mosca, Rosella Neri

##### University of Pisa, Pisa, Italy

###### **Correspondence:** Simone Barsotti

Objective: In patients with idiopathic inflammatory myopathies (IIM), magnetic resonance imaging (MRI) has been proposed as a useful tool for both the diagnosis and the follow-up, but the clinical significance and the pattern of muscular involvement in IIM patients still remains undefined. The primary aim of this study is to analyse the presence of MRI alterations (muscular edema, fatty infiltrates and atrophy) and their correlation with clinical parameters and to evaluate the possible difference in the pattern of muscular alterations between disease subsets in IIM patients.

Methods: We retrospectively collected data from IIM patients (EULAR/ACR criteria) who performed a MRI of pelvic and thigh muscles from 2010 to 2017: 27 had dermatomyositis and 58 polymyositis, mean age of 58.6±13.4 years and mean disease duration of 45±73 months. The images were assessed by a dedicated radiologist in 23 muscles for the presence of muscular edema, fatty infiltrates and atrophy. Moreover, data about serum creatine kinase (CK) and manual muscle test 8 (MMT8) were collected. Data were reported as median±interquartile range, non-parametric tests were used for the analysis.

Results: Sixty-three patients presented muscular edema in at least one muscle. The muscular edema was higher in patients with lower disease duration (9.5±47.0 vs 19.8±97.2 p=0.031). Patients with muscular edema had higher CK levels (1506±1976 vs 235±224 p=0.001) and lower MMT8 (63.6±11.2 vs 69.8±10.7 p=0.031). Forty-eight patients presented fatty infiltrates that were more present in older patients (p=0.01) and in those with longer disease duration (39.1±95.4 vs 9.5±12.5 p=0.024). At multivariate analysis disease duration represented the only independent factor for the presence of muscular fatty infiltrates (p=0.05). Muscular atrophy was present in 17 patients but it was not correlated to age and disease duration. CK and MMT8 were not different in presence/absence of fatty infiltrates or muscular atrophy. Edema and atrophy were not different between poly- and dermatomyositis, while fatty infiltrates were more present in the posterior compartment (biceps femoris and semitendinosus muscle) in patients with PM compared to DM (68.2% vs 29.6% p=0.046).

Conclusions: The alterations identified with MRI are different according to disease duration and the pattern of the fatty infiltration, but not of the inflammation, may present a different distribution between patients with dermatomyositis and polymyositis. Despite the use of MRI for patients with IIM may provide additional information for clinical and biochemical examination, the role for the differential diagnosis and in the follow-up needs to be confirmed.

### P88 Recovering autonomy is a key advantage of home-based immunoglobulin therapy in patients with myositis: a qualitative research study

#### Patrick Chérin^1^; Taylor Pindi^2^; Pierre Clerson^3^; Annaik Dokhan^4^; Yann Fardini^3^; Martin Duracinsky^5^; Jean-Chrales Crave^2^; Olivier Chassany^5^

##### ^1^Pitié-Salpetrière Hospital, Paris, France; ^2^Octapharma, Boulogne-Billancourt, France; ^3^Soladis Clinical Studies, Roubaix, France; ^4^Sirius Customizer, Paris, France; ^5^University Paris-Diderot, Paris, France

###### **Correspondence:** Taylor Pindi

Purpose: Immunoglobulins are second or third-line treatments in dermatomyositis (DM) or polymyositis (PM) refractory to high-dose corticosteroids and immunosuppressants. Immunoglobulins (2g/kg/month) are usually administered intravenously (IVIg) once a month and the patient have to stay at hospital for a few days. Recently, subcutaneous injections (SCIg) have been proposed 2-3 times per week, in some dysimmune diseases. SCIg are administered at home either by the patient himself or by a nurse. We investigated the needs and attitudes of DM and PM patients having the experience of IVIg and SCIg. Patients and methods: Seven patients (6 PM and 1 DM) from a single centre participated in a focus group (N=6) or underwent in-depth interview (N=1). Five patients had the experience of both IVIg at hospital and SCIg at home; one patient has received only IVIg at hospital. Verbatim was recorded and transcribed for further content analysis and computer-aided textual analysis.

Results: Clinical profiles and stories were heterogeneous. At diagnosis, muscle weakness but also severe pain and fatigue were at the forefront of patients’ complaints impairing daily life and autonomy. Patients reported to be considerably improved with immunoglobulins. All patients with experiences of both IVIg and SCIg expressed their preference for SCIg which were described as easy, less disruptive for daily life, well tolerated and less time-consuming. SCIg self-administration at home restored their feeling of autonomy and control. Patients appreciated to be considered actors of their own treatment.

Conclusion: In-depth interviews of DM and PM patients revealed that recovering autonomy and control was a central advantage of home-based SCIg. Home-based SCIg were efficient, well-tolerated and perceived as a good compromise between treatment burden and efficacy.

Keywords: In-depth interviews, focus group, textual analysis, myositis, patients expectations, preference, immunoglobulins, autonomy

### P89 Muscle Function and Health-Related Quality of Life in Patients with Polymyositis and Dermatomyositis

#### Kristofer Andreasson, Li Alemo Munters, Helene Alexanderson

##### Karolinska University Hospital, Stockholm, Sweden

###### **Correspondence:** Kristofer Andreasson

Objective: The objective is to investigate muscle function in terms of maximal voluntary isometric strength (MVIC) and isometric muscular endurance (ME) in patients with newly onset and established PM/DM. A further objective is to investigate if there is a correlation between muscle function and health related quality of life (HRQoL).

Methods: Patients diagnosed with PM or DM (n=8) during September 2017 and September 2018 at Karolinska University hospital in Solna who met the inclusion criteria were asked to participate. Patients with established PM/DM (n=7) where identified through patient register at the same clinic, selected to match the patients with recent onset disease as to age and gender. To asses MVIC and ME, a Biodex dynamometer was used. Three maximum, 4-second-contractions, with a three-minute rest in-between were performed followed by six sets of twelve submaximal contractions ranging from 20 % to 70 % of MVIC. Each set ended with a new maximum repetition registered as percentage of MVIC. SF-36 was used to measure HRQoL. Statistical significance was set to p < 0.05, Mann-Whitney U-test was used to calculate group differences and Spearman’s rho for correlations.

Results: Median age for patients with recent onset PM/DM was 34 (range19–64) years and for established disease 58 (28–66) years. Diagnosis duration was 5.5 (1–6) months and 7.4 (6–36) years for the two groups, respectively. Patients with recent onset disease had a median MVIC of 73 (45–127) Nm and patients with established disease had 70 (41–118) Nm (p=0.46). Patients with recent onset disease and established PM/DM had ME 83 (63–94) % and 91 (85–98) %, respectively (p=0.01). Moderate correlations were found between ME and Vitality (rs=0.56, CI (0.07;0.84) and ME and Physical Role function (rs=0.54, CI (0.03;0.82). There were weak correlations between ME and Physical Function, General Health and Social Function varying between (rs=0.41, CI (-0.13;0.76) and (rs=0.49, CI (-0.03;0.8). A negative and weak correlation between MVIC and Role Emotional was found (rs=-0.3, CI (-0.71;0.25) with even lower correlations to remaining domains.

Conclusion: Patients with recent onset PM/DM seem to have reduced ME compared to patients with established disease. No differences were found regarding maximal voluntary isometric contraction (MVIC). Moderate correlations were found between ME and Vitality and Physical Role function. Other correlations were weak at best. More patients need to be included to reach statistical power to confirm these preliminary results.

### P90 Living with idiopathic inflammatory myopathies – patients’ thoughts, fears, and expectations

#### Hedvig Engberg, Helene Alexandersson, Marie Holmqvist

##### Karolinska Institutet, Stockholm, Sweden

###### **Correspondence:** Marie Holmqvist

Background: Being diagnosed with idiopathic inflammatory myopathies (IIM) alters your life. Little is know of how patients with IIM experienced their period of diagnosis, what they think we should focus on Our aim was therefore to understand the self-experienced difficulties patients with IIM face, to gain a deeper knowledge of the experiences with health care patients with IIM have, and to find out what patients with IIM would like health care and researchers to focus on continuing forward.

Methods: To answer these questions we asked 12 patients with IIM who were followed at the division of rheumatology for consent to interview them. Patients with overlap disease were excluded from participation. Semi-structured interviews were conducted at patients most convenient location, which often was in their home or at the division of rheumatology. The interviews were conducted by a trained medical student, and all interviews were recorded, transcribed and coded by three of the authors. A thematic analysis was performed.

Results: Twelve patients were interviewed between January and February 2018. Median age was 64 years of age at interview (range 45-85), 9 were women, 3 were men. They had a median disease duration of 11 years (range 2-37 years). The results from our interviews were primarily x: 1. we found that the time period up until diagnosis was very emotionally excruciating. Patients had for a long time felt something was not right, and had several contacts with health care facilities, before somebody realized what was the underlying problem. Not being believed was reported to be difficult. 2. Patients need more disease specific information than we are giving them. They also requested more and detailed information about follow up and prognosis of their disease. Further, it was also expressed that being able to discuss their entire life-situation with a physician, specialized in IIM, was of pivotal importance. Being diagnosed and seriously ill at the time of diagnosis also conferred a traumatic experience patients wanted to talk about. Unfortunately, patients also reported that the diagnosis has lead to isolation for them. Their fear of infections and a wish to remain “who they had always been” made patients refrain from socializing with friends and family.

Conclusion: We need to focus more on the holistic care of these patients. Many have traumatic experiences and wish to discuss their lives with their physician.

### P91 Persons with polymyositis and dermatomyositis experience reduced work ability and quality of life

#### Malin Regardt, Karin Åström

##### Karolinska University Hospital, Stockholm, Sweden

###### **Correspondence:** Malin Regardt

Objective: To describe self-rated work ability with two different assessment and quality of life in persons with PM and DM. To investigate correlations between self-rated work ability and quality of life.

Method: Participants were identified through the Swedish Myositis Network registry (SweMyoNet). Of 78 possible participants, 48 agreed to participate in this study. The median (IQR) age were 57 (45-61) years with a median disease duration of 6 (2-14) years. Fifty-three percent of the participants were women. Seventy-seven percent were working, and the remaining were on sick-leave. Self-rated work ability was measured by the questionnaire Work Ability Index (WAI) and the Work Ability Score (WAS) which is a single item question. Quality of life by the SF-36.

Results: Self-rated work ability measured by WAI in persons with PM and DM varied between poor work ability and good work ability. The median value of the group was 34 which indicates less good work ability. Self-rated work ability measured by WAS varied between poor work ability and good work ability. The median of the total group indicates less good work ability. There was a strong correlation between self-rated work ability measured by WAI and WAS (rs 0,879 p = 0,01). Quality of life measured by SF-36 were rated lower in persons with PM and DM when compared to the general population in dimensions; Physical Function, Role-Physical, General Health, Vitality and Social function (p ≤ 0.02). There were moderate to high correlations between self-rated work ability measured by both WAI and WAS, and all dimensions of SF-36 (p < 0.01)

Conclusion: Persons with PM and DM self-rated their work ability as poor and the quality of life were significantly reduced when compared to the general population. The measures WAI and WAS correlates highly with each other and revealed comparable results indicating that WAS may work as well as WAI as a screening tool to identify reduced work ability. The WAS may also be more feasible with just one single question to use in clinical practice. Our results indicate the importance to measure self-rated work ability in persons with PM and DM in clinical practice.

### P92 Female Sexual Function Impairment in Idiopathic Inflammatory Myopathies

#### Barbora Heřmánková, Maja Spiritovic, Sabina Oreska, Hana Storkanova, Karel Pavelka, Ladislav Senolt, Herman Mann, Jiri Vencovsky, Michal Tomcik

##### Institute of Rheumatology, Prague, Czech Republic

###### **Correspondence:** Barbora Heřmánková

Objectives: Idiopathic inflammatory myopathies (IIM) are characterized by chronic muscle inflammation and involvement of internal organs which leads to functional impairment, reduced quality of life including sexual life. The aim of this study was to assess sexual functions/quality of life and pelvic floor function in female IIM patients compared to age-/sex-matched healthy controls (HC) and to analyze the potential impact of disease activity, fatigue, physical activity and depression.

Methods: In total, 22 women with IIM [mean age: 55.1, disease duration: 7.9 years, dermatomyositis (DM, 8)/ polymyositis (PM, 10)/ necrotizing myopathy (IMNM, 3)/ inclusion body myositis (IBM, 1)], who fulfilled the Bohan/Peter 1975 criteria for DM/PM, and 22 healthy controls (mean age: 55.1 years) filled in 12 well-established and validated questionnaires assessing sexual function and pelvic floor function, fatigue, physical activity and depression. We used the following questionnaires: Female Sexual Function Index (FSFI), Brief Index of Sexual Function for Women (BISF-W), Sexual Quality of Life Questionnaire (SQoL-F), Pelvic Organ Prolapse/Urinary Incontinence Sexual Questionnaire (PISQ-12), Pelvic Floor Distress Inventory Questionnaire (PFIQ7), Fatigue Impact Scale (FIS), Beck’s Depression Inventory II (BDI II), Health Assessment Questionnaire (HAQ) and Human Activity Profile (HAP).

Results: Compared to HC, patients with IIM had significantly higher prevalence and greater severity of sexual dysfunction (FSFI, BISF-W: in all subscales as well as total scores), dysfunction of pelvic floor (PISQ-12), and worse sexual quality of life (SQoL-F). Worse scores in IIM patients were associated with elevated muscle enzyme levels [lactate dehydrogenase: FSFI (r=-0.524,p=0.0123), BISFW (r=-0.528,p=0.0115)], greater fatigue [FIS: FSFI (r=-0.434,p=0.0438), BISF-W (r=-0.488, p=0.0211), SQoL-F (r=-0.488,p=0.0070), PISQ-12 (r=0.643,p=0.0013)], more severe depression [BDI-II: PISQ-12 (r=0.474,p=0,0258)], deteriorated quality of life [HAQ: PISQ-12 (r=0.476,p=0.0252)], and worse ability to perform physical activities [HAP: FSFI (r=0.437,p=0.0417), BISF-W (r=0.451,p=0.0351), PISQ-12 (r=-0.494,p=0.0195)].

Conclusions: Women with IIM reported significantly impaired sexual function, sexual quality of life and pelvic floor function compared to age-matched healthy controls. Worse scores in IIM were associated with disease activity, physical activity, fatigue, depression and quality of life. Acknowledgements: Supported by AZV-16-33574A, MHCR 023728, and SVV – 260373.

### P93 Experience of pain in polymyositis and dermatomyositis – a qualitative study

#### Helene Alexanderson, Masoumeh Tasarrofi, Malin Regardt

##### Karolinska University Hospital, Stockholm, Sweden

###### **Correspondence:** Helene Alexanderson

Objective: Studies indicate that adults with polymyositis (PM) and dermatomyositis (DM) have higher self-reported pain compared to general population assessed by the SF-36 domain, Bodily pain (BP). International focus groups and online surveys indicate that pain is an important symptom in PM/DM. Knowledge on myositis-related pain is limited. The objective was to explore experience of pain in adults with PM and DM.

Methods: Patients with adult PM/DM were strategically identified to represent genders, various ages, self-reported pain, diagnosis duration, and disease activity. Inclusion criteria; age > 18 years, VAS pain >10 mm. Exclusion criteria; diagnosis of fibromyalgia or other inflammatory rheumatic disease. Individual interviews using a semi-structured interview guide were conducted. Patients completed the SF-36 survey after the interview. Interviews were audiotaped and transcribed verbatim. Qualitative content analysis was performed by two researchers separately followed by a consensus discussion.

Results: Six patients were included; female n= 4, PM n=4, median (range) age 44.5 (28-71), diagnosis duration 5 (1-22) years, physician global VAS 15 (0-40) mm, pain VAS 66 (30-84) mm. SF-36 BP was 31.5 (22-74), more pain compared to reference value of 70. Three over-arching THEMES, 12 categories and 21 subcategories (subcat) were identified. Theme 1: PAIN WAS WORST IN THE BEGINNING, WAS REDUCED BY CORTICOSTEROIDS, BUT RETURNED WITH TAPERING OF MEDICATION TO SUSTAINED, EVEN WORSENING LEVELS OVER TIME, Category-Factors increasing the pain, (subcat/over exertion, tapering of corticosteroids, cold climate), Category-Factors reducing pain, (subcat/warm climate, medication/adapted exercise, self-management), Category-Localization and character of pain, (subcat/pain was first myositis symptom, pain in muscles and joints, constant pain), Category-Thoughts about pain, (subcat/myositis causes pain, pain is a subjective symptom, fatigue have worse life-impact than pain). Theme 2: INFORMATION ABOUT PAIN IS IMPORTANT, Category-Information from health-care providers (HCP), (subcat/inadequate and wrong information, HCP don’t care about how pain impacts sex-life). Theme 3: PAIN INFLUENCES QUALITY OF LIFE, Category-Social life, (subcat/reaction from family, friends and colleagues, pain hinders social activities), Category-Emotions, (subcat/don’t want to show or talk about my pain, anger, grief and frustration, relief by other’s support), Category-Pain impact on daily activities, (subcat/pain impacts sleep, pain limits daily activities).

Conclusion: Pain seems to be a myositis symptom and could be the first symptom of myositis, could initially be reduced by corticosteroids but worsens with tapering of medication and remain as a chronic symptom worsening over time. HCP need to ask about, measure and give correct information about myositis-related pain.

### P94 A novel autoantibody against DNA damage binding protein-1 in idiopathic inflammatory myopathy

#### Yuji Hosono

##### National Institutes of Health, Bethesda, USA

Objective: Many kinds of myositis specific autoantibodies (MSAs) are detected in idiopathic inflammatory myopathy (IIM) patients. These are useful to diagnose, predict the clinical course, and assess therapy in IIM at an early stage. Here we describe and characterize the clinical significance of a novel autoantibody in IIM directed against the DNA damage binding protein-1 (DDB-1).

Methods: 380 patients with various connective tissue diseases (CTDs) and 20 healthy controls (HCs) were screened for autoantibodies by immunoprecipitation with [35S] methionine-labeled HeLa cells. The target autoantigen was immunoaffinity-purified from HeLa cell extracts and was subsequently identified by peptide mass fingerprinting. Antigen specificity of the serum was further examined by immunoblotting.

Results: An antibody directed against a 120kDa protein was detected in sera from 6 patients with IIM, but not in the sera from other CTD patients or HCs. No patient with anti-120kDa antibody was positive for other myositis-specific autoantibodies. 83% (5 of 6) were polymyositis and 17% (1 of 6) was DM. None with anti-120kDa antibody had complicated cancer. All except 1 (83%, 5 of 6) were negative for antinuclear antibody. Most anti-DDB-1 positive patients (67%, 4 of 6) showed spontaneous improvements without immunosuppressant treatment. Peptide mass fingerprinting and immunoblotting identified DDB-1 as the corresponding autoantigen recognized by anti-120KDa autoantibody.

Conclusions: The anti 120kDa antibody which recognized DDB-1 was a novel marker of IIM. Anti-DDB-1 antibody detection may be helpful for diagnose and have prognostic and therapeutic implications in patients without known MSAs. Our findings may shed new insights into the pathogenesis of IIM.

### P95 Evaluation of the effect of neutralizing anti-IFN-α antibodies produced in one SLE patient vaccinated with IFN-K on myotubes atrophy induced by type I interferon

#### Alexandrine Mahoudeau; Leandro Ladislau; Yves Allenbach; Thérèse Croughs; Allia Gati; Céline Anquetil; Damien Amelin; Olivier Benveniste

##### ., .

###### **Correspondence:** Alexandrine Mahoudeau

Dermatomyositis is an acquired auto-immune disease characterized by skin or muscle lesions and muscle-specific pathological features such as perifascicular muscle fibre atrophy and vasculopathy. Dermatomyositis patients display an upregulation of type I interferon-inducible genes in muscle fibres, endothelial cells, skin, and peripheral blood. We have shown in vitro that the activation of type I interferon in differentiating myoblasts abolished myotube formation with reduced myogenin expression while in differentiated myotubes, we observed a reduction in surface area and an upregulation of atrophy-associated genes (L Ladislau et al. Brain 2018; 141(6):1609-1621). Here, our aim was to evaluate in the same in vitro system, the effect of polyclonal human anti-IFN-α neutralizing antibodies (referred to as anti-IFN-α patient Abs) induced in a lupus patient treated with an immunotherapeutic vaccine interferon-α-kinoid (IFN-K, Neovacs, NCT01058343-Phase I/IIa study-IFN-K-001, 4 injections of IFN-K at 120 μg, BR Lauwerys et al. Arthritis Rheum. 2013;65(2):447-56). Myotubes were treated with recombinant human interferon alpha 2b (IFN-α, 103 U/mL), and with monoclonal anti-human interferon alpha (anti-IFN-α mAb, Clone MMHA-11), or purified mouse IgG1 isotype control (ISO), or anti-IFN-α patient Abs corresponding to total purified IgG from serum of the patient vaccinated with IFN-K (50μL at 1 mg/mL, total IgG purified with NabTM Protein G Spin Kit, Thermo Scientific). To assess the effect of IFN-I pathway activation on myotube surface area, myotubes were immunostained with anti-myosin heavy chain (hybridome, clone MF20) and total surface area was evaluated using the Cell Profiler® software and ImageJ® software (L Ladislau et al. Brain 2018; 141(6):1609-1621). As expected, an atrophy of the myotubes incubated with IFN-α+ ISO (262542 μm²) compared to untreated myotubes (ISO, 448338μm², p< 0.001) was observed. This atrophic effect was not observed when anti-IFN-α mAb, or anti-IFN-a patient Abs were added to the media (434389 μm² p=0.9; 423360 μm² p=0.6, respectively). Anti-IFN-α patient Abs induced after in vivo vaccination by IFN-K in lupus patients have a neutralizing effect on the in vitro bioeffect of IFN-α on myotubes. This observation paves the way of IFN-K as a potential therapeutic approach for dermatomyositis patients.

### P96 Patients with inflammatory myopathies admitted in intensive care unit are characterised by recent onset and untreated active disease as well as older age and high comorbidities

#### Baptiste Michard, Thierry Artzner, Francois Severac, Julien Pottecher, Jean Sibilia, Bernard Geny, Alain Meyer^1^

##### CHU de Strasbourg, Strasbourg, France

###### **Correspondence:** Alain Meyer

Background: The characteristics of adults patients with inflammatory myopathies (IM) admitted to intensive-care unit (ICU) have not yet been assessed.

Objectives: To assess the clinical features, risk-factors and outcome of patients with IM admitted in ICU.

Methods: A single-centre cohort of 509 patients with IM was screened for admission in ICU from 1992 to 2017. Control-patients with IM who had not been hospitalised in ICU were randomly selected.

Results: 32 ICU-admissions were recorded in 27 IM-patients. Characteristics and prognosis of IM-patients in ICU. Patients hospitalised in ICU had a mean age of 63±15 years with SAPS II score of 58±24 and LODS score of 9±5 corresponding to an intermediate severity at admission in ICU. The delay between IM diagnosis and first ICU-admission was 27±43 months. Twelve patients (44%) were admitted within the first month of IM diagnosis, among whom 4 (15%) were diagnosed with IM during ICU stay. Sixteen patients (60%) were not treated at the time of their first ICU-admission. In 56% of the ICU stays, patients had active disease at admission. Patients were most frequently admitted for respiratory failure (88%) but cardiac (47%), renal (47%), neurologic (47%), haematological (22%) and hepatic (15%) failures were also recorded. Infections were present in 72% of the ICU stays. Nine patients (33%) died in ICU and 3 others (11%) within 90 days of the last ICU discharge (vs. 15% during a 7.5±5 years period of follow-up in the control group, p < 0.0001). Risk-factors of ICU-admission. Three risk-factors independently associated with ICU-admission were highlighted: higher age at IM onset (62±13 vs. 53±13 years, p < 0.05), higher rate of chronic kidney failure (26% vs. 0%, p < 0.05) and higher incidence of arterial/venous thrombosis history (37% vs. 0%, p < 0.05). Other risk-factors identified only in univariate analysis included lower BMI (22.6±4.5 vs. 25.4±6.3), a history of interstitial lung disease (48% vs. 30%) and a higher Charlson comorbidity index (4.6±2.6 vs. 3.3±2). The type of IM was not significantly associated with ICU-admission, although no patient admitted in ICU had sIBM. It is noteworthy that cumulative number of immunomodulatory treatments in patients at the time of ICU-admission was lower than in the control group (0.5±0.7 vs. 1.9±0.8 p < 0.001).

Conclusions: IM-patients admitted in ICU frequently have recent onset and untreated active IM with respiratory failure. ICU-admission is associated with older age and a higher number of comorbidities. Mortality is high.

### P97 Comparison of autoantibody specificities tested by a line blot assay and immunoprecipitation-based algorithm in patients with idiopathic inflammatory myopathies

#### Fabricio Espinosa-Ortega, Marie Holmqvist, Helene Alexanderson, Helena Storfors, Tsuneyo Mimori, Ingrid Lundberg, Johan Rönnelid

##### Karolinska University Hospital, Stokholm, Sweden

###### **Correspondence:** Fabricio Espinosa-Ortega

To date, there is no standardized approach of myositis specific autoantibodies/Myositis associated autoantibodies (MSA/MAA) testing to be used in clinical settings. Immunoprecipitation (IP) of RNA’s with silver staining and/or protein IP on cellular lysates is considered the gold standard for most autoantibodies but is time-consuming, does not differentiate antibodies targeting proteins with the same molecular weight, and does not measure levels of autoantibodies and is not routinely available in clinical settings. Line blot assays (LB) represent a faster and semi-quantitative option to detect autoantibodies. Objectives We aimed to compare the performance of a line blot (LB) and immunoprecipitation (IP) assays and to describe clinical associations to autoantibody specificities in a cohort of patients classified as idiopathic inflammatory myopathies (IIM). 119 patients were tested by a commercial LB (Euroline Myositis Antigen Profile 4, Euroimmun, Germany) and a combination of protein- and RNA- IP together with an anti-MDA5 ELISA. Agreement between assays was calculated by Cohen´s kappa coefficient. Results The overall concordance was 76% with moderate agreement (k:0.49). Agreement was very good for anti-SRP (k: 0.85) and anti-Ku (k: 0.85), good for anti-Jo-1 (k: 0.69) and moderate for anti-PmScl (k:0.58), anti-MDA5 (k:0.49), anti-TIF1gamma (0.57). Jo-1 LB+ IIM had interstitial lung disease and arthritis compared to LB-negative (p < 0.001). Nineteen % of DM/PM patients were Jo-1+ compared to 0% of IBM (p < 0.001). Anti-TIF1gamma+ patients had higher frequency of DM skin rash than patients with other specificities (p = 0.003). LB-Mi-2+ was more common compared to IP+. Conclusion In conclusion, the concordance rate between the two assays for detection of myositis autoantibodies was moderate to very good for most prevalent specificities. The LB assay seems to be valid and useful to identify subgroups of IIM with specific clinical features.

### P98 Situ Lymphocyte and Dendritic Cell Characterization in Idiopathic Inflammatory Myopathies

#### Iazsmin Bauer Ventura, Daniel Reilly, Peter Pytel, Vladimir Liarski

##### University of Chicago, Chicago, USA

###### **Correspondence:** Vladimir Liarski

Background/Purpose: The Idiopathic Inflammatory Myopathies (IIM) are the largest number of acquired and potentially treatable muscle disorders. Nevertheless, there are currently no FDA or EMA-approved medications apart from corticosteroids. We speculate the scarcity of effective therapeutics mirrors the lack of in-depth characterization of the immunopathogenesis of IIM. In this pilot study, we aim to apply previously validated approach of multi-channel confocal microscopy using fluorescent antibodies for identification and characterization of DC and lymphocytes populations in biopsies of patients with Dermatomyositis (DM) and Inclusion Body Myositis (IBM).

Methods: A total of 4 DM and 4 IBM samples were stained for myeloid dendritic cells (mDCs) with BDCA1 and CD11c; plasmacytoid DCs (pDCs) with BDCA 2 and CD123; cell nuclei with DAPI (Hoechst 33342), plasma cells with CD138, B-cells with CD20, T-helper lymphocytes with CD4, and cytotoxic T lymphocytes with CD8. These slides were imaged with the SP8 3D 3-color STED laser scanning confocal microscope. Regions of Interest (ROIs) of each biopsy were randomly acquired by means of tiling using a mechanized stage. The resulting image data was reviewed and manually analyzed for number of DCs and lymphocytes by a blinded observer (IBV) using Fiji Software. Mann-Whitney U test was used to calculate statistical significance for all analyses.

Results: IBM biopsies had larger proportions of plasma cells, cell counts means of 22 versus 8 per biopsy (p=0.003), CD4+ lymphocytes, 73 versus 38 cells (p=0.03), CD8+, 36 versus 10 cells (p=0.002) in comparison to DM biopsies. In respect to dendritic cells, double-positive staining for mDCs was also greater in IBM, cell count means of 8 versus 1 per biopsy (p=0.000). Moreover, IBM biopsies had higher counts of BDCA2 positive cells (p=0.008). Biopsies of DM and IBM were similar as for CD20 positive cells and double-positive pDCs.

Conclusion: Our findings challenge the classic correlation between DM, IBM, pDCs, mDCs, and lymphocytes. Interestingly, we found a rich inflammatory milieu in IBM biopsies, with greater amounts of CD8 positive cells and mDCs, in concordance with the literature, but also of BDCA2, and CD4 positive cells. Other cell markers considered characteristic of DM, such as the pDC marker CD123 did not differ among the different diagnosis. This pilot study illustrates the complexity behind the cellular drives of IIM in regards to DCs. Priorly established patterns of inflammation used to describe these diseases must be revisited, as new therapeutic targets are urged in IIM.

### P99 Performance of the new EULAR/ACR Classification Criteria for Idiopathic Inflammatory Myopathies (IIM) in a Large Monocentric IIM Cohort

#### Simone Barsotti^1^, Valérie Leclaire^2^, Antonella Notarnicola^2^, Luoise Ekholm^2^, Lara Dani^2^, Maryam Dastmalchi^2^, Ingrid E. Lundberg^2^

##### ^1^University of Pisa, Pisa, Italy; ^2^Karolinska University Hospital, Stockholm, Sweden

###### **Correspondence:** Simone Barsotti

Background/Purpose: Patients with IIM have been classified mainly according to Bohan and Peter (B&P) criteria, proposed in 1975. In 2017 the new EULAR/ACR criteria were proposed. They are able to classify patients with both adult/juvenile form of IIM and they are applicable also in patients with amyopathic DM (ADM) and in IBM even without the results of muscle biopsy. The objective of this study was to evaluate the performance of the new classification criteria for IIM in a large retrospective cohort of patients.

Methods: Consecutive patients with a clinical diagnosis of IIM, based on physician opinion, referred to a rheumatology unit from 1995 to 2018 were included and assessed according to the B&P and the EULAR/ACR criteria. Data were collected from an existing database and, when missing, additional variables were retrieved from the patient’s records when available. Cohen’s kappa (K) was used to measure the agreement between physician opinion and the classification criteria; in addition, sensitivity and specificity were calculated.

Results: A total of 439 patients were included in the analysis (M 157, F 282, mean age at the onset 53.5±17.5 years). In 18 patients at the onset were less than 18 years old. According to the physician opinion 214 patients had PM, 57 IBM, 57 DM, 12 Juvenile DM (JDM), 5 ADM. With the EULAR/ACR criteria 385 patients (sensitivity 87.7%), could be classified as IIM, 285 definite and 100 probable: 161 PM, 137 DM, 60 IBM, 14 ADM, 13 juvenile IIM. Applying the B&P criteria, 353 patients (sensitivity 80.4%) were classifiable as IIM (183 definite, 170 probable): 204 PM and 149 DM. The concordance between the two criteria (Cohen’s K) was low (k=0.253 p=0.001). Fifty-four patients were not classifiable according to EULAR/ACR criteria (51 PM, 1 DM, 1 ADM, 1 JDM) and 86 with B&P criteria (55 PM, 19 DM, 8 IBM, 1 ADM and 3 JDM). Some patients with myositis specific autoantibodies were not classifiable (12 with EULAR/ACR, 30 with B&P). Missing detailed information on patterns of muscle weakness and some muscle biopsy features were explanations for not being able to classify most patients.

Conclusions: The new EULAR/ACR criteria performed well in a retrospective cohort using data from a myositis register and patient records from a myositis clinic, but they depend on muscle strength testing of upper and lower extremities as well as of proximal and distal muscles in the legs.

### P100 Tfh Cells in Human Myositis

#### Amrutesh Puranik^1^, Mark Jensen^1^, Yogita Ghodke-Puranik^1^, Regine Tipon^1^, Cynthia Loomis^1^, Valerie Mezzano^1^, Shanmugpriya Selvaraj^1^, Theresa Wampler Muskardin^1^, Lauren Pachman^2^, Ann Reed^3^, Timothy Niewold^1^

##### ^1^NYU School of Medicine, New York City, USA; ^2^Northwestern University, Evanston, USA; ^3^Duke University Medical Center, Durham, USA

###### **Correspondence:** Timothy Niewold

Objectives: T and B cells come together in ectopic lymphoid aggregates in myositis, suggesting that local T:B cell interactions could play a role in disease. T follicular helper cells are increased in circulation in patients with active myositis. We studied circulating Tfh cells from myositis patients using single cell RNA-sequencing, and examined the proximity of Tfh cells to B cells in patient biopsies.

Methods: Tfh cells were sorted from peripheral blood and subsets were identified by chemokine markers to designate Tfh1 and Tfh2/17 cell subsets. RNA sequencing was performed on individual cells, and data were analyzed using a pseudotemporal ordering strategy. Biopsies were stained using the OPAL standardized sequential immunofluorescence method for PD-1, CXCR5, CD19 and CD4 in human muscle, and machine learning was used to map proximity of B cells to all T-cells compared with Tfh cells.

Results: We found various subsets within the Tfh pool, corresponding to Tfh1 and Tfh2/17 cells and some cells that looked to be transitioning between states. Tfh2/17 were enriched in myositis patients vs. controls. The Tfh2/17 cells demonstrated an interferon signature, while the Tfh1 cells had a type II interferon and proteasome signature. In tissue, we could demonstrate Tfh cells in close proximity to B cells in lymphoid aggregates.

Conclusion: Tfh cells are present in myositis biopsies juxtaposed to B cells, suggesting productive T:B interactions in the tissue. Tfh subsets in blood from patients demonstrate distinct pathological signatures when compared to controls.

### P101 Role of mitochondria in membrane repair and muscle weakness in myositis

#### Kanneboyina Nagaraju^1^, Jessica Boehler^2^, Adam Horn^2^, James Novak^2^, Ning Li^1^, Svetlana Ghimbovschi^2^, Ingrid Lundberg^3^, Helene Alexanderson^3^, Li Alemo-Munters^3^, Jyoti Jaiswal^2^

##### ^1^Binghamton University, Binghamton, USA; ^2^Children’s National Health System, Washington, USA; ^3^Karolinska University Hospital, Stockholm, Sweden

###### **Correspondence:** Kanneboyina Nagaraju

Objective: Inflammatory myopathies are considered autoimmune disorders of muscle, but whether these diseases are mediated by the adaptive immune response or reflect the chronic inflammatory status of the diseased tissue itself is controversial. Immunosuppressive therapy shows variable efficacy in these patients, and there is little direct relationship between inflammation in muscle and the patient’s degree of weakness and disability. We have hypothesized that muscle weakness and damage is partially independent of adaptive immune response but is dependent on the ability of functional mitochondria to not only provide ATP but also repair muscle membrane after injury.

Methods: We have performed mechanistic studies involving gene and protein expression analysis of innate immune (e.g., toll like receptor) and metabolic pathways (e.g., Harakari) as well as monitoring mitochondrial activity and cells response to injury. We also evaluated effects of a moderate exercise regime on the reversibility of innate immune and metabolic changes in PM and DM patients.

Results: We present a novel model to explain the relatively poor correlation between inflammation and weakness in myositis. This model arose from our work that demonstrated pro-apoptotic mitochondrial protein, Harakari (Hrk) that selectively interact with survival-promoting Bcl-2 and Bcl-X(L), is significantly upregulated in myositis patient biopsies. This observation coupled with our recent studies that demonstrated functional mitochondria are essential not only for ATP generation but also for muscle membrane repair after injury. We found that primary muscle cells isolated from myositis patients showed increased Hrk expression and decreased mitochondrial activity and poor membrane repair after injury. We further confirmed that transient overexpression of Hrk in normal muscle cells lead to defective mitochondria and poor membrane repair that potentially lead to release of Danger Associated Molecular Patterns (DAMPs) as well as serum biomarkers such as creatine kinase in myositis patients. More importantly we found that TLR-7 signaling in skeletal muscle cells induce Hrk expression leading to a self sustaining loop in myositis muscle miroenvironment. We also show that moderate endurance exercise by myositis patients facilitates improved mitochondrial function by slowing disease progression through a reduction in HRK and TLR7 gene expression. This prevents the establishment of an HRK-TLR7 driven feed-forward loop that underlies pathogenesis of this disease

Conclusion: This model predicts that enhancing mitochondrial biogenesis and decreasing inflammatory cytokine signaling in skeletal muscle would improve muscle membrane repair and restore muscle strength in these patients.

### P102 Features of repeated muscle biopsies and of phenotypes of monocytes in paired blood samples and clinical long-term response to treatment in patients with idiopathic inflammatory myopathy – a pilot study

#### Karina R. Gheorghe^1^, Quan Tang^1^, Xing-Mei Zhang^1^, Eva Lindroos^1^, Helene Alexanderson^1^, Cecilia Wick^1^, Mei Bruton^1^, Robert A. Harris^1^, Inger Nennesmo^1^, Ingrid E. Lundberg^1^

##### Karolinska University Hospital, Stockholm, Sweden

###### **Correspondence:** Karina R. Gheorghe

Objective: Idiopathic inflammatory myopathies (IIM) are systemic inflammatory disorders primarily affecting skeletal muscle. Persisting muscle weakness despite immunosuppressive treatment leads to loss of function and disability over time. In this pilot study we aimed to identify biomarkers in repeated muscle biopsies and paired blood samples, taken before and after conventional immunosuppressive therapy, in order to predict long-term therapeutic responses in patients with IIM.

Methods: 13 newly diagnosed IIM patients were selected from our myositis registry (SweMyoNet), comprising six responders and seven non-responders. Treatment response 3 years after diagnosis was defined by achieving at least minimal improvement according to ACR/EULAR 2016 improvement criteria, together with reaching MMT-8 ≥ 78/80. Muscle biopsies were available at baseline from all selected patients. Post-treatment muscle biopsies after a median of 11 months follow-up were available from 9 patients and paired PBMCs from 5 patients. Frozen biopsy sections were stained immunohistochemically for expression of CD3, CD66b, IL-15, CD68, CD163, Major histocompatibility class I (MHC 1) and myosin heavy chain neonatal (MHCn). PBMCs were analyzed by flow cytometry for monocyte phenotypes (CD14, CD16, CD68, CX3CR1, and CCR2).

Results: Before treatment there were no significant differences between responders and non-responders in any of the clinical or muscle biopsy variables or frequency of monocyte subsets. MMT-8 was significantly higher compared to baseline in the responders 1 year after initiation of treatment, and this improvement was maintained at 3-year follow-up. In responders at 3 year follow-up the expression of CD68 in the post-treatment biopsy was significantly lower compared to in non-responders (P < 0.05).

Conclusions: In this pilot study, baseline biopsy, monocyte profile or clinical data did not predict long-term treatment response, but in the post-treatment biopsy within 1 year of immunosuppressive treatment a lower number of macrophages (CD68+) predicted a more favorable long-term clinical response regarding to improved muscle strength and disease activity. This data indicates that repeated muscle biopsy may offer additional information to guide further treatment.

### P103 The Göttingen Interdisciplinary Immunological Case Conference (GIICC): multi-disciplinary collaboration for effective care pathways of patients with myositis

#### Peter Korsten^1^, Jan-Gerd Rademacher, Cornelia Seitz, Jana Zschüntzsch, Rotraud Mößner, Michael Zeisberg, Ulrike Olgemöller, Cordula Buck, Sabrina Zechel, Radovan Vasko, Jens Schmidt

##### Universitätsmedizin Göttingen, Göttingen, Germany

###### **Correspondence:** Peter Korsten

Objectives: Inflammatory myositis (IM) comprises polymyositis (PM), dermatomyositis (DM), necrotizing myositis (NM), overlap myositis (OM), and antisynthetase syndrome (ASS). At the University Medical Center Göttingen, a tertiary reference center for IM, we have established an interdisciplinary case conference for patients in need of a comprehensive and multispecialty assessment.

Methods: This is a single center, retrospective observational analysis of patients evaluated at the GIICC. GIICC has been established as a monthly interdisciplinary meeting with fixed members from the Departments of Nephrology and Rheumatology, Neurology, Pulmonary medicine, Neuropathology, and Dermatology. 1 to 2 attending physicians of each Department regularly attend the conference. Patients with IM can be scheduled for a comprehensive evaluation and data from medical records (history and physical examination, laboratory data, imaging, pathology) are assessed. Consensus is reached by face-to-face discussion of each patient. A report is generated in the general hospital documentation system. Patients can be allotted for additional diagnostic evaluation, change of treatment, or treatment recommendations (“second opinion”) for primary or secondary care providers.

Results: The majority of patients is scheduled for additional diagnostic tests, either at the Department of Neurology or at the Department of Nephrology and Rheumatology. Most patients receive immunosuppressive therapies (second or third line treatments, such as immunosuppressants, IVIG and/or Rituximab) for interstitial lung disease or myositis. Many patients require rheumatologic evaluation for either presence or absence of arthritis, presence of an additional autoimmune disorder or specific diagnostic tests, such as nailfold video capillaroscopy, musculoskeletal ultrasound or MRI. Patients scheduled for neurologic evaluation mostly require re-evaluation of muscle histopathology, repeat EMG testing or additional imaging tests, including MRI of muscles and/or myosonography.

Conclusions: By evaluating patients with IM at the GIICC, our experience is that patients receive a comprehensive, interdisciplinary, high class expert evaluation in a timely manner and with a concrete diagnostic/treatment plan. Furthermore, patient care is harmonized between the respective Departments following a Standardized Operating Procedure. Based on our preliminary retrospective analysis, we plan to prospectively assess GIICC in the setting of a quality improvement study (QIS) following the recommendations of the EQUATOR network.

### P104 ACR/EULAR myositis classification 2017: “real world data” from the Göttingen cohort

#### Kanan Hasanov, Sabrina Zechel, Rachel Zeng, Stefanie Glaubitz, Peter Korsten, Cornelia S. Seitz, Jens Schmidt

##### University Medical Center Göttingen, Göttingen, Germany

###### **Correspondence:** Kanan Hasanov

Background: The ACR/EULAR classification criteria for myositis have recently been published. They are based upon a large cohort of patients from databases, including the network of IMACS and the Euromyositis registry.

Objective: The aim of this study is to provide “real-world” data on the ACR/EULAR classification criteria for myositis by applying the criteria to our cohort of myositis patients at the University Medical Center Göttingen, Germany.

Material and Methods: In a retrospective fashion, 270 patients with myositis were included and IRB-approved informed consent was obtained. The patients were treated at the University Medical Center between 2009 and 2018 and had received the diagnosis polymyositis (PM), dermatomyositis (DM), necrotizing myopathy (NM), anti-synthetase syndrome (ASS) or inclusion body myositis (IBM). The clinical records were assessed for all parameters required for assessment of the ACR/EULAR classification criteria.

Results: The score and diagnosis according to the ACR/EULAR classification is compared to all other major myositis classifications for PM and DM (Bohan & Peter, Tanimoto, Targoff, Dalakas, and Hoogendijk) and for IBM (Dalakas, Griggs, Rose). The data will evaluate the sensitivity and specificity by assessing the respective diagnosis for each of the classification criteria.

Discussion: This study may support the future use of the ACR/EULAR classification criteria for myositis by providing “real-world data” from a well characterized cohort. The data will be helpful for continued update of the classification criteria.

### P105 Incidence of Idiopathic Inflammatory Myopathies (IIM) in Adults in Fife, Scotland, UK

#### John McLaren, Paul Allcoat, Michael Hearst, Elizabeth Furrie, Sarah Hailwood

##### NHS Fife, Kirkcaldy, UK

###### **Correspondence:** John McLaren

Objectives: To determine the Adult incidence of IIM in the Kingdom of Fife, Scotland. The Fife population is 370,000 and constitutes 6.8% of the Scottish Population (5,425,000). 98% of the Fife population is Caucasian. We also sought to characterise the incident IIM cases in terms of subtype and autoantibody profile.

Methods: All newly diagnosed Adult cases (≥ 18 years) of IIM between 01.03.17 and 30.11.18 were recorded (11.75 year period). The cases were identified by a search of the Fife Rheumatic Diseases Unit Database using IIM-specific ICD-10 codes. These were cross-referenced with a prospective personal database of IIM cases established by the Lead Author (JMcL) on 26.02.17. In addition a search of all Fife patients enrolled in the UKMYONET study was also performed. All patients with ‘definite’ or ‘probable’ IIM by the Bohan & Peter 1977 criteria; Clinically defined Inclusion Body Myositis (IBM) by the ENMC 2011 criteria; or other specific criteria in the case of Clinically Amyopathic Dermatomyositis (CADM) and Immune-mediated Necrotising myopathy (IMNM) were included.

Results: 53 newly diagnosed Adult IIM cases were identified. 11 Dermatomyositis (DM) (3 NXP2; 2 Jo-1; 2 TIF1-G; 1 Mi-2; 3 Myositis-specific antibody (MSA) not tested). 4 Polymositis (PM) (1 Jo-1; 1 MDA5; 1 MSA/Myosits-associated antibody (MAA) negative; 1 MSA not tested). 12 Anti-synthetase syndrome (8 Jo-1; 3 PL7; 1 PL12) and 3 patients with Jo-1 isolated Interstitial lung disease (ILD). 5 Overlap Connective tissue disease (CTD) : 2 Systemic Lupus Erythematosus; 2 Systemic sclerosis; 1 Mixed CTD. 5 Cancer-associated myositis (CAM) : 2 lung (1 NXP2 DM & 1 SRP PM); 1 ovarian (DM, MSA not tested); 1 Breast (DM, TIF1-G); 1 Prostate (DM, Mi-2). 5 CADM (2 MDA5; 1 TIF1-G; 1 NXP2; 1 MSA not tested). 4 IMNM (2 HMGCR; 1 HMGCR overlap with IBM; 1 SRP overlap with IBM who developed bladder cancer < 3 years after diagnosis). 4 sporadic IBM (2 cN-1A/Mup44 of which 1 overlap with Primary Sjogren’s Syndrome; 2 MAA not tested).

Conclusions: The incidence of IIM in Fife, Scotland is 4.5 cases per year which equates to an annual incidence of 12.2 cases per million population. This is comparable with the incidence data published for Salford in the UK (17.6). However the IIM subtypes differed from Salford in that Fife has a much lower incidence of PM. 40/49 (82%) of Fife patients who were tested were found to be MSA positive.

### P106 Muscle inflammation and muscle weakness in mouse models of neuromuscular diseases

#### Amanda Mullen^1^, Joyce Rowsell^1^, Kitipong Uaesoontrachoon^1^, Alex MacKinnon^1^, Molly Praest^1^, Sadish Srinivassane^1^, William Ross^1^, Donika Shala^1^, Jordan Warford^1^, Eric Hoffman^2^, Kanneboyina Nagaraju^2^

##### ^1^AGADA Biosciences Inc, Halifax, Canada; ^2^Binghamton University, Binhamton, USA

###### **Correspondence:** Amanda Mullen

Objective: It is generally perceived that excessive muscle inflammation may be responsible for causing muscle weakness in chronic inflammatory muscle diseases. Evaluation of muscle inflammation using qualitative/semi-quantitative histological (e.g., H&E) measures is not optimal in neuromuscular diseases because inflammation is often patchy. Therefore, we have developed a non-invasive quantitative measure of muscle inflammation in live mice using ProSense® 680, a cathepsin (macrophage) activatable fluorescent in vivo imaging agent. Here we have tested the hypothesis that excess muscle inflammation is associated with more muscle weakness.

Methods: We have used male (age: 9-10 weeks) dystrophin deficient mdx (n=12) and normal control (n=8) mice to test this hypothesis. We measured muscle inflammation in the hind limbs of control and mdx mice using near-infrared fluorescent agent, Prosense680 on the IVIS Lumina III Imaging System. We also measured in vivo specific force of the tibialis anterior (TA) muscle on these mice using the Aurora Scientific 809C in-situ Mouse Apparatus. Both assessments were done in a blinded fashion.

Results: Mdx mice showed highly variable but significantly increased inflammation in hindlimbs in comparison to BL10 control mice. Median cathepsin activity measured as radiant efficiency units (REU) in Mdx mice is about 295% higher than control BL10 mice (mdx vs BL10: 4.07E8 vs 1.38E8 REU) suggesting macrophage infiltration in skeletal muscle. Mdx mice also showed lower specific force of the TA muscle in comparison to control BL10 mice. Median TA specific force in mdx mice is 60% lower than control BL10 mice (mdx vs BL10: 26.00 kN/m2 vs 43.20 kN/m2). Correlation of cathepsin activity with specific force of the TA muscle indicates that muscle inflammation is modestly correlated with muscle specific force (p=0.0007, r2 0.48).

Conclusion: We have developed a non-invasive quantitative measure of muscle inflammation that can be used longitudinally in live animals. We found a modest correlation between muscle inflammation and specific force suggesting other non-inflammatory factors may play a role in muscle weakness in dystrophin deficient mdx mice.

### P107 National trends in Utilization of Emergency Department Services and Health Care Cost associated with Idiopathic Inflammatory Myopathy visits: An analysis of Nationwide Emergency Department Survey in USA, 2010-2014.

#### Umar Zahid^1^, Sunita Upreti^1^, Katherine Ellingson^2^, Lisa Christopher-Stine^1^

##### ^1^Johns Hopkins University School of Medicine, Baltimore, USA; ^2^University of Arizona, Tucson, USA

###### **Correspondence:** Umar Zahid; Lisa Christopher-Stine

Introduction: Idiopathic inflammatory myopathies (IIMs) are a cluster of uncommon disorders. Most often these patients are on long term treatment and they tend to have frequent emergency department (ED) visits either with a disease flare or with complications from therapy. Data about the trend of ED utilization and health care burden of IIM patients is lacking. Objective: To estimate the IIMs related ED visits, disposition from ED, health care cost and the predictors of subsequent hospitalization.

Methods: We used the Nationwide Emergency Department Sample (NEDS) dataset. Chi-square and t-tests were used to test statistical significance of categorical and continuous variables respectively. Trend of IIM related visits from 2010-2014 was tested using Cochrane-Armitage-test. Additionally, predictors of hospital admission were assessed by running separate multivariable logistic regression model for each type of IIM (dermatomyositis (DM), polymyositis (PM) & inclusion body myositis (IBM)).

Results: A nationally weighted estimate of 82,241 ED visits with the diagnosis of IIMs (DM=37.8%, PM=54.0% & IBM=8.2%) were identified, that accounted for 0.12% of all ED visits from 2010 to 2014. Over the period of five years, an absolute increase of 20.8% DM related ED visits and 57% IBM related visits were observed (p=0.04) while PM related ED visits decreased 9.3% from 2010-2014. Median number of co-morbidities were increased in patients with IBM compared to PM and DM (IBM=12, PM=11, DM=10). Total national cost of IIM related visits increased by 62% from $6.97 million to $11.3 million from 2010 to 2014 (p < 0.05). Median cost was similar [IBM ($1857) PM ($1850) and DM ($1808)]. Overall 71% of all IIM related ED visits resulted in hospitalization; significant higher rates of hospitalization were noted in IBM patients. However, the rate of hospitalization after an IIM related ED visit decreased by 8% over the study time period. Median length of hospital stay in patients with IIM was 4 days. Among the patients hospitalized with IIM, 51% were discharged home, 24% were transferred to skilled nursing facility and 17% were send home with health care. Several demographic characteristics were found to be significantly associated with a higher odds of hospitalization in an adjusted multivariable regression model such as old age, male gender, geographic location and number of co-morbidities.

Conclusion: ED services utilization and associated total costs are increasing over time for the patients with IIM. However, with the advancement in the treatment, rate of hospitalization and length of stay are significantly decreasing.

### P108 The prevalence of p62 immunostaining in muscle biopsies from patients with myositis, non-inflammatory myopathies, and neurogenic disorders

#### José C. Milisenda^3^, Iago Pinal-Fernandez^1^, Thomas E. Lloyd^2^, Josep Maria Grau^3^, Andrea L. Corse^1^,Andrew L. Mammen^1^

##### ^1^National Institutes of Health, Bethesda, USA; ^2^Johns Hopkins University School of Medicine, Baltimore, USA; ^3^Hospital Clínic de Barcelona, Barcelona, Spain

###### **Correspondence:** José C. Milisenda; Iago Pinal-Fernandez

Objectives: p62 is a marker of autophagy that has been proposed to be useful to diagnose patients with inclusion body myositis. The objective of this study was to assess the diagnostic utility of p62 by comparing the prevalence and pattern of p62 staining in muscle biopsies from patients with inclusion body myositis compared to other types of inflammatory and non-inflammatory myositis.

Methods: All patients from the Johns Hopkins Myositis Center longitudinal cohort from August 2013 to January 2017 with an available muscle biopsy and p62 immunostaining were included in the study. p62 immunostaining was classified as negative if all muscle fibers were negative, and positive if it any of the six patterns described in Figure 1 was present. All biopsies were reviewed by an experienced myopathologist who was not blinded for clinical information and by a myology specialist who was masked to disease activity and subgroup.

Results: 399 muscle biopsies with p62 immunostaining were available for the study. 136 muscle biopsies were from patients with myositis including 37 with IBM, 40 with DM, 31 with immune-mediated necrotizing myopathy [IMNM] and 28 with PM patients. The rest were from patients with other myopathies (n=53), neurogenic disorders (n=85), normal biopsies (n=29) and biopsies with an undefined diagnosis (n=96). The proportion of p62 positive cases were 67.6% in the myositis group and 0% in those with normal biopsies. p62 staining was most prevalent in IBM (92%), IMNM (87%), and dermatomyositis (57%) muscle biopsies (all "p < 0.001") but uncommon in PM (29%) and in other types of myositis (all < 31%). Except for the perivacuolar pattern (19%, "p < 0.001"), IBM was not associated with any characteristic distribution of p62 staining (Table 1).

Conclusion: p62 immunostaining is not specific for IBM and is also common in IMNM and DM muscle biopsies.

### P109 The spectrum of idiopathic inflammatory myopathy in a multi-ethnic Malaysian population

#### Khean Jin Goh, Tomica Ambang, Cheng Yin Tan, Jasmin Raja, Kum Thong Wong

##### University of Malaya, Kuala Lumpur, Malaysia

###### **Correspondence:** Khean Jin Goh

Objectives: To describe the spectrum of inflammatory myopathy in a multiethnic Asian population.

Methods: Patients whose muscle biopsy was diagnosed as inflammatory myopathy at the Pathology Department, University of Malaya between 1995 and 2016 were reviewed. Clinical data and muscle pathology were retrospectively reviewed and re-classified according to the European Neuromuscular Center (ENMC) criteria. Biopsies diagnosed as polymyositis (PM) or nonspecific myositis were specifically reviewed by a muscle pathologist and re-classified if necessary. Serological studies were carried out in some cases only. Inclusion body myositis (IBM) was classified according to the ENMC IBM diagnostic criteria. Overlap myositis is there was associated connective tissue disease and myositis associated with antisynthetase syndrome (ASS) were diagnosed if there were positive antisynthetase antibodies or if there were additional compatible clinical features such as interstitial lung disease, Raynaud phenomenon or mechanics hands.

Results: There were 338 patients, 221 (65.4%) were female. Ethnic groups included 165 (48.8%) Chinese, 118 (34.9%) Malays, 46 (13.6%) Indians and 9 (2.7%) other ethnicities, reflecting the ethnic breakdown in urban Malaysia. 138 (40.8%) were dermatomyositis (DM) (63 (42.7%) juvenile DM (JDM) and 75 (57.2%) adult DM); 80 (23.7%) immune-mediated necrotizing myopathy (IMNM); 37 (10.9%) PM; 33 (9.8%) overlap myositis (including 6 with ASS) and 19 (5.6%) were IBM. A further 32 (9.5%) were classified as nonspecific myositis. Mean age and range for myositis subgroups were 7.7 years (1-17) (JDM), 49 (20-80) (adult DM), 40.4 (6-80) (IMNM), 49.6 (17-79) (PM), 61.2 (40-79) (IBM) and 37.2 (8-61) (overlap myositis). There were more women in all subtypes except IBM where 63.2% were men. All subtypes had more ethnic Chinese-Malaysian patients except JDM and overlap myositis in which there were more ethnic Malays. Of 36 IMNM patients with serological testing, 16 (44.4%) were positive for anti SRP antibody, six (16.7%) were anti HMGCR antibody and two were positive for both antibodies. 12 (33.3%) were negative for both antibodies. In 6 patients with ASS, anti Jo1 was positive in 5 patients and anti PL7 was positive in one. There were 8 (2.4%) malignancies, 7 (11.1%) adult DM and 1 nonspecific myositis.

Conclusions: Common inflammatory myopathy subtypes are dermatomyositis and immune-mediated necrotizing myopathy. IBM is seen Malaysians but in a lower proportion of cases. The subtypes and demographic characteristics of inflammatory myopathy in Malaysians are similar to other populations.

### P110 The role of Muscle Biopsy Score in Assessing Disease Activity in Adult Patients with Idiopathic Inflammatory Myopathies

#### Lining Zhang, Xin Lu, Xiaolan Tian, Xiaoxiao. Zheng

##### China-Japan Friendship Hospital, Beijing, China

###### **Correspondence:** Lining Zhang

Objective: The aim of this study was to investigate the role of muscle biopsy score in relation to the disease activity of idiopathic inflammatory myopathy(IIM) in Chinese adult patients.

Methods: Total 423 adult IIM patients in China-Japan Friendship Hospital from December 2008 to June 2017 were included in this study. The score of disease activity was assessed using IMACS Core Set Measures of IIM. The muscle biopsy specimens of IIM patients were performed by immunohistochemical staining of MHC-I, CD3 and CD20 molecules. Furthermore, the muscle biopsy score of each specimens was measured separately by four parts of muscle tissue including muscle fiber, inflammation infiltrating, blood vessel and connective tissue.

Results: The degree of muscle biopsy score was associated with the overall disease activity (r=0.228, P< 0.05), the increase of muscle activity(VAS)(r=0.407, P< 0.05) and creatine kinase(CK) levels (r=0.466, P< 0.05) of IIM patients, respectively. Moreover, among untreated patients, the muscle VAS of dermatomyositis(DM) were positive correlated to the muscle biopsy total score, the score of muscle fiber and inflammation infiltrating of muscle tissue(r=0.30, 0.312 and 0.241, P< 0.05) respectively. While, the high score of muscle fiber was related to the disease activity of polymyositis(PM) patients (r=0.478, P< 0.05). When analyzed according to different myothitis specific antibodies(MSAs) groups among initial untreated patients,including anti-TIF-1 antibody,anti-NXP-2 antibody,anti-Mi-2 antibody,anti-MDA-5 antibody,anti-Jo-1 antibody,non Jo-1 anti-synthetase antibody,anti-SRP antibody,anti-HMGCR antibody,and antibody-negtive bodies,we found that patients with anti-SRP or anti-HMGCR autoantibodies showed high correlation between MB score with muscle activity(VAS)(r=0.933 and 0.975,P< 0.05),which showed no significant correlations in other autoantibody subgroups.

Conclusion: The increased muscle biopsy score, including muscle fiber and inflammation infiltrating score in muscle tissue are related to the disease activity of PM and the CK levels of DM, as well as in some of the MSAs positive subgroups. The measures of muscle biopsy score can be used as an effective tool to evaluate the disease activity of IIM.

### P111 Hsp90 is increased in plasma and muscle tissue in idiopathic inflammatory myopathies and correlates with disease activity and skeletal muscle involvement

#### Hana Storkanova^1^, Sabina Oreska^1^, Maja Spiritovic^2^, Barbora Hermankova^2^, Olga Krystufkova^1^, Herman Mann^1^, Karel Pavelka^1^, Jiri Vencovsky^1^, Ladislav Senolt^1^, Michal. Tomcik^1^

##### ^1^Institute of Rheumatology, Prague, Czech Republic; ^2^Charles University, Prague, Czech Republic

###### **Correspondence:** Hana Storkanova

Objectives: Heat shock proteins (Hsps) are chaperones playing important roles in skeletal muscle physiology, adaptation to exercise or stress, and activation of inflammatory cells. The aim of our study was to assess Hsp90 expression in muscle biopsies and plasma of patients with idiopathic inflammatory myopathies (IIM) and to characterize its association with IIM-related features.

Methods: Total of 277 patients with IIM (198 females, 79 males; mean age 54.8; disease duration 4.1 years; dermatomyositis (DM, 104)/polymyositis (PM, 104)/cancer associated myositis (CAM, 42)/ necrotizing myopathy (IMNM, 27)) and 100 age-/sex-matched healthy individuals were included in plasma analysis and 50 muscle biopsy samples were stained for Hsp90 (PM-10, DM-10, IMNM-10, myodystrophy-10, myasthenia gravis-10). Patients with PM/DM fulfilled Bohan and Peter criteria and CAM was defined as cancer within 3 years of IIM diagnosis. Plasma Hsp90 was measured by ELISA kit (eBioscience, Vienna, Austria). Data are presented as median.

Results: In muscle biopsies Hsp90 expression was higher in IIM than in myodystrophy (myasthenia gravis used as another control was negative). Increased Hsp90 was detected in perifascicular degenerating and regenerating fibers, inflammatory cells (DM, PM), and necrotic and regenerating fibers (IMNM). Plasma Hsp90 levels were increased in IIM patients compared to healthy controls (20.2 vs. 9.2 ng/mL, p < 0.0001), and in individual subgroups of IIM vs. healthy controls (PM: 19.4, DM: 22.4, CAM: 19.1, IMNM: 19.6 ng/mL, p < 0.0001 for all). Hsp90 levels in all patients positively correlated with LD and AST (r=0.551, p < 0.0001; r=0.372, p < 0.0001, respectively), and there was a trend towards correlation with CK (r=0.111, p=0.068). Increased Hsp90 was associated with decreased MMT-8 values (r=-0.136, p=0.029), in particular in proximal muscles. Hsp90 positively correlated with patient- and doctor- evaluated disease activity (r=0.222, p=0.0004; r=0.217, p=0.0005, respectively), pulmonary and muscle disease activity (r=0.201, p=0.001; r=0.146, p=0.018, respectively), MITAX and MYOACT (r=0.175, p=0.005; r=0.159, p=0.012, respectively), and with MDI extent/severity (r=0.215, p=0.003; r=0.120, p=0.041, respectively). Higher Hsp90 was found in patients with interstitial lung disease, cardiac involvement and dysphagia (25.4 vs. 18.9, p=0.004; 27.5 vs. 19.3, p=0.004; 25.0 vs. 18.2 ng/mL, p=0.018, respectively).

Conclusions: We demonstrate increased Hsp90 expression in IIM muscle biopsy samples, specifically in inflammatory cells, degenerating, regenerating and/or necrotic fibers. Increased Hsp90 plasma levels in IIM patients are associated with disease activity and damage, and with the involvement of proximal skeletal muscles, heart and lungs. Acknowledgement: Supported by AZV-16-33542A, MHCR 023728, and SVV – 260373.

### P112 A high prevalence of conformational epitopes in patients with anti-TIF1γ limits the utility of solid-phase immunoassays as a detection method

#### Sarah Tansley, Zoe Betteridge, Hector Chinoy, Lucy Wedderburn, Neil McHugh

##### Royal National Hospital for Rheumatic Diseases, Bath, UK

###### **Correspondence:** Sarah Tansley

Objectives: Autoantibodies targeting TIF1γ form the most common serological sub-group of patients with juvenile-onset myositis, and are clinically important in adult onset disease where they are strongly associated with malignancy. The desire for non-specialist, high throughput laboratory systems to detect myositis autoantibodies has delivered a range of immunoassays to detect key myositis autoantibodies, including anti-TIF1γ. Conformational epitopes are known to occur in a number of autoimmune diseases and have also been described with anti-TIF1γ. We aimed to determine whether conformational epitopes impacted on the sensitivity of different immunoassays.

Methods: We had previously determined the autoantibody status of over 3,000 patients with myositis using radioimmunoprecipitation. Anti-TIF1γ positive samples (determined by the presence of a 155/140kDa band on immunoprecipitation) were selected for further analysis by ELISA using recombinant TIF1γ (159 samples analysed, 72 juvenile-onset) and a commercially available lineblot (Euroimmun) (102 samples analysed, 53 juvenile-onset). Reverse immunoprecipitation blotting was performed using either monoclonal anti-TIF1γ(Sigma) or patient sera to confirm the presence of anti-TIF1γ in ELISA and lineblot negative sera.

Results: Using the immunoprecipitation result as a reference, 125/159 (79%) anti-TIF1γ positive samples were also positive by ELISA. Lineblot was less sensitive and only 59/102 (58%) of samples tested were positive. There was no difference in sensitivity between those with adult and juvenile-onset disease. Only two samples negative by ELISA (14%) were positive by lineblot, and both were weak positives. Sixteen of the ELISA and lineblot negative samples were further analysed by reverse immunoprecipitation blotting: 11/16 (69%) were positive, confirming the presence of autoantibodies targeting TIF1γ. Five (83%) of 6 adult patients with confirmed anti-TIF1γ and a known history of malignancy were positive by ELISA and 4 (66%) by lineblot.

Conclusions: In our cohort, ELISA was a more sensitive method for detecting anti-TIF1γ than lineblot, but both methods can fail to identify anti-TIF1γ in patients with malignancy. Approximately 1 in 5 myositis patients with anti-TIF1γ determined by immunoprecipitation had a negative result by ELISA but over a third were negative using a commercially available lineblot. In most patients with a 155/140kDa band on immunoprecipitation who test negative for anti-TIF1γ by ELISA and lineblot, anti-TIF1γ can be confirmed by reverse immunoprecipitation blotting. This suggests the presence of a conformational epitope. Alternative methodologies should be considered to enable sensitive, high-throughput testing for this clinically important autoantibody.

### P113 Towards disentangling the genetics of myositis: the DISSECT project experience

#### Matteo Bianchi^1^, Antonella Notarnicola^2^, The Dissect Consortium, Louise Pyndt Diederichsen^3^, Øyvind Molberg^4^, Hector Chinoy^5^, Janine Lamb^5^, Lars Rönnblom^1^, Kerstin Lindblad-Toh^1^, Ingrid E. Lundberg^2^

##### ^1^Uppsala University, Uppsala, Sweden; ^2^Karolinska Institutet, Stockholm, Sweden; ^3^Odense University Hospital, Odense, Denmark; ^4^University of Oslo, Oslo, Norway; ^5^University of Manchester, Manchester, UK

###### **Correspondence:** Matteo Bianchi

Objectives: The DISSECT project is a northern European multidisciplinary framework aiming at disentangling the genetics of myositis and other inflammatory autoimmune diseases, all sharing the type I interferon signature. Within this collaborative project, the main objectives of the myositis working-group have been the following: • To collect detailed clinical and serological information, as well as generate targeted resequencing data from northern European myositis patients and healthy controls. • To establish a database and a user-friendly analytical platform that will provide a foundation for our myositis research projects. • To dissect the genetic components underlying myositis and its disease mechanisms.

Methods: Detailed clinical and demographic data, as well as biological specimens and serological measurements for myositis-characterizing autoantibodies have been obtained for 560 Scandinavian myositis patients (Sweden, Denmark, Norway) and 424 patients from Great Britain. The majority of the cases presented either with polymyositis or dermatomyositis. Additionally, healthy controls matched for population ancestry were sampled (1,319 from Sweden and Norway) or relevant data retrieved from publicly available repositories (1,812 British controls). We performed targeted resequencing of 1,853 genes involved in immune function and autoimmunity in all the sampled myositis patients and controls. Targets included coding sequences, full untranslated regions and conserved intronic and intergenic regions. After preliminary bioinformatic analysis, all the processed and quality-controlled genetic data, as well as the individual metadata, were uploaded and now constitute the core of the database together with several tailored statistical genetic tools for downstream analyses.

Results: After resequencing (targeting 32 Megabases, ~40x coverage), genetic imputation and subsequent quality control, both the Scandinavian and the British datasets comprise more than 500,000 high quality variants. Despite experiencing issues with harmonizing the analysis in different cohorts and with using out-of-study sample-sets, single variant and aggregate case-control association tests, as well as meta-analysis are being performed in order to confirm known and unravel novel loci associated with myositis. For example, the human leucocyte antigen locus shows a statistically significant association (p-value < 1x10-16) with myositis in both cohorts. Moreover, cases are being stratified according to their serological status and evaluated for any association with genetic variants with polymyositis and dermatomyositis.

Conclusion: We expect that our study will ultimately enable to improve diagnostics and targeted treatments by finding disease variants likely to be causative in the studied populations, thus promoting precision therapy for myositis. Moreover, the establishment of the DISSECT myositis research database will facilitate future collaborative studies on myositis.

### P114 Predictive factors for treatment response for patients with idiopathic inflammatory myopathies a registry-based cohort

#### Hector Fabricio Espinosa-Ortega, Marie Holmqvist, Maryam Dastmalchi, Ingrid E. Lundberg, Helene Alexanderson

##### Karolinska University Hospital, Stockholm, Sweden

###### **Correspondence:** Hector Fabricio Espinosa-Ortega

Objectives: The aim of this study was to test the usefulness of autoantibodies and clinical features as predictors for response to immunosuppressive therapy after one year in with patients with idiopathic inflammatory myopathies followed longitudinally in an electronic myositis registry used in health care.

Methods: Patients identified in the SweMyoNet registry between January 1st, 2003 and December 31st, 2015 within 12 months of diagnosis were included. Patients with inclusion body myositis were excluded. The follow-up period ended at the closest visit to one year after start of treatment when response to therapy was assessed. Treatment of the individual patient was based on the treating physician´s decision. Patients were categorized serologically by the presence of 1) any antisynthetase autoantibody, 2) non-antisynthetase autoantibody, 3) any myositis associated antibody, and 4) negative to any of these. The ACR/EULAR 2016 criteria for Clinical Response were applied to measure response, and it was categorized in minimal (20-39/100), moderate (40 59/100) and major (>60/100) response. We tested the association between serological groups and other predictors with every category of response by logistic regression modelling.

Results: 179 patients were identified. Mean age at inclusion was 57 years (±14), 65% were female. 59 (33%) patients had dermatomyositis, 81 (45%) polymyositis and 39 (22%) overlap myositis. 136 patients (77%) were positive to any autoantibody, 49% of these (n=67) were positive to myositis specific antibodies. 91% were given glucocorticoid treatment, 72% immunosuppressive drugs, 22% cyclophosphamide and 13% a biological drug. 111 of the 179 patients (62%) met the criteria for minimal response, 68 (38%) for moderate response and 32 (18%) for major response. We found no difference between the autoantibody defined subgroups and each of the categories of response. When we analysed time of first symptoms to diagnosis and glucocorticoid dose at baseline, we found that each month of delay from first symptoms to diagnosis was negatively associated with a decrease of 13% in the odds to achieve major response (OR 0.87, 95%CI 0.79-0.99) and an increase of 1mg of glucocorticoid at baseline was independently associated to 4% and 3% in the odds to achieve minimal and moderate response, respectively (OR 1.04 95%CI 1.02-1.06; 1.03 95% CI 1.01-1.05).

Conclusions: A shorter time span from first symptoms to diagnosis and more intensive initial immunosuppressive treatment were independently associated to higher rates of clinical improvement after one year with pharmacological treatment.

### P115 Muscle ultrasound in patients with Inclusion Body Myositis: differentiating from mimics

#### Kristofoor Leeuwenberg^1^, Lisa Christopher-Stine^2^, Julie Paik^2^, Eleni Tiniakou^2^, Nens van Alfen^1^, Jonne Doorduin^1^, Christiaan Saris^1^, Jemima Albayda^2^

##### ^1^Radboud University, Nijmegen, Netherlands; ^2^Johns Hopkins University School of Medicine, Baltimore, USA

###### **Correspondence:** Kristofoor Leeuwenberg

Objectives: Inclusion Body Myositis (IBM) is the most common inflammatory myopathy in individuals over the age of 50 years. When typical features of IBM are missing, its presentation can easily be confused with other forms of myositis or neuromuscular disorders. Recent studies have shown that muscle ultrasound (US) has the potential to differentiate between IBM and its mimicking diseases. In this study we aimed to further evaluate the ability of US to differentiate between IBM and Polymyositis/Dermatomyositis (PM/DM) and neuromuscular disorders using two separate cohorts.

Methods: Patients were included from two centers: the Johns Hopkins Myositis Center and the Muscle Center Radboudumc. We compared echo intensity and muscle thickness of the flexor digitorum profundus (FDP), gastrocnemius, rectus femoris and vastus lateralis muscles in patients with IBM (n = 41), PM/DM (n = 37), other neuromuscular disorders (n = 11) and healthy controls (n = 88). All patients with IBM, PM and DM met the EULAR/ACR classification criteria for each disease. The neuromuscular disorders consisted of five myopathies, four motor neuron diseases (ALS or PSMA), one polyneuropathy and one Lambert Eaton myasthenic syndrome.

Results: Echo intensity was higher and muscle thickness lower in all four muscles in IBM compared to PM/DM. When comparing IBM to the neuromuscular disorders, only the FDP showed a significantly higher echo intensity in IBM. The vastus lateralis was the only muscle with a significantly lower muscle thickness than the neuromuscular disorders. When evaluating the diagnostic potential per muscle group, logistic regression models using both ultrasound parameters showed that the FDP and vastus lateralis were the most discriminatory for IBM. However, the FDP proved to be the best candidate with a mean sensitivity/specificity of 94,9%/78,9% versus PM/DM and 93,8%/70,0% versus other neuromuscular disorders.

Conclusion: Among several characteristically involved muscles, the FDP and quadriceps muscles are the most discriminating for IBM on US when comparing to mimicking diseases (other myositis and neuromuscular controls). This may have value as a diagnostic tool for IBM.

### P116 Quantitative Strength Profiling in Inclusion Body Myositis: Faster Deterioration in Males and Positive Associations with Functional Assessment Tools

#### James Lilleker^1^, Alexander Oldroyd^1^, Hector Chinoy^1^, James Miller^2^

##### ^1^University of Manchester, Manchester, UK; ^2^Royal Victoria Infirmary, Tyne, UK

###### **Correspondence:** James Lilleker

Background: Inclusion body myositis (IBM) is a rare idiopathic inflammatory myopathy subtype. Previous observational research has been limited to short-term studies. Detailed characterisation of the progression of muscle weakness is essential for clinical prognostication and delineating research end-points. Our study aimed to describe the disease progression rate in IBM using measures of muscle strength and functional assessment tools.

Methods: We performed a retrospective analysis of quantitative muscle strength data (six upper-limb and five lower-limb muscle groups using a CITEC dynamometer) and functional assessment tool results (IBM Functional Rating Scale [IBM-FRS] and the Neuromuscular Symptom and Disability Score [NSS]), systematically collected from verified IBM cases between July 2003 and March 2015 at a single UK neuromuscular clinic. Yearly rate of strength change was estimated for each muscle group and associations between strength and the IBM-FRS / NSS were examined using simple linear regression.

Results: Data from 80 IBM cases (44% female) were analysed, a total of 232 person-years and a median 2.3 years (IQR 0.6, 4.4) follow-up. Median age at IBM diagnosis was 68.4 years (62.4, 74.3). 4,760 strength measurements, 357 NSS measurements and 127 IBM-FRS measurements were performed. Similar rates of deterioration were found for right and left sided muscle groups. Annual rate of strength loss was greatest for grip, pinch and knee extension (-5.5% [95% CI -6.3, -4.6], -16.0% [-18.4, -13.5] and -7.1% [-7.6, -6.5] respectively). The rate of strength loss was greatest for the male cohort. Increased strength measurements were associated with improved IBM-FRS / NSS scores for most muscle groups tested.

Conclusion: This is the first long-term study to comprehensively demonstrate quantitative strength change in a large IBM cohort. Strength reduction occurred more rapidly in the male population. Our study provides valuable natural history data to support discussions regarding prognosis, will focus effective choice of outcome measures and inform study numbers for adequate statistical power in future clinical trials.

### P117 Application of electrical impedance myography (EIM) as a potential biomarker of Inclusion Body Myositis (IBM): A pilot study

#### Bhaskar Roy^1^, Seward B. Rutkove^2^, Richard J. Nowak^1^

##### ^1^Yale School of Medicine, New Haven, USA; ^2^Beth Israel Deaconess Medical Center, Boston, USA

###### **Correspondence:** Bhaskar Roy

Objective: To assess muscle impedance parameters as potential biomarker of inclusion body myositis (IBM).

Background: There is an unmet need for an objective, easily applicable biomarker to assess IBM disease severity. Electrical impedance myography (EIM), a noninvasive technique, has shown promise as a potential biomarker in several neuromuscular disorders. This pilot study is evaluating the potential of EIM as a biomarker of IBM (clinicaltrials.gov || NCT03633318).

Design/Methods: We intend to recruit 20 patients with clinicopathologically confirmed IBM and 12 healthy controls. We present a preliminary analysis of 5 IBM patients (4 men, 1 woman; mean age 66 years) enrolled since August 2018. Healthy control data was not yet available to analyze. Each subject went through manual muscle testing (MMT) in neck flexion/extension, and 15 extremity muscles on both sides (maximum MRC score of 160), 6-minute walk (6MW), handgrip dynamometry, IBM-functional rating scale (IBM-FRS), and EIM measurements of deltoid, biceps, forearm flexors, rectus femoris, tibialis anterior, and medial gastrocnemius. Spearman rank correlation was used.

Results: Mean ± SD of MMT, grip strength (kg; Right/Left), IBM-FRS, 6MW (feet) were 130.75/160 ± 15, 9.8 ± 5/8.4 ± 5.4, 28.6 ± 7, and 1187 ± 480 respectively. A strong correlation was noted between averaged EIM-50 kHz and averaged ratio of EIM 200 kHz/50 kHz (EIM-200/50) from six muscles with IBM-FRS and 6MW distance (absolute rho ≥ 0.9, p-value ≤ 0.001). Hand grip strength showed moderate correlation with EIM-50 kHz from forearm muscles: rho 0.66, p-value 0.037. At individual muscle level, a moderate correlation was noted between EIM-50 kHz, EIM-200/50 and the weak muscles; rho values 0.58, -0.59 respectively with p-values < 0.001.

Conclusion: Preliminary results suggest that EIM may be a potential IBM biomarker and useful clinical trial outcome measure. These results should be interpreted carefully given the small sample size to date and further validation studies required. Our study remains open to enrollment with additional data available for presentation forthcoming.

### P118 Clinical description of familial inclusion body myositis and molecular genetic analysis

#### Akatsuki Kubota^1^, Hiroyuki Ishiura^1^, Jun Mitsui^1^, Jun Shimizu^1^, Shoji Tsuji^2^, Tatsushi Toda^1^

##### ^1^University of Tokyo, Tokyo, Japan; ^2^International University of Health and Welfare, Otawara, Japan

###### **Correspondence:** Akatsuki Kubota

Objective: Inclusion body myositis (IBM) is a chronic progressive myopathy with inflammatory and degenerative features, and its cause remains unknown. IBM is generally considered as a sporadic disease, but there are rarely familial cases. Here, we report a familial case of IBM.

Methods: The proband and his cousin suffered from middle-age onset, chronic progressive muscle weakness. Both patients were examined, and histopathological analysis of biopsied muscle specimens was performed. DNA samples were collected from the two IBM patients, and two healthy brothers of the proband. Given the pedigree tree, assuming X-linked inheritance, we conducted single nucleotide polymorphism (SNP) genotyping of the pedigree members.

Results: Both patients showed characteristic finger flexor weakness with moderately increased serum creatine kinase levels. Muscle biopsy showed cytotoxic T cells surrounding and sometimes invading non-necrotic myofibers, and inclusion bodies. These findings lead to diagnosis of clinicopathologically defined IBM for both patients. SNP typing showed that approximately 1.1Mb at Xp22 were shared exclusively by the two patients.

Conclusions: Further genetic analysis is needed to explore the causative gene for familial IBM.

### P119 DMF diminishes inflammatory response in muscle cells

#### Karsten Schmidt, Alice Faust, Simranjeet Kaur, Peter Balcarek, Jens Schmidt

##### Universitätsmedizin Göttingen, Göttingen, Germany

###### **Correspondence:** Karsten Schmidt^1^

Objective: This study aims to evaluate the effect of dimethylfumarate (DMF) in in vitro and in vivo model systems for inclusion body myositis (IBM). Background: The pathogenesis of IBM is caused by degenerative and inflammatory mechanisms. Various immunosuppressive therapies have failed to show a significant beneficial effect in clinical studies. DMF is an anti-inflammatory drug used in multiple sclerosis and psoriasis. It is known to diminish the cellular stress response to proinflammatory stimuli through activation and nuclear translocation of NRF-2.

Methods: The effect of DMF was studied in vitro in a well-established proinflammatory cell culture model system originated from a myoblast cell line and cultured primary muscle cells on RNA and protein level using quantitative PCR, immunocytochemistry and western blotting. For in vivo studies a previous characterized double-transgenic mouse strain (APP/PS1) was used. The mice were challenged with a proinflammatory stimulus via injection of LPS in the calf. Quantitative PCR, immunohistochemistry, FACS-analysis were performed to evaluate the effect of DMF on NRF-2 expression and translocation on cell stress and inflammatory response.

Results: In cell culture we found that DMF led to an upregulation and nuclear translocation of NRF-2 in rhabdomyosarcoma cells. Furthermore, DMF diminishes the inflammatory changes in the mouse model of IBM (APP/PS1 transgenic mice) after intramuscular injection with LPS. However, expression analysis revealed partly unaltered proinflammatory cytokines in DMF treated mice compared to non-treated, suggesting a rather focused therapy effect than an overall immunosuppressive effect.

Conclusion: We are able to show a molecular effect of DMF in muscle cells by translocation of NRF-2 and upregulation of associated genes. Secondly, we could show a significant reduction of inflammation under treatment with DMF in double-transgenic mice (APP/PS1) injected intramuscular with LPS.

### P120 Nonspecific pattern of muscular cN-1A expression in inclusion body myositis

#### Anke Rietveld, Thomas Oosterhof, Saskia Lassche, Baziel van Engelen, Benno Küsters, Christiaan Saris, Ger Pruijn

##### Radboud University, Nijmegen, Netherlands

###### **Correspondence:** Anke Rietveld

Objective: Inclusion body myositis (IBM) is a slowly progressive acquired myopathy with inflammatory and myodegenerative features. Autoantibodies against cytosolic 5’-nucleotidase (cN-1A) are present in 33-76% of IBM patients. We investigated the pattern of cN-1A expression in healthy and diseased muscle tissue to gain more insight in the mechanisms that result in anti-cN-1A autoreactivity in IBM.

Methods: cN-1A was visualized in muscle tissue of IBM patients by immunohistochemical and immunofluorescent staining. Healthy individuals and patients with facioscapulohumeral dystrophy and oculopharyngeal muscular dystrophy were used as controls (n = 8 in each group). cN-1A expression was semi-quantitatively compared between these groups and related to other histopathological features such as COX-negativity and inflammatory changes.

Results: Immunohistochemical cN-1A staining showed predominant cN-1A expression in type 2 myofibers in all groups. Regenerating fibers occasionally showed cN-1A expression, irrespective of the disease. Rimmed vacuoles were present in one biopsy of an IBM patient, showing cN-1A expression around these rimmed vacuoles. Immunofluorescent cN-1A staining showed perinuclear cN-1A accumulation in all groups.

Interpretation: cN-1A is predominantly expressed in type 2 glycolytic myofibers and around myonuclei. These findings are not IBM-specific. The occasional expression of cN-1A in regenerating fibers in all groups encourages further research to clarify whether the presence of regenerating fibers is related to the development of anti-cN-1A autoreactivity.

### P121 The German patient registry for inclusion body myositis

#### Sabine Krause, Simone Thiele, Marcel Heidemann, Karsten Schmidt, Matthias Vorgerd, Jens. Schmidt, Maggie C. Walter

##### Friedrich Baur Institute, Munic, Germany

###### **Correspondence:** Sabine Krause

Objectives: Inclusion body myositis (IBM) is a quite common form of myositis in patients aged over 50 years. It is a rare disease with an estimated prevalence between 4.5 and 9.5/million rising up to 50/million for individuals over 50 years with clinical heterogeneity in different populations and ethnic groups. Patients suffer from slowly progressive muscle weakness resulting in severe disability and display varying degrees of dysphagia. No causative treatment is currently available but new therapeutic strategies are investigated and innovative clinical trials are expected in the coming years. A German patient registry for IBM was established to overcome the bottlenecks of data fragmentation, lack of harmonization and lack of trial readiness for this disease. We aim to include harmonized datasets of German patients diagnosed with IBM based on standardized clinical and histopathological findings (revised Hilton–Jones criteria).

Methods: A harmonized dataset including clinical data and muscle biopsy reports is recorded since 01/2016 using a dual patient and professional online report system (www.ibm-register.de). National legislation, data protection laws and ethical recommendations are strictly followed in this process. The registry aims at collecting high-quality, curated and regularly updated longitudinal data of the majority of German IBM patients. In addition, the registry may help to address research questions like prevalence, natural history and disease course.

Results: Currently, the registry is being used to evaluate up-to-date epidemiological data. In particular, the occurrence of swallowing disorder and the cN-1A autoantibody are being assessed.

Conclusions: The IBM registry provides the unique opportunity not only to characterize and effectively recruit German patients for international clinical trials and to support the translation of new therapies from “bench to bedside”, but also to improve patient care by assessing standards of diagnosis and care.

### P122 Inclusion Body Myositis (IBM) Symptom Flares and Associated Impact from the Patient Perspective

#### Antonis Kontekakis, Winnie Nelson^1^, Lisa Christopher-Stine^2^, William Kelly^2^, Linda Kobert^3^, Barima Opong-Owusu^1^, Michael Reed^4^

##### ^1^ Mallinckrodt Pharmaceuticals; Mountain View, USA; ^2^ Johns Hopkins University; Baltimore, USA; ^3^ The Myositis Association; Alexandria, USA; ^4^ Vedanta Research; Chapel Hill, USA

###### **Correspondence:** Antonis Kontekakis

Objectives: IBM is a progressive and chronic inflammatory myopothy in which flares, or worsening symptoms, have not been well characterized from the patient perspective. This analysis explores IBM symptom flare frequency, corresponding disability and rate of unplanned medical encounters from patient self-report.

Methods: Online surveys were collected from volunteer patients from The Myositis Association and Johns Hopkins Myositis Center. Sociodemographics, flare symptoms, Health Assessment Questionnaire Disability Index (HAQ-DI) and HAQ-Pain index, Patient Health Questionnaire (PHQ-4), emergency department and urgent care (ER/UC) visits and hospital admissions were assessed. Flare frequency was obtained by asking, “Have you ever had a flare or worsening myositis symptoms?” and if yes, “How many times in the past 12 months?”

Results: 233 individuals with a self-reported diagnosis of IBM completed the survey between December 2017 and May 2018. Recall of prior symptom flares was reported by 187 respondents (97.2% Caucasian, 31% female, 17.1% employed, mean age 68.9 years). Among the 187 respondents, 48 (25.7%) reported never having flares or no flares in the past year, 69 (36.9%) reported 1-3 flares, 35 (18.7%) reported 4-6 flares, and 35 (18.7%) reported ³ 7 flares in the past year. Among the 139 respondents with self-reported flares in the past year, the most common worsened symptoms were muscle weakness (96%), trouble standing from a seated position (71%), trouble climbing stairs (67%), extreme fatigue (56%) and dysphagia (51%). Increasing flare frequency was associated with marginally significant HAQ-DI (F=2.177, P < .092) and significantly greater mean HAQ-Pain (F=13.846, P < .001) as well as more PHQ anxiety (χ2=10.862, P < .012) and depression (χ2=7.061, P < .071). 15.5% reported past year ER/UC use, and 8.6% reported one or more hospital admissions, but neither was associated with flare frequency, perhaps due to the chronic and slowly progressive nature of IBM.

Conclusion: Despite being rarely described in the literature, self-reported symptom flares or periods of worsening symptoms were found to be common among patients with IBM, with 74% reporting one or more occurrences in the past year. Muscle weakness was the most common flare symptom, and dysphagia occurred in half of the patients during a flare. Flare frequency was associated with greater pain and more disability as well as greater risk of clinical anxiety and depression. Higher frequency of patient-reported flares may be a marker of worse physical and mental functioning and supports their importance in clinical assessment of IBM.

### P123 Characterization of inflammation-induced mitochondrial cell stress in human skeletal muscle in vitro and ex vivo

#### Stefanie Meyer^1^, Leila Scholle^2^, Sabrina Zechel^1^, Susann Kummer^3^, Jan Dudek^4^, Heiko Lorenz, Anna Hell^1^, Christine Stadelmann^1^, Stephan Zierz^2^, Jana Zschüntzsch^1^, Jens Schmidt^1^

##### ^1^Universitätsmedizin Göttingen, Göttingen, Germany, ^2^Universitätsklinikum Halle, Halle, Germany; ^3^Universitätsklinikum Heidelberg, Heidelberg, Germany; ^4^Universitätsklinikum Würzburg, Würzburg, Germany

###### **Correspondence:** Stefanie Meyer

Objectives: Inclusion body myositis (IBM) is a relatively common inflammatory myopathy (IM) in patients above fifty years of age. Typical finding in IBM muscle histopathology includes an autoreactive inflammation, degenerative changes and marked alterations of mitochondria. These include an abnormal mitochondrial proliferation, multiple deletions in the mitochondrial DNA and dysfunction of the cytochrome C oxidase (COX), a protein-complex of the mitochondrial respiratory chain. The role of these abundant mitochondrial alterations in IBM compared to other IM remains elusive. We hypothesized that inflammatory cell stress leads to mitochondrial dysfunction in human muscle cells and, thereby, causes muscle fiber damage and wasting.

Methods: Cell culture of primary myotubes from human non – myopathic muscle samples were exposed to pro-inflammatory cytokines such as interferon-gamma (IFN-γ) and interleukin-1beta (IL-1β). Relevant markers of fusion (Mfn1, Mfn2), fission (Drp1, Fis1), respiratory chain (COX IV) and transporter of the outer mitochondrial membrane (TOM20) were analyzed by qPCR, Immunocytochemistry and Western Blot. The enzyme activity of complexes of the respiratory chain was assessed using spectrophotometric analysis. Muscle biopsies of patients with IBM, polymyositis (PM), dermatomyositis (DM), necrotizing myopathy (NM) and muscular dystrophies were studied for inflammatory and mitochondrial changes by qPCR and Immunocytochemistry.

Results: After exposure to inflammatory cytokines, a significant increase of the Drp1 - and Fis1- expression in the cultured cells was noted as well as an increased fission of the mitochondrial network as evidenced by TOM20 staining. Additionally, a significant decrease of the COXIV – protein amount upon pro-inflammatory cytokines was noted. IBM muscle biopsies displayed a significant increase of COX – deficient fibers, ragged red fibers and rimmed vacuoles, which confirmed IBM by means of histopathology. In all IBM biopsies, an increased expression of Fis1 and Drp1 was detected in comparison to non– myopathic muscle.

Conclusion: Using an established cell culture model for myositis, the results demonstrate that pro-inflammatory cytokines lead to a shift of mitochondrial dynamics towards fission and decreased expression of respiratory chain proteins. Ex vivo data of IM patients is in line with the in vitro findings, showing signs of increased fission processing. The data offer an explanation how myoinflammation can lead to muscle fiber damage through alterations of mitochondrial dynamics and consecutive dysfunction. These findings could be relevant for developing new therapeutic strategies e.g. mitochondrial protective molecules, to reduce the burden of chronic muscle inflammation.

### P124 Fingolimod and IgG in experimental treatment of an IBM mouse model for muscle regeneration

#### Per-Ole Carstens, Sebastian Höller, Alexis Grimm, Jens Schmidt

##### Universitätsmedizin Göttingen, Göttingen, Germany

###### **Correspondence:** Per-Ole Carstens

Objectives: Inclusion body myositis (IBM) is an autoimmune-mediated inflammatory myopathy, which is resistant to immunosuppressive treatment. The complex pathophysiology is characterized by mechanisms of inflammation, degeneration and an impaired regenerative capacity. Our aims were i) to establish a model of muscle regeneration in IBM mice and ii) to analyze the effects of fingolimod and immunoglobulins (IgG) on regeneration.

Methods: As an animal model, double transgenic mice with a muscle specific overexpression of amyloid precursor protein (APP, Swedish mutation) and a mutated presenilin, leading to an IBM-like phenotype were used (hereinafter referred to as “IBM mice”). Muscle damage was induced by the injection of the venom notexin into the right tibialis anterior muscle. The left muscle was treated with saline as a solvent control. 14 animals were treated with fingolimod, which was administered daily via the drinking water (dose: 3 mg/kg). 16 animals were treated with IgG injected intraperitoneally at day 0 and 1 (dose: 1 g/kg). 30 animals remained untreated. For clinical assessment, mice were scored daily by a score adapted from the experimental autoimmune myositis scale, which ranges from 5 (paralysis of the complete hind leg) to 0 (normal). Additionally, the grip strength was measured daily. At day 10, all animals were sacrificed and the tibialis muscle of both sides were analyzed. The muscle regeneration and necrosis were analyzed by histology and the levels of mRNA expression for markers of regeneration (myogenin, MyoD, PAX7), inflammation (IL-1β, CXCL-9) and atrophy (Trim63) were measured by qRT-PCR.

Results: The clinical scoring and grip strength displayed no differences between treated and untreated animals. Both groups had an initial mean score of 4 after the notexin injection and improved to a score around 1 at day 10. Histologically, the degree of regeneration and necrosis was comparable in both treatment groups compared to controls and analysis by qRT-PCR did not produce relevant differences.

Conclusions: Impaired muscle regeneration is a relevant part of the complex pathophysiology of IBM. We were able to establish a model of muscle regeneration in the IBM mouse, but our treatment attempts failed. Reasons could be, that the treatment start is at the same time point as the muscle damage, which might be to late. Another explanation could be, that IgG and fingolimod are generally not operative in regenerative pathways in the skeletal muscle. More studies are needed to further elucidate novel pharmacological approaches in experimental regeneration in mouse muscle.

### P125 Clinical mimics of inclusion body myositis and how to recognize them

#### Stefanie Glaubitz, Rachel Zeng, Karsten Schmidt, Per-Ole Carstens, Mateja Smogavec, Jens Schmidt, Jana Zschüntzsch

##### Universitätsklinikum Göttingen, Göttingen, Gemany

###### **Correspondence:** Stefanie Glaubitz

Background: Inclusion body myositis (IBM) is an idiopathic inflammatory myopathy with typical clinical symptoms and histological features. However, due to a range of acquired or genetically determined myopathies with similar presentations, IBM is often missed and diagnosed in error as another neuromuscular disease.

Objective: To discuss and identify clinical pitfalls in the diagnosis of IBM by means of case reports.

Methods: We provide typical case reports of facioscapulohumeral muscular dystrophy (FSHD), titinopathy (TMD/LGMD2J), anoctaminopathy (LGMD2L), polymyositis and other neuromuscular diseases that can mimic IBM. The data are carefully discussed and typical clinical pitfalls are summarized.

Results: Along with dermatomyositis, polymyositis, overlap myositis and (immune mediated) necrotizing myopathy, IBM belongs to the group of idiopathic inflammatory myopathies (IIM). The typical clinical presentation of IBM involves slowly progressive muscle atrophy and weakness with a preference of long finger flexors, knee and foot extensors, often with an asymmetric distribution. Other symptoms may include myalgia and dysphagia. The diagnosis of IBM is currently based on a defined composition of clinical, serological and muscle histologic criteria. Additionally, electromyography, muscle MRI and muscle ultrasound may help to confirm the diagnosis. A muscle biopsy is required to diagnose a clinico-pathologically defined IBM, showing the co-existence of myodegenerative and inflammatory pathologic features. Nevertheless, the diagnosis of IBM or especially the differentiation from other neuromuscular diseases can be difficult due to atypical clinical presentations. Histopathological findings can also lead to confusion since inflammatory infiltrates can also be found in hereditary myopathies like dysferlinopathy or facioscapulohumeral dystrophy. The myodegenerative characteristics of myofibrillar myopathies like vacuoles and abnormal accumulations of filamentous proteins can also be mistaken.

Conclusion: IBM is often missed and diagnosed in error as another neuromuscular disease. However, careful consideration of clinical and histopathological features can help to differentiate IBM from other neuromuscular diseases and lead to the correct diagnosis. Only a correct and reliable diagnosis of IBM allows for recruitment to clinical studies and helps to find novel, exploratory treatment modalities and prevent unnecessary treatments.

### P126 Clinical profile of inclusion body myositis/myopathy (IBM) in a referral neuromuscular center

#### Payam Soltanzadeh^1^, Nimish Thakore^2^, Richard Prayson^2^, Kerry Levin^2^

##### ^1^UCLA, Los Angeles, USA; ^2^Cleveland Clinic, Ohio, USA

###### **Correspondence:** Payam Soltanzadeh

Objectives: Clinical overview of a large series of inclusion body myositis/myopathy (IBM) patients from a referral neuromuscular center.

Methods: We identified patients with the diagnosis of inclusion body myositis/myopathy (IBM) who were referred to the Cleveland Clinic Neuromuscular Center in Cleveland Ohio (USA) between February 2012 and October 2017. Griggs, ENMC, and MRC criteria were used to stratify patients into different diagnostic categories based on clinical, electrodiagnostic, and pathologic findings. Creatine kinase (CK) levels, age of onset, initial symptoms or presenting features, major electromyographic (EMG) findings, and relevant laboratory data were also collected.

Results: One hundred and seven (107) patients were identified among whom 95 were still alive at the time of study enrollment. Fifty-one patients had detailed biopsy findings consistent with the diagnosis of sporadic IBM (sIBM), among whom one had HIV infection and one had chronic hepatitis C disease. Among the 49 pathologically “pure” sIBM, 21 had “definite” sIBM per Griggs criteria, 38 had “definite” sIBM per ENMC criteria, and 26 had “clinically defined” sIBM per MRC criteria. In patients with ENMC definite sIBM, 23 (60%) presented with proximal leg weakness and two patients with asymptomatic hyperCKemia. Irritable myopathy with mixed neurogenic and myopathic motor units was the most common electrodiagnostic pattern in patients with sIBM. Irritable myopathy in forearm flexor muscles was commonly noted in patients with sIBM. Fragile X-associated tremor and ataxia syndrome (FXTAS), VCP proteinopathy, and GNE myopathy were the genetic muscle diseases mimicking sIBM.

Conclusion: Diagnostic certainty of sporadic inclusion body myositis (sIBM) varies depending on what criteria are used. More patients are now diagnosed with sIBM based on clinical and EMG criteria without getting muscle biopsies. Infectious and genetic muscle diseases can mimic sIBM and asymptomatic hyperCKemia could also be a presenting feature.

### P127 Characterising the immune response associated with Inclusion Body Myositis

#### Jerome Coudert^1^, Kelly Beer^1^, Phillipa Lamont^2^, Merrilee Needham^1^

##### ^1^Murdoch University, Murdoch, Australia; ^2^Royal Perth Hospital, Perth, Australia

###### **Correspondence:** Jerome Coudert

Background: Inclusion body myositis (IBM) is the most common acquired skeletal muscle disease associated with aging and occurs more commonly in men. It is relentlessly progressive over time resulting in muscle wasting and loss of function, which negatively impacts patient mobility and quality of life. Histologically, IBM is characterised by a combination of inflammatory and degenerative changes. Immune cells accumulate within the endomysium, and surround and invade the myofibres. They are mainly composed of T-cells and macrophages. In addition, myofibers express upregulated MHC class I molecules on their surface thereby behaving as antigen presenting cells, promoting antigen presentation to and targeting by cytotoxic CD8+ T cells. The biological mechanisms responsible for IBM aetiology appear to be quite unique, and as a result, conventional immunosuppressive treatments which are used in other inflammatory myopathies are not effective and often even have deleterious effects. Objectives: A detailed characterisation of the autoimmune response in IBM over time is therefore necessary to understand the cellular and molecular basis of this dysregulation and to design more targeted therapies.

Methods: We undertook to analyse the immune system in the blood of our patients using a flow cytometry-based approach, in order to identify changes occurring over time. In addition, we analysed the T-cell receptor (TCR) variability; these molecules are generated upon rearrangement of the genes that encodes the TCR region. Each T-cell possesses a unique gene rearrangement and accumulation of cells expressing identical TCR demonstrates clonal expansion in response to a particular antigen. The CDR3 is a highly variable domain of the TCR and it constitutes the molecular basis for T-cell specificity. We sequenced the TCR of T-cell subsets isolated from the blood and from diseased muscles to identify potential predominant TCR gene rearrangements and CDR3.

Results: The flow cytometry analysis revealed high frequencies of circulating activated / memory T lymphocyte subsets with evidence of inflammatory and cytotoxic functions. The presence of inflammatory macrophages was also evident. The analysis of TCR variability using next generation sequencing revealed clonally expanded T cells both within muscles and in the blood.

Conclusion: We expect that our work using newly available technologies will provide critical information building on previous studies, to understand what drives the autoimmune response associated with IBM and will help identifying the antigen responsible for the autoreactive T cells, overall paving the way toward more targeted and successful therapies.

### P128 Association of HLA with anti-cN1A antibodies in IBM patients

#### Jerome Coudert^1^, Kelly Beer^1^, Christine Bundell^2^, Anna Brusch^2^, Abha Chopra^1^, Merrilee Needham^1^

##### ^1^Murdoch University, Murdoch, Australia; ^2^PathWest, Nedlands, Australia

###### **Correspondence:** Jerome Coudert

Background: Inclusion body myositis (IBM) is the most common acquired skeletal muscle disease associated with aging. It is relentlessly progressive over time resulting in muscle wasting and loss of function, which has a major impact on their quality of life and ability to live independently. Histologically, IBM is characterised by a combination of inflammatory and degenerative changes. Immune cells accumulate within muscle tissues, surround and invade the myofibres; they are mainly composed of T-cells as well as macrophages. The local production of inflammatory cytokines and chemokines further recruits and stimulate leucocytes, while exacerbating the degenerative process in myofibres manifesting as protein accumulation. Recent findings revealed the presence of autoantibodies in many patients, suggesting that B-cells may also play a role in IBM pathogenesis. We found in one third of our IBM patients antibodies against cytosolic 5’-nucleotidase 1A (cN1A). cN1A is a cytosolic enzyme involved in the conversion of adenosine monophosphate (AMP) to adenosine and catalyses the hydrolysis of nucleotides to nucleosides. cN1A is most abundant in skeletal muscles. In IBM, it is aberrantly distributed within muscle in areas of myofibres degeneration. However anti-cN1A is rarely detected in other forms of inflammatory myopathies, suggesting it could have potential as a biomarker in this clinical context of myositis. Even though the precise role of anti-cN1A remains uncertain in IBM, results obtained from experimental animal models suggests that these antibodies may have a pathogenic role in myofibre degeneration.

Objectives: We hypothesised that the production of anti-cN1A in IBM patients was associated with genetic factors such as HLA allotype.

Methods: We analysed samples from IBM patients that are both positive and negative for the anti-cN1A antibodies. Serum samples were analysed by ELISA for the presence of anti-cN1A antibodies while DNA isolated from blood cells was sequenced by Illumina next generation sequencing to define the HLA haplotype of these patient groups.

Conclusion: Knowledge of association of HLA carriage with anti-cN1A production may further improve the diagnostic power and specificity of anti-cN1A antibody testing for IBM, whilst requiring only a slightly larger blood sample rather than requiring a far much invasive muscle biopsy. If future studies confirm that these antibodies are not only biomarkers but also mediate muscle damages, HLA typing will provide a predictive test before circulating anti-cN1A reach conclusive levels; which may support the implementation of antibody-specific treatments.

### P129 Reliability and validity of an activity limitation measure in persons with IBM

#### Malin Regardt^1^, Lisa Christopher Stine^2^

##### ^1^Karolinska University Hospital, Stockholm, Sweden; ^2^John Hopkins University, Baltimore, USA

###### **Correspondence:** Malin Regardt

Objective: The aim of this study was to test validity and reliability of the questionnaire Disability in the Arm Shoulder and Hand (DASH) for patients with IBM. A second aim was to describe activity limitation measured by the Canadian occupational Performance measure (COPM).

Method: Persons diagnosed with IBM were identified through the Swedish Myositis Network (SweMyoNet) quality registry in Stockholm Sweden. A total of 36 persons with IBM were included in the registry and were invited to participate. A total of 17 men and 9 women agreed to participate. Median (Q1-Q3) age was 74 (70-79) years and the median (Q1-Q3) disease duration was 7 (3-8) years. Activity limitation were assessed by the questionnaire Disability of the Arm, Shoulder and Hand (DASH) and the The Canadian occupational performance measure (COPM) which investigate patient derived areas of daily activities. The data collection was performed at the Karolinska university hospital in Stockholm Sweden. At baseline both DASH and COPM were performed. The participants received a second DASH questionnaire to be answered within two weeks (Follow-up) and send back to the researcher.

Results: There were good correlations between baseline measure and follow-up on DASH (rs 0.997; p=0.01) Indicating that the DASH is consistent over a short period of time. The results from COPM showed a variety of activities persons with IBM experienced problem with. Area with most activity limitations were basic self-care area such as dressing and grooming, fall, feeding, managing communication. Instrumental activities such as managing instruments, shopping and meal preparation. Leisure activities such as playing an instrument, run, paint and social activities such as visit friends, social engagements. Some of these activities were found in the DASH but not all. E.g. missing socializing with friends and family, problems swallowing or were environment dependent

Conclusion: The results indicate that DASH have a good test re-test reliability DASH includes some of the activities that persons with IBM experience difficulties with but not all. The participants experienced difficulties in all areas of life.

### P130 B cell mechanisms in chronic muscle inflammation

#### Per-Ole Carstens^1^, Luisa Müllar^1^, Arne Wrede^2^, Martin Wachowski^1^, Almuth Brandis^3^, Sabine Krause^4^, Stephan Zierz^5^, Jens Schmidt^1^

##### ^1^University Medical Center Göttingen, Göttingen, Germany; ^2^Saarland University Medical Center, Saarland, Germany; ^3^Klinikum Region Hannover, Hannover, Germany; ^4^Ludwig-Maximilians-University of München, Munic, Germany; ^5^University Hospital Halle/Saale, Halle, Germany

###### **Correspondence:** Per-Ole Carstens

Objectives: Inclusion body myositis (IBM) and polymyositis (PM) are autoimmune-mediated myopathies, typically with a chronic disease course and development of paresis and impaired ambulation. The pathophysiology is complex and characterized by mechanisms of inflammation with predominance of T cells and macrophages. Beside these mechanisms, several autoantibodies have been identified in myositis and point towards a relevant involvement of B-cell-immunity. Our aim was to identify B-cell-mediated immunomechanisms in myositis. We studied the B cell activating factors BAFF and APRIL and the chemokines that control recruitment of B cells in vitro and ex vivo in IBM and PM compared to muscular dystrophies and non-myopathic controls.

Methods: Muscle biopsy specimen from patients with IBM (n=9), PM (n=9), muscular dystrophy (n=9) and non-myopathic controls (n=9) were analyzed for mRNA-expression of BAFF, APRIL, CXCL-12 and CXCL-13 and serial sections of skeletal muscle biopsies were stained for BAFF and CXCL-12. Myotubes derived from patients without myopathic changes were exposed to an inflammatory environment and the mRNA expression as well as the protein expression of BAFF, APRIL, CXCL-12 and CXCL-13 were measured by qPCR and by immunocytochemistry.

Results: The mRNA-expression of BAFF, APRIL and CXCL-13 was significantly higher in IBM and PM compared to healthy controls. In IBM, also the mRNA-expression of CXCL-12 was significantly higher. Muscles of patients with IBM displayed the largest proportion of positive stained muscle fibers for BAFF and CXCL-12. The exposure of myotubes to IFN-γ or IL-1β, respectively, led to a significant mRNA upregulation of BAFF and CXCL-12. The expression of APRIL and CXCL-13 was not altered by these cytokines. Immunocytochemistry showed a trend for upregulated protein-levels of BAFF and CXCL-12 in human myotubes.

Conclusions: Our results substantiate the hypothesis of an involvement of B cell-associated mechanisms in the pathophysiology of IBM and PM. The muscle fibers themselves may participate in the survival and differentiation of B cells and maintain the inflammation.

### P131 Active immunization mice model of sporadic inclusion body myositis

#### Nozomu Tawara

##### Graduate School of Medical Sciences Kumamoto University, Kumamoto, Japan

Objective: Sporadic inclusion body myositis (sIBM) is the most frequent acquired inflammatory myopathy in patients older than 50 years of age. Lack of effective treatments and unclearness of pathogenesis in sIBM may be associated with absence of appropriate animal models. Recently, an autoantibody against cytoplasmic 5’-nucleotidase 1a (cN1A) was identified in the sera of patients with sIBM. In the current study, we aimed to generate the active immunization mice model using with cN1A peptides to realize the pathogenesis of sIBM.

Methods: Three different sequences of cN1A peptides (P1, P2, and P3) with complete Freund’s adjuvant (CFA) were injected into the bilateral foot pads of C57BL/6J mice (n= 5 per each peptide). Pertusis toxins (PT) were injected intraperitoneally at the same time. Injection of peptides and CFA were repeated 3 times every week. As control mice, CFA and PT were injected alone. We evaluated time course of body weight, motor activity, myopathological changes and protein expression levels of autophagy relating proteins.

Results: The mice injected with cN1A peptide showed significant differences in motor activity. Myopathological analysis revealed inflammatory cellular infiltrates in the peptide injected mice. Westernblotting analysis showed that p62 was upregulated in peptide injected mice.

Conclusions: The mice immunized with specific cN1A peptide can mimic partly clinicopathological phenotypes of sIBM. cN1A active immunization mice can be an appropriate animal model to elucidate pathogenesis and to search effective treatment of sIBM.

### P132 Unravelling the pathogenesis of brachio-cervical myopathy associated to Scleroderma

#### Xavier Suárez-Calvet, Eduard Gallardo, Jorge Alonso-Pérez, Ivan Castellví, Ana Carrasco-Rozas, Alicia Alonso-Jimenez, SusanaLópez-Fernández, Joana Turón-Sans, Ricard Rojas-García, Jordi Díaz-Manera

##### ^1^Sant Pau Biomedical Research Institute, Barcelona, Spain

###### **Correspondence:** Xavier Suárez-Calvet

Objectives: To describe patients with brachio-cervical myopathy associated to Scleroderma from a clinical, serological and histological point of view. To elucidate specific pathogenic events occurring in these patients using in vitro experiments.

Methods: We reviewed clinical, immunologic, muscle MRI, capillaroscopy, muscle biopsy and response to treatment data from the patients. Cytokine arrays were performed using sera from age and sex-matched controls, scleroderma patients and from our cohort. In vitro, we analyzed the effect of dysregulated molecules on human cells that are presumably involved in the pathogenesis of this disease such as muscle cells, fibroblasts, endothelial cells and fibroadipogenic progenitors.

Results: Herein we describe 11 patients with brachio-cervical inflammatory myopathy, 10 women and one man. Symptoms at onset were neck and proximal upper limbs muscle weakness with relative sparing of lower limbs. Mean age of symptoms onset was 51.2 years old. All patients had associated features of scleroderma, like thickening of the skin, sclerodactylia, facial telangiectasias or Raynaud's phenomenon. Dysphagia was present in 72.2% of patients and lung involvement in 36.6% of patients. Capillaroscopy showed a scleroderma pattern in six patients. Antibody tests were negative except for some patients who presented different reactivities (one ACA-B, one SSA/Ro, two Th/To and one anti-Ku and anti-Mi2). Muscle biopsies showed fiber size variability, necrosis, fibrosis and increased expression of MHC class I. A variable degree of macrophage infiltration was observed, and some biopsies showed CD20, CD4 and CD8 infiltrates and complement deposition. Cytokine arrays showed dysregulated cytokines involved in fibrosis, inflammation and angiogenesis. We are investigating in vitro whether the dysregulated molecules present in the sera of patients reproduce our in vivo observations. Ongoing studies of proliferation, migration, differentiation and gene expression will elucidate the specific role of these molecules in the pathogenesis of this particular form of myositis.

Conclusion: We present a significant number of patients with a poorly reported association between brachio-cervical myopathy and scleroderma which is manifested as a form of myositis. It is imperative to consider this entity because a correct diagnosis has important implications for treatment. Expanding the knowledge of this entity will help to understand the specific mechanisms implicated in the muscle degeneration observed.

### P133 Vacuolar myopathy with monoclonal gammapathy and stiffness: A new Monoclonal gammopathy of muscle significance

#### Yves Allenbach^1^, Emmanuelle Salort-Campana^2^, Edoardo Malfatti^3^, Bruno Eymard^4^, Shahram Attarian^2^, Andre Maues de Paula^2^, Aude Rigolet^4^, Sarah Leonard-Louis^4^, Olivier Benveniste^4^, Tanya Stojkovic^4^

##### ^1^Sorbonne Universités, Paris, France; ^2^Assistance Publique-Hopitaux Marseille, Marseille, France; ^3^Assistance publique–Hôpitaux de Paris, Paris, France; ^4^Hôpital Pitié Salpêtrière, Paris, France

###### **Correspondence:** Yves Allenbach

Objective: Monoclonal gammopathy have been related with numerous neurological disorders but the spectrum of associated myopathies remains poorly described. We report a new acquired myopathy associated with monoclonal gammopathy.

Methods: Three patients were prospectively analysed. In addition to patients’ characteristics, electrophysiological data, muscle biopsy analysis and outcomes of patients were collected.

Results: Three patients aged from 38 to 56 years were analysed. All suffered from a muscle weakness with a sub-acute onset and stiffness, in a context of severe weight loss. The muscle deficit mainly involved the proximal limbs and axial muscles. Creatine kinase level was increased [1400-2900 I.U/L] and electromyography revealed a myogenic pattern and spontaneous high frequency discharges. Muscle biopsies showed the association of vacuoles filled with glycogen and mild inflammation. There was no evidence for genetic glycogen metabolic disorder. IgGκ monoclonal gammopathy was identified in all cases. There was no sign of lymphoplasmocytic proliferation. All patients improved with a treatment combining corticosteroid, intravenous immunoglobulin and immunosuppressants, and dramatic improvement was observed in two patients.

Conclusion: we reported a new monoclonal gammopathy associated muscle disease defined by a vacuolar myopathy characterized by axial and proximal muscle weakness with prominent stiffness and high frequency discharges on electromyography.

### P134 Characteristics of myositis - rheumatoid arthritis overlap patients in a Hungarian cohort

#### Levente Bodoki^1^, Zoltán Griger^1^, Melinda Nagy-Vincze^1^, Zoe Betteridge^2^, Katalin Dankó^1^

##### ^1^University of Debrecen, Debrecen, Hungary; ^2^Bath Institute for Rheumatic Diseases, Bath, UK

###### **Correspondence:** Levente Bodoki

Overlap syndromes are autoimmune disorders in which classification criteria of at least two connective tissue diseases (CTDs) are fulfilled. The Division of Clinical Immunology in Debrecen, Hungary oversees patients with idiopathic inflammatory myopathies (IIMs) since the 1980’s. In a work called The Myositis Antibody Research Project, which is coordinated together with the Bath Institute for Rheumatic Diseases (UK), authors investigated 330 patients with myositis, all treated in this Hungarian center. The aim of this retrospective study was to investigate the frequency, clinical and serological parameters and therapeutic options in myositis – rheumatoid arthritis overlap cases in this group of 330 patients. The frequency of overlap cases, as seen in literature, was 13.64%. The importance of myositis-associated antibodies (MAAs) can be seen on this results: 44.44% of overlap patients, while only 7.02% of not overlap patients were MAA positive. Out of our 45 overlap patients twenty-seven myositis - RA overlap patients could be identified. Among these 27 patients the women:men ratio was 12.5:1, PM:DM ratio was 2.375:1. Muscle weakness and sceletal involvement was present in every patient, myalgia in 77.78% and Raynaud’s phenomenon in 48.15% of the patients; general symptoms during disease relapse (fatigue, weight loss, fever) were present in 59.26%, 18.52% and 14.82%, respectively. Ten cases had seropositive rheumatoid arthritis, with high titer of rheumatoid factor. Authors underline the importance of lung involvement as internal organ manifestation: 8 patients had anti-synthetase antibodies (six anti-Jo-1, one anti-PL-7 and one anti-PL-12). In anti-PL-7 and anti-PL-12 positive patients interstitial lung disease (ILD) appeared some years before myositis and early agressive management of ILD resulted in fast remission of muscle weakness. Anti-Mi-2 and anti-SRP could be detected in two patients. Most frequent used disease modifying antirheumatic drugs were cyclophosphamide, methotrexate and cyclosporine, 19 patients received DMARD combination therapy. Biological therapy was used in 3 patients, intravenous or subcutaneous immuneglobulin in 4 patients and one patient with RA – myositis – antiphospholipid syndrome died because of asipartion pneumonia. Authors conclude that it is really challenging to treat these overlap cases: they form a very heterogenous group of patients and management has to be personalized.

### P135 The Role of Muscle Biopsy Score in the Prediction of the Outcome of Adult Idiopathic Inflammatory Myopathies

#### Xin Lu, Xiaoxiao Zheng, Lining Zhang, Xiaolan Tian, Qingyan Liu, Wenli Li, Qinglin Peng, Guochun Wang

##### China Japan Friendship Hospital, Beijing, China

###### **Correspondence:** Xin Lu

Objective: The aim of the study was to investigate the relationship between the quantitative muscle biopsy score (qMBS) and prognosis of adult patients with idiopathic inflammatory myopathies (IIM).

Methods: Muscle biopsy was taken from biceps brachii muscle in 326 dermatomyositis and 89 polymyositis patients according to 1975 Bohan&Peter criteria. Muscle frozen sections were performed immunohistochemical staining of MHC-I, CD3 and CD20 molecules. According to the method of previous study on evaluating qMBS in juvenile dermatomyositis, the qMBS of adult IIM were evaluated in all patients at baseline (the time of muscle biopsy). Patients with a disease duration more than 6 months at baseline were assessed the Myositis Damage Index (MDI) according to IMACS outcome measures. All these patients were followed-up at least once then re-assessed MDI at last visit. The progression of MDI between baseline and last visit was used to represent the prognosis of IIM. Clinical data included myositis specific-autoantibodies (MSAs) status of all patients were collected.

Results: Patients with anti-TIF1-γ and anti-MDA5 antibodies had the highest and the lowest qMBS (5 ± 3.54 vs 2 ± 3.3) at baseline within all patients. However there were no statistical significant difference among all MSAs subgroups. At baseline, 285 patients with disease duration more than 6 months were assessed MDI. There was positive correlation between qMBS and baseline MDI in anti-MDA5-positive subgroup (r = 0.437, p = 0.042) and negative correlation between qMBS and MDI in male patients (r = -0.226, p = 0.039). Of 105 patients with a minimum of 6 months of follow-up were re-assessed MDI at last visit. Mean follow-up for these patients was 19 months (range 25 - 75 months). In generalized linear model, patients with higher age of onset and longer duration of follow-up had increased risk of MDI progression (B = 0.001 and 0.002, p < 0.01), respectively. Meanwhile, patients with anti-Mi-2 and anti-HMGCR-antibodies at diagnosis had decreased risk of MDI progression (B = -0.037 and -0.063, p < 0.05), respectively. However, for analysis of the correlation of qMBS and MDI outcome in combination with MSA subtype, we found that the higher qMBS at diagnosis in patients with anti-NXP2 and anti-MDA5 antibodies were associated with increased risk of MDI progression (B = 0.022 and 0.007, p < 0.05), respectively.

Conclusion: The study suggested that evaluation of qMBS in combination with MSA subtype at diagnosis may be useful to predict the long-term outcome of adult IIM.

### P136 Creatine Kinase in Systemic Sclerosis

#### Aurélien Chepy^1^, Sandrine Morell-Dubois^1^, Thomas Quémeneur^2^, Eric Hachulla^1^, Noémie Le Gouellec^2^

##### ^1^CHRU Lille Hopital Claude Huriez, Lille, France; ^2^Centre Hospitalier Valenciennes, Valenciennes, France

###### **Correspondence:** Aurélien Chepy

Context: Elevated creatine kinase CK is frequent during systemic sclerosis (SSc) and associated with reduced survival.

Objectives: To describe the characteristics of patients with SSc and elevated CK, and identify the factors associated with the persistence of this elevation.

Methods: Forty-eight adult patients with SSc and high CK were identified among 541 SSc patients in Lille internal medicine department, tertiary center for autoimmune diseases. The diagnosis of SSc was based on ACR classification criteria. CK had to be measured at least twice and be at least once above the normal range (180 IU/L for women and 195 IU/L for men). Persistent elevation of CK was defined as at least 2 elevated CK measurements. Clinical, biological and imaging data were retrospectively collected.

Results: The sex ratio was 0.76 (21 men). Sixteen patients (33%) had diffuse cutaneous SSc (dcSSc), 31 (65%) had limited cutaneous disease (lcSSc) and one patient had a sine scleroderma type of SSc. Thirty-two patients (67%) had persistent elevation of CK. The following characteristics were associated with persistent elevated CK : a shorter delay between the onset of Raynaud phenomenon and the first rise of CK (7.5 ± 11.4 vs 14.9 ± 15.1 years, p = 0.018), an elevated CK-level at SSc diagnosis (71.9 % vs 25 %, p= 0.005), dcSSc (46.9 % vs 6.3%, p = 0.008), less frequent ACA (6 % vs 50 %, p=0.018), the presence of muscle symptoms (56.3 % vs 6.3%, p = 0.001), a higher CK-level (786.2 ± 832 vs 223.5 ± 37.3 IU/L, p < 0.001) and a higher C-Reactive Protein (CRP) (15.8 ± 21.5 vs 3 ± 1.8 mg/L, p = 0.007). After a mean follow-up of 4.9 ± 3.6 years, myositis was suspected in 23 patients on the results of electroneuromyogram or muscle MRI. Muscle biopsy was performed in 16 patients in this group and found myositis in 12 cases. Myositis was not diagnosed in any of the patients with transient elevation of CK.

Conclusion: In patients with SSc, the persistence of elevated CK was predictive of the occurrence of myositis. A diffuse cutaneous subtype of SSc, muscle symptoms, high level of CK at SSc diagnosis, CK-level over double normal values and CRP-level were predictive of a persistent elevation of CK in our cohort. Patients with these characteristics should be closely monitored for possible evolution toward myositis.

### P137 PD-1 and PD-L1 inhibitors-associated myositis, Characteristic histopathological muscle pattern

#### Ana Matas^1^, Albert Selva O'Callaghan^2^, M. Noelia Antoniol^1^, Sergio Prieto-González^1^, Carlos Cabrera^1^, Noemi Reguart^1^, Gemma Vila Pijoan^2^, Josep M. Grau^1^, José C. Milisenda^3^

##### ^1^Hospital Clinic, Barcelona, Spain; ^2^Vall d'Hebron General Hospitalia, Barcelona, Spain; ^3^Hospital Clinic and IDIBAPS, Barcelona, Spain

###### **Correspondence:** Ana Matas; José C. Milisenda

Objectives: Drug-induced myopathy is one of the most common causes of muscle disease. Recently, it has been described as an association between programmed death-1 (PD-1) inhibitors and immune-related adverse events affecting different organs including skeletal and cardiac muscle. Here, we describe the clinical and pathological findings of six unrelated patients with PD-1 and PD-L1 inhibitors-associated myopathy.

Methods: We did a retrospective study, analyzing our database from January 2017 to July 2018 where 317 muscle biopsies for diagnostic purposes were made. Two myology experts took care of the patients and, performed and analyzed muscle biopsies. Muscle biopsies were frozen in cooled isopentane, cryostat sectioned and stained and reacted routinely (minimum 16 staining’s and reactions).

Results: We have identified 6 patients receiving anti-PD-1 or PD-L1 inhibitors suffering from either muscle weakness, asthenia, myasthenic syndrome-like, cardiac involvement, or other muscle related-symptoms. Muscle biopsy showed unequivocally showed an inflammatory myopathy in all of them. Furthermore, muscle biopsy showed in most of the cases a marked phenomenon of necrosis, and muscle regeneration with perivascular and endomisial inflammatory infiltrates with a large component of macrophagic cells (figure 1). Also, a tendency to perifascicular atrophy was noted in some cases. The positivity of MHC class I antigens predominated in the perifascicular zones of the affected areas. MAC (C5b9) positivity was observed either in the sarcolemma or in the cytoplasm of some cells (Table 2). To note that serum muscle enzymes were elevated in only 3 patients (Table 1).

Conclusion: Although skeletal and cardiac muscle involvement as an adverse effect of anti-PD-1 inhibitors seems to the rare, it is important their early diagnosis in order to prevent a poor outcome. In addition, the pathological picture seems to be quite different from other inflammatory myopathies, with a prominent aggregate of histiocytes.

### P138 Immune checkpoint inhibitor-associated myositis: a new and severe entity amongst immune inflammatory myopathies

#### Céline Anquetil, Joe-Elie Salem, Bénédicte Lebrun-Vignes, Douglas B. Johnson, Andrew L. Mammen, Werner Stenzel, Sarah Leonard-Louis, Olivier Benveniste, Javid J. Moslehi, Yves Allenbach

##### ., .

###### **Correspondence:** Céline Anquetil

Objectives: Immune checkpoint inhibitors (ICI) therapy is an expanding field in cancer treatment. Immune-related adverse events (irAE) can potentially affect any organ system with a different tropism depending on the therapeutic targets (Programmed cell Death 1 (PD-1), PD-ligand 1 (PD-L1) or Cytotoxic T Lymphocyte-Associated Protein 4 (CTLA-4)). ICI-associated myositis was initially reported as a rare irAE with an incidence of < 1% in the pivotal clinical trials. However, with the wide use of ICI, numerous patients harbored a new clinical and histological phenotype combining myositis, myasthenia gravis-like symptoms (especially oculomotor disorders and ptosis) and myocarditis. Importantly, ICI-associated myositis was a severe irAE with an overall mortality rate of 21.2%, increased to 51.7% in patients with concomitant myocarditis. We aimed to better characterize ICI-associated myositis patients and identify factors associated with poor outcome.

Methods: Data were collected from the VigiBase (http://www.vigiaccess.org/) and the World Health Organization database of individual safety case reports (Uppsala, Sweden) through November 2018. Cases were assessed using the High Level Term “Muscle infections and inflammations” of the Medical Dictionary for Regulatory Activities terms and at least one of seven ICIs.

Results: 277 myositis patients were identified in Vigibase with a dramatic increase in the reporting rate over the last years (before 2016, 0.5 case/month vs 11.7 cases/month in 2018). They received monotherapy in 83.4% of the cases, usually anti-PD1ICI. We confirmed the specific phenotype previously described combining cardiotoxicity (20.9%, n=58) including myocarditis (n=38) and myasthenia gravis-like symptoms (n=44). Other concurrent irAE were reported: hepatitis (n=20), colitis (n=8), nephritis (n=4), meningitis or encephalitis (n=4), thyroiditis (n=3), hypophysitis (n=3), and vitiligo (n=2). The overall mortality was 21% (n=54/257). In univariate analysis, the mortality rate was significantly higher in patients with concurrent cardiotoxicity (53.7% vs 12.3%, p< 0.0001) and in patients treated with combotherapy (anti-PD1 and anti-CTLA-4) compared to monotherapy (25.9% vs 19.6%, p=0.04). In multivariate analysis, cardiotoxicity and cancer progression were the two independent factors associated with fatal outcome (respectively, OR=9.7 p< 0.0001 and OR=15.2 p= 0.0002). The median time of myositis onset was 32 days [18.3-54.5] after the initial exposure to ICIs (data available in 88 patients). In the group of patients with fatal outcome, the median time of myositis onset was significantly shorter 18 days [14.5-23.5] vs 34 days [21-65] p=0.002.

Conclusion: Mortality in patients with ICI-associated myositis is related to cardiotoxicity and cancer progression. Moreover, severe form occurs early after the first infusion of ICI.

### P139 Mass cytometry reveals an impairment of B cell homeostasis in Anti-synthetase syndrome

#### Gaelle Dzangué-Tchoupou^1^, Yves Allenbach^2^, Corinna Preusse^3^, Werner Stenzel^3^, Olivier Benveniste^2^

##### ^1^Center of research in Myology, Paris, France; ^2^Pitié-Salpêtrière University Hospital, Paris, France; ^3^Charité Universitätsmedizin Berlin, Berlin Germany

###### **Correspondence:** Gaelle Dzangué-Tchoupou

Background: Myositis is a heterogeneous group of auto-immune disease involving several extra-muscular manifestations. Well-defined homogenous groups of myositis have been described e.g: inclusion body myositis (IBM), anti-3-hydroxy-3-methylglutaryl coA reductase (HMGCR) myopathy, anti-signal recognition particle (SRP) myopathy and the anti-synthetase syndrome (ASyS). Patients with the ASyS present antibodies directed against amino-acyl-t-RNA synthetases, amongst which histidyl t-RNA synthetases (Jo1) antibodies are the most frequent. Recent data suggest the implication of B and NK cells in the physiopathology of ASyS anti-Jo1+ patients; nevertheless their role and activation states are poorly described. In addition, the implication of other cell types is poorly described.

Objectives: Our aim was to describe precisely and deeply the immune profile of the different cell subsets potentially involved in the physiopathology of anti-Jo1+ ASyS. This could allow a better comprehension of disease mechanisms and the identification of potential therapeutic targets.

Methods: We performed deep immune-profiling using 37 markers by mass cytometry on peripheral blood cells from 10 active untreated anti-Jo1 patients. Results were compared to those of 17 healthy controls (HD) and a pool of 26 other active myositis control patients (IBM: n=10, anti-HMGCR+ myopathy: n=9 and anti-SRP+ myopathy: n=7).

Results: We observed a decrease in the frequency of memory B cells in anti-Jo1 patients (mean±SEM: anti-Jo1 = 13±3%, HD = 37±4% and other myositis = 32±3), counterbalanced by an increase in the frequency of naïve B cells (mean±SEM: anti-Jo1 = 86±3%, HD = 61±4% and other myositis = 66±3). Interestingly, immunostainings of muscle biopsies from anti-Jo1 patients show perifascicular infiltrations of memory B cells, suggesting that they niche within the muscle. Activation markers such as CD95 and HLA-DR did not show any differences within B cells of Anti-Jo1+ ASyS patients in comparison to myositis controls. In line with the literature, we also observed an increase in the frequency of activated CD57+ CD56+ NK cells in anti-Jo1+ ASyS patients compared to control groups.

Conclusion: Here, we show an impairment of B cell homeostasis in anti-Jo1+ ASyS patients in comparison to other myositis patients and healthy subjects.

### P140 Anti-Jo1 positive myositis patients display a characteristic IgG Fc-glycan profile which is further enhanced in anti-Jo1 autoantibodies

#### Catia Fernandes-Cerqueira, Nuria Renard, Antonella Notarnicola, Edvard Wigren, Susanne Gräslund, Roman Zubarev, Ingrid E. Lundberg, Susanna Lundström

##### Karolinska University Hospital, Stockholm, Sweden

###### **Correspondence:** Catia Fernandes-Cerqueira

Objectives: IgG Fc-glycans affect IgG function and are altered in autoimmune diseases and autoantibodies. Anti-histidyl tRNA synthetase autoantibodies (anti-Jo1) are frequent in myositis associated with interstitial lung disease (ILD). We tested if total IgG Fc-glycans from Jo1+ versus Jo1- myositis patients and anti-Jo1-IgG showed characteristic differences, and if particular Fc-glycan features could be associated with specific clinical manifestations.

Methods: Total IgG was isolated by affinity purification from serum of 44 myositis patients (19 Jo1+ and 25 Jo1-) and 24 age/sex matched healthy controls (HC). Anti-Jo1-IgG was further purified from eleven patients using a recombinant Jo1-coupled affinity column. A shotgun proteomics approach was used to profile serum-derived IgG-Fc-glycans and IgG-chain distributions. Uni- and multivariate statistics were used to find characteristics and correlate data with clinical information.

Results: A high abundance of agalactosylated IgG1 Fc-glycans was observed in myositis patients compared to HC. Using intra-individual normalization of the main agalactosylated glycan (FA2) of IgG1 vs FA2-IgG2, myositis and HC were distinguished with an area under the curve (AUC) of 79±6%. For Jo1+ the AUCs went up to 88±6%. Bisected and afucosylated Fc-glycans were significantly lower in Jo1+ compared to Jo1- patients. Anti-Jo1 IgG enriched from eleven patients contained even lower abundance of bisected, afucosylated and galactosylated forms compared to matched total IgG. ASS and ILD diagnosis correlated with Jo1+ characteristic Fc-glycan features via multivariate analysis.

Conclusion: The anti-Jo1+ patient IgG Fc-glycan profile contains phenotype specific features which may underlie the pathogenic role of Jo1 autoantibodies.

### P141 Association of anti-Ro52 autoantibodies with interstitial lung disease and more severe disease manifestations in juvenile idiopathic inflammatory myopathies

#### Sara Sabbagh^1^, Iago Pinal-Fernandez^1^, Takayuki Kishi^1^, Ira Targoff^2^, Frederick Miller^1^, Andrew Mammen^1^, Lisa Rider^1^

##### ^1^National Institutes of Health, Bethesda, USA; ^2^Oklahoma Medical Research Foundation, Oklahoma City, USA

###### **Correspondence:** Sara Sabbagh

Objectives: Myositis specific autoantibodies (MSA) and myositis associated autoantibodies (MAA) in adult and juvenile idiopathic inflammatory myopathies (JIIM) confer specific phenotypes. In adults, anti-Ro52 autoantibodies (Abs) are associated with anti-synthetase (ARS) Abs and severe interstitial lung disease (ILD). There are few studies examining anti-Ro52 Abs in JIIM. Our purpose was to define the prevalence and clinical features of anti-Ro52 Abs in a large cohort of JIIM patients.

Methods: Sera from 307 patients with juvenile dermatomyositis (JDM), 26 patients with juvenile polymyositis (JPM), and 44 patients with juvenile connective tissue disease-myositis overlap (JCTM) were screened for anti-Ro52 Abs by ELISA. Clinical characteristics were compared between patients with and without anti-Ro52 Abs.

Results: Anti-Ro52 Abs were detected in 14% of JIIM patients. Anti-Ro52 Abs were more common in patients with ARS Abs (64%, p < 0.001) and anti-MDA5 Abs (31%, p < 0.008). After controlling for the presence of MSAs, anti-Ro52 Abs were highly associated with ILD (36% vs 4%, p < 0.001), dyspnea on exertion (59% vs 25%, p < 0.001), and higher pulmonary scores at diagnosis (median 0.18 vs 0.08, p=0.004). Among anti-MDA5+ patients, Ro52 reactivity was associated with ILD: 70% (7/10) of anti-Ro52+ and 9% (2/22) of anti-Ro52- patients had ILD. Among ARS+ patients, 100% (9/9) of anti-Ro52+ and 40% (2/5) of anti-Ro52- patients had ILD (p=0.03). Patients with co-existing anti-p155/140 and anti-Ro52 Abs also had an increased frequency of ILD (15% vs 1%, p=0.03) and dyspnea on exertion (50% vs 16%, p=0.01) compared to anti-p155/140 positive patients who were anti-Ro52 negative. Anti-Ro52 Abs were also associated with more severe disease. Anti-Ro52+ patients more often had chronic continuous (78% vs 52%, p=0.05) and less often monocyclic disease course (3% vs 24%, p=0.02). Anti-Ro52+ patients were more often ACR functional class IV (11% vs 4%, p=0.008). Anti-Ro52 Abs were associated with an increased total number of medications received (mean 4.8 vs 3.8, p=0.04) and anti-Ro52+ patients more often received intravenous pulse steroids (79% vs 52%, p=0.03). Anti-Ro52+ patients less frequently had a remission (5% vs. 27%, p=0.05). Anti-Ro52 Abs had a weak association with HLA DRB1*1402 and DQA1*0503, which lost significance after adjustment for multiple comparisons.

Conclusions: Anti-Ro52 Abs are most prevalent in juvenile myositis patients with anti-MDA5 and ARS Abs. Even after adjusting for the presence of MSAs, anti-Ro52+ patients were more likely to have ILD and other pulmonary manifestations. Anti-Ro52+ patients have more severe disease with poorer outcomes.

### P142 Anti-Jo-1 antibody-positive patients show different clinical symptoms, histological features and molecular gene expression compared to anti-PL-7 and anti-PL-12 antibody-positive patients

#### Barbara Paesler, Laure Gallay, Yves Allenbach, Olivier Benveniste, Nathalie Streichenberger, Werner Stenzel, Corinna Preuße

##### Charité Universitätsmedizin Berlin, Berlin, Germany

###### **Correspondence:** Barbara Paesler

Objective: To compare skeletal muscle biopsies of patients with the three most common anti-synthetase syndrome-associated antibodies, using modern myopathologic methods in order to define common or divergent pathomechanisms.

Methods: Skeletal muscle biopsies from patients carrying one out of three anti-synthetase syndrome–associated antibodies Jo-1 (n=16), PL-7 (n=10) or PL-12 (n=13) were analyzed by histopathology, quantitative PCR, and electron microscopy.

Results: Evaluating the clinical history of 39 patients with anti-synthetase syndrome we discovered that anti-Jo-1 positive patients show eminently higher disease activity and more severe muscle symptoms than anti-PL-7 or anti-PL-12 positive patients. Thus, arthralgia and mechanic hands occurred more often, while anti-PL-7 or anti-PL-12 positive patients rather tended to show lung involvement and fever. All three groups showed similar histological features in typical topographic distribution, e.g. atrophic and necrotic myofibers in the perifascicular region surrounded by infiltrating immune cells and prominently fragmented connective tissue. However, it became apparent that anti-Jo-1 positive patients showed a more severe and acute clinical picture than PL7/PL12+ patients. In keeping with this, qPCR analyses showed an up regulation of genes involved in a proinflammatory immune responses such as INFγ, TNFα, IL-6, IL-1b and STAT1 in muscle tissue derived from anti-Jo-1+ patients, whereas muscle from anti-PL-7+ and PL-12+ patients showed a significantly increased expression of cytokines providing rather profibrogenic and immune regulatory effects such as TGFβ and IL-4R. Of note, considerable amounts of plasma cells occurred in the skeletal muscle tissue of all three groups, accompanied by elevated levels of chemokines (CXCL12, CXCR4) and transcription factors (IL-6, APRIL, BAFF) that attract plasma cells and regulate their function. On the ultrastructural level, Jo1+ patients harbored intranuclear actin aggregates in about 75% whereas in PL7, and PL12+ patients they occurred more rarely.

Conclusion: Anti-Jo-1 antibody-positive patients show distinct clinical features and their muscles appeared more severely affected as compared to muscle biopsies from anti-PL7 & -PL-12-positive patients. Conversely anti-PL-7 or anti-PL-12 positive patients appeared very similar in terms of milder skeletal muscle affection and non-muscular symptoms such as lung involvement. The striking occurrence of plasma cells in affected skeletal muscle tissue is overt in all three antibody groups and may therefore be considered as a characteristic and diagnostic feature of anti-synthetase syndrome-associated myositis. Based on their molecular profile we hypothesize that they may use the skeletal muscle as a survival niche.

### P143 Hand Radiographic Lesions Are Frequent in Antisynthetase Syndrome Arthritis and Are Associated with a Longer Duration of the Disease

#### Cerise Guillochon-Petitcuenot^1^, Gwenael Balazuc^1^, Thibault Willaume^1^, Guillaume Bierry^1^, Francois Maurier^2^, Naji Afif^3^, Laurent Messer^4^, Jacques-Eric Gottenberg^1^, Jean Sibilia^1^, Alain Meyer^1^

##### ^1^Strasbourg University, Stasbourg, France; ^2^Hôpitaux Privés de Metz, Metz, France; ^3^Centre hospitalier de Mulhouse, Mulhouse, France; ^4^Hôpitaux civils de Colmar, Colmar, France

###### **Correspondence:** Alain Meyer

Objectives: to report prevalence, characteristics and risk factors of radiographic abnormalities in antisynthetase syndrome (ASS) patients with joint involvement.

Methods: Mono-centric cohort of 101 patients with ASS was screened for hand X-ray availability. All the X-rays were read by two radiologists experienced in osteoarticular diseases. Patients with osteoarthritic lesions, chondrocalcinosis, gout or ACPA positive test were excluded from the analysis. ASS patients with hand radiographic lesions were compared to ASS patients with normal hand Xray.

Results: Twenty-four patients had at least one available hand X-ray, three were excluded because of osteoarthritic lesions or ACPA positivity. Thus, twenty-one were included followed during nineteen years (399 patient-years). Patients had history of arthralgia (n= 19), arthritis (n=15). Twelve patients (57.1%) had normal hand X-ray(s), while nine (42.9%) had radiographic lesions including: periarticular calcifications (77.8%), joint pinching (66.7%), subluxations (66.7%), erosions (22.2%) and demineralization (11.1%). Sex-ratio and age at diagnosis were not significantly different between the two groups. However, duration of ASS was much longer in the abnormal Xrays group (11 years IQR 5-18.5; vs 2 years IQR 0.5-6.5, p = 0.02), as was the duration of joint involvement (8 years IQR 2.3-15.5; vs 2 years IQR 0-5, p = 0.06). History of extra-articular involvements (fever, myositis, interstitial lung disease, Raynaud’s phenomenon, sclerodactylia, mechanic’s hands) did not differ between the two groups, except for calcinosis that tended to be more frequent in case of radiographic lesion (33.3 % vs 0%, p = 0.06). Distribution of anti-tRNA synthetase antibodies (anti-Jo1 n=15, anti-PL7 n=2, anti-PL12 n=1, anti-EJ n=3 and anti-OJ n=1) was similar in the two groups. Rheumatoid factor tended to be more frequently positive in case of radiological abnormality (22.2 % vs 9.1 %, p = 0,57). Despite much higher disease duration, presence of persistent joint symptoms was similar in both groups (one third). However, patients with radiographic lesions did not received significantly higher number of immunomodulatory drugs (1 IQR 0-2.8; vs 1 IQR 0-1.5, p = 0.48) and their dosage of corticosteroids at diagnosis of hand radiographic lesions tended to be higher than the patients without hand radiographic lesions at last X-ray (5 mg IQR 0-7.5; vs 1 mg IQR 0-6.5, p = 0.37). Handicap, evaluated by Health Assessment Questionnaire, tended to be higher in patients with radiographic lesions (0.85 IQR 0.3-1.2; vs 0.45 IQR 0.1-0.9, p=0,49).

Conclusion: Hand radiographic lesions are frequent in ASS-arthritis and increase with duration of the disease.

### P144 A rare case of anti-PL12 syndrome and Amyotrophic Lateral Sclerosis

#### Ejaz Shamim^1^, Loraine Yarngo^1^, Abigail Rapp^2^, Christabel Chan^2^, Melissa Sinkiewicz^1^, Melvin Kong^1^, Ivan Lim^1^

##### ^1^Kaiser Permanente, Potomac, USA; ^2^The George Washington University, Washington, USA

###### **Correspondence:** Ejaz Shamim

Background: Idiopathic inflammatory myopathies (IIM) are a rare group of systemic muscle disorders. Amyotrophic Lateral Sclerosis (ALS) is the commonest of the motor neuron disorders. It is extremely rare that the two conditions occur together.

Objective: Here we describe a unique patient with IIM and ALS. Methods: in our neuromuscular multidisciplinary clinic in Kaiser Permanente MidAtlantic States, we follow patients with ALS and IIM from Washington, DC, Maryland and Virginia. This is a very rare case that has not been reported in the past.

Results: The patient is a left hand African American Male who initially presented to rheumatology department with joint swelling. His anti-CCP and anti-SSA were mildly elevated with elevation in the CK. Electromyographic (EMG) studies suggested chronic neurogenic changes in several cervical and lumbar nerve roots affecting distal and proximal muscles. Myopathic changes were also noted. MRI of the lumbar and cervical spine indicated degenerative joint disease. He started to develop mild proximal muscle weakness. He also started to have shortness of breath on mild exertion. Myositis specific antibody panel was sent as he also had mild proximal muscle weakness, revealing the presence of anti-PL 12 antibody. The result was confirmed. His pulmonary function tests were normal and continue to be normal. For the shortness of breath, no etiology was found. The patient had an EMG of the diaphragm which was suggestive of ALS. He had a muscle biopsy of the right thigh. It revealed chronic inflammation with scattered atrophic and necrotic muscle fibers. His muscle biopsy was sent for another opinion. In the meantime, he is being treated for ALS and for myositis. Compared to other ALS patients in our clinic, his progression is slower than expected.

Conclusion: Here we describe a rare instance of patient who has been diagnosed with ALS and meets Bohan Peter criteria for probably myositis with anti-PL 12 syndrome. He is being treated for both conditions. In a recent population-based study from Taiwan, 6 of more than 9,000 IIM patients with polymyositis had ALS. None were noted to have anti-synthetase syndrome.

### P145 Diagnostic value of perifascicular HLA-DR expression in anti-synthetase syndrome associated myopathy

#### Jantima Tanboon, Michio Inoue, Ichizo Nishino

##### National Center of Neurology and Psychiatry, Tokyo, Japan

###### **Correspondence:** Jantima Tanboon

Objective: To assess diagnostic value of perifascicular (PF) HLA-DR expression for anti-synthetase syndrome associated myopathy (ASM)

Method: We retrospectively review 565 consecutive muscle biopsies with known associated myositis specific autoantibody (MSA) diagnosed as ASM (n=175), Dermatomyositis (DM, n=101) and immune mediated necrotizing myopathy (IMNM, n= 289) in National Center of Neurology and Psychiatry (NCNP), Tokyo, Japan. Immunohistochemical study including HLA-ABC, HLA-DR, C5b-9, and MxA are available in all cases. ASM and IMNM include in study cohort are negative for MxA. Inclusion body myositis (IBM) is not included in this study. We evaluate HLA-DR expression pattern by classifying it into 5 categories: 0 = negative staining, 1 = scattered or patchy HLA-DR staining muscle fibers without definite PF localization, 2 = a few to several discontinuous HLA-DR positive fibers with PF localization, 3 = small number of (2-5) continuous HLA-DR positive fibers with PF localization, 4 = large number of continuous HLA-DR positive fibers with PF localization involving at least one side of a fascicle, 5 = large continuous HLA-DR positive fibers with PF localization plus > 10 positive fibers scattered in fascicles. We postulate significant PF expression for category 4 and 5.

Result: HLA expression is most commonly present in ASM (54.3%, 95/175) follow by DM (27.9%, 28/101) and IMNM (4.8%, 14/289: anti-SRP IMNM 5/170 and anti-HMGCR IMNM 9/119), respectively. Category 4 and 5 expressions are most commonly present in ASM (38.3%, 67/175) and occasionally and rarely present in DM (7.9%, 8/101) and IMNM (0.3%, 1/289), respectively. Anti-Jo1 associated myopathy is the most common ASM subtype associated with HLA-DR expression (60.7%, 34/56).

Conclusion: Perifasicular HLA-DR expression may help narrow scope of muscle pathology differential diagnosis to ASM especially anti-Jo-1 associate myopathy in MxA negative case.

### P146 Interstitial lung disease in Idiopathic Inflammatory Myopathies: ultrasound assessment of pleural irregularities and comparison with clinical, serological and functional aspects

#### Elisa Cioffi, Simone Barsotti, Chiara Romei, Alessandra Tripoli, Claudia Roncella, Chiara Cardelli, Marts Mosca, Fabio Falaschi, Rossella Neri

##### University of Pisa, Pisa, Italy

###### **Correspondence:** Elisa Cioffi

Background: Interstitial Lung Disease(ILD) is one of the most frequent and significant extra-muscular manifestations in Idiopathic Inflammatory Myopathies(IIM). Recently, several authors proposed ultrasound(US) quantification of pleural irregularities(PIs) as a possible method to detect ILD in IIM patients. The objective of our study was to evaluate the prevalence of PIs and to compare the results obtained to clinical symptoms/signs, autoantibodies, high resolution chest TC(HRCT) and Pulmonary Function Test(PFT).

Methods: 45 patients(30 females, 15 males, mean duration disease 54.4 ±86.1 months, median age 63.6±11.9 years) with a diagnosis of IIM according to Bohan and Peter criteria (19PM, 20DM, 1MCI, 3overlap and 1paraneoplastic form) who required HRCT evaluation were enrolled. Data collected included presence of anti-synthetase autoantibodies(ASS), dyspnoea assessment by means MRC scale, PFT parameters, questionnaires as Leicester Cough Questionnaire(LCQ) and Saint George Respiratory Questionnaire(SGRQ). Thoracic US evaluation in 53 anterior and posterior thoracic areas was performed using a high frequency 8-18MHz linear probe. We evaluated the pleural profile aspect assigning each space a score (regular=0, mild irregular=1, irregular=2), the sum of the scores at anterior, posterosuperior, posteroinferior and the total PIs score were calculated. HRCTs were assessed by an expert radiologist for the presence of signs of ILD.

Results: The mean PIs total score obtained with lung US was 26.6±13.9(anterior 10±6.5, posterosuperior 4.8±4.4 and posteroinferior 11.7±6). Patients with ILD at HRCT had a PIs total score significantly higher than patients without ILD (p= 0.003) and it was true also for the singular lung sites, especially for the posteroinferior (p= 0.001) and posterosuperior (p= 0.032) areas. Dyspnoea more than2 MRC scale was related with a different mean PIs total score compared to dyspnoea less-equal 2MRC scale (p=0.01). The presence of oough and fine crackles at the thoracic auscultation were associated to higher PIs score (respectively p= 0.049 and p=0.001), particularly in posterosuperior areas. Also, the LCQ significantly correlated with the PIs total score (p=0.02, r=-0.448), particularly in the posterosuperior areas(p=0.02,r=-0.446). Patients with ASS (p= 0.002) and particularly anti-Jo1 (p= 0.001), showed a PIs total score significantly higher than patients without ASS. FEV1(p=0.001, r=-0.591), TLC(p=0.001,r=-0.695) and LCO(p=0.004,r=-0.532) significantly correlated to the US PIs score.

Conclusions: The US PIs score is correlated to presence of ILD, clinical, serological and functional parameters in patients with IIM-related ILD and may improve the bedside evaluation of the patient in the routine clinical practice. Further studies are needed in particular to assess the prognostic value of lung ultrasound in IIM

### P147 A case of myositis with positive anti-PM/Scl antibody

#### Suur Biliciler, Ali Reza Shoraka

##### UTHealth Science Center, Housten, USA

###### **Correspondence:** Suur Biliciler

Introduction: Even though Anti-PM/Scl is a common marker of either systemic sclerosis or its overlap syndromes pathological findings in muscle and clinical presentation of patients harboring this antibody is very variable.

Case Presentation: We present a case of 71 year-old female with subacute progressive weakness which started in legs then progressed to arms and face over three months. She developed head drop within six months of presentation. Patient was found to have telangiectasias, weakness upper more than lower extremities, proximal more than distal, head drop and bilateral scapular winging, scaly finger tips with no ulcers or necrosis. On further workup, she showed myopathic changes in EMG and muscle biopsy showed dense collections of inflammatory cells in a nodular fashion. Laboratory work up showed elevated CK, ESR, high titer ANA and positive double-stranded ANA, rheumatoid factor and anti-PM/Scl antibody. Genetic testing was negative for known muscular dystrophies including fascioscapulohumeral dystrophy. Although voltage gated potassium channels complex antibody was found in serum, malignancy workup was negative. She was started on steroid treatment followed by addition of steroid sparing agents. Her lower extremity weakness improved significantly in lower extremities with some improvement in arm and neck muscles. Our case did not meet criteria for systemic sclerosis.

Conclusions: The fact that our patient has prominent cervico-brachial weakness is compatible with previous cases. There are few studies that show anti-PM/Scl antibody prevalence in patients with no definite systemic sclerosis upon presentation. Such cases are important to determine and diagnose to improve our knowledge about disorders related of positive anti-PM/Scl antibody.

### P148 Anti-tRNA Synthetase and PmScl antibody status is associated with distinct immunophenotypes irrespective of clinical classification

#### Erin Wilfong, Katherine Vowell, Leslie Crofford, Peggy Kendall

##### Vanderbilt University, Nashville, USA

###### **Correspondence:** Erin Wilfong

Introduction: In 2015, the ATS/ERS published preliminary classification criteria for interstitial pneumonia with autoimmune features (IPAF) with a goal of identifying patients with interstitial lung disease who might benefit from immunosuppression. The inclusion of the myositis associated antibodies – particularly the anti-tRNA synthetase (ARS) and anti-Pm/Scl antibodies – has been debated as clear systemic syndromes have been described for these antibodies. Here, we aimed to investigate the immunophenotypes of patients with both ARS and anti-Pm/Scl antibodies and ascertain if immunophenotypes correlated with clinical classification criteria or serologic status.

Method: Peripheral blood mononuclear cells (PBMCs) were collected from 12 patients with idiopathic inflammatory myopathy (IIM) or IPAF; patients were phenotyped by chart abstraction. Clinical classification was performed using the 2015 IPAF and 2017 ACR/EULAR IIM criteria. 18 healthy controls were used for comparison. Cryopreserved PBMCs were subjected to cytometery by time of flight (CyTOF) to investigate B, T, and innate cell subsets. Analysis was performed in Cytobank using unsupervised multiparameter methods as well as traditional biaxial gating. Statistical significance was determined with Mann-Whitney tests.

Result: 6/12 patients met classification criteria for IIM; the remainder met criteria for IPAF. Serologically, 5 patients were anti-PmScl positive, 2 were anti-Jo1 positive, 2 were anti-PL7 positive, 2 were anti-PL12 positive, and 1 was anti-EJ positive. All patients required escalation of immunosuppression at enrollment due to active disease. Compared to healthy controls, several B- and T-cell populations were decreased for all clinical and serologic statuses, including class-switch and unclass-switched memory B-cells, T follicular helper cells, and CXCR5+PD1- central memory CD4+ T-cells. Patients with anti-Jo1 or anti-PmScl antibodies had increased frequency of classical monocytes compared to non-Jo1 ARS patients or healthy controls (27.5% vs. 12.61% and 7.73%, p=0.03 and p=0.002). Compared to other positive serology and healthy controls, patients with anti-Jo1 antibodies had increased frequency of CXCR4+CD21lo B-cells (53% v. 1.6% and 0.79%, p=0.03 and p-0.02), CD8+CD11c+CD38- T cells (12.84% vs. 0.2% and 0.2%, p=0.03 and p=0.01), and CD4+CXCR4hi naïve T cells (21.7% vs. 0.29% and 0.01%, p=0.03 and p=0.01).

Conclusion: Various anti-tRNA synthetase and anti-PmScl antibodies are associated with distinct immunophenotypes. These immunophenotypes may serve as biomarkers if verified in larger cohorts and may also inform treatment strategies in patients who have similar clinical phenotypes but divergent underlying immunologic pathophysiology.

### P149 Autoantigen Histidyl-tRNA Synthetase (HisRS) is secreted into circulation and inversely correlates with anti-HisRS autoantibody levels

#### Catia Fernandes-Cerqueira^1^, Ryan Adams^2^, Antonella Notarnicola^1^, Andrea Cubitt^2^, David J. King^2^, Ingrid E. Lundberg^1^, Per-Johan Jakobsson^1^, Leslie A. Nangle^2^

##### ^1^Karolinska Universty Hospital, Stockholm, Sweden; ^2^aTyr Pharma, San Diego, USA

###### **Correspondence:** Catia Fernandes-Cerqueira

Objectives: Histidyl-transfer RNA synthetase (HisRS) is a cytoplasmic enzyme and a major autoantigen in idiopathic inflammatory myopathy (IIM) and anti-synthetase syndrome (ASS). We investigated if HisRS is detectable in circulation and whether the free extracellular HisRS levels correlate with anti-HisRS autoantibodies.

Methods: HisRS and anti-HisRS autoantibodies were quantified in sera from 357 IIM/ASS patients (61 anti-HisRS+ and 286 anti-HisRS-) and 115 age/gender matched healthy controls (HC). Levels of HisRS antigen were measured by an immunoassay employing two non-competing monoclonal antibodies targeting the N-terminal (WHEP) domain of HisRS. Anti-HisRS autoantibodies were accessed by an immunoassay which used a commercial N-terminal HARS monoclonal antibody. Interstitial lung disease (ILD) status was present in 51 of 70 anti-HisRS+ IIM/ASS patients.

Results: HisRS was detectable in circulation with higher levels in patients with IIM/ASS compared to HC. When subgrouping patients into autoantibody status (Jo1+ vs Jo1-) significantly lower levels of serum HisRS (3 pM) were detected in anti-HisRS+ IIM/ASS patients, compared to anti-HisRS- IIM/ASS and matched HC and (13 pM and 8 pM, respectively p < 0.001). Levels of free HisRS in HC were also significantly lower in comparison to the anti-HisRS- IIM/ASS group (p < 0.001). IIM/ASS diagnosed with ILD had significantly lower serum HisRS levels (6 pM) compared to IIM/ASS patients without ILD (11 pM, n=209). This observation may reflect the fact that a large majority of anti-HisRS+ IIM/ASS were diagnosed with ILD (86%). Although the low number of anti-HisRS+ IIM/ASS patients without ILD in our cohort (n=7), we nonetheless observed slightly elevated serum HisRS levels in this group (5 pM), compared to 3 pM in anti-HisRS+ IIM/ASS diagnosed ILD (n=44). Presence of anti-HisRS autoantibodies in serum of anti-HisRS+ IIM/ASS individuals correlated negatively with levels of free HisRS (Spearman correlation r=-0.57; p < 0.0001).

Conclusion: The identification of HisRS autoantigen in serum, and the inverse correlation between levels of HisRS and cognate anti-HisRS antibodies in circulation may explain the access autoantibodies have to intracellular targets. Free extracellular HisRS may exert unknown non-canonical functions which may be impaired in Jo1+ IIM/ASS due to neutralization by the anti-HisRS autoantibodies, and consequently contributing to IIM/ASS pathogenesis.

### P150 Course of interstitial lung disease in antisynthetase syndrome with immunosuppressive therapy

#### Linn Riedel, Jens Schmidt, Jan-Gerd Rademacher, Radovan Vasko, Peter Korsten

##### Universitätsmedizin Göttingen, Göttingen, Germany

###### **Correspondence:** Peter Korsten

Objectives: The antisynthetase syndrome (ASS) is characterized by the presence of myositis, fever, arthritis, raynaud’s phenomenon, mechanic’s hands and interstitial lung disease (ILD). While not present in all patients, ILD represents a major organ complication, potentially leading to pulmonary fibrosis and increased mortality. While various treatment regimens have been used, there are no consensus guidelines on how to optimally treat patients with ASS-ILD. Therefore, we aimed to analyze the disease course of patients at our center.

Methods: This is a single-center retrospective observational trial of prospectively collected routine medical data. Patients were identified by the presence of anti-tRNA-synthetase-antibodies (ARS) through our laboratory’s database. Patients were included if ILD was present and if sufficient data were available for further analysis. Immunosuppressive treatment regimens were recorded. As outcome measures, we analyzed creatinine kinase levels and pulmonary function tests (PFT, forced vital capacity, FVC, and diffusion capacity of carbon monoxide, DLCO) at various time points (0-6, 7-12, and >12 months after first diagnosis). Also, we quantitively assessed pulmonary fibrosis/infiltrates with Pixmeo OsiriX Lite software on computed chest tomography (CT) scans before and during treatment.

Results: 27 patients with ASS were identified. Of these, 13 were excluded because they did not have ILD. Of the remaining 14 patients (51,8%) 10 were female (71,42%), and 4 were male (28,57%). Median disease duration was 51 months, follow-up was 21 months. Of these, 12 (85,71%) received treatment with glucocorticosteroids, 7 (50%) azathioprine, 6 (42,9%) methotrexate, 1 (7,1%) mycophenolate, 2 (14,3%) leflunomide, 6 (42,9%) rituximab, 3 (21,4%) cyclophosphamid, 1 (7,1%) cyclosporine, and 1 (7,1%) tocilizumab. Median CK level at diagnosis was 673,5 U/L, which decreased to 78,5 U/L, 78 U/L, and 83 U/L after 6, 12 and >12 months, respectively. Pulmonary function testing was only inconsistently assessed. Quantitative fibrosis score analysis on CT scans showed a marked decrease during treatment.

Conclusions: Our data suggest that immunosuppressive treatments are effective in reducing CK levels. ILD improved or stabilized in all but one patient. CT scans showed a reduction of areas with ILD in the majority of patients undergoing immunosuppressive therapies. Our data suggest that a standardized fashion of ILD assessment in myositis patients is warranted. Glucocorticoid treatment is effective in improving CK levels and ILD findings. However, all patients required additional immunosuppression, mostly with azathioprine or rituximab, to achieve an improvement of ILD on chest imaging.

### P151 Association between pathological features of skeletal muscle and clinical phenotypes in anti-synthetase syndrome

#### Xiaolan Tian, Lining Zhang, Hhongxia Yang, Yamei Zhang, Wenli Li, Qingyan Liu, Qinglin Peng, Xin Lu

##### China-Japan Friendship Hospital, Beijing, China

###### **Correspondence:** Xiaolan Tian

Objective: To analyze the characteristics of skeletal muscle involvement and muscle pathology in patients with anti-synthetase syndrome (ASS).

Methods: 486 patients with idiopathic inflammatory myopathy (IIM) diagnosed in China-Japan Hospital from May 2008 to March 2018 were retrospectively analyzed. All patients underwent muscle biopsy. According to the pathological characteristics of muscle biopsy, they were divided into six pathological types: pathologic DM (pDM), pathologic PM (pPM), unspecified myositis(USM), necrotizing myositis (NAM), and MHC-I group and the normal pathological group. Immunoblotting was used to detect myositis specific antibody (MSA) .The relationships between the clinical features of different anti-synthetase antibodies and the pathological features of muscle biopsy were analyzed.

Results: Among 486 IIM patients, 77 (15.8%) were positive for anti-ARS antibody, 55 (70.5%) were female, the onset age was 50.1±11.6 years, and the disease course was 21.8 ±42.7 months. The most commonly seen subgroup was the Anti-Jo-1 antibody positive patients (36 cases, 7.4%), while the anti-PL-7 positive patients with 22 cases (4.5%), anti-PL-12 positive patients with 9 cases (1.9%), anti-EJ positive patients with 10 cases (2.1%) and no anti-OJ antibody positive patients. There was no significant difference in the characteristics of muscular and extramuscular involvement between the groups. The most commonly seen pathological type of muscles was USM (37 cases, 48.1%), followed by pDM (14 cases, 18.2%), the normal pathological group (13 cases, 16.9%), NAM group (10 cases, 13.0%) and MHC-I group (3.9%), and no patients of pPM. There was no statistical difference in the pathological characteristics of muscle among the antibody subgroups. There were 16 CADM patients and 61 non-CADM patients. The proportion of patients with normal muscle pathology in the former group was higher than that in the latter group. Perifascicular lesions including perifascicular atrophy and perifascicular necrosis were rare in patients with ASS.

Conclusion: ASS is a subtype of IIM with relatively high homogeneity. Muscle involvement is common and the pathological characteristics of muscle are not specific.

### P152 The experience of Juvenile Dermatomyositis described through poetry

#### Polly Livermore, Lucy R. Wedderburn, Faith Gibson

##### Great Ormond Street Institute of Child Health, London, UK

###### **Correspondence:** Polly Livermore

Objectives: Juvenile Dermatomyositis (JDM) is a rare, potentially life-threatening condition with no known cure. There is no research to date that documents how children and young people feel about their condition, from their perspective. This study asked children and young people what it is like to have JDM. Presented here are their experiences told through the medium of poetry.

Methods: Interpretive Phenomenology interviews aided by creative methods, were used to capture the lived experience. Data were obtained from 15 young people with JDM, between 8 and 19 years of age from one tertiary institution’s Paediatric Rheumatology department in the UK using audio-taped interviews. Data were analysed phenomenologically, using a process that derives narratives from transcripts resulting in a phenomenon which is evident in the experiences shared. The data were crafted into stories. During this process, the words of the young people were shaped into ‘research poems’ which illustrate the real emotions and experience of children and young people living with JDM.

Results: Fifteen stories were crafted, with one phenomenon and five shared narratives for each of the five themes identified. Eleven individual poems have been constructed. Titles of which reflect these experiences, such as ‘Tomato face’ and ‘The day my JDM came to town’ An example of a poem follows: Worry “You give me a name that I can’t say, But I have to explain what it is every day, I’m 10 now, but I worry about what my JDM is doing to me, It’s not easy to see, But I know it’s there, Inside me”.

Conclusion: This study is the first of its kind to ask young people to talk about what it is like to have Juvenile Dermatomyositis. This process of reducing the transcripts into poetry will be discussed and all the poems will be presented for the first time as a powerful method of sharing real life voices from young people themselves. The results will aid clinicians to gain a deeper understanding of what daily life is like for these young people, and provide ideas on how to enhance psychosocial functioning. Through sharing these poems it will encourage health professionals to consider the impact JDM has, not only on the physical health, but more importantly, the impact on a young person’s psychological well-being.

